# Sequence-Dependent Folding of Recognition-Encoded Melamine Oligomers

**DOI:** 10.1021/jacs.6c07701

**Published:** 2026-07-01

**Authors:** Anca-Luiza Cotîrlan, Cecilia J. Anderson, Nia E. J. Eyre, Daniil O. Soloviev, Ben Iddon, Federica Balduzzi, Christopher A. Hunter

**Affiliations:** Yusuf Hamied Department of Chemistry, 2152 University of Cambridge, Lensfield Road, Cambridge CB2 1EW, U.K.

## Abstract

Recognition-encoded melamine oligomers (REMO) equipped with complementary 4-nitrophenol (**D**) and phosphine oxide (**A**) side chains show sequence-dependent folding properties that resemble the folding of single-stranded nucleic acids. Automated solid phase synthesis was used to obtain a series of REMO with an **A** at one end of the chain and a **D** at the other. The two complementary recognition units were separated by 0–8 blanks (**O**, triazines equipped with alkyl side chains). UV–vis absorption denaturation experiments with perfluoro-*tert*-butanol in dichloromethane solution were used to quantify intramolecular 4-nitrophenol·phosphine oxide base-pairing in these oligomers. 1,2-Folding was not detected, but the other oligomers populate 50–90% of the folded state depending on sequence. Another series of oligomers with two complementary recognition units at each end of the chain was used to investigate folding of hairpin loop and helical structures. In these systems, the folded single strand makes two base-pairing interactions, so folding is governed by the effective molarities for the two intramolecular H-bonds, EM_f_, and a cooperativity parameter, α. At least four looped-out bases are required to form a stable hairpin: two hairpin sequences, **ADO**_**4**_ **AD** and **ADO**_**5**_ **AD**, showed strong positive allosteric cooperativity (α > 10) with 96% population of the folded state. The helical sequence, **DAO**_**4**_ **AD**, which showed strong positive cooperativity is consistent with a hexagonal grid model for the conformation of the backbone. These sequence–structure relationships show that favorable folding motifs arise when the positions of complementary recognition sites align with the conformational preferences of the triazine-piperazine backbone.

## Graphical abstract

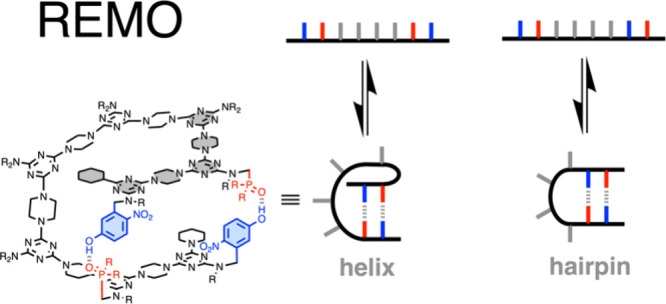

## Introduction

The functional properties of polypeptides and single stranded nucleic acids are encoded in the sequence of different side chains attached to a polymer backbone.^1,2^ Sequence-dependent folding of these polymers into highly organized three-dimensional structures leads to selective binding and catalytic properties.^3–10^ In recent years, progress has been made in the development of synthetic oligomers that fold in a predictable manner, and there are some examples that show selective recognition of substrates and catalytic activity.^11–14^ In general, the folding properties of these synthetic systems are defined by the structure of the backbone that leads to specific conformational preferences.^15–17^ Here we describe a different class of synthetic foldamers that have a uniform backbone and folding properties that are encoded by the sequence of different side chains.

Figure 1 shows the chemical structure of a recognition-encoded melamine oligomer, or REMO.^18^ The uniform backbone is made of alternating 1,3,5-triazine and piperazine units, and the sequence of each oligomer is defined by side chains that carry phosphine oxide or phenol recognition units. H-bond base-pairing interactions between phenol (**D**) and phosphine oxide (**A**) recognition units lead to sequence-selective duplex formation between complementary sequences:^18^ Figure 1 shows the structure of the **DAA·ADD** duplex, and REMO duplexes with up to 12 base-pairs have been characterized.^19^ We use the term base-pair to describe the phenol·phosphine oxide H-bonding interactions by analogy with the purine·pyrimidine H-bonding interactions in nucleic acids.

**Figure 1:**
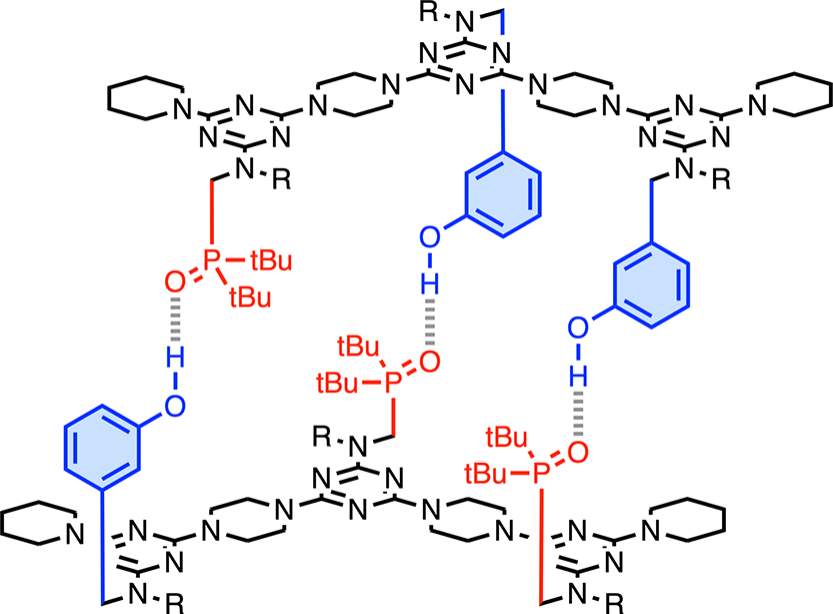
H-bonded duplex formed by two sequence-complementary recognition-encoded melamine oligomers (REMO) due to phenol·phosphine oxide base-pairing interactions. R = solubilizing groups.

Mixed sequence REMO 3-mers form sequence-selective duplexes with high fidelity (Figure 2a),^18^ but for the self-complementary 4-mer **DADA**, intramolecular base-pairing interactions between the terminal recognition units lead to 1,4-folding of the single strand and reduce the stability of the duplex (Figure 2b).^20^ Intramolecular folding equilibria are likely to become more important in longer mixed sequence oligomers, and exploitation of the REMO architecture will require a better understanding of the interplay between folding and duplex formation. Here we introduce a method for quantifying the folding properties of REMO and show that particularly stable folds are found for specific sequence motifs.

**Figure 2:**
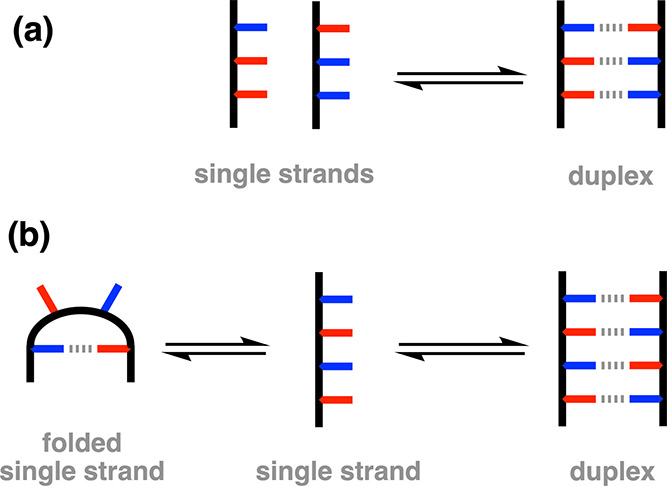
(a) Cartoon representation of formation of the mixed sequence 3-mer duplex **AAD**·**DDA**. (b) For the self-complementary 4-mer **DADA**, intramolecular 1,4-folding interactions in the single strand compete with duplex formation.

## Results and Discussion

### Approach

We recently introduced an automated solid phase synthesis approach to REMO.^21^ This methodology allows us to address the folding of REMO in a systematic manner. Figure 3 shows a family of oligomers that have a phosphine oxide recognition unit (**A**) at one end and a complementary 4-nitrophenol recognition unit (**D**) at the other end, separated by variable numbers of blanks (**O**), which are triazines equipped with solubilizing groups instead of recognition units. In these **AO**_**n**_ **D** oligomers, only one intramolecular H-bonding interaction is possible, and the UV–vis absorption of the 4-nitrophenol moiety provides a direct readout of H-bond formation. Here we show how these oligomers can be used to measure the effective molarities for all possible intramolecular base-pairing interactions from 1,2-folding up to 1,10-folding.

**Figure 3:**
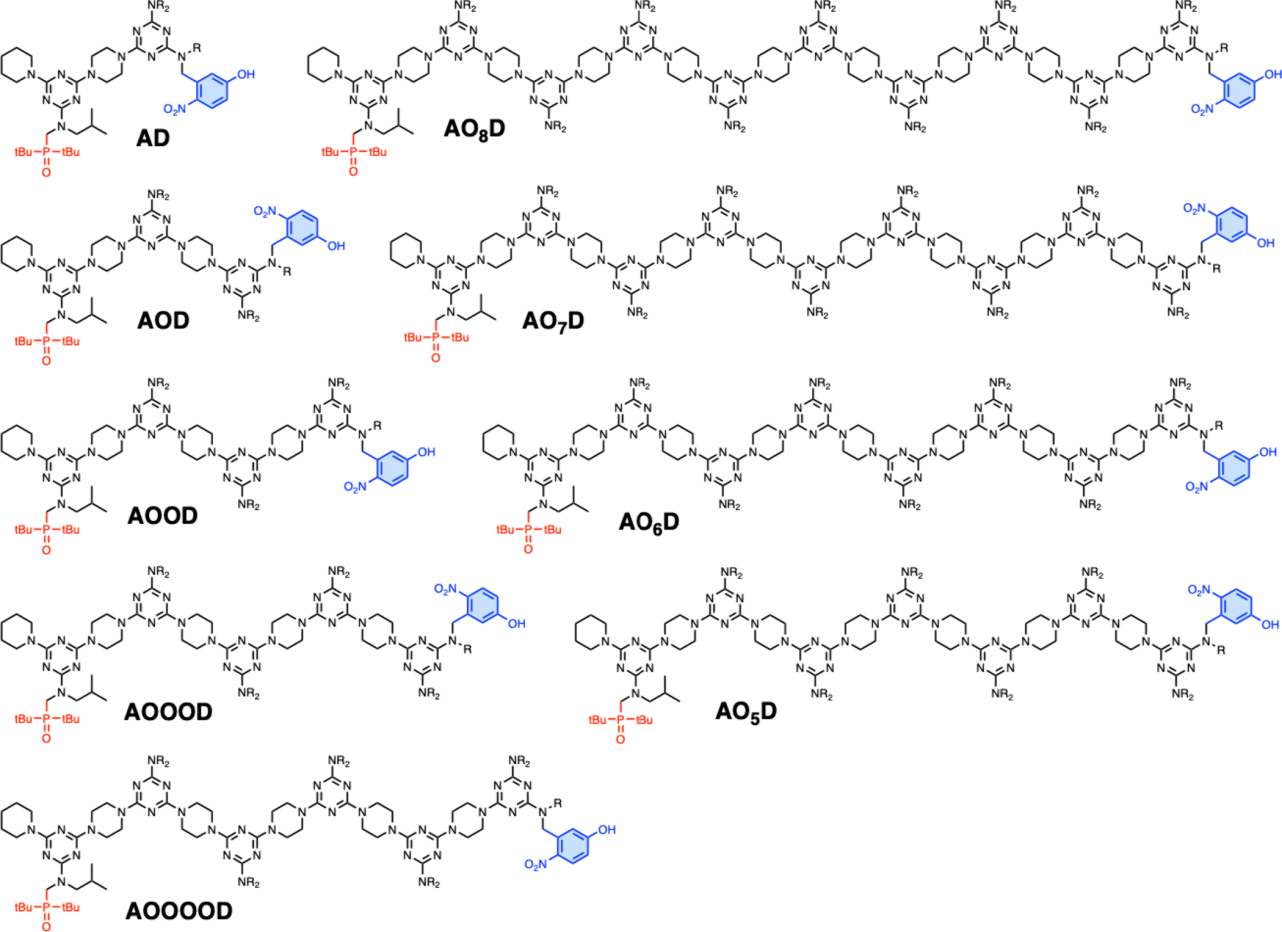
REMO equipped with complementary terminal recognition units, phosphine oxide (**A**) and 4-nitrophenol (**D**), separated by n blanks (**O**). R = 2-ethylhexyl.

### Synthesis

The longer **AO**_**n**_ **D** oligomers (n = 1–8) were synthesized using the automated solid phase synthesis protocol described previously (Scheme 1).^21^ The blank dichlorotriazine building block (**1**) required for oligomer synthesis was prepared in one step from cyanuric chloride and di-2-ethylhexylamine. The phosphine oxide dichlorotriazine (**2**) has been described previously.^18^ Scheme 2 shows the route used for functionalization of Wang resin for use in synthesis of REMO equipped with a terminal 4-nitrophenol recognition unit. The synthesis of amine **3** was described previously,^22^ and this compound was Fmoc protected in good yield to give **4**. Deprotection of the 4-methoxybenzyl protecting group with TFA gave **5**, which was coupled with Wang resin under Mitsunobu conditions. Treatment of a small amount of the Fmoc-protected resin with DBU allowed the loading to be determined (0.11 mmol g^–1^).^21^ This resin was used in a Liberty Blue peptide synthesizer to obtain the **AO**_**n**_ **D** oligomers in excellent yield and purity (see SI for details).

**Scheme 1:**
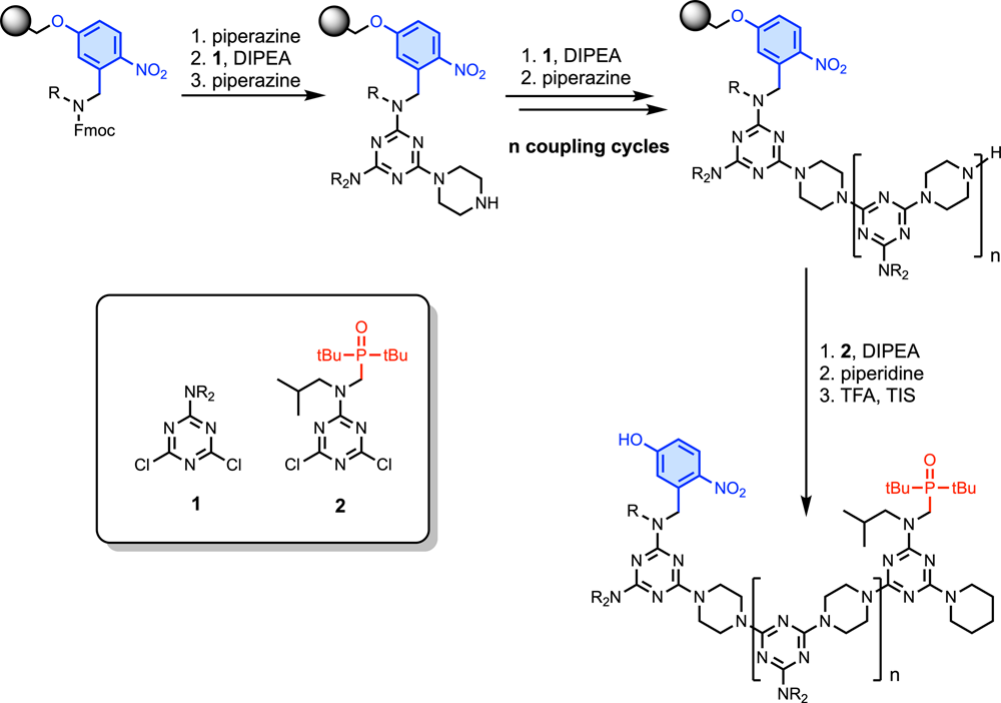
Automated Solid Phase Synthesis of **AO**_**n**_ **D** (n = 2–8)^a^

a R = 2-ethylhexyl.

**Scheme 2:**
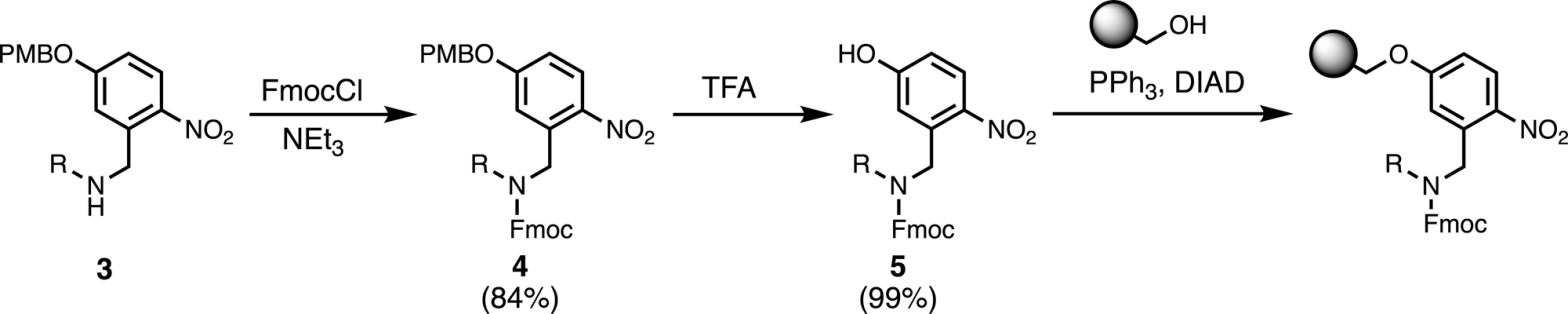
Functionalization of Wang Resin for Use in REMO Synthesis^a^

a R = 2-ethylhexyl, PMB = *p*-methoxybenzyl.

The shorter oligomer **AD** was synthesized by sequential S_N_Ar reactions of the corresponding dichlorotriazines with bis-2-ethylhexylamine, piperazine, and piperidine (see SI for details). In addition, a number of shorter oligomers were synthesized to characterize the duplex forming properties of REMO that cannot fold (Figure 4, see SI for synthetic details).

**Figure 4:**
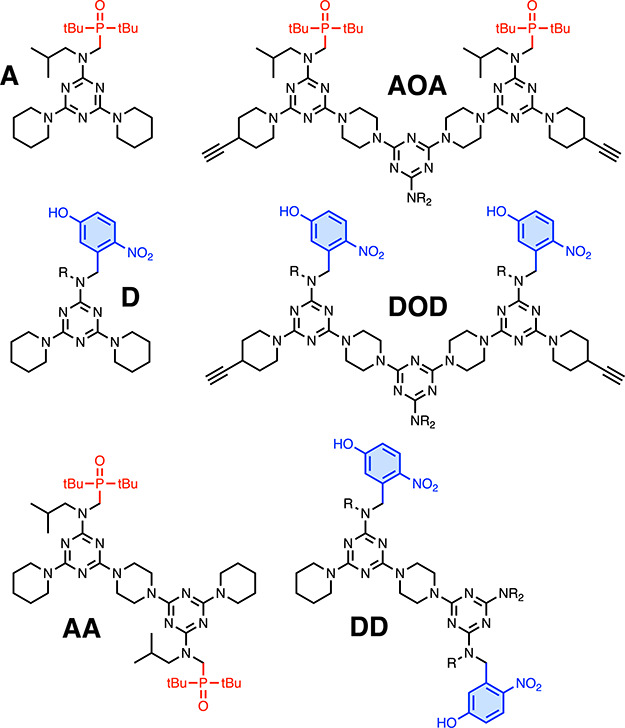
REMO used to study interactions between oligomers that cannot fold. R = 2-ethylhexyl.

### REMO Self-Assembly Pathways

Figure 5 compares the ^31^P NMR spectrum of the **A** 1-mer and the UV–vis absorption spectrum of the **D** 1-mer with corresponding spectra of the **AO**_**n**_ **D** oligomers. The ^31^P NMR signals in the oligomer spectra are broadened, and in some cases, splitting is apparent, which is due to population of different rotamers around the exocyclic nitrogen triazine bond that are in slow exchange on the chemical shift time scale. However, in all cases, the chemical shift of the ^31^P signal due to the **AO**_**n**_ **D** phosphine oxide is significantly larger than the value observed for corresponding signal in the reference compound **A**, which is indicative of H-bonding interactions in the oligomer solutions (Figure 5a). The UV–vis spectra of the **AO**_**n**_ **D** oligomers all have absorption maxima, λ_max_, between 315 and 326 nm, which is significantly higher than the value observed for corresponding signal in the reference compound **D** (303 nm) and indicative of H-bonding interactions in the oligomer solutions (Figure 5b). These experiments show that both the phosphine oxide and 4-nitrophenol recognition units form H-bonds in dichloromethane solution, which is good evidence for the formation of 4-nitrophenol·phosphine oxide base-pairs. However, these experiments do not distinguish between intramolecular interactions and intermolecular interactions. Moreover, the NMR experiments were carried out at millimolar concentrations, which favors intermolecular interactions, and the UV–vis experiments were carried out at micromolar concentrations, which favors intramolecular interactions.

**Figure 5:**
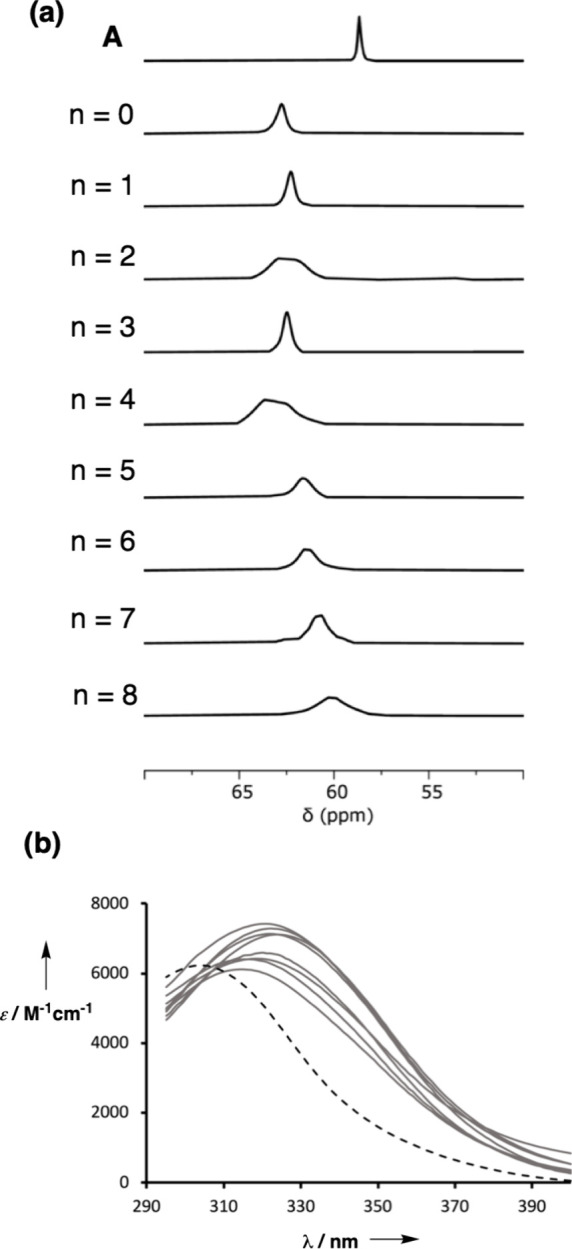
a) 162 MHz ^31^P NMR spectra of approximately 3 mM solutions of **A** and **AO**_**n**_ **D** (*n* = 0–8) in deuterodichloromethane at 298 K. b) UV–vis absorption spectra of 100 μM solutions of **D** (dotted line) and **AO**_**n**_ **D** (*n* = 0–8, continuous lines) in dichloromethane at 298 K.

Figure 6 shows H-bonding equilibria that are possible for a REMO equipped with two complementary recognition units (**AOOD** is illustrated, but the species are the same for the other **AO**_**n**_ **D** oligomers). Intramolecular 4-nitrophenol·phosphine oxide base-pairing interactions lead to folding, and intermolecular base-pairing interactions lead to duplex or polymer formation. The polymerization channel involves multiple species of different chain length, but we will refer to the total population of oligomers present as chains with *N* ≥ 1 as the polymer. The three self-assembled states highlighted in Figure 6 have different stoichiometries, so the population distribution can be varied by changing the total concentration of oligomer. At high concentrations, the polymer will dominate, and at low concentrations, only the single strand states will remain. It is possible to determine the concentration regimes in which the duplex and polymer are likely to compete with folding by using oligomers that cannot fold (see Figure 4) to directly measure values for the association constant for formation of an intermolecular 4-nitrophenol·phosphine oxide base-pair, *K*_A·D_, and the effective molarity for formation of the intramolecular base-pair leading to the duplex, EM_d_.

**Figure 6:**
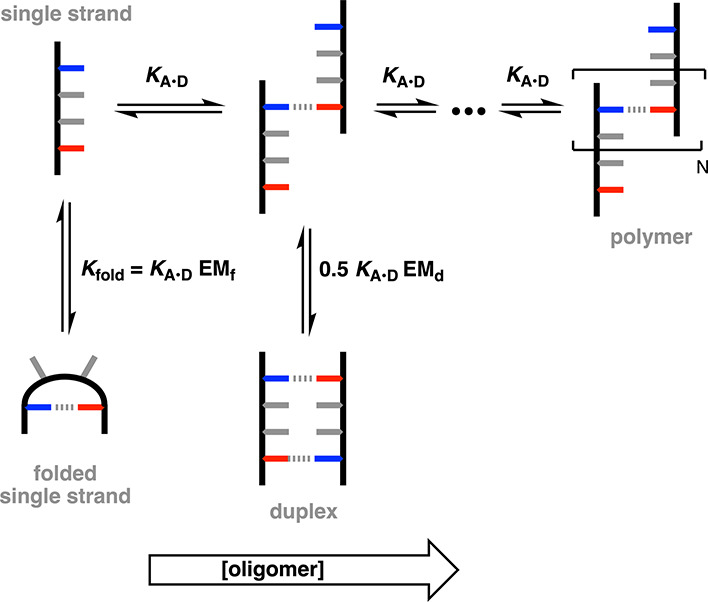
H-bonding equilibria for **AO**_**n**_ **D** REMO equipped with two complementary recognition units (red and blue bars). **AOOD** is illustrated with blank recognition sites shown as gray bars. At high concentrations of oligomer, the polymer dominates, at intermediate concentrations, the duplex may be populated, and at lower concentrations, the two single strand states dominate. *K*_A·D_ is the association constant for formation of an intermolecular 4-nitrophenol·phosphine oxide base-pair, and the populations of folded single strand and duplex are governed by the effective molarities for formation of intramolecular base-pairs, EM_f_ and EM_d_.

### Stability of the Polymer

The value of *K*_A·D_ was determined using a UV–vis absorption titration of the 1-mer **A** into the 1-mer **D** in dichloromethane. The titration data fit well to a 1:1 isotherm, giving value of *K*_A·D_ of 1,900 ± 300 M^–1^ (see SI for details). For an isodesmic polymerization, the speciation of polymer is directly determined by this parameter,^23,24^ and we conclude that the population of polymer will be less than 10% at oligomer concentrations lower than 50 μM (= 0.1/*K*_A·D_).

### Stability of the Duplex

Although EM_d_ will depend on the number of blanks separating the two recognition units, it is possible to obtain limits on the value of EM_d_ using the reference compounds in Figure 4. The value of EM_d_ for the duplex formed by **AD** should be the same as the value for formation of the **AA**·**DD** duplex, and the value of EM_d_ for the duplex formed by **AOD** should be the same as the value for formation of the **AOA**·**DOD** duplex. Since none of **AA**, **DD**, **AOA** and **DOD** can fold, the association constant for duplex formation can be measured directly by UV–vis titrations of the phosphine oxide oligomer into the complementary 4-nitrophenol oligomer. Figure 7 shows an example of the titration data for the **AA**·**DD** duplex. **DD** has a UV–vis absorption maximum at 303 nm in dichloromethane solution, and when **AA** was added, a new band appeared at 326 nm. Figure 7b shows that the titration data fit well to a 1:1 binding isotherm, and the root of mean square error (RMSE) between the experimental data and calculated spectra plotted in Figure 7d shows that the value of the association constant can be accurately determined under these conditions. The association constant is 64,000 ± 1,000 M^–1^, which is more than an order of magnitude larger than the value of *K*_A·D_. This result indicates that there is cooperative formation of two H-bonds in the **AA**·**DD** complex and gives a value of EM_d_ of 9 ± 1 mM (*K*_AA·DD_ = 2 EM_d_ *K* ^2^_A·D_).^18^ The association constant for formation of the **AOA**·**DOD** duplex is 12,000 ± 1,000 M^–1^, which corresponds to EM_d_ = 1.7 ± 0.2 mM (see SI for details). Presumably, the increase in chain length and the number of rotatable bonds separating the recognition units lowers the value of EM_d_ in **AOA**·**DOD** compared with **AA**·**DD**, and this result suggests that the values of EM_d_ will be even lower for longer oligomers where the recognition units are separated by more blanks.

**Figure 7:**
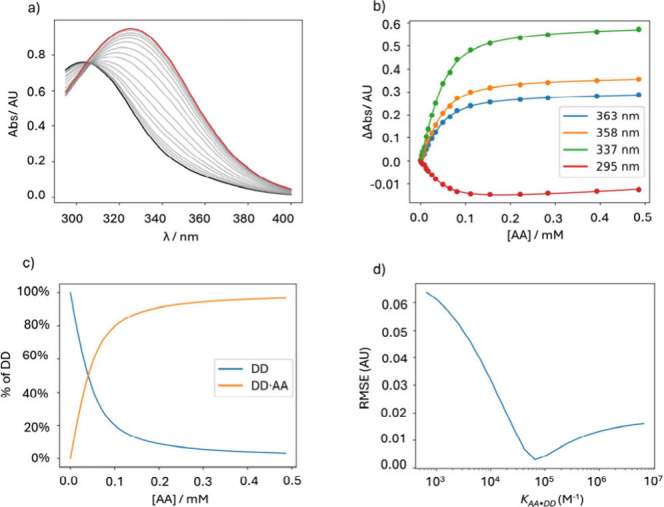
UV–vis absorption titration of **AA** into **DD** (50 μM) in dichloromethane at 298 K. a) UV–vis absorption spectra showing free **DD** in black and final spectrum in red. b) Best fit of the change in UV–vis absorbance at selected wavelengths to a 1:1 binding isotherm allowing for guest absorption. c) Calculated populations of different species containing **DD**. d) Relationship between the RMSE between the experimental data and calculated spectra plotted as a function of the value of *K*_AA·DD_.^25^

The experimental values of EM_d_ measured for the homo-oligomers can be used to estimate association constants for the duplexes formed by the self-complementary hetero-oligomers. There is a difference in symmetry, which leads to a 4-fold reduction in the stability of a **AO**_**n**_ **D**·**AO**_**n**_ **D** duplex compared with the corresponding **AO**_**n**_ **A**·**DO**_**n**_ **D** duplex,^18^ so we predict association constants of 16,000 M^–1^ and 3,000 M^–1^ for the **AD**·**AD** and **AOD**·**AOD** duplexes respectively, and lower values for the longer **AO**_**n**_ **D** oligomers.^26^ We can therefore conclude that the population of the duplex will be less than 10% at concentrations lower than 10 μM (with the exception of **AD**).

### Denaturation Experiments on **AO_n_D** Oligomers

Population of the folded single strand state of the **AO**_**n**_ **D** oligomers shown in Figure 6 was investigated using perfluoro-*tert*-butanol (PFTB) denaturation experiments. PFTB is a very strong H-bond donor, which can be used to break 4-nitrophenol·phosphine oxide base-pairing interactions by H-bonding to the phosphine oxide recognition unit, and denaturation can be detected by monitoring the change in the UV–vis absorption spectrum of the 4-nitrophenol recognition unit that is released.^27^ Moreover, PFTB is a very weak H-bond acceptor, so there is no interaction with the 4-nitrophenol recognition unit, even at very high PFTB concentrations.^27^ The association constant for the interaction of PFTB with a non-H-bonded phosphine oxide recognition unit, *K*_*d*_, was determined using a ^31^P NMR titration of PFTB into the 1-mer **A** in deuterodichloromethane (*K*_*d*_ = 3,100 ± 300 M^–1^, see SI for details). If a **AO**_**n**_ **D** oligomer populates any of the self-assembled states shown in Figure 6, then the apparent association constant for the interaction of this oligomer with PFTB, *K*_obs_, will be lower than the value of *K*_*d*_. In other words, denaturation with PFTB provides a method for quantifying the extent of folding, duplex and polymer formation. UV–vis absorption PFTB denaturation experiments were therefore carried out for each of the **AO**_**n**_ **D** oligomers at two different oligomer concentrations (10 μM and 100 μM) in dichloromethane solution.

Figure 8 shows the titration data for addition of PFTB to a 100 μM solution of **AD** in dichloromethane. On addition of PFTB, the intensity of the absorption maximum at 319 nm decreased, and a new band appeared at 303 nm, which is characteristic of a 4-nitrophenol that is not involved in any H-bonding interactions, i.e. the denatured single strand. The titration data could be fit to a simple 2-state isotherm that included only the free and bound oligomer, and the apparent association constant for interaction with PFTB, *K*_obs_, is 1,700 ± 100 M^–1^. This value is significantly lower than *K*_*d*_, which indicates that 4-nitrophenol·phosphine oxide base-pairing interactions compete with PFTB binding. When the titration was repeated at an oligomer concentration of 10 μM, the value of *K*_obs_ obtained by fitting the data to a 1:1 binding isotherm was significantly higher than in the 100 μM experiment (*K*_obs_ = 2,800 ± 500 M^–1^, see SI for details). Since the polymer is not significantly populated at these concentrations and the population of the folded single strand is concentration-independent, the concentration-dependent value of *K*_obs_ observed for **AD** indicates that this oligomer forms a duplex, which is more populated in the 100 μM experiment than in the 10 μM experiment. This result is consistent with the association constant estimated above for formation of the **AD**·**AD** duplex (16,000 M^–1^).

**Figure 8:**
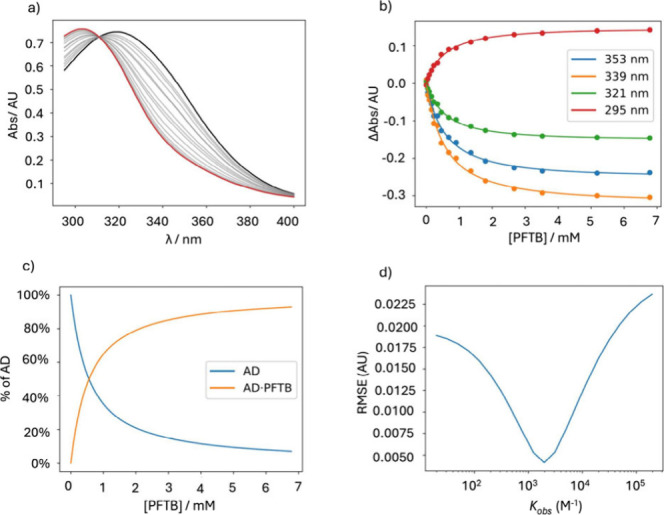
UV–vis absorption denaturation of **AD** (100 μM) with PFTB in dichloromethane at 298 K. a) UV–vis absorption spectra showing the starting spectrum in black and the final spectrum in red. b) Best fit of the change in UV–vis absorbance at selected wavelengths to a 2-state isotherm allowing for guest absorption. c) Calculated populations of different species containing **AD**, d) Relationship between the RMSE between the experimental data and calculated spectra plotted as a function of the value of *K*_obs_.^25^

The PFTB denaturation data for all of the **AO**_**n**_ **D** oligomers also fit well to 1:1 binding isotherms, and the resulting values of the apparent association constant, *K*_obs_, are listed in Table 1. In all cases, the 100 μM experiment gave a value of *K*_obs_ that is significantly lower than the value of *K*_d_ (3,100 M^–1^), confirming significant population of at least one of the self-assembled species shown in Figure 6. For **AD** and **AOD**, the value of *K*_obs_ is concentration-dependent, but for all of the other oligomers the value of *K*_obs_ measured in the 10 μM experiment is within error of the value measured in the 100 μM experiment. Moreover, the wavelength of the absorption maximum, λ_max_, in the absence of PFTB is concentration-dependent for **AD** and **AOD** but not for the other oligomers (Table 1). These results indicate that the duplex is significantly populated for **AD** and **AOD**, but the H-bonding interactions observed for the longer oligomers are exclusively due to intramolecular base-pairing in the folded single strand.

**Table 1:** PFTB Denaturation Experiments: UV–Vis Absorption Maxima, Apparent Association Constants for Interaction with PFTB, and Parameters That Govern Single Strand Folding Measured in Dichloromethane at 298 K^a^

**[oligomer]**	10 μM	100 μM	10 μM	100 μM			**% folded**
**oligomer**	**λ**_**max**_ **(nm)**	**λ**_**max**_ **(nm)**	***K***_**obs**_ **(M** ^**–1**^ **)**	***K***_**obs**_ **(M** ^**–1**^ **)**	**EM**_**f**_ **(mM)**	***K***_**fold**_	**1 H-bond**
**AD**	311	319	2800 ± 300	1700 ± 130	-	-	-
**AOD**	313	315	3000 ± 160	2500 ± 170	0.7 ± 0.1	1.3 ± 0.2	57%^b^
**AO**_**2**_ **D**	322	322	850 ± 80	760 ± 20	1.6 ± 0.1	3.0 ± 0.2	75%
**AO**_**3**_ **D**	325	325	320 ± 60	320 ± 10	4.5 ± 0.1	8.5 ± 0.2	90%
**AO**_**4**_ **D**	326	326	300 ± 10	290 ± 20	5.1 ± 0.3	9.7 ± 0.6	91%
**AO**_**5**_ **D**	320	320	840 ± 40	780 ± 90	1.9 ± 0.3	3.6 ± 0.6	78%
**AO**_**6**_ **D**	318	318	760 ± 20	800 ± 70	1.3 ± 0.2	2.5 ± 0.4	71%
**AO**_**7**_ **D**	314	314	1700 ± 200	1400 ± 100	0.6 ± 0.1	1.1 ± 0.2	53%
**AO**_**8**_ **D**	320	320	1400 ± 200	1100 ± 100	0.8 ± 0.1	1.5 ± 0.2	60%

a All values are the average of three independent experiments, and errors are quoted as twice the standard error of the mean.

b This value is the population calculated at a concentration of 1 μM, where the duplex does not compete.

Having established which of the self-assembled species shown in Figure 6 are present, the denaturation data can be quantitatively analyzed to determine the equilibrium constant and effective molarity for folding of the single strand, *K*_fold_ and EM_f_. For the oligomers that do not populate the duplex (i.e., **AO**_**2**_ **D** to **AO**_**8**_ **D**), the denaturation data were fit to a 5-state isotherm that included the single strand, the folded single strand, the polymer, the single strand bound to PFTB, and the polymeric species bound to PFTB on the chain end. The association constant for the interaction of PFTB with a non-H-bonded phosphine oxide recognition unit, *K*_*d*_, and the value of *K*_A·D_, which governs the polymerization process, are both known. Therefore, assuming that the UV–vis absorption spectrum of a H-bonded and a non-H-bonded 4-nitrophenol can be treated as constants, the denaturation data can be fit by optimizing only one additional parameter, EM_f_.

Figure 9 shows the result for **AOOD**. The denaturation data fit well to the 5-state isotherm (Figure 9b), and the RMSE plot in Figure 9d shows that the value of EM_f_ can be accurately determined under these conditions. The speciation plot in Figure 9c shows that the polymer is not populated to any significant extent, as expected (red line). In the absence of denaturant, the folded single strand is the major species (75%, green line in Figure 9c).

**Figure 9:**
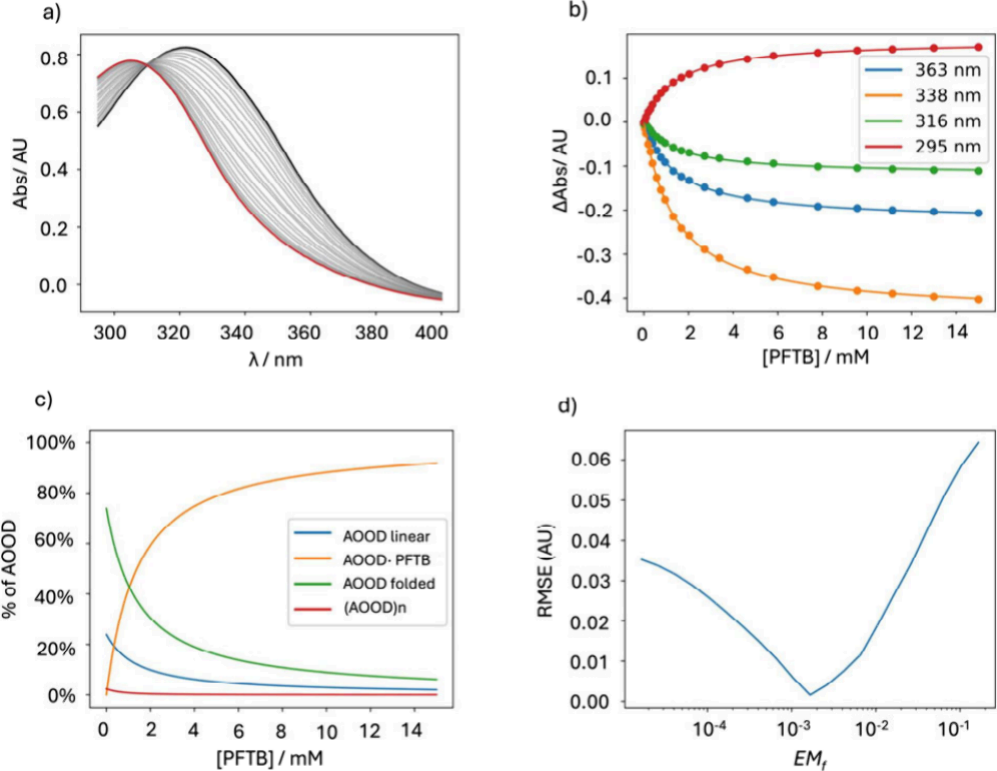
UV–vis absorption denaturation of **AOOD** (100 μM) with PFTB in dichloromethane at 298 K. a) UV–vis absorption spectra showing the starting spectrum in black and the final spectrum in red. b) Best fit of the change in UV–vis absorbance at selected wavelengths to a 5-state isotherm (the single strand, the folded single strand, the polymer, the single strand bound to PFTB, and the polymeric species bound to PFTB on the chain end) allowing for guest absorption. c) Calculated populations of different species containing **AOOD**, d) Relationship between the RMSE between the experimental data and calculated spectra plotted as a function of the value of EM_f_.^25^

Similar results were obtained for the longer oligomers, **AO**_**3**_ **D** to **AO**_**8**_ **D** (see SI for details). The values of EM_f_, *K*_fold_ and the populations of the folded single strand are summarized in Table 1. The 1,5 and 1,6-folds give the most stable intramolecular base-pairing interactions, and the folded single strand is 90% populated for **AO**_**3**_ **D** and **AO**_**4**_ **D**. The equilibrium constants for folding (*K*_fold_ = *K*_A·D_ EM_f_) correlate well with the values of *K*_obs_ (eq 1, R^2^ = 0.99), and the populations of the folded single-strand correlate with the wavelengths of the UV–vis absorption maximum: **AO**_**3**_ **D** and **AO**_**4**_ **D** have a λ_max_ of 325 and 326 nm, which are similar to the value for the fully bound **AA**·**DD** duplex in Figure 7 (326 nm).

1 $$K_{\mathrm{obs}}=\frac{K_{d}}{1+K_{\mathrm{fold}}}$$

Analysis of the denaturation data for **AD** and **AOD** is more complicated, because in both cases the duplex is populated, so two different effective molarities, EM_f_ and EM_d_, are required to fit the data. However, we have already measured EM_d_ for **AA**·**DD** and **AOA**·**DOD**, and assuming that these values are transferrable to the isomeric duplexes **AD**·**AD** and **AOD**·**AOD**, the denaturation data can be fit by optimizing only one parameter, EM_f_, in the same way as for the other oligomers. The only difference is that the duplex is included as an extra species in a 6-state isotherm. Figure 10 shows the result for **AOD**. The denaturation data fit well to the 6-state isotherm (Figure 10b), and the RMSE plot in Figure 10d shows that the value of EM_f_ can be accurately determined under these conditions. The speciation in Figure 10c shows that small amounts of duplex and polymeric species are present (≈ 10% each, red and purple lines), and in the absence of denaturant, the single strand and folded single strand are equally populated (≈ 40% each, green and blue lines). For **AD**, the folded single strand was not populated at all, and the denaturation data fit equally well to a model that does not include this species (see SI for details). These results show that 1,2-folding does not occur in this system.

**Figure 10:**
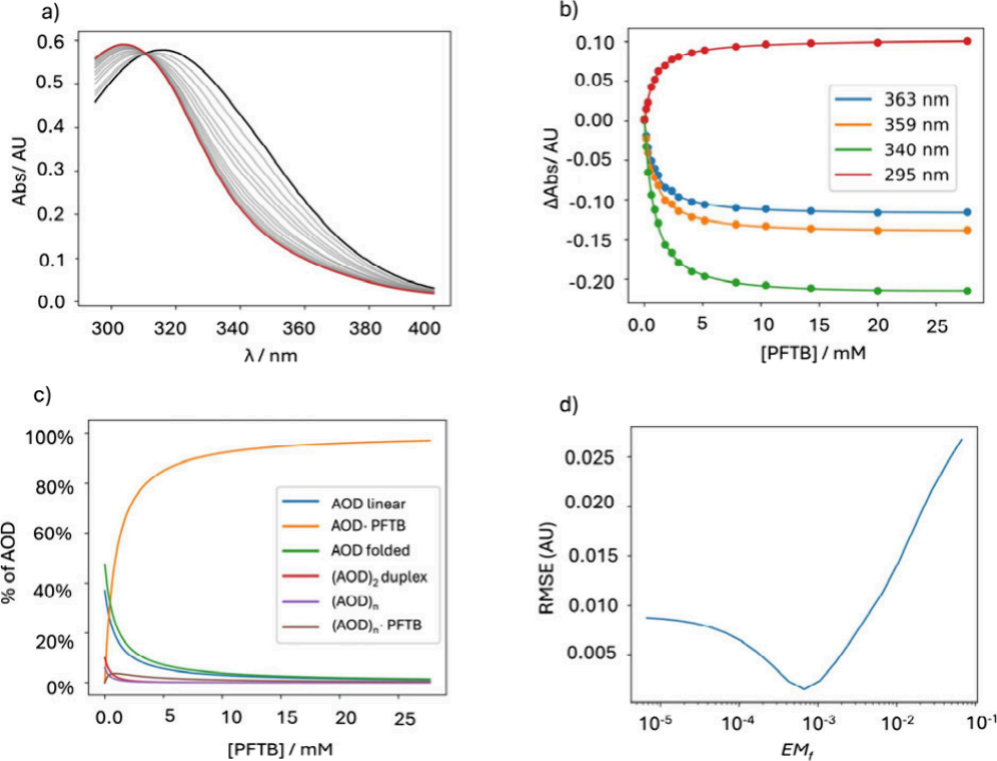
UV–vis absorption denaturation of **AOD** (100 μM) with PFTB in dichloromethane at 298 K. a) UV–vis absorption spectra showing the starting spectrum in black and the final spectrum in red. b) Best fit of the change in UV–vis absorbance at selected wavelengths to a 6-state isotherm (the single strand, the folded single strand, the duplex, the polymer, the single strand bound to PFTB, and the polymeric species bound to PFTB on the chain end) allowing for guest absorption. c) Calculated populations of different species containing **AOD**, d) Relationship between the RMSE between the experimental data and calculated spectra plotted as a function of the value of EM_f_.^25^

The relative robustness of the folded structures can be evaluated for the **AO**_**n**_ **D** oligomers by comparing the % folded values listed in Table 1. The 1,5 and 1,6-folds are 90% populated, whereas the other oligomers are between 50% and 80% folded. Figure 11 summarizes the folding behavior, showing the relationship between EM_f_ and the separation of the two recognition units. The backbone is sufficiently rigid to prevent 1,2-folding, but all of the other oligomers fold to some extent. For most sequences, the value of EM_f_ is around 1 mM, but 1,5 and 1,6-folding is significantly more favorable with EM_f_ values of 5 mM. Figure 12 provides a rationalization of these results. Figure 12a shows the lowest energy structure of one repeat of the REMO backbone calculated using density functional theory (B3LYP/6-31G**), and this conformation is identical to that found in an X-ray crystal structure of a REMO 2-mer (see SI for details). The conformation of the piperazine unit is a chair, and the piperazine nitrogen atoms are trigonal planar to allow conjugation with the triazine ring. This structure suggests that the backbone is roughly flat with a 1.2 Å step out of the plane at each piperazine unit. The most important degree of freedom accessible to the REMO backbone is therefore a flip between the two coplanar conformations around each triazine-piperazine bond, so that the triazine units of the backbone fall on a hexagonal grid, as illustrated in Figure 12. The conformations in Figures 12b and 12c allow recognition units at positions 1 and 5 or positions 1 and 6 to come into close proximity, which accounts for the high values of EM_f_ observed for these sequences. For shorter oligomers, a planar backbone would hold the terminal recognition units further apart, so some unfavorable distortion is required to allow intramolecular base-pairing. For longer oligomers, the lower values of EM_f_ may be due to conformational strain or restriction of conformational flexibility.

**Figure 11:**
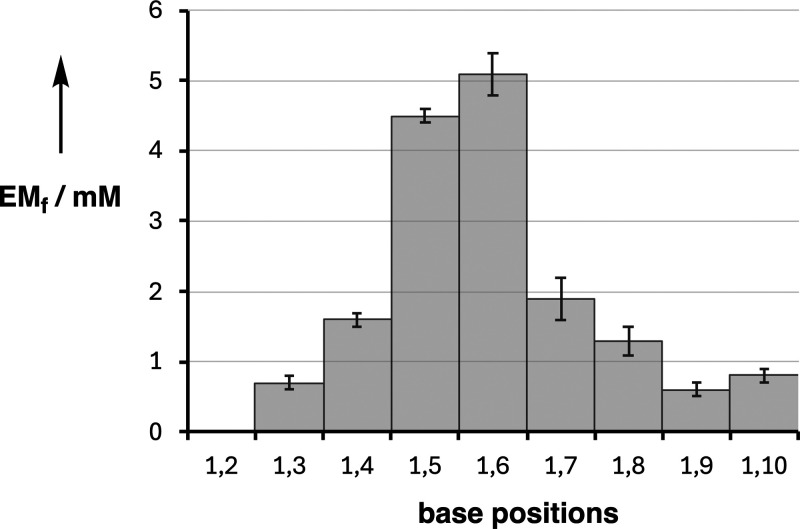
Relationship between EM_f_ and the location on the REMO backbone of two complementary recognition units that can form a base-pair.

**Figure 12:**
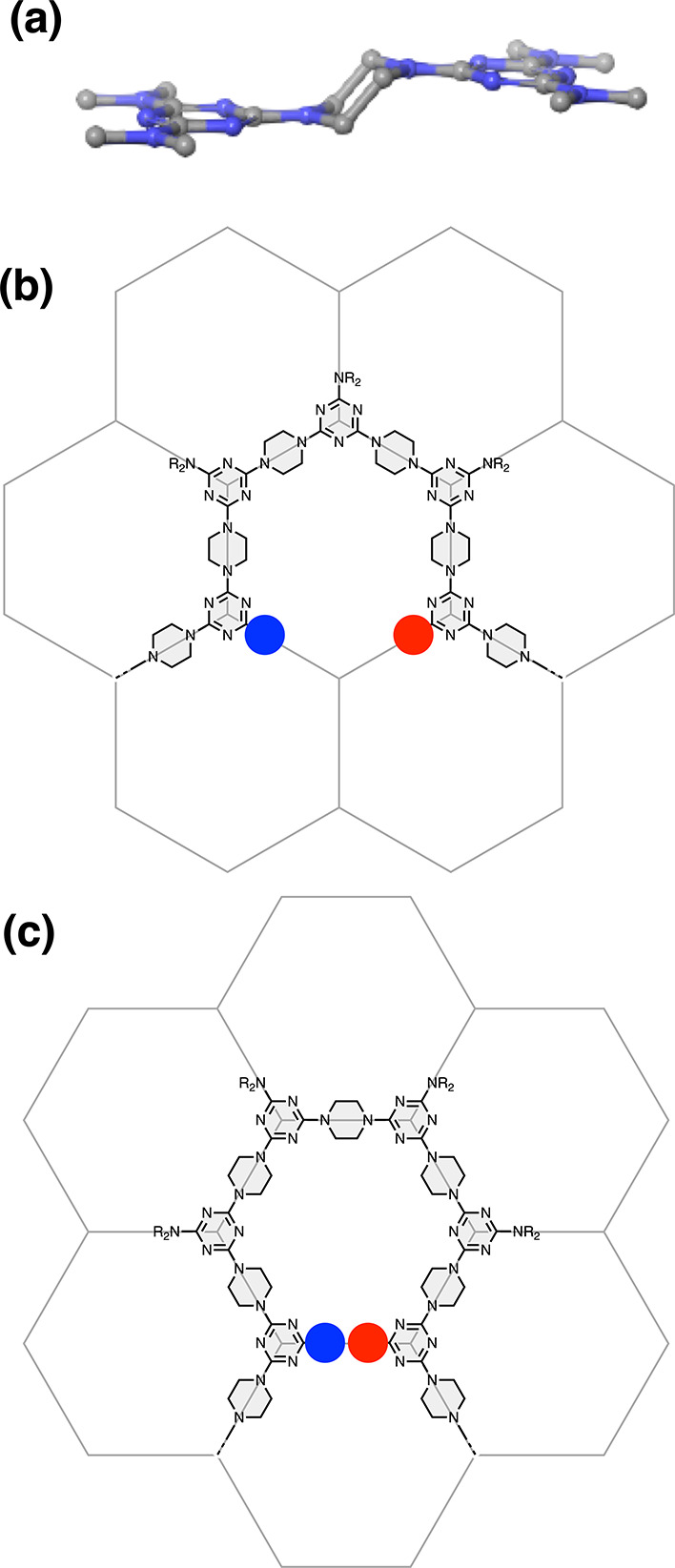
a) Lowest energy structure of the REMO backbone calculated with density functional theory (B3LYP/6-31G**). b) REMO folding on a hexagonal grid puts complementary recognition units (red and blue) at positions 1 and 5 in close proximity, or c) positions 1 and 6 in close proximity.

We have previously reported that 1,3-folding did not occur in chloroform for REMO equipped with phenol and phosphine oxide recognition units.^18^ The reason for this apparent discrepancy can be understood based on differences in the values of *K*_A·D_: 1,900 M^–1^ for 4-nitrophenol·phosphine oxide in dichloromethane, and 120 M^–1^ for phenol·phosphine oxide in chloroform.^19^ Assuming that the value of EM_f_ measured for 1,3-folding (0.7 mM) can be used for the two different systems, the values of *K*_fold_ are 0.08 for a phenol-phosphine oxide REMO in chloroform and 1.3 for a 4-nitrophenol-phosphine oxide REMO in dichloromethane. In other words, 1,3-folding is difficult to detect for a phenol-phosphine oxide REMO in chloroform (<10% populated), because the extent of folding depends as much on the strength of the base-pairing interaction as on the conformational properties of backbone.

### Folding of Oligomers with Four Recognition Units

Knowledge of the effective molarities for formation of intramolecular base-pairs provides a basis for tackling more complicated sequences. Figure 13 shows the structures of five different REMO sequences that can all form two intramolecular base-pairs. These oligomers were synthesized using automated solid phase synthesis (see SI for details). For four out of five oligomers, the wavelength of the UV–vis absorption maximum, λ_max_, is close to the value for the fully bound **AA**·**DD** duplex in Figure 7 (326 nm), which suggests that both of the 4-nitrophenol moieties are fully H-bonded (Table 2). The exception is **DAO**_**3**_ **AD**, which appears to be less folded than the other four sequences. The folding equilibria were investigated using UV–vis PFTB denaturation experiments at two different oligomer concentrations (5 μM and 50 μM) in dichloromethane solution. The PFTB denaturation data for all of these oligomers fit well to 1:1 binding isotherms, and the resulting values of the apparent association constant, *K*_obs_, are listed in Table 2. The values of *K*_obs_ measured in the 50 μM experiments are within error of the values measured in the 5 μM experiments, and the values of λ_max_ are also concentration-independent. There are two possible interpretations: neither the duplex nor the polymer is significantly populated at these concentrations, and folding dominates; or either the duplex or the polymer is fully bound over this concentration range with an association constant greater than 10^6^ M^–1^(i.e., 98% bound at 5 μM), and these species compete with folding. These two scenarios can be distinguished using a more detailed comparative analysis of the denaturation data obtained at 5 μM and at 50 μM.

**Figure 13:**
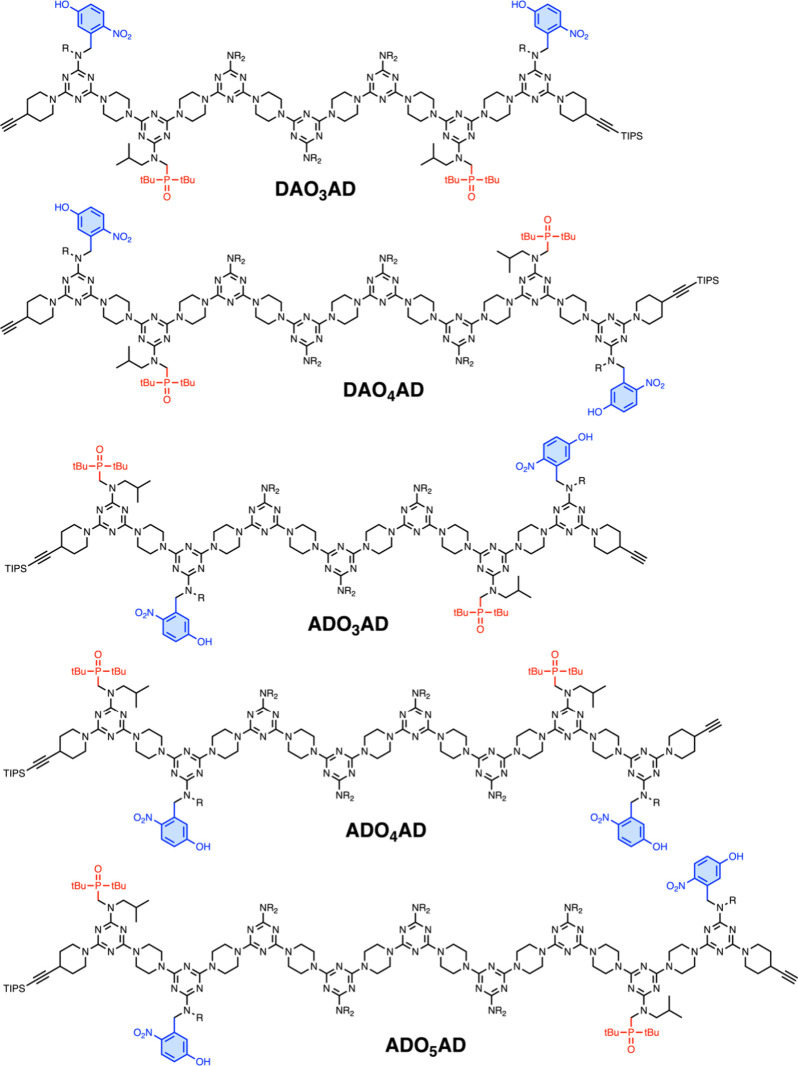
REMO equipped with two pairs of complementary terminal recognition units, phosphine oxide (**A**) and 4-nitrophenol (**D**), separated by 3 to 5 blanks (**O**). R = 2-ethylhexyl.

**Table 2:** PFTB Denaturation Experiments: UV–Vis Absorption Maxima, Apparent Association Constants for Interaction with PFTB, and Parameters That Govern Single Strand Folding Measured in Dichloromethane at 298 K^a^

**[oligomer]**	5 μM	50 μM	5 μM	50 μM			**% folded**	
**oligomer**	**λ**_**max**_ **(nm)**	**λ**_**max**_ **(nm)**	***K***_**obs**_ **(M** ^**–1**^ **)**	***K***_**obs**_ **(M** ^**–1**^ **)**	**α**	***K***_**fold**_	**1 H-bond**	**2 H-bonds**
**DAO**_**3**_ **AD**	323	323	560 ± 30	460 ± 20	0.2 ± 0.02	19 ± 2	49%	48%
**DAO**_**4**_ **AD**	325	325	240 ± 50	240 ± 50	5 ± 0.5	65 ± 7	10%	89%
**ADO**_**3**_ **AD**	325	325	560 ± 80	420 ± 60	2 ± 0.5	60 ± 20	16%	82%
**ADO**_**4**_ **AD**	326	326	140 ± 20	140 ± 20	14 ± 3	340 ± 70	3%	96%
**ADO**_**5**_ **AD**	325	325	240 ± 40	260 ± 430	30 ± 6	120 ± 20	4%	96%

a All values are the average of three independent experiments, and errors are quoted as twice the standard error of the mean.

For oligomers with four recognition units, a large number of different species can be formed. Figure 14 shows representative examples of different types of self-assembled species that are possible for **ADO**_**4**_ **AD**. In this case a variety of different folded, duplex and polymer structures are possible. For the polymerization channel, the experiments on **AD** show that formation of species with two intermolecular base-pairing interactions (*K*_AD·AD_ = 16,000 M^–1^) outcompetes the formation of any assemblies where two molecules are connected by a single base-pairing interaction (*K*_A·D_ = 1,900 M^–1^), so the latter were not considered. Formation of the fully H-bonded duplex is governed by a single unknown parameter EM_d2_, the effective molarity for formation the intramolecular **AD**·**AD** interaction illustrated in Figure 14.

**Figure 14:**
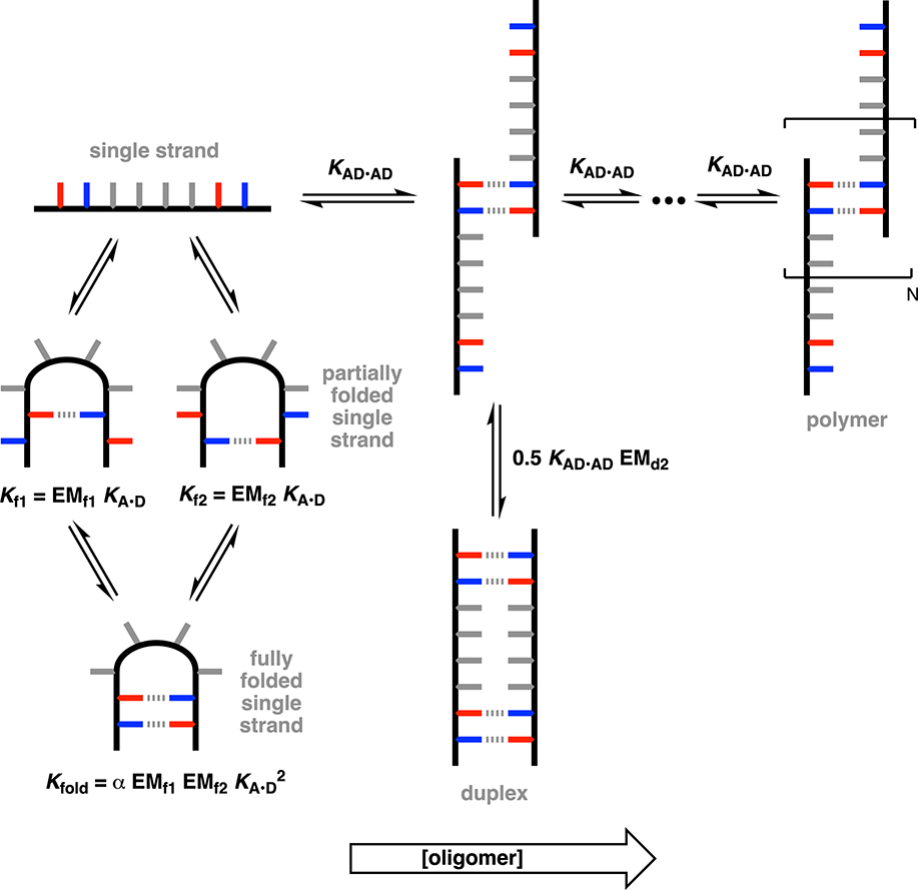
H-bonding equilibria for REMO equipped with two pairs of complementary recognition units (red and blue bars). **ADO**_**4**_ **AD** is illustrated with blank recognition sites shown as gray bars. Additional species that are partially assembled or involve single intermolecular 4-nitrophenol·phosphine oxide base-pair interactions are not significantly populated and are not shown. *K*_A·D_ is the association constant for formation of an intermolecular 4-nitrophenol·phosphine oxide base-pair. *K*_AD·AD_ is the association constant for formation of an **AD**·**AD** complex with two intermolecular 4-nitrophenol·phosphine oxide base-pairs. EM_d2_ is the effective molarity for formation of an intramolecular **AD**·**AD** interaction in the duplex. Three different folded states are possible, and the stability relative to the unfolded single strand (*K*_f1_, *K*_f2_ or *K*_fold_) depends on the effective molarities for the two intramolecular base-pairing interactions, EM_f1_ and EM_f2_, and the cooperativity between the two interactions in the fully folded single strand, α.

For the folding channel, the stabilities of the partially folded single strand states (*K*_f1_ and *K*_f2_), which have one intramolecular H-bonding interaction, are already known from the values of EM_f_ in Table 1. For the **ADO**_**4**_ **AD** example illustrated in Figure 14, EM_f1_ and EM_f2_ correspond to the values for 1,6 and 1,8-folding, respectively. The only new species is the fully folded single strand that makes two intramolecular 4-nitrophenol·phosphine oxide H-bonds. If the two H-bonds have no effect on one another, then the stability of this state will be given by the product of the equilibrium constants for the two singly H-bonded species, *K*_f1_ *K*_f2_. However, cooperativity between the two interactions in the doubly H-bonded state could lead to either an increase or a decrease in stability, and the parameter used to quantify this kind of allosteric cooperativity is α (*K*_fold_ = α *K*_f1_ *K*_f2_ = α EM_f1_ EM_f2_ *K* ^2^_A·D_).^28^ A value of α that is less than one indicates negative cooperativity, which could be caused by geometric incompatibility between the two H-bonds. A value of α that is greater than one indicates positive cooperativity, where the two H-bonds reinforce one another in the fully folded single strand.

The denaturation data for these systems were therefore fit to multistate isotherms that included a variety of single strand, duplex and polymeric species, bound to different number of PFTBs (see SI for details). Since the values of *K*_A·D_, *K*_AD·AD_ and the effective molarities for the partially folded states are known, the denaturation data can be fit by optimizing only one parameter, the cooperativity factor α for the folding channel, or the effective molarity EM_d2_ for the duplex channel. Attempts to fit the data with isotherms that allowed simultaneous optimization of both of these parameters failed to converge due to overfitting. Isotherms that did not include the fully folded single strand and optimized just EM_d2_ were able to fit the data, but the resulting values of EM_d2_ varied by an order of magnitude depending on the oligomer concentration (see SI for details). In contrast, isotherms that did not include the duplex and optimized just α not only fit the data well but also gave consistent values of α at different oligomer concentrations. These results indicate that the duplex is not populated to any significant extent and that the four single strand states dominate the self-assembly of these oligomers at micromolar concentrations.

Figure 15 shows the result for **ADO**_**4**_ **AD**. The denaturation data fit well to a 14-state isotherm (see SI for details of all species), and the RMSE plot in Figure 15d shows that the value of α can be accurately determined under these conditions. The speciation plot in Figure 15c shows that three major species are populated. In the absence of denaturant, the single strand is fully folded with two intramolecular H-bonds (96%). In the presence of small amounts of PFTB, one of the intramolecular base-pairing interactions is broken, and the partially folded single strand states bound to one PFTB are populated. In the presence of excess PFTB, the fully denatured single strand bound to two PFTBs dominates. The denaturation data for the other oligomers also fit well to multistate isotherms, and the values of α and populations of the partially folded and fully folded single-strands are summarized in Table 2 (see SI for details).

**Figure 15:**
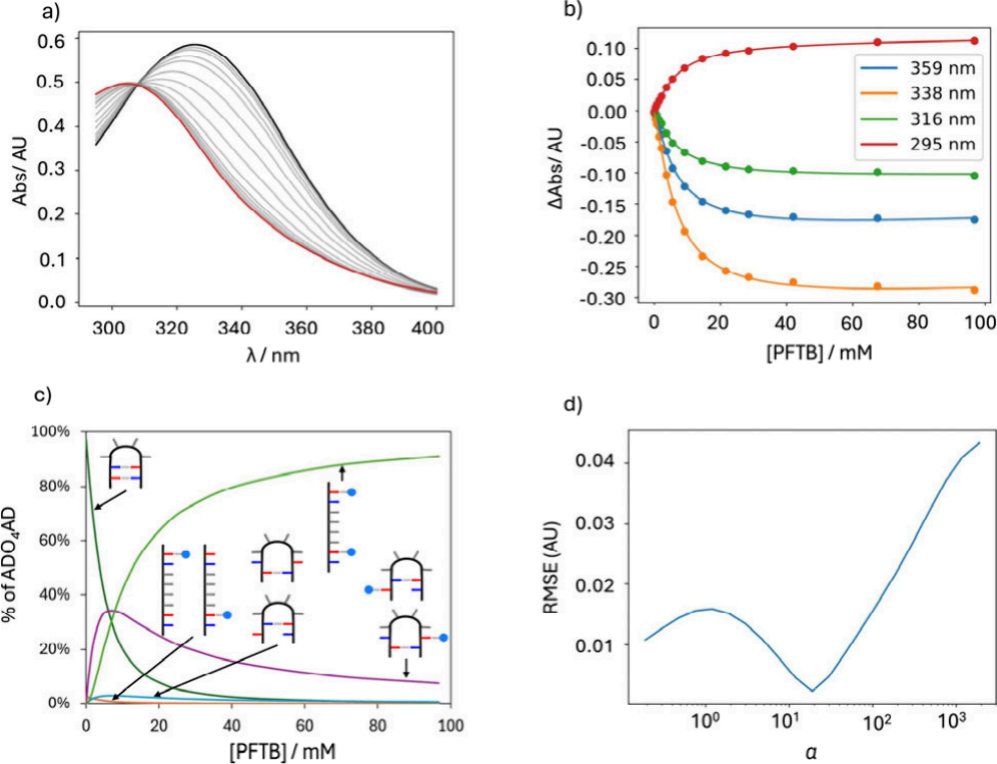
UV–vis absorption denaturation of **ADO**_**4**_ **AD** (50 μM) with PFTB in dichloromethane at 298 K. a) UV–vis absorption spectra showing the starting spectrum in black and the final spectrum in red. b) Best fit of the change in UV–vis absorbance at selected wavelengths to a 14-state isotherm allowing for guest absorption (see SI for details). c) Calculated populations of different species containing **ADO**_**4**_ **AD**: the single strand, the two partially folded single strands, the fully folded single strand, the single strand bound to one PFTB, the single strand bound to two PFTBs, and the two partially folded single strands bound to one PFTB (species that are not significantly populated are not shown). d) Relationship between the RMSE between the experimental data and calculated spectra plotted as a function of the value of α.^25^

The sequences of the recognition units in these oligomers dictate the topology of the folded state. The **ADO**_**n**_ **AD** sequences should encode hairpin loops (Figure 14), whereas the **DAO**_**n**_ **AD** sequences should encode helices (Figure 16). Two of the three **ADO**_**n**_ **AD** sequences in Table 2 show strong positive cooperativity (α > 10), and the fully folded single strand is almost fully populated, which means that hairpin loops are likely to be a highly favored folding motif for mixed sequence REMO. The third shorter sequence, **ADO**_**3**_ **AD**, shows very weak positive cooperativity, which suggests that a minimum of four looped-out bases is required to form a stable hairpin structure.

**Figure 16:**
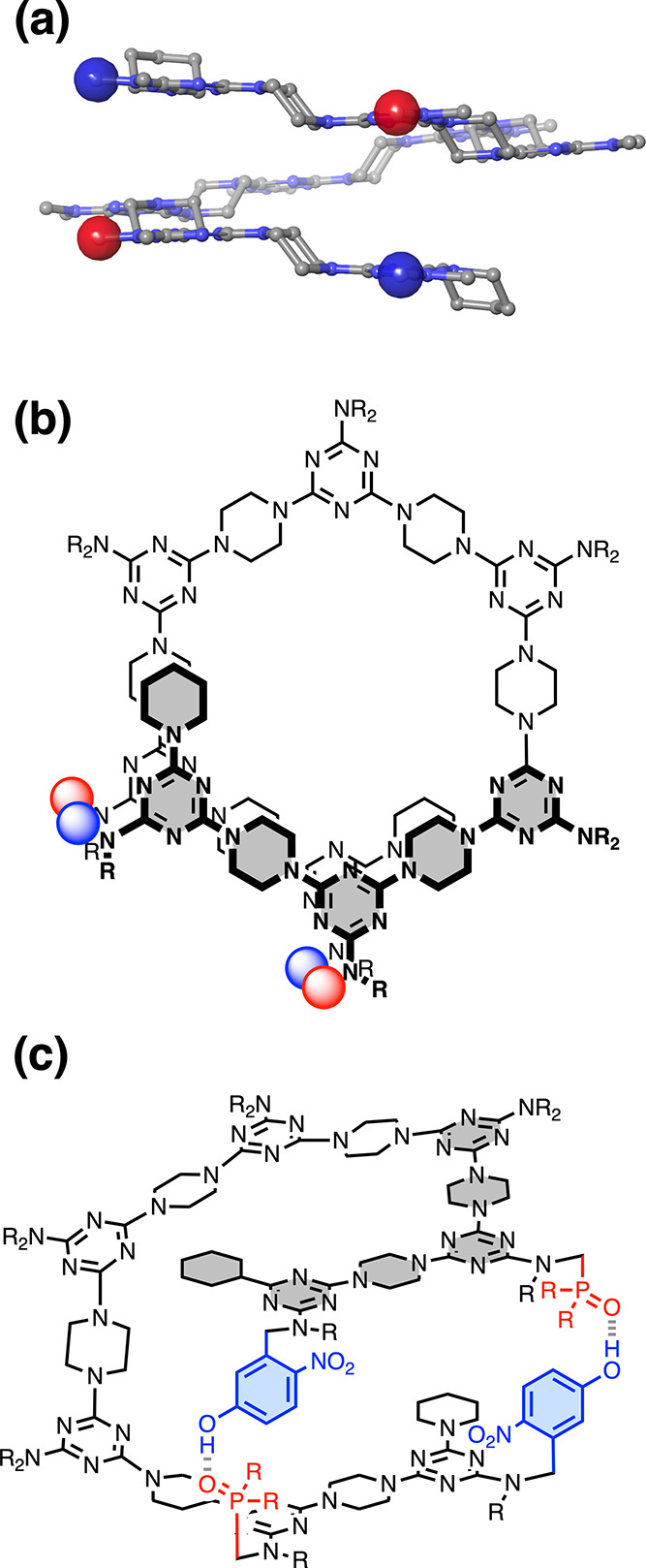
a) Molecular model of a REMO 8-mer in a helical conformation built from energy minimized backbone fragments (density functional theory B3LYP/6-31G**). All side chains were replaced by methyl groups, and the positions of the 4-nitrophenol and phosphine oxide recognition units in **DAO**_**4**_ **AD** are highlighted as blue and red spheres, respectively. b) Top view of the helical structure of the **DAO**_**4**_ **AD** fully folded single strand. c) Side view of the **DAO**_**4**_ **AD** helix.

One of the **DAO**_**n**_ **AD** sequences shows negative cooperativity (**DAO**_**3**_ **AD**) and the other shows positive cooperativity (**DAO**_**4**_ **AD**). Figure 16a shows a model of the REMO backbone in a helical conformation. The triazines lie on the hexagonal grid illustrated in Figure 12, but the piperazine chair conformations allow the backbone to progressively step upward out of the plane to generate a three-dimensional helix. The strain-free backbone conformation illustrated in Figure 16a places the side chains at positions 1 and 7 and positions 2 and 8 on top of one another. This conformation therefore promotes simultaneous formation of 1,7 and 2,8-base-pairing interactions, which accounts for the positive cooperativity observed when **DAO**_**4**_ **AD** folds. Top and side views of the base-pairing interactions in the **DAO**_**4**_ **AD** helix are illustrated in Figures 16b and 16c, respectively. Folding of **DAO**_**3**_ **AD** into a helical conformation with two intramolecular base-pairs requires interactions between the side chains at positions 1 and 6 and positions 2 and 7, which presumably involves a significant distortion of the backbone away from the strain-free conformation shown in Figure 16a, so in this case, the partially and fully folded single strands are equally populated.

The populations of the H-bonded states in Tables 1 and 2 allow an analysis of the relative robustness of the folded structures formed by different oligomers. For the **AO**_**n**_ **D** oligomers, the 1,5 and 1,6-folds are 90% populated, and other folded structures are less stable (50–80% folded). For the **DAO**_**n**_ **AD** and **ADO**_**n**_ **AD** oligomers, there is almost none of the unfolded state (<3%) in any case, and the two most stable folds are the **ADO**_**4**_ **AD** and **ADO**_**5**_ **AD** hairpin loops, where the doubly H-bonded states are 96% populated. The REMO hairpin loop structure has obvious analogies with the stem loop structures that are a characteristic feature of the folding of nucleic acids. For REMO, at least 4 looped out bases are required to form a stable hairpin, which is very similar to nucleic acids, where the most stable motif is the tetraloop with 4 unpaired bases.^29^

## Conclusions

Recognition-encoded melamine oligomers (REMO) equipped with phenol and phosphine oxide side chains form H-bonded duplexes in a sequence-selective manner due to intermolecular base-pairing interactions between complementary recognition units. It is also possible to make intramolecular phenol·phosphine oxide base-pairing interactions between two recognition units on the same oligomer, which leads to folding of single strands in a manner that resembles the folding of single stranded nucleic acids. In this paper, a method was developed for quantifying folding propensity as a function of REMO sequence.

Automated solid phase synthesis was used to obtain a series of REMO that have a phosphine oxide recognition unit (**A**) at one end of the chain and a complementary 4-nitrophenol recognition unit (**D**) at the other. The two recognition units were separated by 0 to 8 blanks (**O**), which are triazines equipped with alkyl side chains instead of recognition units. The wavelength of the UV–vis absorption maximum due to the 4-nitrophenol unit provides a direct readout of the extent of base-pair formation in these oligomers, and all of the **AO**_**n**_ **D** sequences show clear evidence of 4-nitrophenol·phosphine oxide H-bonding interactions. Denaturation experiments using perfluoro-*t*-butanol, which is a very strong H-bond donor and weak H-bond acceptor, were used to break up the base-pairing interactions by H-bonding to the phosphine oxide recognition units. This process was monitored by observing changes in the UV–vis absorption spectrum as the non-H-bonded 4-nitrophenol was released. Denaturation experiments were carried out at different oligomer concentrations to distinguish intermolecular base-pairing interactions from intramolecular folding: population of a duplex or supramolecular polymer is concentration-dependent but the folded single strand is not.

The results were used to determine the effective molarities for intramolecular folding, EM_f_, as a function of chain length. 1,2-folding was not detected, and **AD** forms exclusively the **AD**·**AD** duplex. The other oligomers fold, and in most cases, the value of EM_f_ is around 1 mM, which results in a population of two-thirds for the folded state with a single intramolecular base-pair in dichloromethane solution. The exceptions are the **AO**_**3**_ **D** and **AO**_**4**_ **D** sequences, where the value of EM_f_ is 5 mM, leading to 90% population of the folded state in dichloromethane.

Conformational analysis of the REMO backbone suggests that the piperazine units prefer the chair conformation with the piperazine nitrogen atoms trigonal planar in conjugation with the triazine ring. The lowest energy conformations of the backbone are roughly planar placing the triazine units on a hexagonal grid. This structure allows the terminal recognition units in **AO**_**3**_ **D** and **AO**_**4**_ **D** to come into close proximity, favoring the intramolecular base-pairing interactions involved in 1,5- and 1,6-folding.

A second series of oligomers with two recognition units at each end of the chain were synthesized, and folding was investigated using the same denaturation experiment. **ADO**_**n**_ **AD** sequences encode a hairpin loop structure, whereas **DAO**_**n**_ **AD** sequences encode a helical structure. In these systems, the fully folded single strand makes two base-pairing interactions, so in addition to the effective molarities for the two intramolecular interactions, folding is governed by a cooperativity parameter, α, which quantifies how much the two H-bonds affect one another. Two of the hairpin sequences, **ADO**_**4**_ **AD** and **ADO**_**5**_ **AD**, showed strong positive cooperativity (α > 10), but **ADO**_**3**_ **AD** did not, which indicates that at least four looped out bases are required to form a stable REMO hairpin loop. One of the helical sequences, **DAO**_**4**_ **AD**, showed strong positive cooperativity, which is consistent with the hexagonal grid conformational model for the backbone. A 60° turn per residue aligns the complementary recognition sites at positions 1 and 7 and positions 2 and 8.

The experiments described here lay the foundations for exploration of more complicated folds using the REMO system. The sequence-structure relationships identified using the relatively simple oligomers described above already highlight some particularly favorable folding motifs, where the positions of complementary recognition sites fall in register with the conformational properties of the triazine-piperazine backbone. Moreover, the observation of well-defined stable folded conformations suggests that sequence-encoded functional properties are a realistic possibility for these nonbiological polymers.

## Supplementary Material



## Notes

Materials and methods, detailed synthetic procedures, characterization including NMR spectra, UPLC traces and HRMS of all compounds, X-ray crystal structures, and NMR and UV–vis absorption titration data and data fitting analysis (PDF)
