# Supplementary material for: Sequence-Dependent Folding of Recognition-Encoded Melamine Oligomers
Source: J Am Chem Soc. 2026 Jul 1;148(27):29094–106. doi: 10.1021/jacs.6c07701 (PMC13383713; doi:10.1021/jacs.6c07701)
Supplement: Supplementary file 1 [file ja6c07701_si_001.pdf]

# Sequence-Dependent Folding of Recognition-Encoded Melamine Oligomers.

Anca-Luiza Cotîrlan, Cecilia J. Anderson, Nia E. J. Eyre, Daniil O. Soloviev, Ben Iddon, Federica Balduzzi and Christopher A. Hunter*

Yusuf Hamied Department of Chemistry, University of Cambridge, Lensfield Road, Cambridge CB2 1EW, UK. Email: herchelsmith.orgchem@ch.cam.ac.uk.

# Supplementary information

Synthesis ........ S4
Building blocks ........ S5
**1** ........ S6
**2** ........ S7
**3** ........ S7
**4** ........ S8
**5** ........ S10
**Resin** ........ S12
**6** ........ S12
**7** ........ S12
**8** ........ S12
**9** ........ S13
Small oligomers ........ S16
**yD*n** ........ S17
**yAn** ........ S19
**AA** ........ S22
**DD** ........ S22
**AD** ........ S24
**D*OD*** ........ S27
**DOD** ........ S29
**AOA** ........ S31
General procedures for SPS and oligomers ........ S34
**AOD** ........ S35
**AOOD** ........ S37
**AOOOD** ........ S39
**AOOOOD** ........ S41

**$AO_5D$** ........ S43
**$AO_6D$** ........ S45
**$AO_7D$** ........ S47
**$AO_8D$** ........ S49
**$DAO_3ADy$** ........ S51
**$DAO_4AD$** ........ S54
**$ADO_3AD$** ........ S56
**$ADO_4AD$** ........ S58
**$ADO_5AD$** ........ S60
X-ray structure of REMO 2-mer ........ S62
**$S_a$** ........ S62
**$S_b$** ........ S63
**$S_c$** ........ S65
**$S_d$** ........ S67
Titration data ........ S71
**A**•PFTB ........ S71
**A•D** ........ S74
**AA•DD** ........ S75
**AOA•DOD** ........ S76
**$AO_nD$**•PFTB fitted to 1:1 binding isotherm ........ S77
**AD**•PFTB fitted to 1:1 binding isotherm ........ S77
**AOD**•PFTB fitted to 1:1 binding isotherm ........ S79
**AOOD**•PFTB fitted to 1:1 binding isotherm ........ S81
**AOOOD**•PFTB fitted to 1:1 binding isotherm ........ S83
**AOOOOD**•PFTB fitted to 1:1 binding isotherm ........ S85
**$AO_5D$**•PFTB fitted to 1:1 binding isotherm ........ S87
**$AO_6D$**•PFTB fitted to 1:1 binding isotherm ........ S89
**$AO_7D$**•PFTB fitted to 1:1 binding isotherm ........ S91
**$AO_8D$**•PFTB fitted to 1:1 binding isotherm ........ S93
**$AO_nD$**•PFTB fitted to complex models ........ S95
**AD**•PFTB fitted to duplex model ........ S95
**AOD**•PFTB fitted to duplex+folding model ........ S98
**AOOD**•PFTB fitted to folding model ........ S101
**AOOOD**•PFTB fitted to folding model ........ S104
**AOOOOD**•PFTB fitted to folding model ........ S106
**$AO_5D$**•PFTB fitted to folding model ........ S108

**$AO_6D$**•PFTB fitted to folding model ........ S110
**$AO_7D$**•PFTB fitted to folding model ........ S112
**$AO_8D$**•PFTB fitted to folding model ........ S114
**$DAO_nAD$**•PFTB and **$ADO_nAD$**•PFTB fitted to 1:1 binding isotherm ........ S116
**$DAO_3AD$**•PFTB fitted to 1:1 binding isotherm ........ S116
**$DAO_4AD$**•PFTB fitted to 1:1 binding isotherm ........ S118
**$ADO_3AD$**•PFTB fitted to 1:1 binding isotherm ........ S120
**$ADO_4AD$**•PFTB fitted to 1:1 binding isotherm ........ S122
**$ADO_5AD$**•PFTB fitted to 1:1 binding isotherm ........ S124
**$DAO_nAD$**•PFTB and **$ADO_nAD$**•PFTB fitted to duplex model ........ S126
**$DAO_3AD$**•PFTB fitted to duplex model ........ S128
**$DAO_4AD$**•PFTB fitted to duplex model ........ S130
**$ADO_3AD$**•PFTB fitted to duplex model ........ S132
**$ADO_4AD$**•PFTB fitted to duplex model ........ S134
**$ADO_5AD$**•PFTB fitted to duplex model ........ S136
**$DAO_nAD$**•PFTB and **$ADO_nAD$**•PFTB fitted to folding model ........ S138
**$DAO_3AD$**•PFTB fitted to folding model ........ S140
**$DAO_4AD$**•PFTB fitted to folding model ........ S143
**$ADO_3AD$**•PFTB fitted to folding model ........ S146
**$ADO_4AD$**•PFTB fitted to folding model ........ S149
**$ADO_5AD$**•PFTB fitted to folding model ........ S152

# Synthesis

All the reagents and materials used in the synthesis of the compounds described below were bought from commercial sources, without prior purification. Dry THF, DCM and toluene were obtained from Grubbs PS-MD-5 and used with no further degassing. Dry DCM and methanol were obtained from the departmentally managed stills.

Thin layer chromatography was on silica gel 60F (Merck) on glass plates. Flash chromatography was carried out on an automated system (Combiflash Rf+ or Combiflash Rf Lumen) using prepacked cartridges of silica (25µ PuriFlash® columns). FT-IR spectroscopy was carried out using a PerkinElmer Spectrum One spectrometer equipped with an ATR cell. HRMS spectroscopy was carried out using Agilent 1100 HPLC coupled with Waters LCT Premier TOF. The LCMS analysis of samples was performed using Waters Acquity H-class UPLC coupled with a single quadrupole Waters SQD2. Two methods have been used.

- ACQUITY UPLC BEH C4 Column, 300Å, 1.7 µm, 2.1 mm X 50 mm was used as the UPLC column. The conditions of the UPLC method are as follows: Solvent A: Water +0.1% Formic acid; Solvent B: Tetrahydrofuran +0.1% Formic acid; Gradient of 0-4 minutes 30% - 100%B + 2 minute 100% B with re-equilibration time of 2 minutes. Flow rate: 0.4 ml/min; Column temperature of 40 °C; Injection volume of 2 µL. The signal was monitored at 254 nm and 280 nm.
- ACQUITY UPLC CSH C18 Column, 130Å, 1.7 µm, 2.1 mm X 50 mm, was used as the UPLC column. The conditions of the UPLC method are as follows: Solvent A: Acetonitrile +0.1% Formic acid; Solvent B: THF +0.1% Formic acid; Gradient of 0-1 minutes 0% B, 1-5 minutes 0% to 100% B + 1 minute 100% B with re-equilibration time of 1 minutes. Flow rate: 0.6 ml/min; Column temperature of 40°C; Injection volume of 2 µL. The signal was monitored at 254 nm.

All chemical shifts (δ) are reported in ppm and coupling constants (*J*) reported in Hz. Splitting patterns are reported as follows: s (singlet), d (doublet), t (triplet), q (quadruplet), m (multiplet). Broad signals are reported as 'br'.

Some compounds, display slowly exchanging rotamers in solution the NMR spectra. Where distinguishable, all the corresponding peaks are listed together in square brackets. Where corresponding peaks overlap, they are reported with a chemical shift range.

Molecular structures were drawn with ChemDraw. Titration data were fitted using Musketeer[1]. NMR spectra were analysed using Mestrenova.

# Building blocks

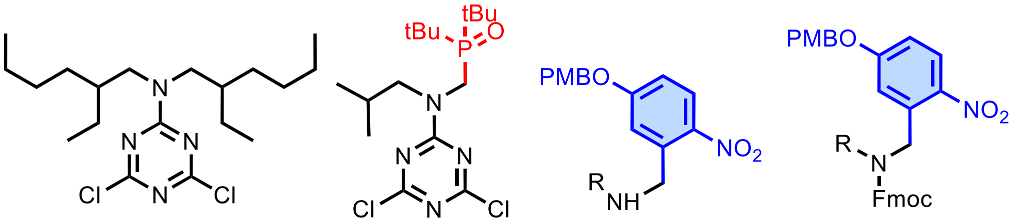


**1** **2** **3** **4**

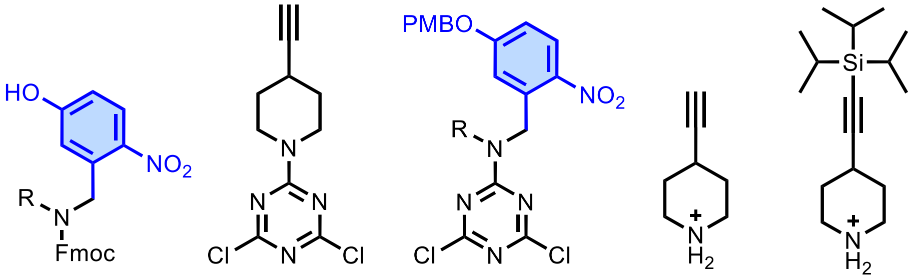


**5** **6** **7** **8** **9**

R = 2-ethylhexyl

*Figure S 1 Summary of small molecules and building blocks used in the synthesis of REMO.*

# 1

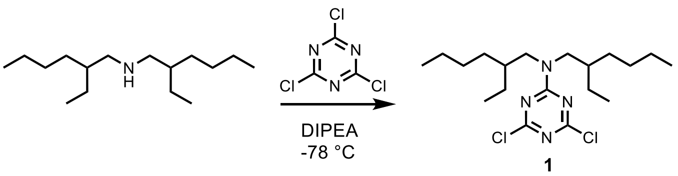


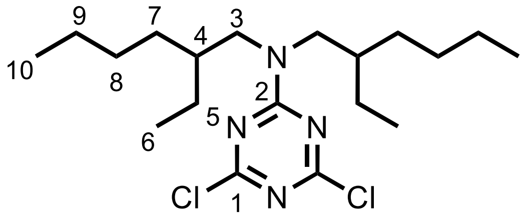

**1**

Cyanuric chloride (6.2 g, 31 mmol, 1.03 eq.) and potassium carbonate (6.9 g, 50 mmol, 1.7 eq.) were dissolved in dry THF (150mL) and cooled to -78 °C. Bis(2-ethylhexyl)amine (7.2 g, 30 mmol) was added dropwise, and the reaction stirred at -78 °C for 2h, then dissolved in ethyl acetate (200 mL), washed with HCl sol. (1M, 3 x 200 mL), brine (2 x 100 mL) and water (2 x 100 mL). The organic phase was dried over anhydrous magnesium sulfate and the solvent removed *in vacuo* to obtain **7** as a colorless oil (6 g, 15.5 mmol, 51%).

**$^{1}$H NMR** (400 MHz, $CDCl_3$) $\delta_H$ 3.54 - 3,44 (m, 4H, C3H), 1.79-1.71 (m, 2H, C4H), 1.37-1.10 (m, 16H, C5H, C7H, C8H & C9H), 0.88 (t, $^{3}J_{HH}$ = 7.4 Hz, 12H, C6H & C10H).
**$^{13}$C NMR** (101 MHz, $CDCl_3$) $\delta_C$ 169 (C1), 165 (C2), 51.0 (C3), 37.2 (C4), 30.4 (C7), 28.5 (C5), 23.8 (C8), 23.1 (C9), 14.1 (C10), 10.7 (C6).
**HRMS (ES+):** calcd. for $[C_{19}H_{34}N_4Cl_{2}+H]^+$ is 388.2161, found 388.2152 (-2.20 mDa).
**FT-IR (ATR):** $\nu_{max}$/cm$^{-1}$ 2959, 2929, 2873, 2860, 1566, 1551, 1504, 1474, 1440, 1380, 1352.

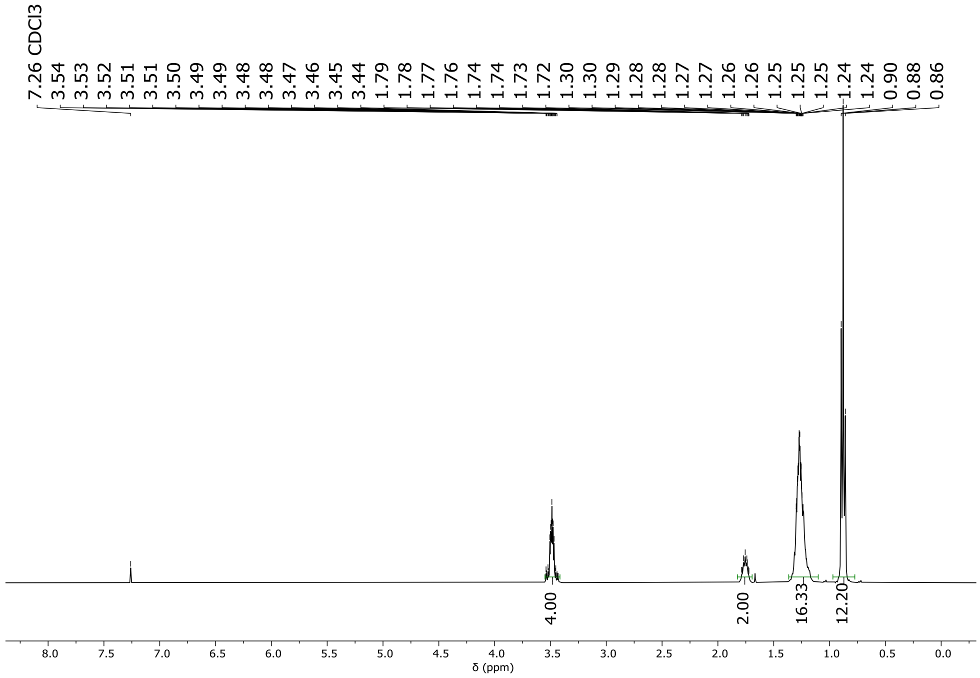


*Figure S 2 $^{1}$H NMR (400 MHz, $CDCl_3$) spectrum of compound **1**.*

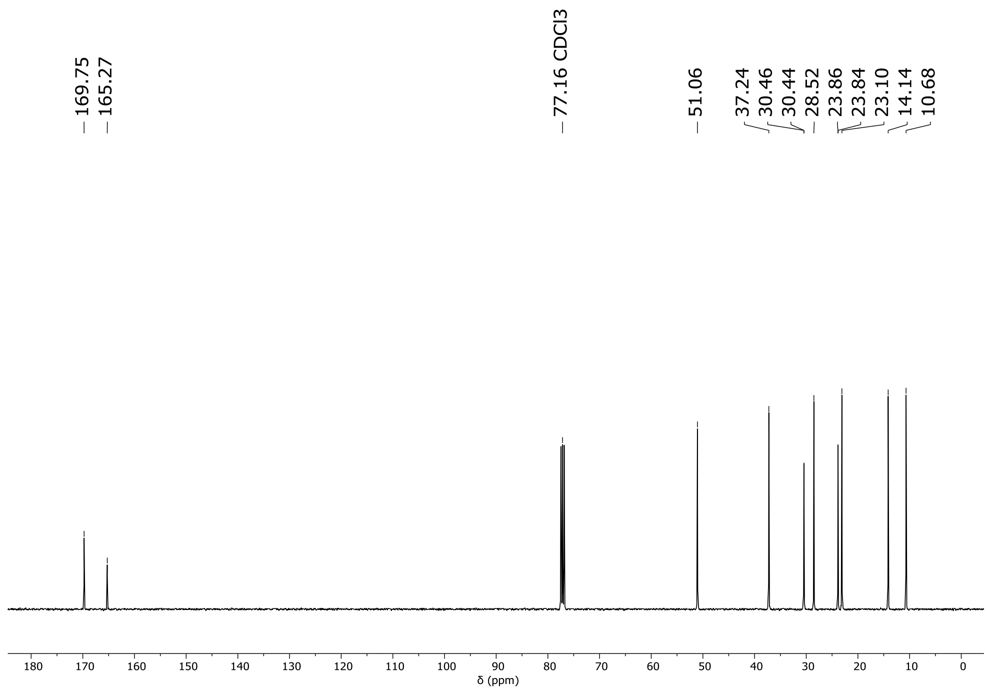


*Figure S 3 $^{13}C$ NMR (101 MHz, $CDCl_3$) spectrum of compound **1**.*

# 2

**2** was synthesis according to literature procedures. [2]

# 3

**3** was synthesis according to literature procedures.[3]

# 4

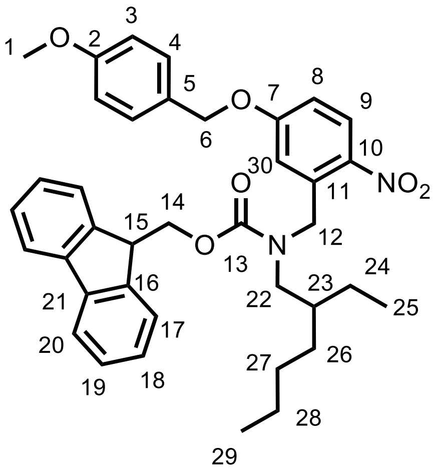


**4**

**3** (1.20 g, 3.0 mmol) was dissolved in anhydrous dichloromethane (12 mL) prior to the slow addition of fluorenylmethyloxycarbonyl chloride (0.93 g, 3.6 mmol) while stirring at 0 °C. Triethylamine (0.54 mL, 3.9 mmol) was added dropwise and the reaction was left stirring overnight. The reaction mixture was diluted with dichloromethane (15 mL), then washed with potassium carbonate (2 x 20 mL), brine (2 x 30 mL) and dried over anhydrous magnesium sulfate. The solvent was then removed in vacuo and the intermediate product was purified by flash column chromatography ($SiO_2$, 0-20%, ethyl acetate in petroleum ether) to yield **4** as a pale yellow oil (1.58 g, 2.5 mmol, 84%).

**$^1$H NMR** (400 MHz, $CDCl_3$) $\delta_H$ [8.20 (d, $^3J_{HH}$ = 9.1 Hz), 8.12 (d, $^3J_{HH}$ = 9.0 Hz), 1H, C9H], [7.75 (d, $^3J_{HH}$ = 7.6 Hz, 1H), 7.59 (m, 1H), C20H], [7.59 (m, 1H) & 7.37 (m, 1H) C20H], [7.37 (m, 1H) & 7.11 (m, 1H) (C18H)], 7.35 (m, 5H, C5H, C18H&C19H), 6.94 – 6.83 (m, 3H, C3H&C8H), 6.66 – 6.57 (m, 1H, C30H), 4.94 (m , 2H, C12H), [4.81 (m, 1H) & 4.42 (m, 1H) (C14H)], 4.65 (m, 2H, C6H), 4.23 - 4.04 (m, 1H, C15H), 3.76 (m, 3H, C1H), 3.1 - 2.84 (m, 2H, (C22H)), 1.61 (s, 1H, C23H), 1.42 – 1.10 (m, 23H), 1.4-0.9 (m, 8H, C28H, C27H, C26H & C24H), 0.9-0.6 (m, 6H, C25H&C29H).
**$^{13}$C NMR** (101 MHz, $CDCl_3$) $\delta_C$ 164 (C7), 160 (C2), 157 (C13), 144 (C21), 142 (C16), 130 &129 (C4), 128.80 (C9), 127.95 (C19), 127.26 (C18), 125 (C18), 120.4 & 120.2 (C20), 115 (C5), 114 (C3), 113 (C30) , 70.7 (C6), 67.6 (C14), 55.7 (C1), 53.9 (C12 or C22), 52.3 (C12 or C22), [49.62 & 47.55 (C15)], 38.34 (C23), 30.79 (C26 or C24), 29.02 (C26 or C24), 24.100 (C27), 23.50 (C28), 14.48 (C29 or C25), 10.94 (C29 or C25).
**HRMS (ES+):** calcd. for $[C_{38}H_{42}N_2O_6+H]^+$ is 622.3043, found 622.3048 (0.05 mDa)
**FT-IR (ATR):** $v_{max}$/cm$^{-1}$ 2960, 2933, 2903, 2875, 2862, 2359, 2252, 1696, 1613, 1579, 1514, 1453, 1429.

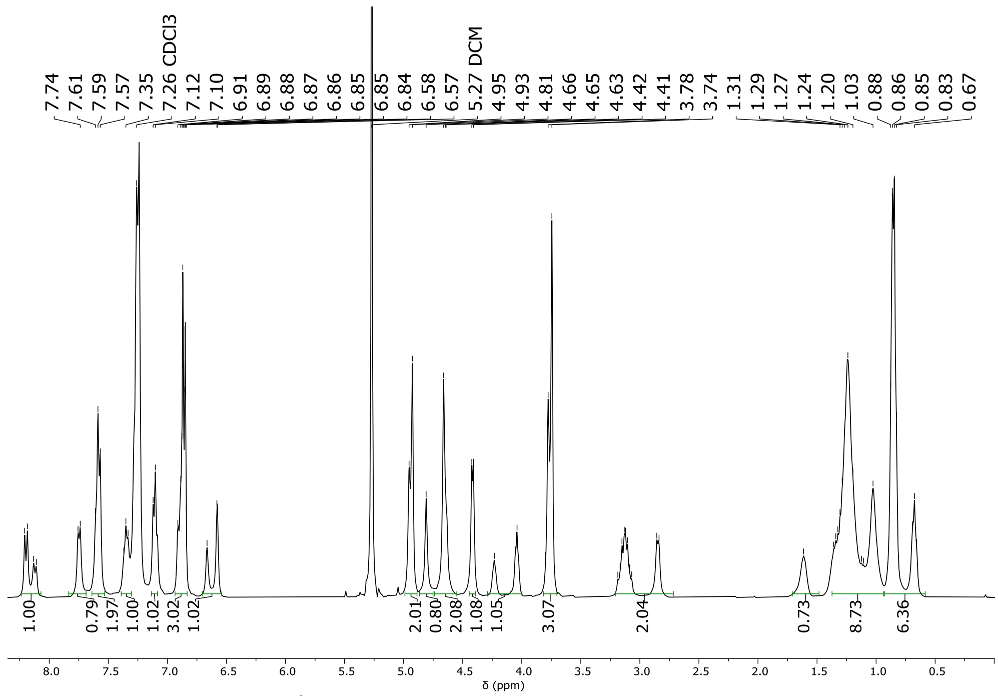


*Figure S 4 $^{1}$H NMR (400 MHz, $CDCl_3$) spectrum of compound **4**.*

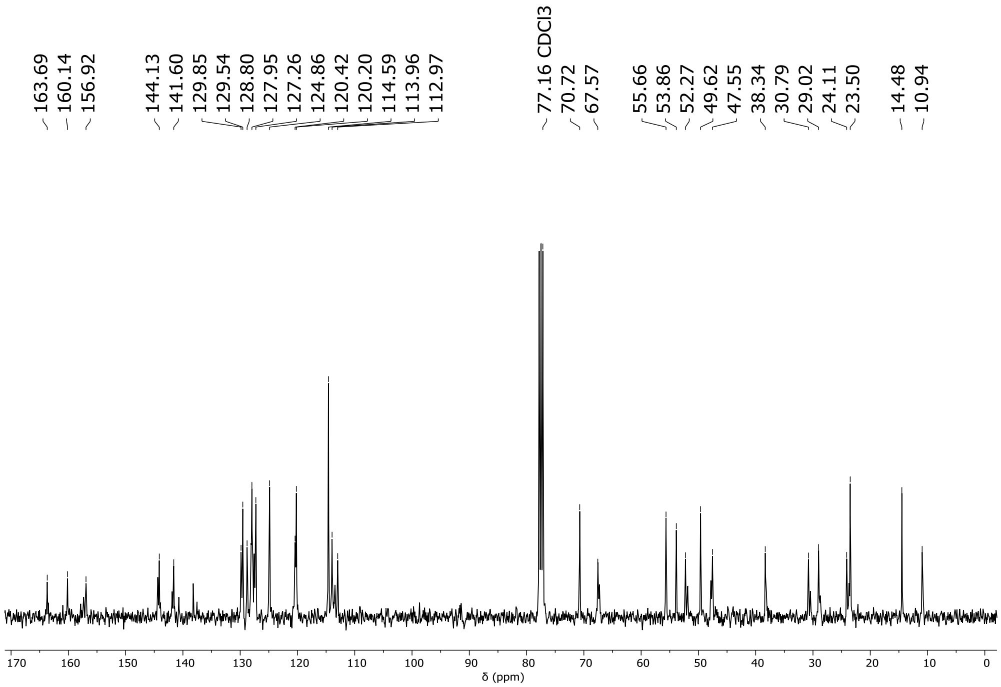


*Figure S 5 $^{13}$C NMR (101 MHz, $CDCl_3$) spectrum of compound **4**.*

**5**

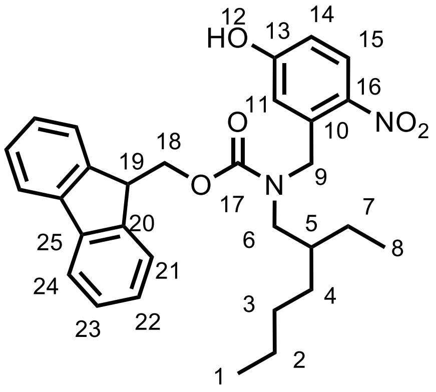


**5**

**4** (1 g, 1.6 mmol) was dissolved in a 9:0.5:0.5 mixture of TFA:TIS:DCM (10 mL), stirred for 1h then the solvent flushed with nitrogen for 72h. The residue was dissolved in DCM (20 mL) and washed with sat. sodium bicarbonate solution (2 x 30 mL), brine (10 mL) and water (10 mL). The organic phase was dried over anhydrous magnesium sulfate and the solvent removed *in vacuo*. The crude was purified by flash column chromatography ($SiO_2$, 0-20% methanol in DCM) to obtain **5** as a white powder (0.8 g, 1.6 mmol, 99%).

**$^1$H NMR (400 MHz, TCE at 373 K)** $\delta_H$ 8.09 (d, $^3J_{HH}$ = 8.9 Hz, 1H, C15H), 7.74 (d, $J$ = 7.6 Hz, 2H, C24H), 7.50 (d, $^3J_{HH}$ = 7.4 Hz, 2H, C21H), 7.40 (t, $^3J_{HH}$ = 7.5 Hz, 2H, C22H or C23H), 7.29 (t, $^3J_{HH}$ = 7.5 Hz, 2H, C22H or C23H), 6.84 (dd, $^3J_{HH}$ = 8.9, $^4J_{HH}$ = 2.7 Hz, 1H, C14H), 6.55 (d, $J$ = 2.7 Hz, 1H, C11H), 4.77 (s, 2H, C9H), 4.62 (d, $J$ = 5.5 Hz, 2H, C18H), 4.19 (q, $^3J_{HH}$ $J$ = 6.7 Hz, 1H, C19H), 3.13 (s, 2H, C6H), 1.59 (s, 1H, C5H), 1.31 – 1.20 (m, 8H, C2H, C3H, C4H, C7H), 0.93 (t, $J$ = 7.1 Hz, 3H, C1H or C8H), 0.85 (t, $^3J_{HH}$ = 7.4 Hz, 3H, C1H or C8H). Contains traces of ethyl acetate.

**$^{13}$C NMR (176 MHz, $CDCl_3$)** $\delta_C$ 163.1 (C13), 157.5 (C17), 143.2 (C25), 141.2 (C20), 139.6 (C10), 137.1 (C16), 129.3 (C15), 127.9 (C23), 127.1 (C22), 124.5 (C21), 120.0 (C24), 115.0 (C14), 112.8 (C11), 68.3 (C18), 51.9 C(6), 49.4 (C9), 46.9 (C19), 37.9 (C5), 30.3 (C4 or C7), 28.6 (C4 or C7), 23.6 C3), 23.1(C2), 14.1 (C1 or C8), 10.5 (C1 or C8).

**HRMS (ES+):** calcd. for $[C_{30}H_{34}N_2O_5+H]+$ 503.2546, found 503.2547 (-1.17 ppm).
**FT-IR (ATR):** $\nu_{max}$/cm$^{-1}$ 2972, 2927, 2894, 2877, 1682, 1598, 1580, 1519, 1451, 1379, 1332.

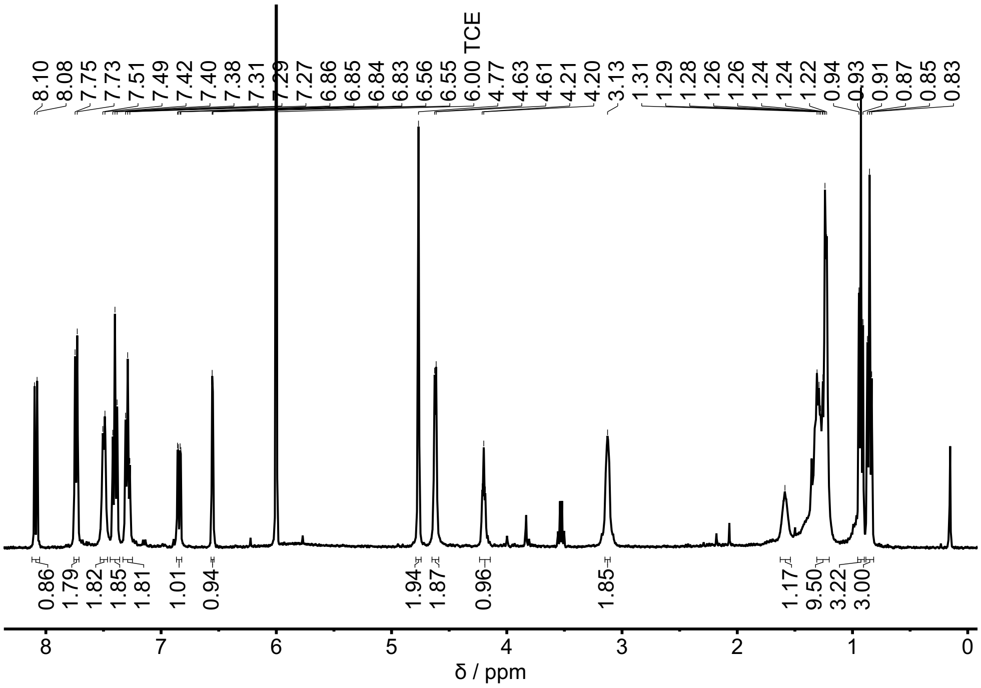


*Figure S 6 $^{1}$H NMR (400 MHz, TCE at 373 K) spectrum of compound **5**.*

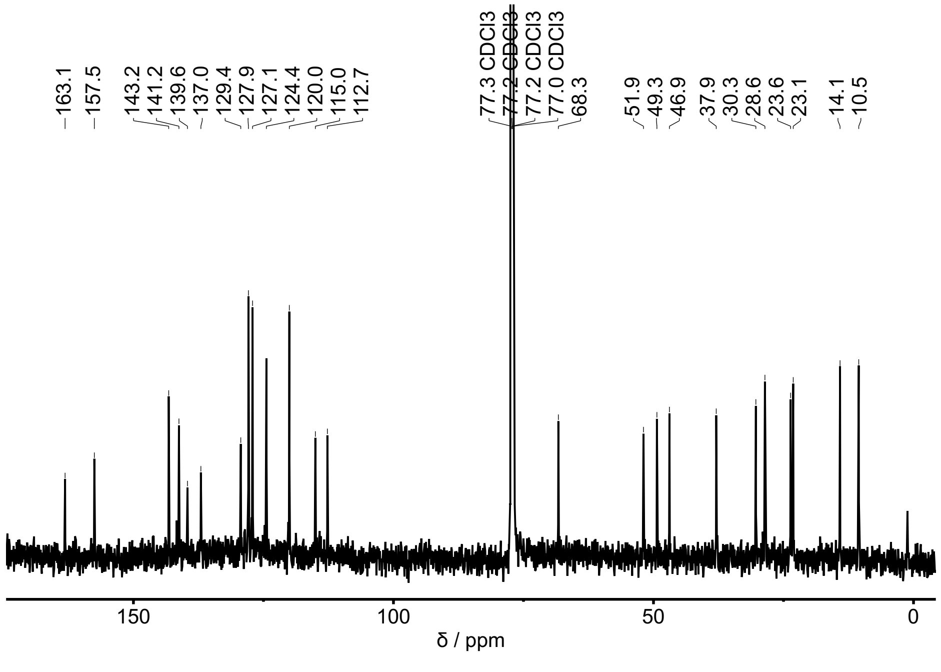


*Figure S 7 $^{13}$C NMR (176 MHz, $CDCl_3$) spectrum of compound **5**.*

# Resin

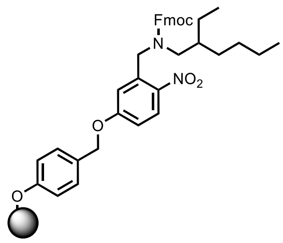

TentaGel S Wang resin (resin-4-hydroxybenzylalcohol) (90 mesh) (2 g, 3 mmol based on advertised loading) was swollen in dry THF (2 mL) for 10 mins before a solution of **14** (0.6 g, 1.2 mmol) and triphenylphosphine (0.32g, 1.2 mmol) in dry THF was added to the resin. A solution of diisopropyl azodicarboxylate (0.25 mL, 240 mg, 1.2 mmol) was diluted with THF (18 mL) then added dropwise to the resin. The resin was heated in a microwave reactor at 70 °C for 45 minutes. Then, it was washed with DMF (5 x 30mL) and then DCM (5 x 30 mL).

**Quantification of resin loading:** 10 mg of functionalised resin was treated with a solution of DBU in DMF (2 mL, 2 vol. %) and stirred for 30 mins. 0.1 mL of the solution was removed from the resin and diluted with 0.4 mL acetonitrile. A 0.1 mL aliquot of the resultant solution was taken and diluted to 2 mL with acetonitrile. The absorbance of the DBU-fulvene adduct ($\lambda = 304$ nm, $\varepsilon = 9254$ $M^{-1}$ $cm^{-1}$) was measured to estimate the resin loading (0.11 mmol $g^{-1}$).

# 6

**6** was synthesised according to literature procedure.[4]

# 7

**7** was synthesised according to literature procedure. [3]

# 8

**8** was generated *in situ* by stirring 1-Boc-4-Ethynylpiperidine in TFA:DCM 1:1 for 2h, then flushing with nitrogen for 12h.

# 9

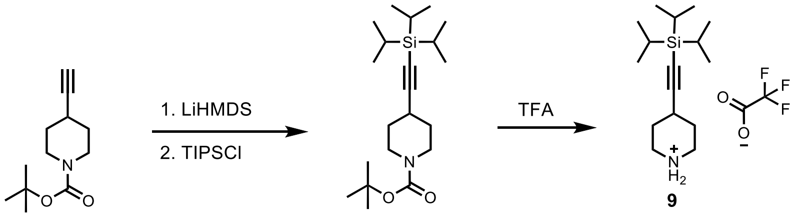


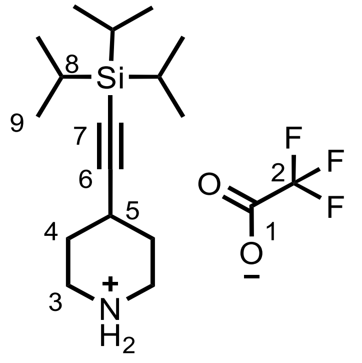

**9**

4-Ethynylpiperidine-1-carboxylic acid tert-butyl ester (10.52 g, 50.3 mmol) was dissolved in THF (100 mL). Lithium bis(trimethylsilyl)amide solution (1.0 M in THF, 55 mL, 55 mmol) was added dropwise to the reaction mixture at –78 °C over 30 minutes. The reaction mixture was stirred for 1 hour at –78 °C prior to the addition of triisopropylsilyl chloride (10.7 mL, 55 mmol) at –78 °C. The reaction was then warmed to room temperature and stirred overnight (15 hours). The reaction was quenched with water (200 mL) and extracted with ethyl acetate (3 x 150 mL). Solvent was removed from the organic phase under reduced pressure to yield a yellow/brown oil which was purified by silica plug (petroleum ether and ethyl acetate). The solvent was removed *in vacuo,* then the crude was dissolved in a mixture of 1:1 DCM:TFA (10 mL) and stirred for 2h. The solvent was removed by flushing with nitrogen to yield **9** as an off-white solid product (quantative yield).

**$^1$H NMR** (400 MHz, $CDCl_3$) $\delta_H$ 9.25 (s, 2H, NH), 3.36 – 3.23 (m, 2H, C3H), 3.19 – 3.11 (m, 2H, C3H'), 2.89 (p, $^3J_{HH}$ = 4.5 Hz, 1H, C5H), 2.15 – 2.02 (m, 2H, C4H), 1.93 – 1.82 (m, 2H, C4H'), 1.14 – 0.95 (m, 21H, C8H&C9H).

**$^{13}$C NMR** (101 MHz, $CDCl_3$) $\delta_C$ 107.8 (C1), 84.3 (C6 & C7), 41.3 (C3), 28.1 (C4), 25.6 (C5), 18.7 (C9), 11.2 (C8).

Identity is confirmed by single crystal X-ray data, molecular structure shown on page S15. For crystal structure and refinement details see page S70.

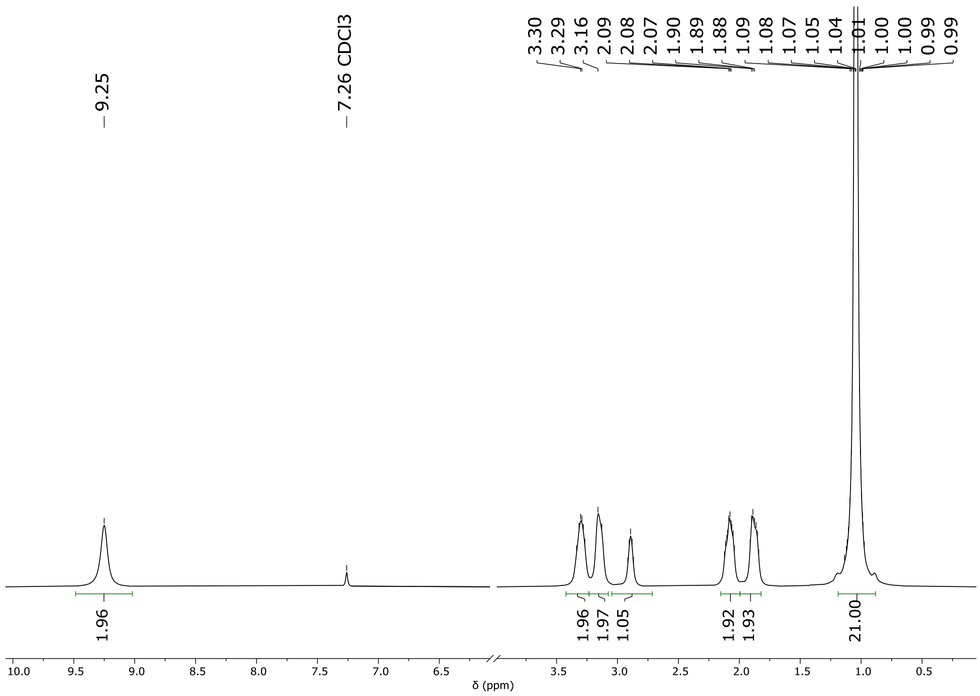


*Figure S 8 $^{1}$H NMR (400 MHz, CDCl$_3$) spectrum of compound **9**.*

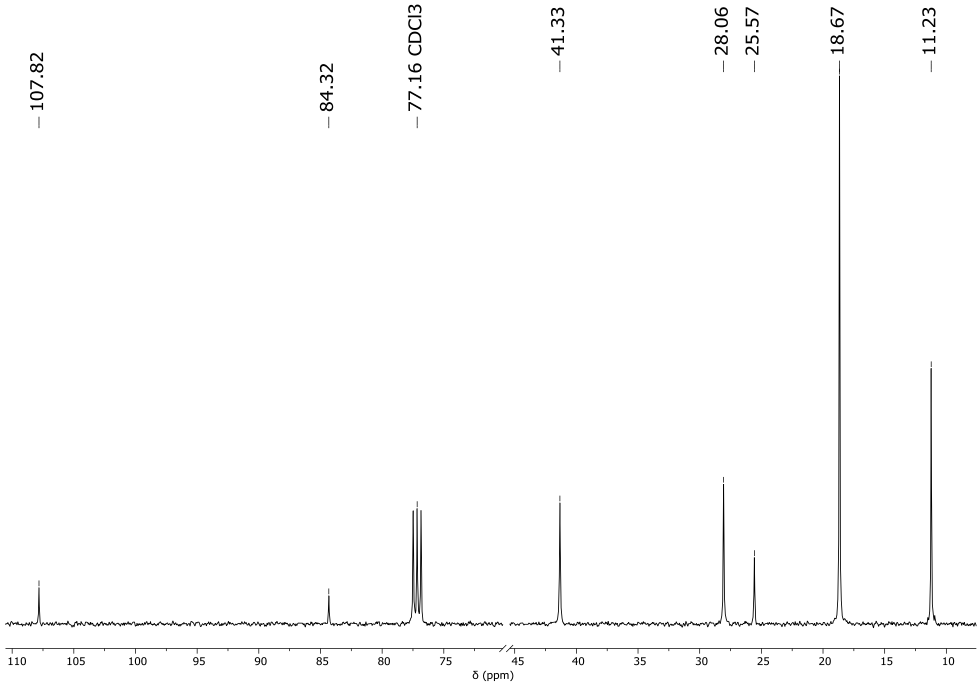


*Figure S 9 $^{13}$C NMR (101 MHz, CDCl$_3$) spectrum of compound **9**.*

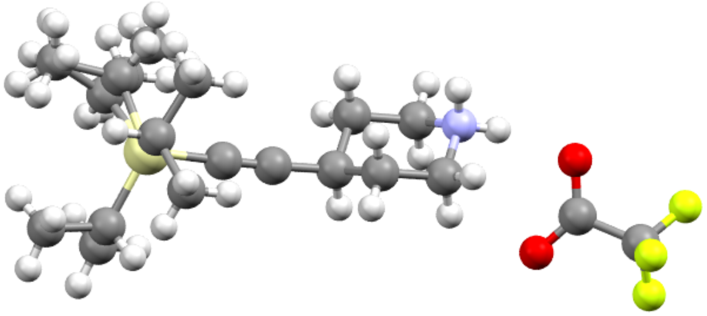

*Figure S 10 Molecular structures of **9** taken from X-ray crystal structure determined by Dr. Andrew D. Bond.*

# Small oligomers

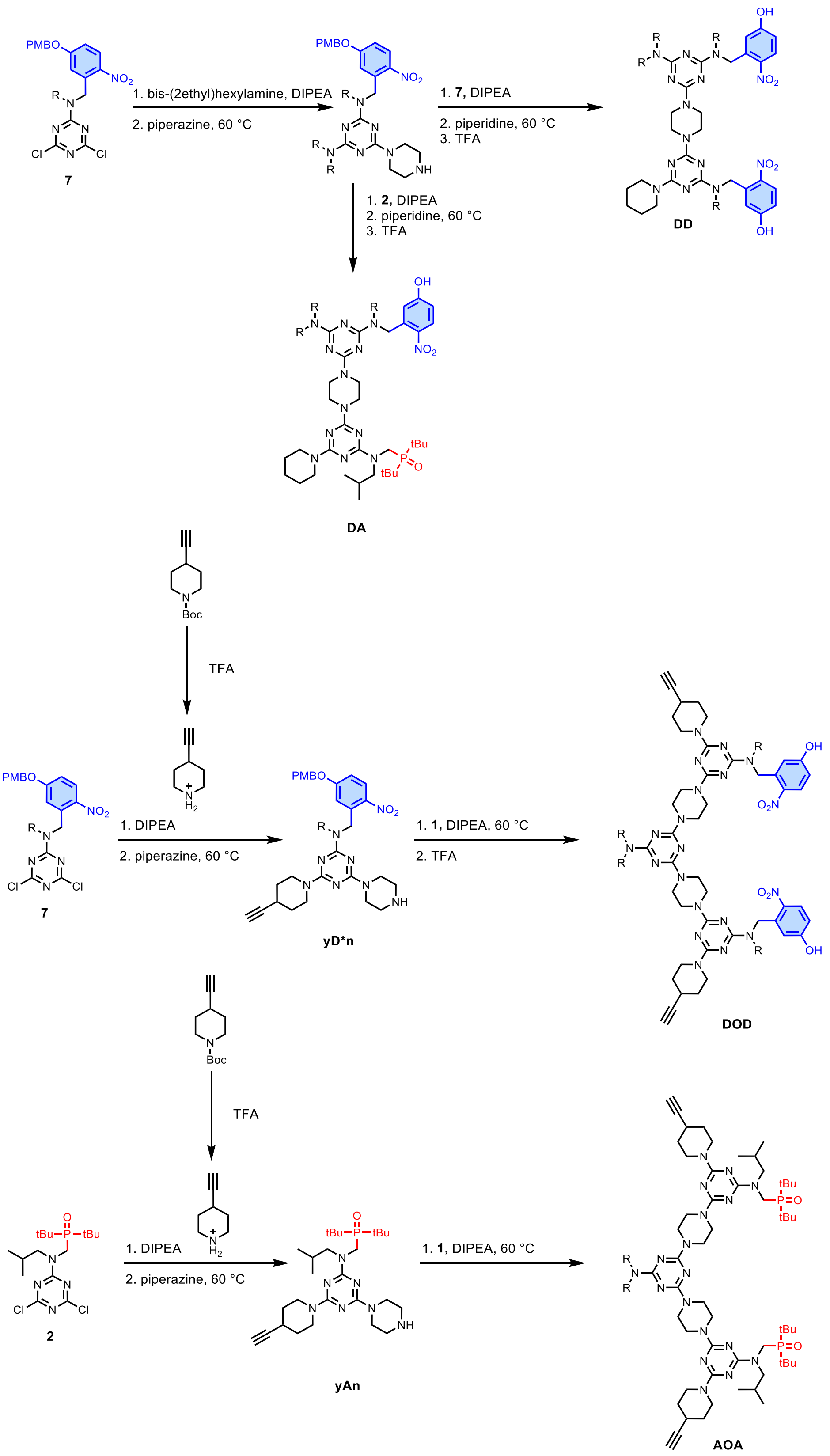


R= 2-ethylhexyl

*Figure S 11 Synthetic strategy for synthesis of* ***DA****,* ***DD****,* ***AOA*** *and* ***DOD****.*

# yD*n

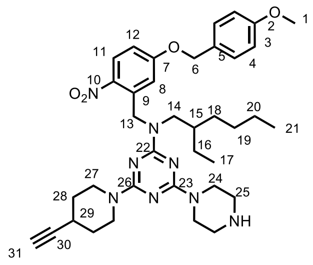

yD*n

TFA (35.8 mL, 467.5 mmol, 32.7 eq.) was added to a solution of 1-Boc-4-ethynylpiperidine (3 g, 14.3 mmol, 1 eq.) in DCM (107.5 mL) and the resulting solution was stirred at room temperature for 30 minutes. The solvent mixture was concentrated by flushing with $N_2$ overnight to give a yellow compound that was used without further purification.

4-ethynylpiperidine salt (326 mg, 1.46 mmol, 1 eq.) was dissolved in THF (14.6 mL) and DIPEA (0.75 mL, 4.39 mmol, 3 eq.) was added. This solution was added dropwise to a solution of **7** (800 mg, 1.46 mmol, 1 eq.) in THF (14.6 mL) and the resulting solution was stirred at 0 °C for 2 hours. When the reaction had gone to completion, piperazine (1.26 g, 14.63 mmol, 10 eq.) was added at 0 °C and the mixture was heated to 60 °C and left to stir overnight. EtOAC (20 mL) was added and washed with water (3 × 10 mL). The organic layer was dried with $MgSO_4$ and concentrated *in vacuo*. The crude product was purified by column chromatography (silica gel, 0-10% methanol in DCM) to give **yD*n** as a yellow oil (720 mg, 1.074 mmol, 74%).

**$^1$H NMR** (500 MHz, $CDCl_3$): $\delta_H$ 8.11 (d, $^3J_{HH}$ = 9.0 Hz, 1H, C11H), 7.28 (d, $^3J_{HH}$ = 7.1 Hz, 2H, C4H), 6.89-6.85 (m, 3H, C3H & C12), 6.74 (dd, $^3J_{HH}$ = 9.9 Hz, $^4J_{HH}$ = 2.8 Hz, 1H, C9H), 5.07 (s, 2H, C13H), 4.98 (s, 2H, C6H), 4.18 (br s, 1H, C27H), 3.85 (t, J= 5.0 Hz, 2H, C27H), 3.81 (s, 3H, C1H), 3.49-3.37 (m, 3H, C24H & C27H), 3.21 (br s, 1H, NH), 2.97 (t, $^3J_{HH}$ = 5.1 Hz, 2H, C24H), 2,88 (br s, 1H, C24H), [2.66 (br s), 2.55 (br s) 1H, C29H], [2.11 & 2.06 (m, 1H, C31H)], 1.86 (m, 1H, C28), 1.80 (br s, 1H, C15H), 1.63-1.35 (m, 3H, C15H), 1.35-1.24 (m, 8H, C20H, C19H, C18H & C16), 0.86 (m, 6H, C17H & C21H).

**$^{13}$C NMR** (126 MHz, $CDCl_3$): $\delta_C$ 165.9 (C=N), 165.1 (C=N), 164.8 (C=N), 162.7 (C7), 159.6 (C2), 141.2 (C10), [139.3, 139.1 (C9)], 129.4 (C4), 127.6 (C11) 127.4 (C5), 114.0 (C8), 112.4 (C12), 86.7 (C30), 70.2 (C31), 55.2 (C1), 50.9 (C14), 48.8 (C13), [45.3 44.9, 43.2, 42.9 (C24 & C25)], [41.5, 41.4 (C27)], 38.1 (C15), [31.2, 31.0 (C28)] 30.6 (C16 or C18), 28.7 (C16 or C18), 27.2 ( C29), 23.7 (C19), 23.1 (C20), 14.0 (C17 or C21), 10.6 (C17 or C21).

**HRMS (ES+):** calculated for $[C_{37}H_{50}N_8O_4+H]^+$: 671.4033, found: 671.4056 (+3.4 ppm).

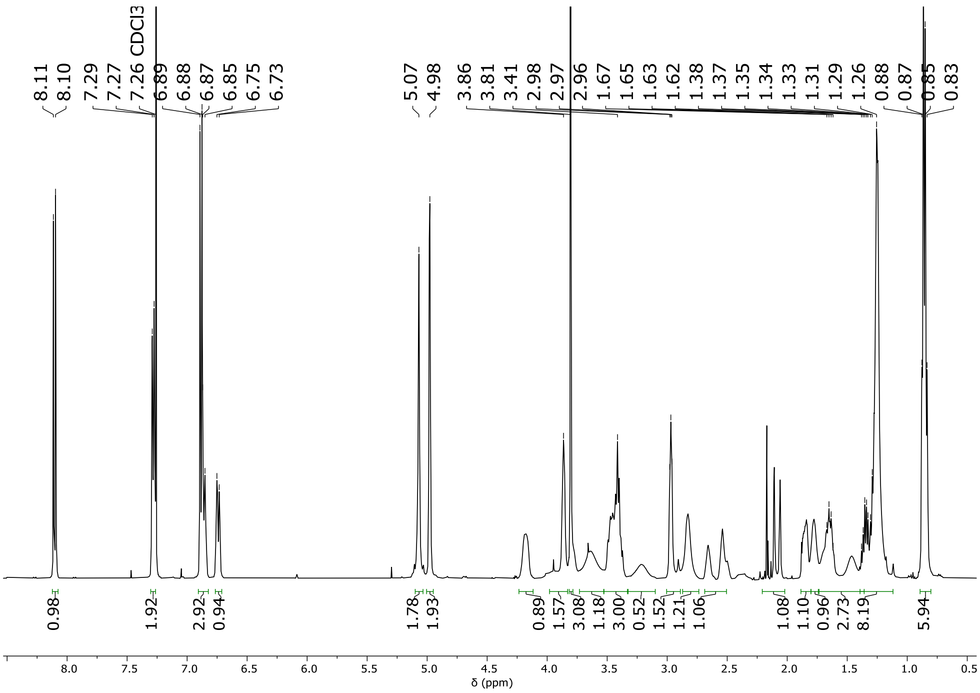


*Figure S 12 $^{1}$H NMR (500 MHz, CDCl$_3$) spectrum of compound* ***yD*n***.

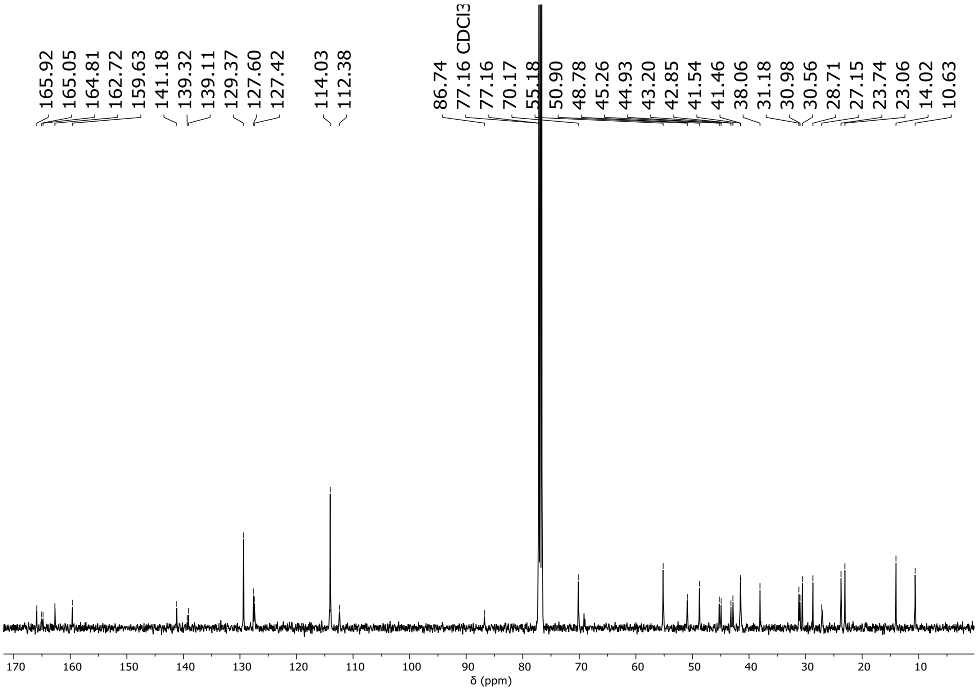


*Figure S 13 $^{13}$C NMR (126 MHz, CDCl$_3$) spectrum of compound* ***yD*n***.

# yAn

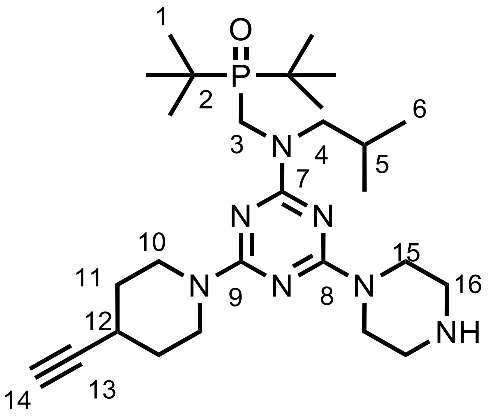

**yAn**

A solution of 4-ethynylpiperidine (452 mg, 2.03 mmol, 1 eq.) and DIPEA (0.95 mL, 6.09 mmol, 3 eq.) in THF (20.3 mL) was added dropwise to a solution of **2** (800 mg, 2.03 mmol, 1 eq.) in THF (20.3 mL) at 0 °C. The reaction was stirred for 2 hours at 0°C and monitored by UPLC-MS. Piperazine (1.45 mL, 18.5 mmol, 10 eq.) was added directly at 0 °C and the mixture was heated to 60 °C and stirred overnight. EtOAc (20 mL) was added and the solution was washed with water (3 × 10 mL). The organic layer was dried with $MgSO_4$ and concentrated *in vacuo*. The crude product was purified by column chromatography (silica gel, 0-30% MeOH in DCM) to give **yAn** as a white solid (628 mg, 1.21 mmol, 60%).

**$^1$H NMR** (500 MHz, $CDCl_3$): $\delta_H$ 4.37 (br s, 2H, C3H), 4.15-4.11 (m, 2H, C10H), 3.82 (d, $^3J_{HH}$ = 7.3 Hz, 2H, C4H), 3.74 (t, $J$ = 5.1 Hz, 4H, C15H&C16H), 3.45 (m, 2H, C10H'), 2.87 (t, $^3J_{HH}$ = 4.8 Hz, 4H, C15H &C16H), 2.64 (s, 1H, C12H), 2.20-2.16 (m, 1H, C5H), 2.10 (s, 1H, C14H), 1.84-1.80 (m, 2H, C11H), 1.64-1.57 (m, 2H, C11H'), 1.26 (d, $^3J_{PH}$= 12.9, 18H, C1H), 0.86 (d, $^3J_{HH}$ = 6.7 Hz, 6H, C6H)

**$^{13}$C NMR** (126 MHz, $CDCl_3$): $\delta_C$ [165.8-165.0 (C7, C8 & C9)], 87.3 (C13), 69.7 (C14), 53.4 (C4), [46.0, 45.8, 44.0 & 43.7 (C16&C15)], 41.7 (C5), 38.4 (C1), 37.9 (d, $^1J_{PC}$ = 55.3 Hz, C2), 31.7 (C11), 27.35 (C12), 26.8 (C1), 26.5 (C5), 20.9 (C6).

**$^{31}$P NMR** (202 MHz, $CDCl_3$): $\delta_P$ 59.0 (d $^1J_{PC}$ = 59.1 Hz)

**HRMS (ES+):** calculated for $[C_{27}H_{48}N_7OP+H]^+$: 513.3736, found: 518.3759 (+4.4 ppm).

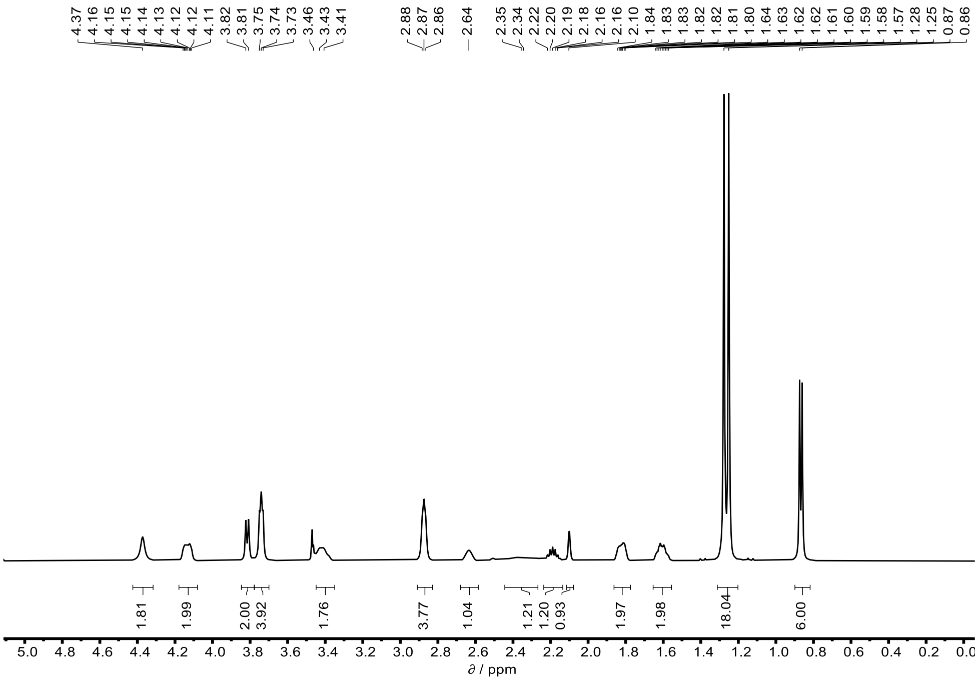


*Figure S 14 $^{1}H$ NMR (500 MHz, $CDCl_3$) spectrum of compound **yAn**.*

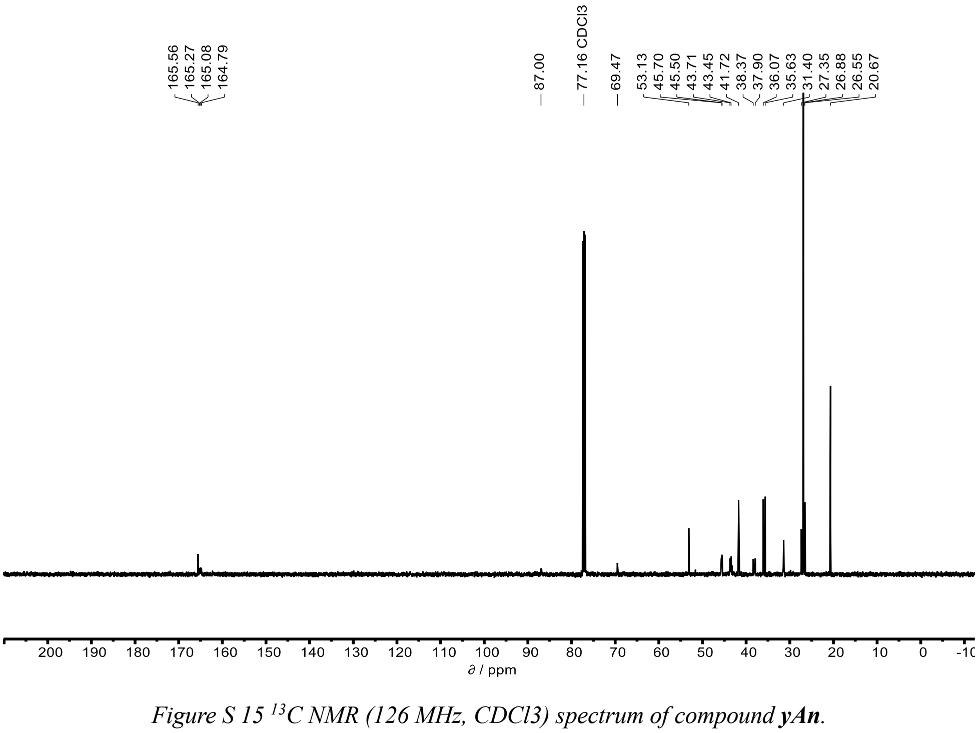


*Figure S 15 $^{13}C$ NMR (126 MHz, CDCl3) spectrum of compound **yAn**.*

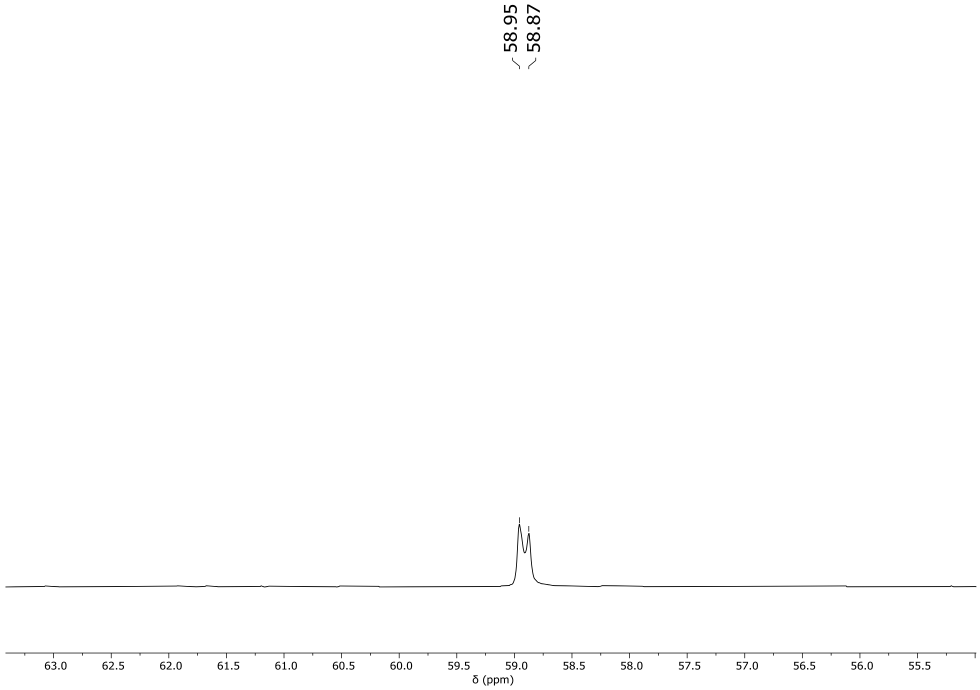


*Figure S 16 $^{31}$P NMR (162 MHz, CDCl3) spectrum of compound **yAn**.*

# AA

**AA** was synthesized according to literature procedures.[2]

# DD

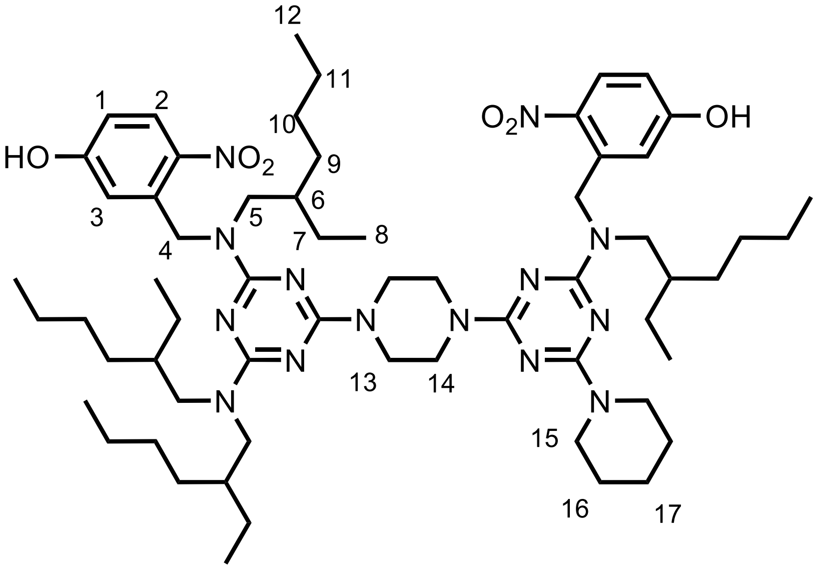

**DD**

**7** (500 mg, 0.9 mmol) was dissolved in THF (5 mL) and 2-ethylhexylamine (438 mg, 0.9 mmol, 1 eq.) and DIPEA (0.3 mL, 1.8mmol, 2 eq.) were added. The reaction was stirred at room temperature for 3h. Piperazine (75 mg, 8 mmol, 3.5 eq.) was added, and the reaction microwaved at 65 °C for 20 minutes. The solution was diluted with DCM (50mL) and washed with HCl sol. (1M, 3 x 100 mL), brine (100 mL) and water (100 mL). The organic phase was dried over anhydrous magnesium sulfate then run through a silica plug by eluting with DCM.

The solvent was removed in vacuo and the residue redissolved in THF (5mL). **7** (500 mg, 0.9 mmol) and DIPEA (0.6 mL, 3.6 mmol, 4 eq.) were added, and the reaction was stirred at room temperature for 3h. Piperidine (1 mL, 9 mmol, 10 eq.) was added, and the reaction microwaved at 65 °C for 20 minutes. TFA (10mL) was added and the reaction stirred for 1h. The solvent was removed by flushing with nitrogen for 48h, the residue was dissolved in DCM (50mL) and washed with HCl sol. (1M, 3 x 100 mL), brine (100 mL) and water (100 mL). The crude was purified by flash column chromatography ($SiO_2$, 0-15% methanol in DCM) to obtain **DD** as a pale-yellow solid (833.5 mg, 0.78 mmol, 85%).

**$^1$H NMR** (400 MHz, $CDCl_3$) $\delta_H$ 8.06 (m, 2H, C1H), 6.82 – 6.28 (m, 4H, C2H&C3H), 5.08 (d, $^3J_{HH}$ = 21.6 Hz, 4H, C4H), 3.99 – 3.02 (m, 20H, C13H, C14H, C15H, C16H& C5H), 2.35 – 2.00 (m, 1H, C17), 1.80 (m, 4H, C5H), 1.49 – 0.99 (m, 32H, C7, C9, C10 & C11), 1.01 – 0.7 (m, 24H, C8 & C12).

**$^{13}$C NMR** (101 MHz, $CDCl_3$) $\delta_C$ 166.4 (C=N), 161.3 (C-OH), 141.5 (C-NO2), 128.6 (C2), 115.1 & 114.1 (C3), 51.5 (C5), 44.7 & 43.5 (C13, C14 & C15) , 38.7 (C6) , 31.1 (C16) , 30.1 (C7 or C9), 29.3 (C7 or C9), 26.3 (C17), 24.3 (C10), 23.6 (C11), 14.6 (C12 or C8), 11.2 (C12 or C8).

**HRMS (ES+):** calcd. for $[C_{61}H_{99}N_{14}O_6+H]^+$ is 1123.7875, found 1123.7867 (+0.78 ppm).

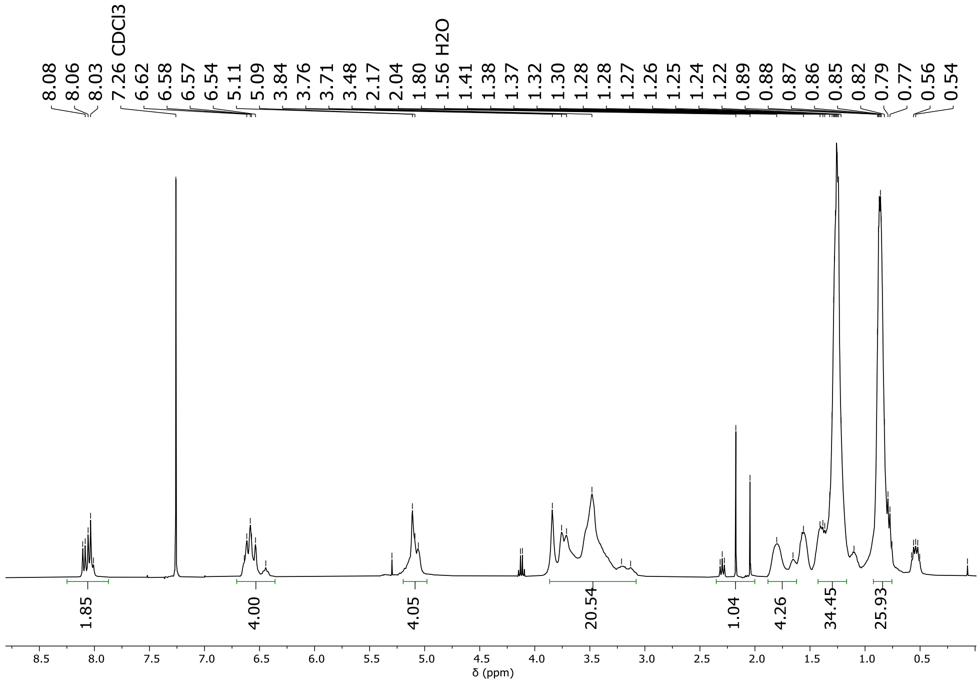


*Figure S 17 $^{1}$H NMR (400 MHz, $CDCl_3$) spectrum of compound **DD**.*

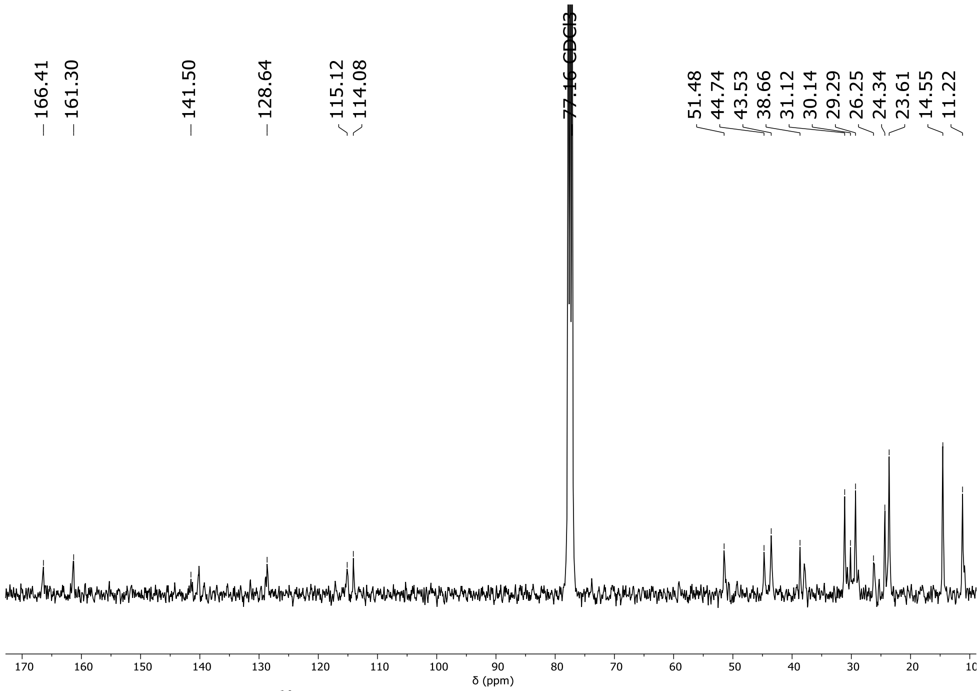


*Figure S 18 $^{13}$C NMR (101 MHz, $CDCl_3$) spectrum of compound **DD**.*

# AD

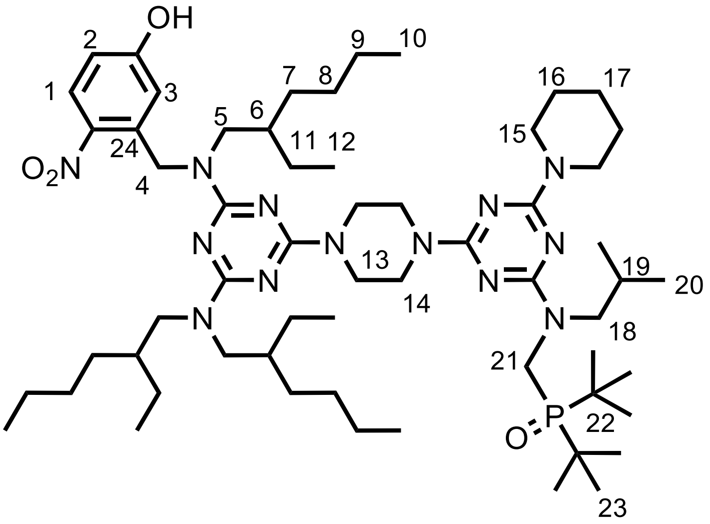


**AD**

**7** (500 mg, 0.9 mmol) was dissolved in THF (5 mL) and 2-ethylhexylamine (438 mg, 0.9 mmol, 1 eq.) and DIPEA (0.3 mL, 1.8 mmol, 2 eq.) were added. The reaction was stirred at room temperature for 3h. Piperazine (387 mg, 4.5 mmol, 5 eq.) was added, and the reaction microwaved at 65 °C for 20 minutes. The solution was diluted with DCM (50mL) and washed with HCl sol. (1M, 3 x 100 mL), brine (100 mL) and water (100 mL). The organic phase was dried over anhydrous magnesium sulfate then run through a silica plug by eluting with DCM.

The solvent was removed *in vacuo* and the residue redissolved in THF (5mL). **3** (259 mg, 0.9 mmol, 1 eq.) and DIPEA (0.6 mL, 3.6 mmol, 4 eq.) were added, and the reaction was stirred at room temperature for 3h. Piperidine (1 mL, 9 mmol, 10 eq.) was added, and the reaction microwaved at 65 °C for 20 minutes. TFA (10mL) was added and the reaction stirred for 1h. The solvent was removed by flushing with nitrogen for 48h, the residue was dissolved in DCM (50mL) and washed with HCl sol. (1M, 3 x 100 mL), brine (100 mL) and water (100 mL). The crude was purified by flash column chromatography ($SiO_2$, 0-15% methanol in DCM) to obtain **AD** as a pale-yellow solid (853.5 mg, 0.78 mmol, 87%).

**$^{1}$H NMR** (400 MHz, $CDCl_3$) $\delta_H$ [8.13 - 8.05 (m, 1H, C1H)], 6.8 (m, 2H, C2H & C3H), 5,40 - 5.10 (m, 2H, C4H), 4.45 (m, 2H, C21H), 4.00-3.00 (m, 24H, C5H, C13H, C14H, C15H & C18H), 2.35&1.90 (m, 2H, C15H), 1.85 -1.70 (m, 3H, C6H), 1.65&1.25 (m, 48H, C7H, C8H, C9H, C11H, C16H, C17H & C23H), 1.00&0.55 (m, 24H, C10H, C12H & C20H).
**$^{13}$C NMR** (176 MHz, $CDCl_3$) $\delta_C$ 165.21 (C=N), 164.9 (C=N), 164.1 (C=N), 160.7 (C-OH), 147.3 (C-NO2), 139.1 (C24), 128.6 (C1), [117.0 & 115.3 (C2)], [114.5 & 114.1 (C3)], [53.6 & 53.1 (C18)], 51.6 (C5), 49.8 (C4), 44.67, 44.23, 43.43 & 42.82 (C13, C14, C15 & C19), 38.23 (C6), 37.45 (d, $^{1}J_{PC}$ = 59.4, C21), [35.86 & 35.55 (d, $^{1}J_{PC}$ = 55.3 Hz, C22)], 30.56 (C16), 30.09 (C7 or C11) 28.74 (C7 or C11), 26.64 (C17), 25.76 (C23), 24.85 (C8), [23.84 & 23.08, (C10), 20.40 (C20), 14.05 (C10 or C12), 10.70 (C10 or C12).
**$^{31}$P NMR** (162 MHz, $CDCl_3$) $\delta_p$ 62.8.
**HRMS (ES+):** calcd. for $[C_{59}H_{105}N_{13}O_4+H]^+$ is 1090.8153, found 1090.8145 (0.78 ppm).

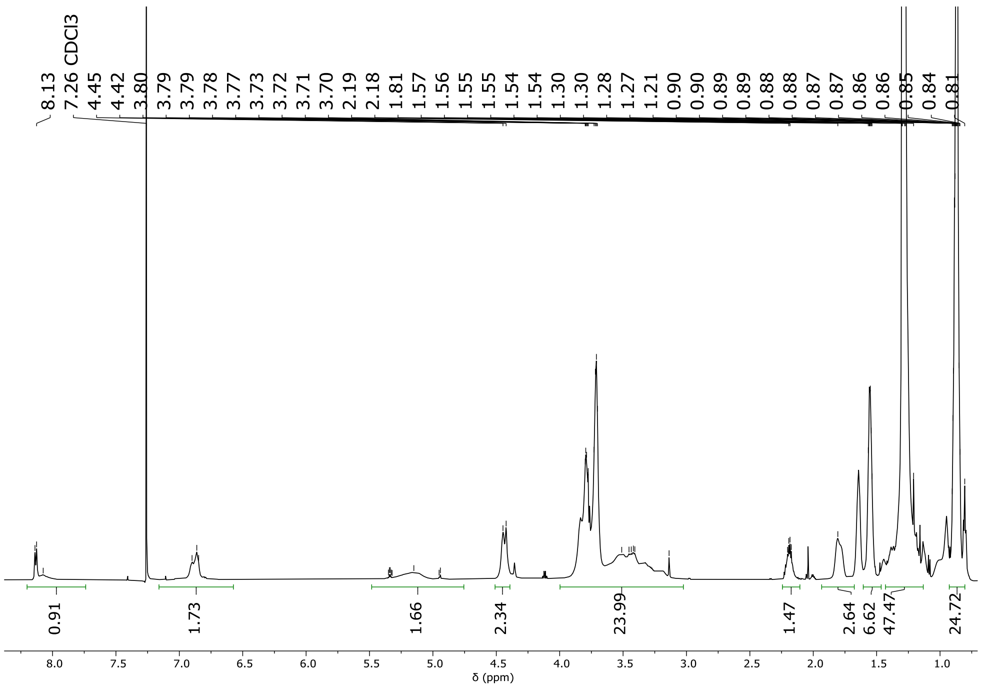


*Figure S 19 ¹H NMR (400 MHz, CDCl3) spectrum of compound **AD**.*

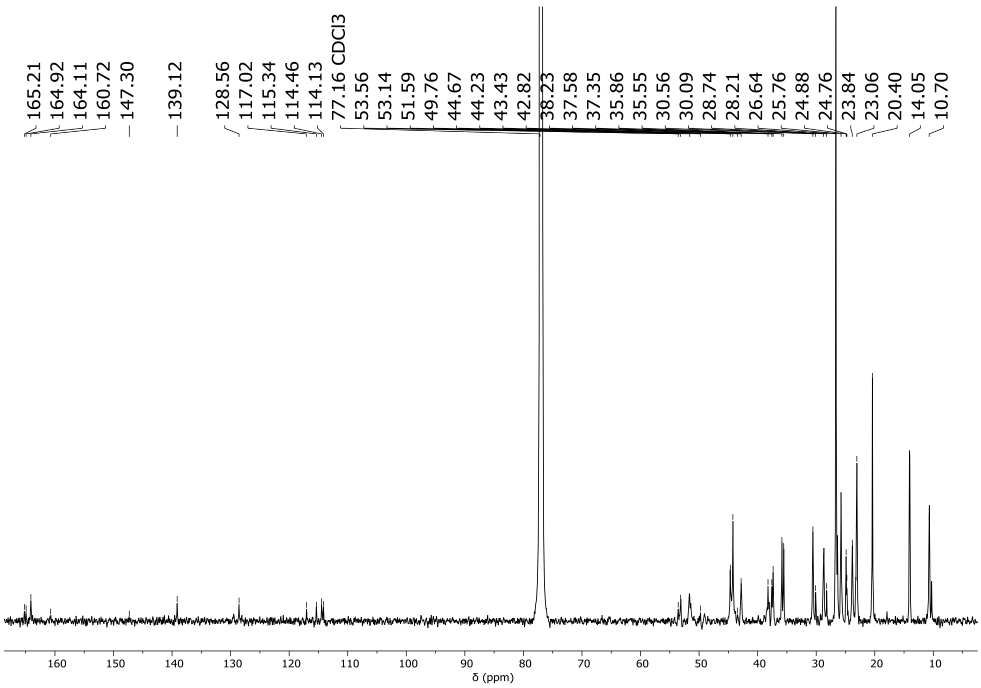


*Figure S 20 ¹³C NMR (176 MHz, CDCl3) spectrum of compound **AD**.*

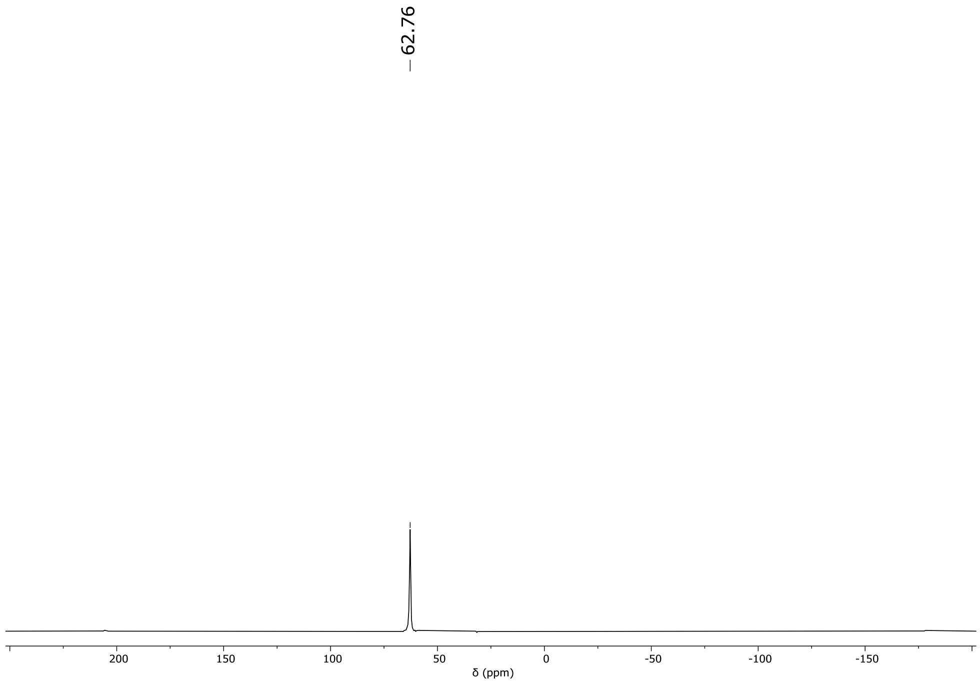


*Figure S 21 $^{31}$P NMR (162 MHz, CDCl3) spectrum of compound **AD**.*

# D*OD*

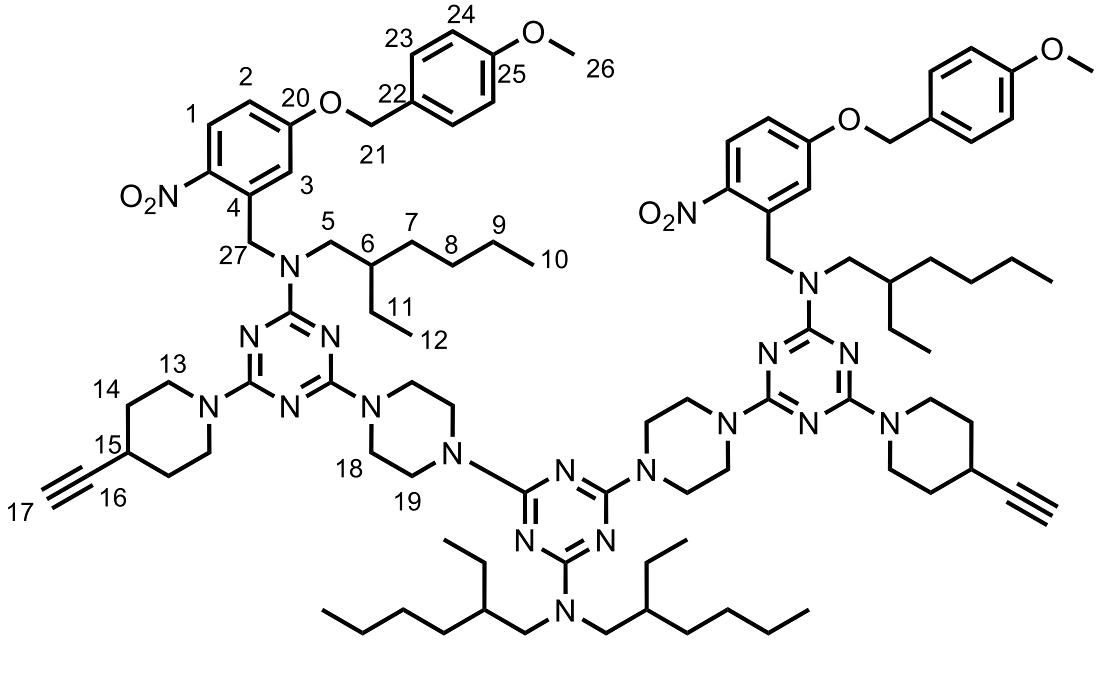

**D*OD***

A solution of compound **yD*n** (400 mg, 0.60 mmol, 5 eq.) in THF (1.2 mL) was added to a solution of compound **1** (46 mg, 0.12 mmol, 1 eq.) in THF (6 mL). DIPEA (0.041 mL, 0.23 mmol, 2 eq.) was added and the solution was stirred at 60 °C for 16 hours. The reaction was monitored by UPLC-MS to ensure that it went to completion. EtOAc (15 mL) was added, and the solution was washed with water (3 × 10 mL), dried with $MgSO_4$ and the solvent was removed *in vacuo.* The crude product was purified by column chromatography (silica gel, 0-100% ethyl acetate in petroleum ether, then 0-10% methanol in ethyl acetate) to give **D*OD*** (122 mg, 0.074 mmol, 61%) as a yellow solid.

**$^1$H NMR** (700 MHz, $CDCl_3$): $\delta_H$ [8.16 (d, $^3J_{HH}$ = 8.9 Hz), 8.13 (d, $^3J_{HH}$ = 9.1 Hz, 2H, C1H, 7.30 (d, $^3J_{HH}$ = 8.8 Hz, 4H, C23H), 6.91-6.88 (m, 6H, C2H&C24H) , 6.80 (m, 2H, C3H), [5.14 & 5.11 (s, 4H, C27H)], 5.00 (s, 4H, C21H), 4.23 (m, 2H, C13H), 3.84-3.41 (m, 36H, C26H, C18H, C19H, C13H & C5H), [2.69 & 2.57 (s, 2H, C15H)], [2.14, 2.09 (d, $^4J_{HH}$ = 2.29 Hz, 2H, C17H)], 1.90-1.66 (m, 12H, C2H&C6H), 1.40-1.29 (m, 32H, C7H, C8H, C9H & C11H), 0.93-0.87 (m, 24H, C10H&C12H).

**$^{13}$C NMR** (176 MHz, $CDCl_3$): $\delta_C$ 166.4-165.2 (C=N), [163.2, 163.1 (C20)], 160.1 (C25), [141.7, 141.6 (C-NO2)], 139.7 (C4), 129.8 (C23), 128.4 (C23), [114.6, 114.5 (C3)], 114.4 (C24), [113.0, 112.8 (C2)], 87.3 (C16), 70.6 (C21), 69.4 (C17), 55.6 (C26), [51.3, 51.2 (C5)], [50.6, 50.5 (C27)], 49.2 (C18), [43.5, 43.3 (C19)], [42.0, 41.9 (C13)], [38.5 & 37.9 (C6)], [31.7, 31.4, 31.0, 29.3, 29.2, 27.6 & 27.5 (C7, C14, C15 & C11)], 24.2 (C8), 23.5 (C9), 14.5 (C10 or C12), 11.1 (C10 or C12)].

**HRMS (ES+):** calculated for $[C_{93}H_{132}N_{20}O_8+H]^+$: 1658.0615, found: 1658.0674 (+3.6 ppm).

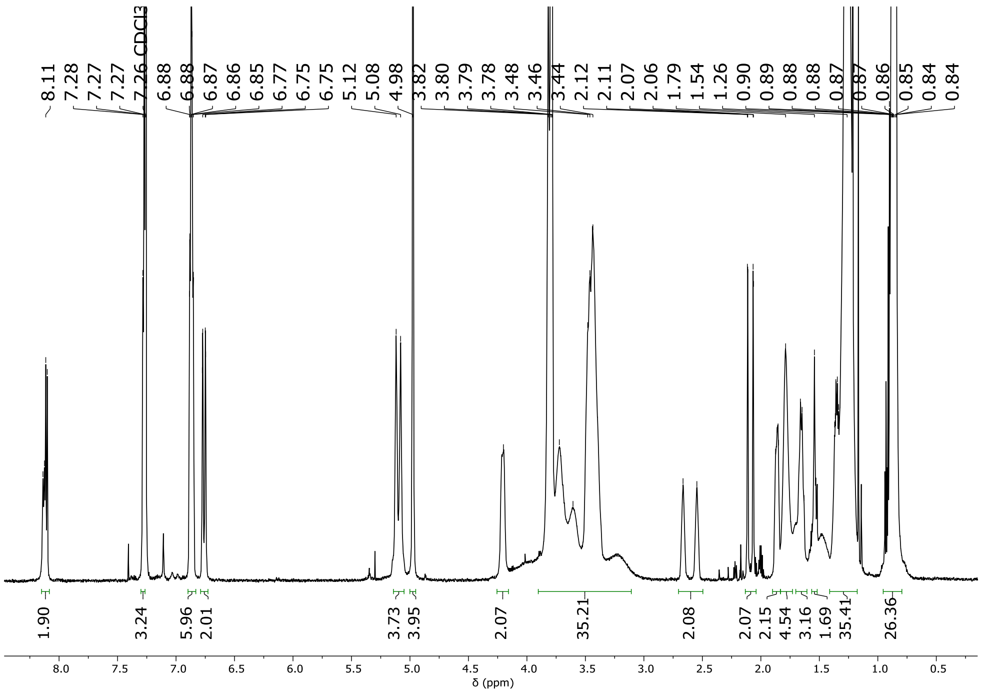


*Figure S 22 $^{1}H$ NMR (500 MHz, $CDCl_3$) spectrum of compound **D*OD***.*

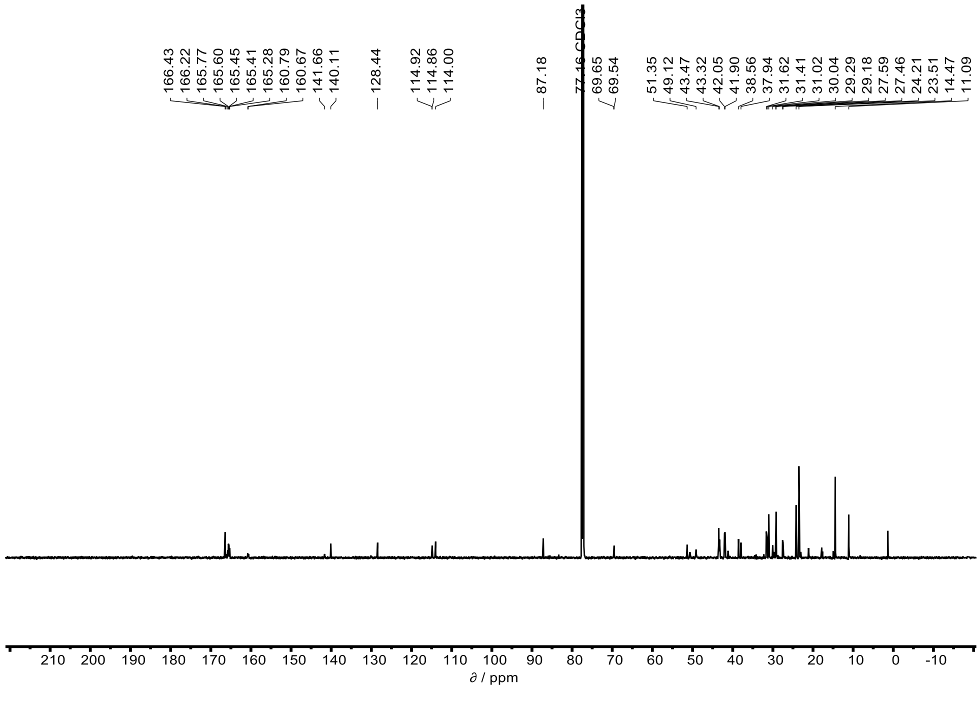


*Figure S 23 $^{13}C$ NMR (126 MHz, $CDCl_3$) spectrum of compound **D*OD***.*

# DOD

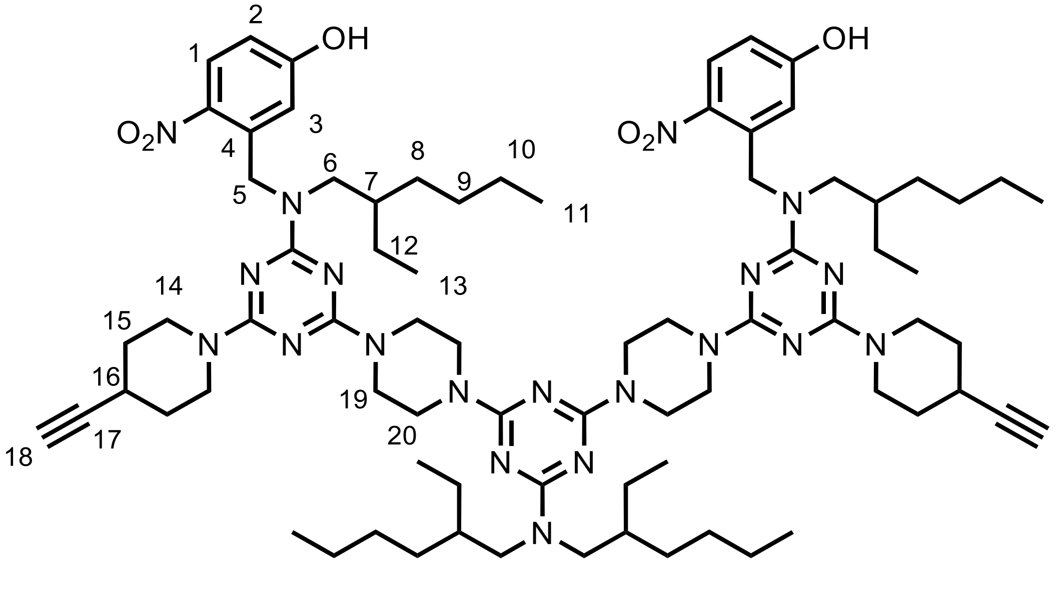


**DOD**

Compound **D*OD*** (70 mg, 0.042 mmol) was dissolved in DCM (0.42 mL) and an equal volume of TFA (0.42 mL) was added and the mixture was left to stir at room temperature for 3h. The solution was flushed with. The crude was purified by column chromatography (silica gel, 0-20% methanol in DCM) to give **DOD** (quantitative yield).

**$^{1}$H NMR** (700 MHz, $CDCl_3$): $\delta_H$ 8.06 (m, 2H, C1H), 6.69-6.58 (m, 4H, C2H&C3H), [5.11, 5.09 (s, 4H, C5H)], 4.19 (m, 2H, C14H), 3.82-3.41 (m, 32H, C6H, C19H, C14H & C20H), [2.64, 2.55 (s, 2H, C16H)], [2.12, 2.06 (s, 2H, C18H)], 1.86-1.33 (m, 8H, C7H & C15H), 1.28-1.21 (m, 36H, C8H, C9H, C10H & C12H), 0.91-0.87 (m, 24H, C11H & C13H).

**$^{13}$C NMR** (176 MHz, $CDCl_3$): $\delta_C$ 166.4 - 165.1 ( C=N), [160.6, 160.5 (C-OH)], 141.3 (C-NO2), 139.9 (C4), 128.3 (C1), [114.7, 114.7 (C3)], 113.8 (C2), 87.0 (C17), [69.5, 69.4 (C18)], 51.2 (C6), 50.5 (C5), 48.9 - 43.1 (C19 & C20)], [41.9, 41.7 (C14)], 38.4, 37.8, 31.4, 31.2- 27.3 (C8, C12, C15 & C16), 24.0 (C9), 23.3 (C10), 14.3 (C11 or C13), 10.9 (C11 or C13) .

**HRMS (ES+):** calculated for $[C_{77}H_{116}N_{20}O_6+H]^+$: 1417.9465, found: 1417.9558 (-0.5 ppm).

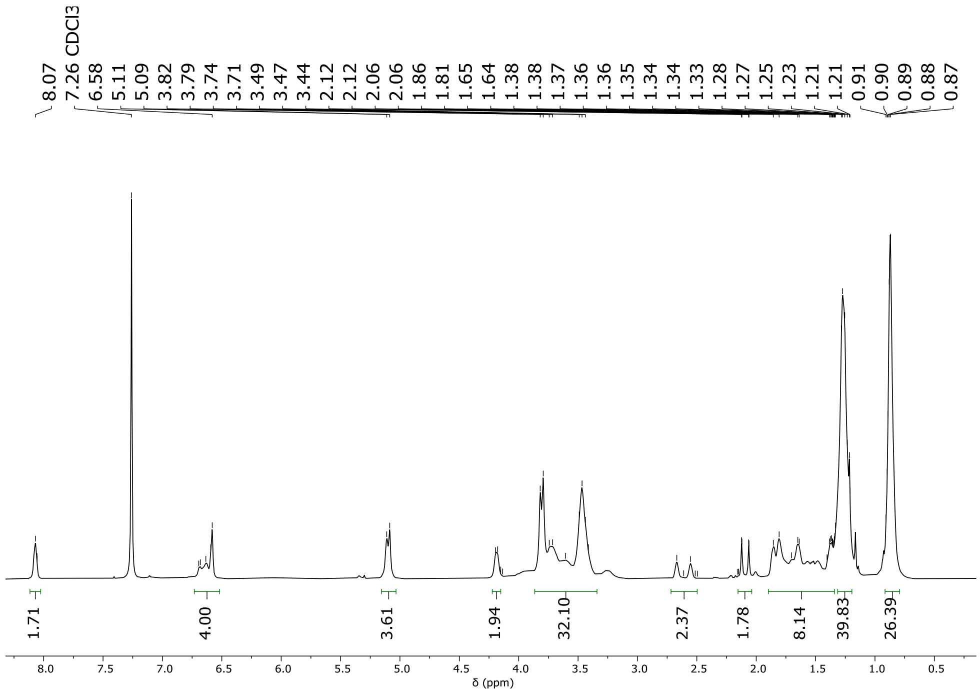

*Figure S 24 ¹H NMR (500 MHz, $CDCl_3$) spectrum of compound **DOD**.*

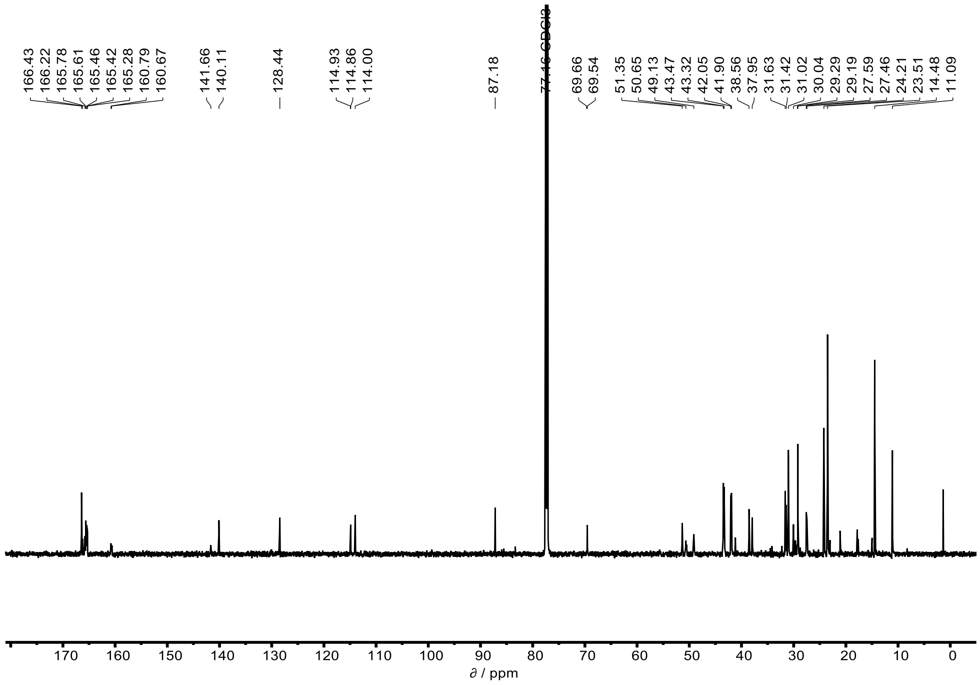

*Figure S 25 ¹³C NMR (126 MHz, $CDCl_3$) spectrum of compound **DOD**.*

# AOA

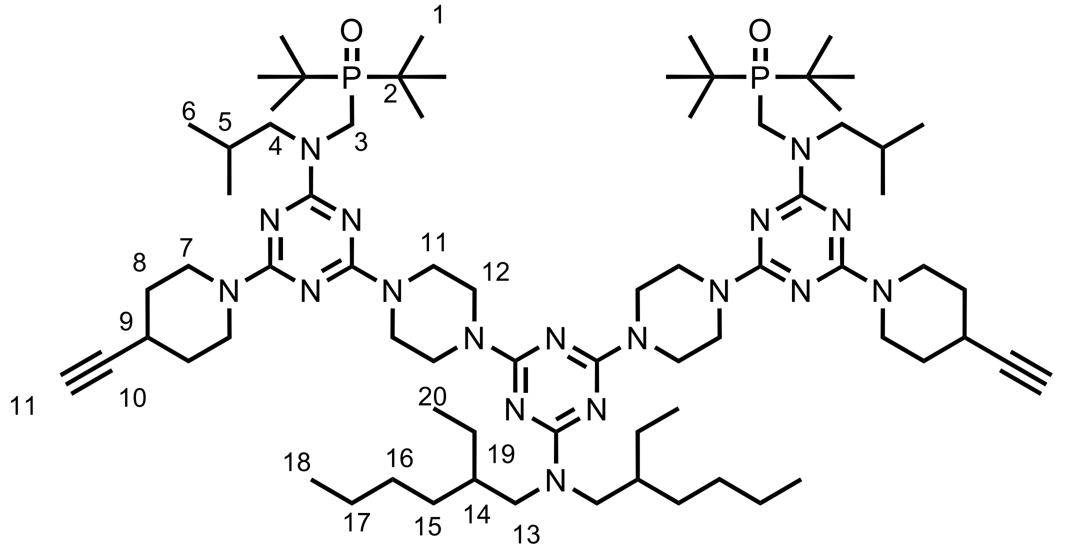


**AOA**

Compound **8** (402 mg, 0.8 mmol, 5.8 eq) in THF (1.2 mL) was added to compound **1** (53 mg, 0.135 mmol, 1 eq.) in a solution of THF (6 mL). DIPEA (0.041 mL, 2 eq.) was added and the solution was left to stir at 60 °C for 67 hours to form a pale-yellow solution. The reaction was monitored by UPLC-MS to ensure that it went to completion. EtOAc (15 mL) was added and the solution was washed with water (3 × 10 mL), dried with $MgSO_4$ and the solvent then removed *in vacuo.* The crude product was purified by column chromatography (silica gel, 0-30% MeOH in DCM) to give **AOA** (178 mg, 0.131 mmol, 97%).

**$^1$H NMR** (700 MHz, $CDCl_3$) $\delta_H$ 4.39 (s, 4H, C3H), 4.15 (m, 4H, C7H), 3.84 (d, $^3J_{HH}$ = 7.2 Hz, 4H, C4H), 3.78 (s, 16H, C11H & C12H), 3.43 (m, 8H, C13H & C7H), 2.65 (s, 2H, C9H), 2.21 (m, 2H, C5H), 2.11 (s, 2H, C11H), 1.83 (m, 6H, C14 & C8), 1.64-1.60 (m, 4H, C4H), 1.26 (m, 52H, C1H, C15H, C16H, C17H, C19H), 0.87 (m, 24H, C6H&C18H).

**$^{13}$C NMR** (176 MHz, $CDCl_3$) $\delta_C$ 166.2 - 165.1 (C=N & C-OH), 87.0 (C10), 69.7 (C11), 53.3 (C4 or C13), 50.9 (C4 or C13), 43.4 (C11 & C12), 41.9 (C7), 38.4 (d, $^1J_{PC}$ = 59.4, C3), 38.0 (C14), 36.1 (d, $^1J_{PC}$ = 55.4, C2), 31.6 (C8 or C14), 31.01 (C8 or C14), 29.3 (C9 or C15 or C19), 27.6 (C9 or C15 or C19), 27.1 (C9 or C15 or C19), 26.8 (C1), 24.2 (C16), 23.5 (C17), 20.9 (C6), 14.5 (C18 or C20), 11.2 (C18 or C20).

**$^{31}$P NMR** (283 MHz, $CDCl_3$): $\delta_P$ 58.5.

**HRMS (ES$^+$):** calculated for $[C_{73}H_{128}N_{18}O_2P_2+H]^+$: 1352.0021, found: 1352.0035 (+1.0 ppm).

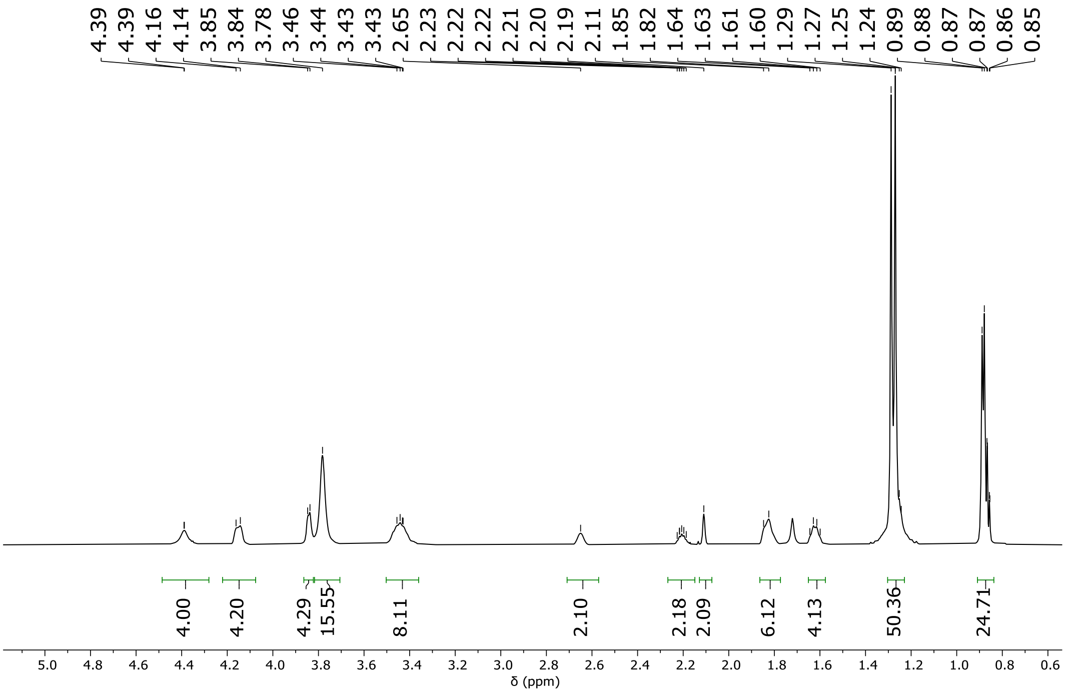


*Figure S 26 $^{1}H$ NMR (500 MHz, $CDCl_3$) spectrum of compound **AOA**.*

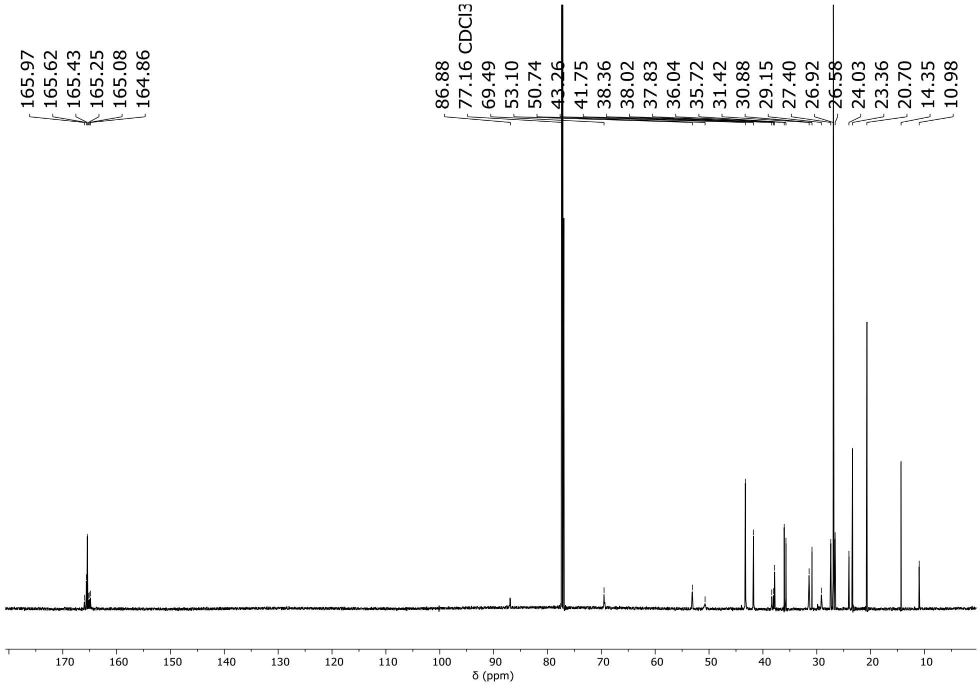


*Figure S 27 $^{13}C$ NMR (126 MHz, $CDCl_3$) spectrum of compound **AOA**.*

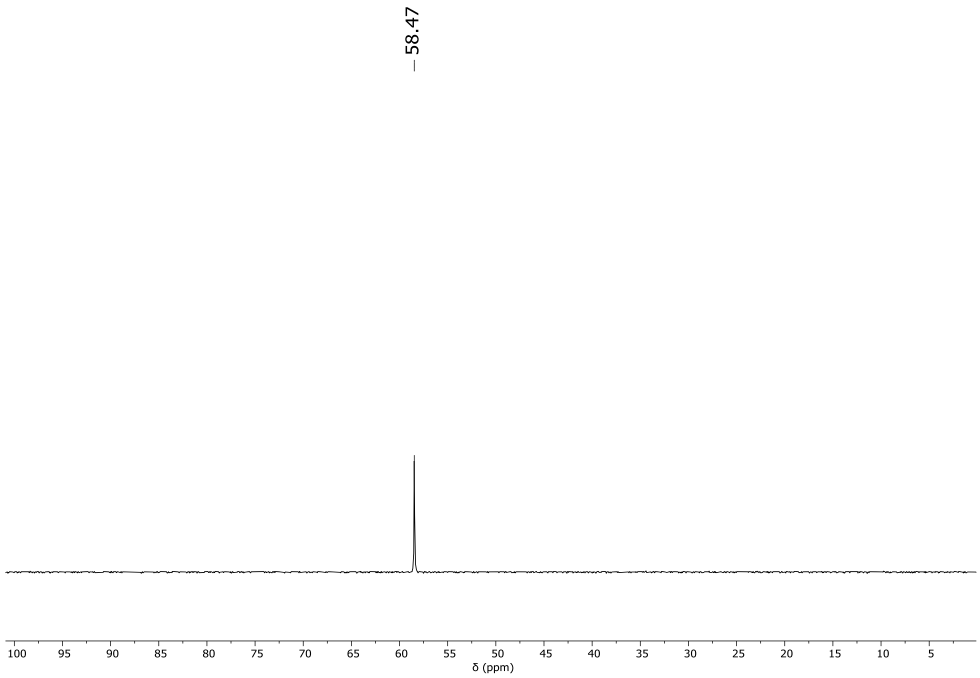


*Figure S 28 $^{31}$P NMR (162 MHz, $CDCl_3$) spectrum of compound* ***AOA***.

# General procedures for SPS and oligomers

The first step of every run is the *Fmoc deprotection*: the loaded Wang resin was agitated in the piperazine DMF solution (7 mL, 0.7 M, 2 x 10 min). The deprotection solution was then drained and the resin was washed with DMF (4 x 5 mL). The first substitution and the oligomer elongation were carried out by *coupling cycle*: the resin-bound oligomer was first agitated in a solution of **1, 2, 6, and 7** (0.1 M, 10 eq.) and DIPEA (0.1 M, 10 eq.) in DMF (5 mL) for 15 mins at 90 °C. This coupling solution was drained, and the resin was washed with DMF (4 x 5 mL). The resin-bound oligomer was then agitated in a solution of piperazine (5 mL, 0.7 M) in DMF for 15 min at 90 °C. The second coupling solution was drained, and the resin was washed with DMF (4 x 5 mL). The last coupling cycle was carried out without the piperazine addition, so that the oligomer is taken off the Liberty Blue synthesiser as a mono-chloro compound and washed with DMF (5 x 10 mL) and DCM (10 x 10 mL). In the *manual capping*, the loaded resin was then swollen in NMP (5 mL), DIPEA (0.05 mL) and 10 equivalents of capping amine (**8** and **9**) and then reacted in microwave reactor at 90 °C for 20 minutes. The resin was then washed with DMF (5 x 10 mL) and DCM (10 x 10 mL). The *cleavage* of the resin doubles as PMB deprotection for the nitrophenol recognition unit and was carried out by agitating the resin in a 9:0.5:0:5 solution of TFA:TIS:DCM (10mL) overnight. The resulting solution as concentrated under nitrogen, washed with water (2 x 20 mL), dried over anhydrous magnesium sulfate and purified by flash column chromatography ($SiO_2$, 0-20% MeOH in DCM).

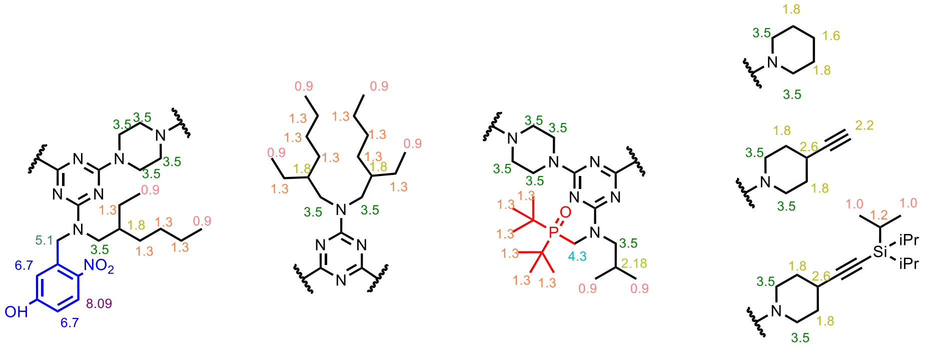

*Figure S 29 Guide of $\delta_H$ (in ppm) used for the $^1H$ NMR spectra assignment of REMOs made by automated SPS.*

# AOD

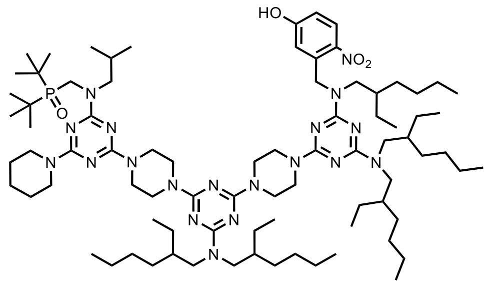


**$^{1}$H NMR** (400 MHz, $CDCl_3$) $\delta_H$ 8.08 (m, 1H), 7.10 (m, 1H), 6.65 (m, 1H), 5.10 (m, 2H), 4.45 (m, 2H), 3.50 (m, 32H), 2.20 (m, 1H), 1.85 - 1.65 (m, 11H), 1.25 (m, 58H), 0.90 (m, 36H).
**$^{31}$P NMR** (203 MHz, $CDCl_3$) $\delta_p$ 62.3.
**HRMS (ES+):** calcd. for $[C_{82}H_{146}N_{19}O_4P+H]^+$ is 1493.1616, found 1493.1635 (1.30 ppm)
**UPLC trace** *UPLC Conditions*: ACQUITY UPLC BEH C4 Column, 300Å, 1.7 µm, 2.1 mm X 50 mm was used as the UPLC column. The conditions of the UPLC method are as follows: Solvent A: Water +0.1% Formic acid; Solvent B: Tetrahydrofuran +0.1% Formic acid; Gradient of 0-4 minutes 30% - 100%B + 2 minute 100% B with re-equilibration time of 2 minutes. Flow rate: 0.4 ml/min; Column temperature of 40 °C; Injection volume of 2 µL. The signal was monitored at 254 nm.
**ESI-MS** Calculated Mass: 747.3 $[M+H]^+$, 1493.1 $[M+2H]^{2+}$; Mass found (ESI+): 1494.5 $[M+H]^+$, 747.5 $[M+2H]^{2+}$.

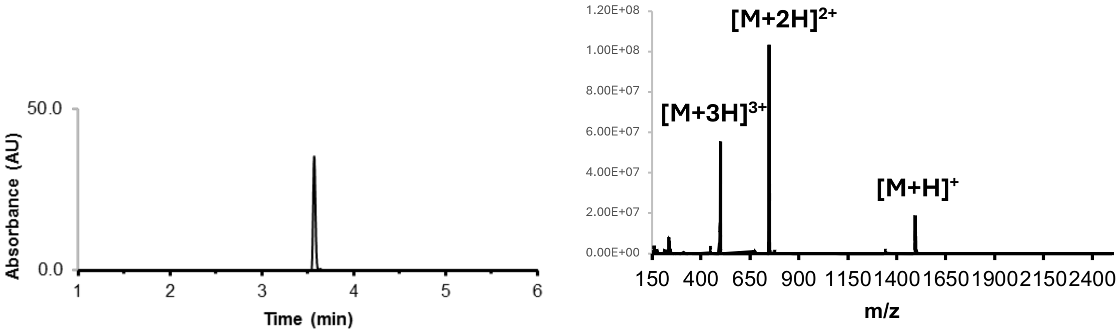


*Figure S 30 UPLC trace of **AOD** (UPLC Conditions: ACQUITY UPLC BEH C4 Column, 300Å, 1.7 µm, 2.1 mm X 50 mm was used as the UPLC column. The conditions of the UPLC method are as follows: Solvent A: Water +0.1% Formic acid; Solvent B: Tetrahydrofuran +0.1% Formic acid; Gradient of 0-4 minutes 30% - 100%B + 2 minute 100% B with re-equilibration time of 2 minutes. Flow rate: 0.4 ml/min; Column temperature of 40 °C; Injection volume of 2 µL. The signal was monitored at 254 nm) and ESI-MS.*

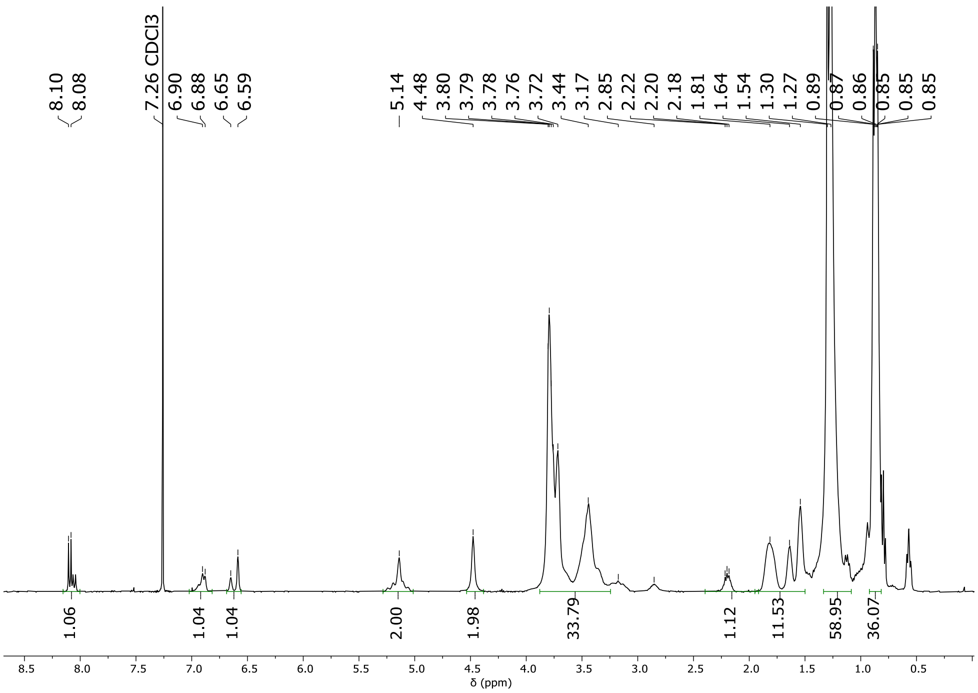


*Figure S 31 $^{1}$H NMR (400 MHz, CDCl$_3$) spectrum of compound **AOD**.*

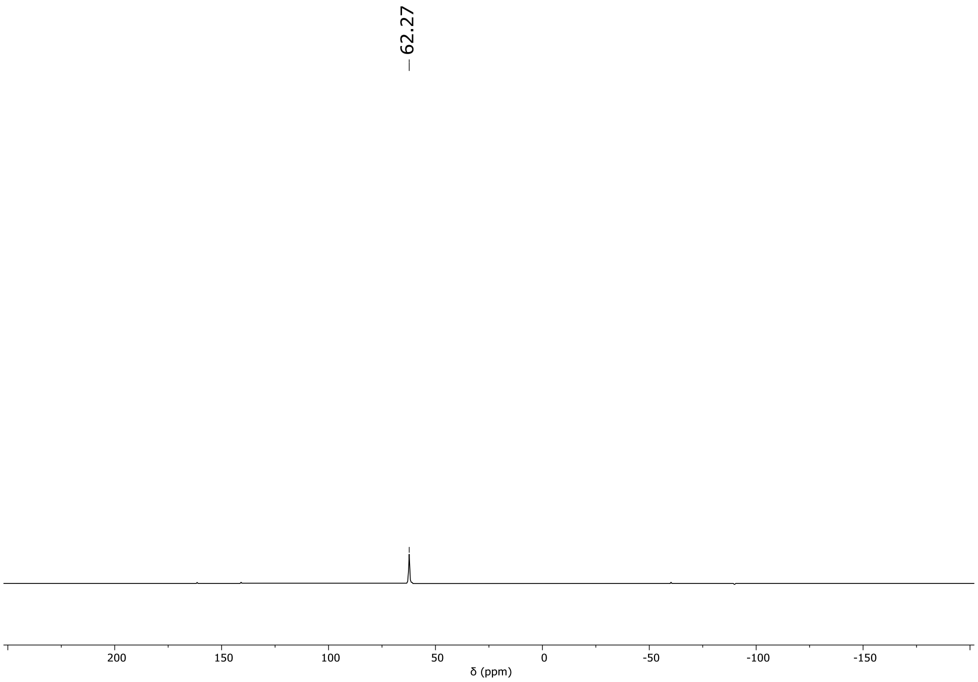


*Figure S 32 $^{31}$P NMR (203 MHz, CDCl$_3$) spectrum of compound **AOD**.*

# AOOD

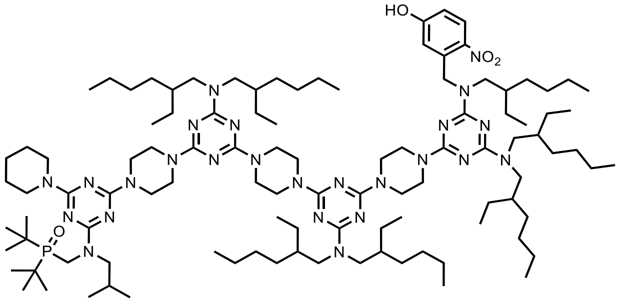

**$^1$H NMR** (400 MHz, $CDCl_3$) $\delta_H$ 8.08 (m, 1H), 7.10 (m, 1H), 6.65 (m, 1H), 5.10 (m, 2H), 4.45 (m, 2H), 3.50 (m, 44H), 2.20 (m, 1H), 1.85 - 1.65 (m, 13H), 1.25 (m, 74H), 0.90 (m, 48H).
**$^{31}$P NMR** (162 MHz, $CDCl_3$) $\delta_p$ 62.3.
**HRMS (ES+):** calcd. for $[C_{105}H_{188}N_{25}O_4P+H]^+$ is 1895.5110, found 1895.5087 (1.22 ppm)
**UPLC trace** *UPLC Conditions*: The conditions of the UPLC method are as follows: Solvent A: Water +0.1% Formic acid; Solvent B: Tetrahydrofuran +0.1% Formic acid; Gradient of 0-4 minutes 30% - 100%B + 2 minute 100% B with re-equilibration time of 2 minutes. Flow rate: 0.4 ml/min; Column temperature of 40 °C; Injection volume of 2 µL. The signal was monitored at 254 nm.
**ESI-MS** Calculated Mass: 1896.3 $[M+H]^+$, 948.9 $[M+2H]^{2+}$, 632.5 $[M+3H]^{3+}$ Mass found (ESI+): 1896.5 $[M+H]^+$, 948.9 $[M+2H]^{2+}$, 632.5 $[M+3H]^{3+}$.

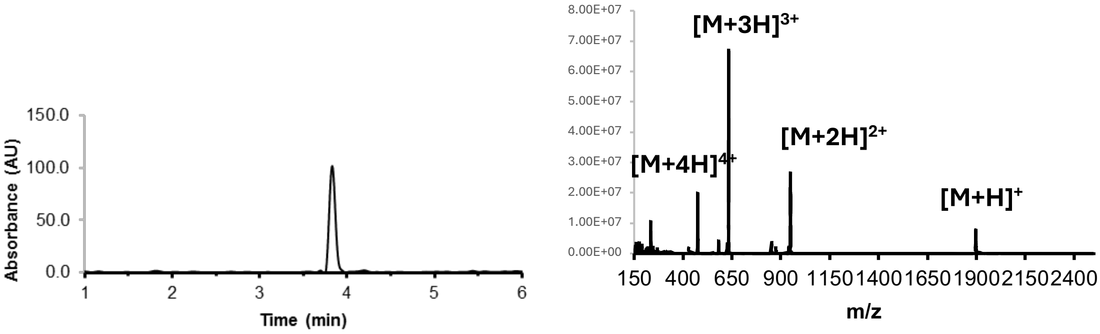


*Figure S 33 UPLC trace of **AOOD** (UPLC Conditions: The conditions of the UPLC method are as follows: Solvent A: Water +0.1% Formic acid; Solvent B: Tetrahydrofuran +0.1% Formic acid; Gradient of 0-4 minutes 30% - 100%B + 2 minute 100% B with re-equilibration time of 2 minutes. Flow rate: 0.4 ml/min; Column temperature of 40 °C; Injection volume of 2 µL. The signal was monitored at 254 nm) and ESI-MS.*

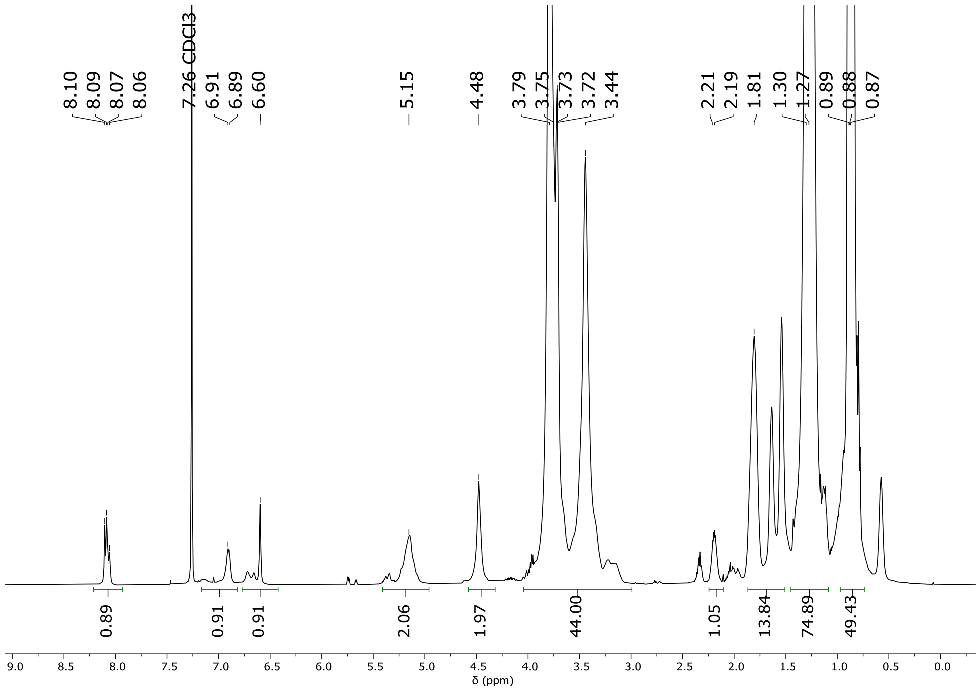

*Figure S 34 $^{1}H$ NMR (400 MHz, $CDCl_3$) spectrum of compound **AOOD**.*

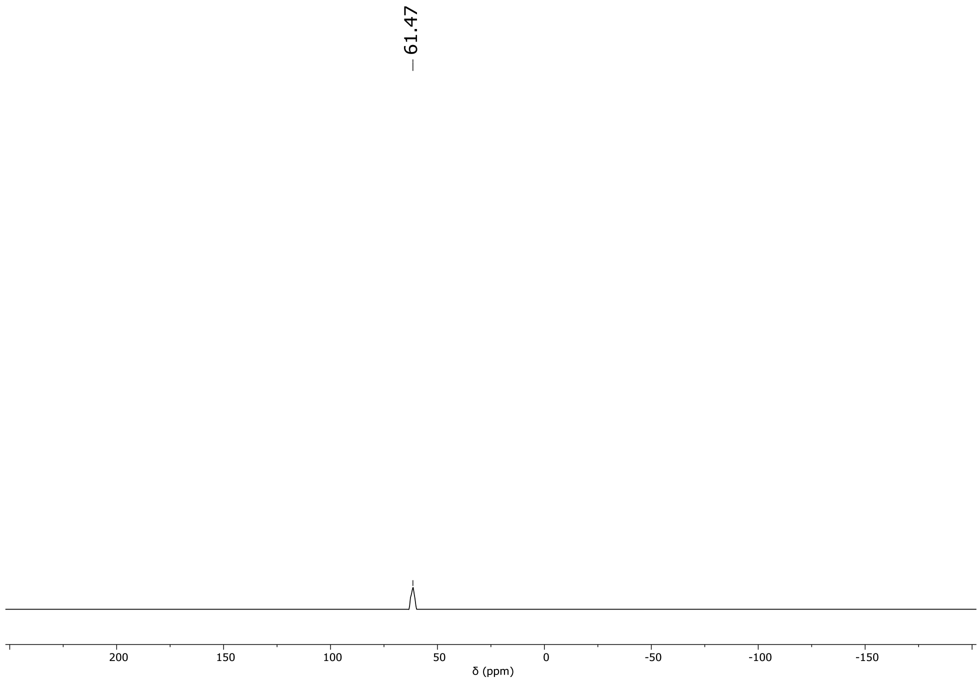

*Figure S 35 $^{31}P$ NMR (162 MHz, $CDCl_3$) spectrum of compound **AOOD**.*

# AOOOD

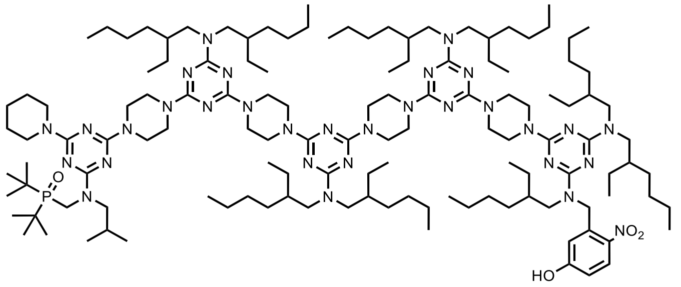

**$^{1}$H NMR** (400 MHz, CDCl$_3$) $\delta_H$ 8.08 (m, 1H), 7.10 (m, 1H), 6.65 (m, 1H), 5.10 (m, 2H), 4.45 (m, 2H), 3.50 (m, 56H), 2.20 (m, 1H), 1.85 - 1.65 (m, 15H), 1.25 (m, 90H), 0.90 (m, 60H).
**$^{31}$P NMR** (162 MHz, CDCl$_3$) $\delta_p$ 62.48.
**HRMS (ES+):** calcd. for $[C_{128}H_{231}N_{31}O_4P+H]^+$ is 2297.8610, found 2297.8557 (2.27 ppm)
**UPLC trace** *UPLC Conditions*: The conditions of the UPLC method are as follows: Solvent A: Water +0.1% Formic acid; Solvent B: Tetrahydrofuran +0.1% Formic acid; Gradient of 0-4 minutes 30% - 100%B + 2 minute 100% B with re-equilibration time of 2 minutes. Flow rate: 0.4 ml/min; Column temperature of 40 °C; Injection volume of 2 µL. The signal was monitored at 254 nm.
**ESI-MS** Calculated Mass: 2300.5 $[M+H]^+$, 1149.9 $[M+2H]^{2+}$, 766.9 $[M+3H]^{3+}$ Mass found (ESI+): 2300.5 $[M+H]^+$, 1149.9 $[M+2H]^{2+}$, 767.0 $[M+3H]^{3+}$.

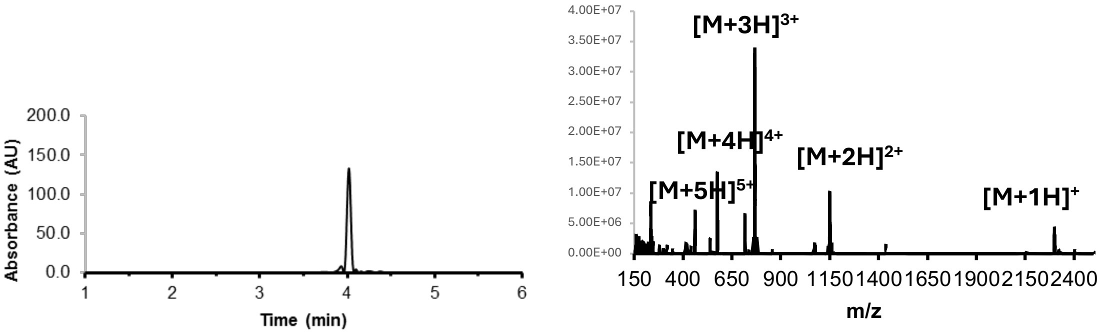


*Figure S 36 UPLC trace of **AOOOD** (UPLC Conditions: The conditions of the UPLC method are as follows: Solvent A: Water +0.1% Formic acid; Solvent B: Tetrahydrofuran +0.1% Formic acid; Gradient of 0-4 minutes 30% - 100%B + 2 minute 100% B with re-equilibration time of 2 minutes. Flow rate: 0.4 ml/min; Column temperature of 40 °C; Injection volume of 2 µL. The signal was monitored at 254 nm) and ESI-MS.*

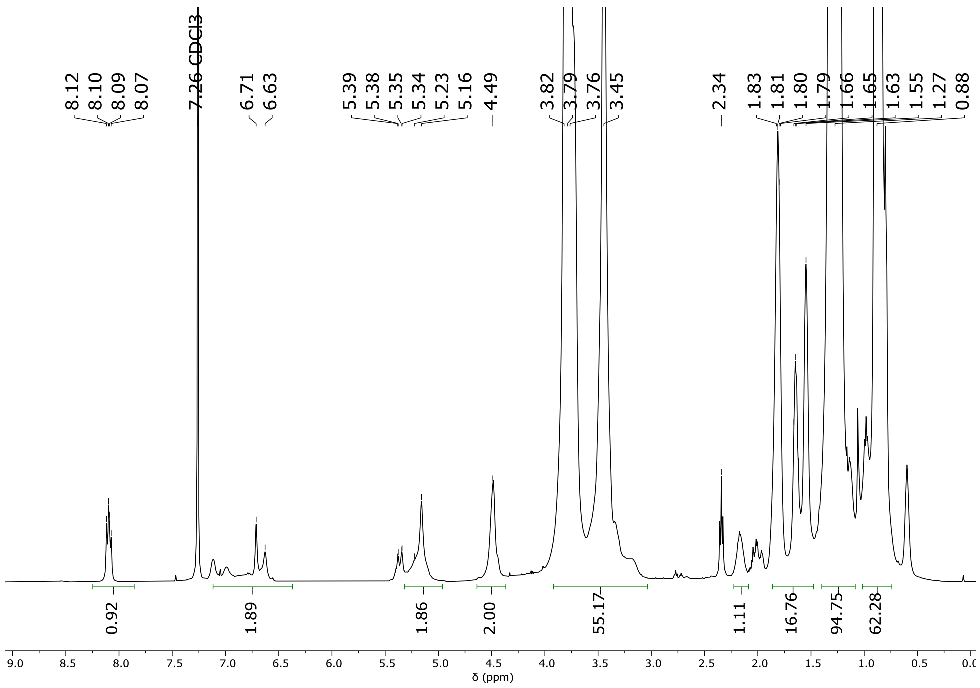


*Figure S 37 $^{1}H$ NMR (400 MHz, $CDCl_3$) spectrum of compound **AOOOD**.*

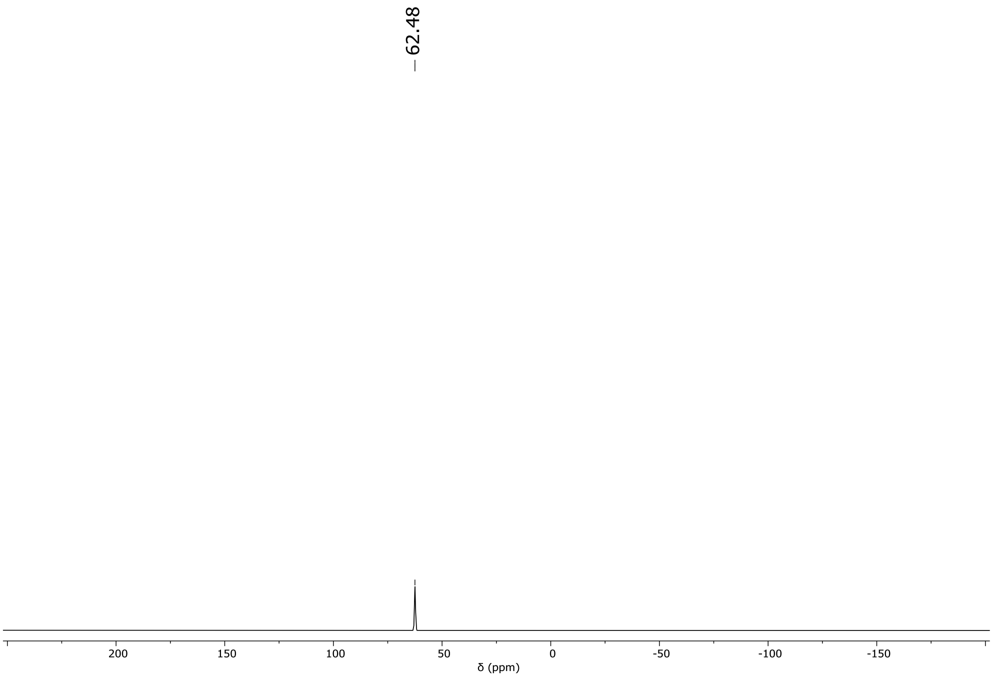


*Figure S 38 $^{31}P$ NMR (162 MHz, $CDCl_3$) spectrum of compound **AOOOD**.*

# AOOOOD

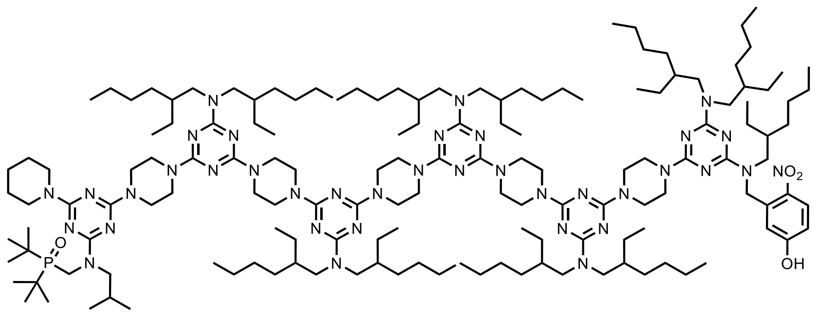


**$^1$H NMR** (400 MHz, $CDCl_3$) $\delta_H$ 8.08 (m, 1H), 7.10 (m, 1H), 6.65 (m, 1H), 5.10 (m, 2H), 4.45 (m, 2H), 3.50 (m, 68H), 2.20 (m, 1H), 1.85 - 1.65 (m, 17H), 1.25 (m, 106H), 0.90 (m, 72H).
**$^{31}$P NMR** (162 MHz, $CDCl_3$) $\delta_p$ 63.5, 62.4.
**HRMS (ES+):** calcd. for $[C_{151}H_{272}N_{37}O_4P+H]^+$ is 2700.2117, found 2700.2034 (3.10 ppm)
**UPLC trace** *UPLC Conditions*: The conditions of the UPLC method are as follows: Solvent A: Water +0.1% Formic acid; Solvent B: Tetrahydrofuran +0.1% Formic acid; Gradient of 0-4 minutes 30% - 100%B + 2 minute 100% B with re-equilibration time of 2 minutes. Flow rate: 0.4 ml/min; Column temperature of 40 °C; Injection volume of 2 µL. The signal was monitored at 254 nm.
**ESI-MS** Calculated Mass: 1552.9 $[M+2H]^{2+}$, 1035.5 $[M+3H]^{3+}$, 776.7 $[M+4H]^{4+\cdot}$ Mass found (ESI+): 1552.9 $[M+2H]^{2+}$, 1003.5 $[M+3H]^{3+}$,776.6$[M+4H]^{4+\cdot}$

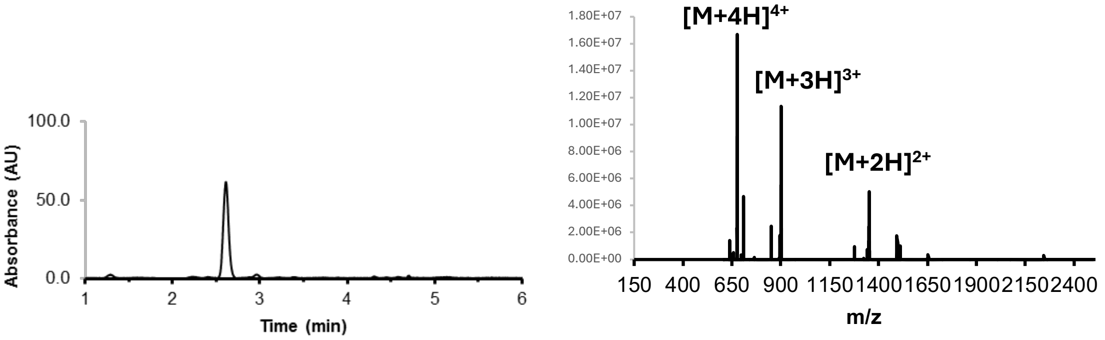


*Figure S 39 UPLC trace of **AOOOOD** (UPLC Conditions: The conditions of the UPLC method are as follows: Solvent A: Water +0.1% Formic acid; Solvent B: Tetrahydrofuran +0.1% Formic acid; Gradient of 0-4 minutes 30% - 100%B + 2 minute 100% B with re-equilibration time of 2 minutes. Flow rate: 0.4 ml/min; Column temperature of 40 °C; Injection volume of 2 µL. The signal was monitored at 254 nm) and ESI-MS.*

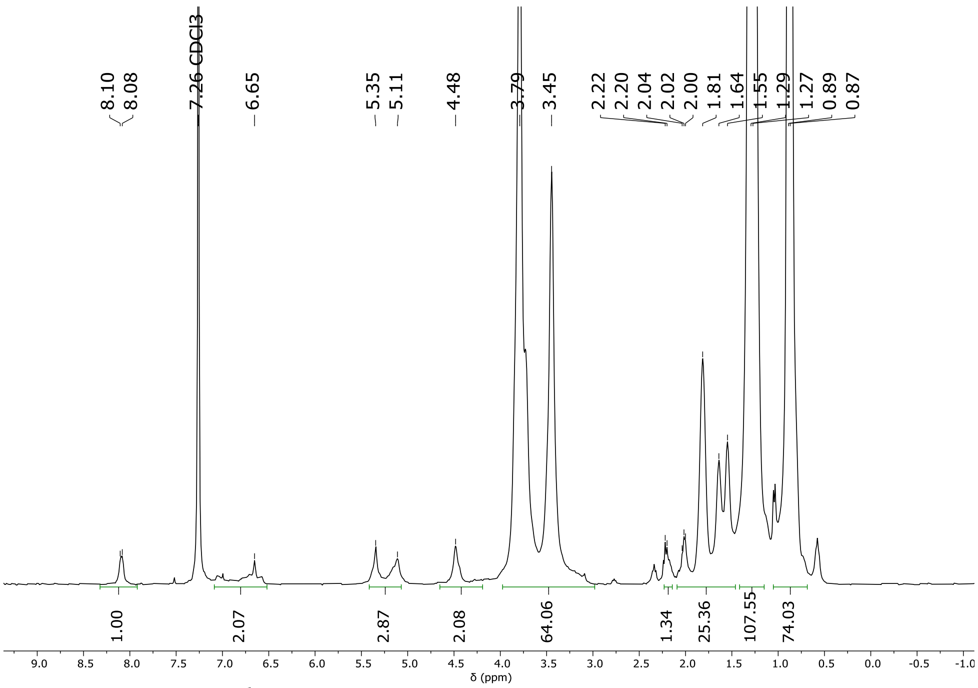


*Figure S 40 $^{1}H$ NMR (400 MHz, $CDCl_3$) spectrum of compound **AOOOOD**.*

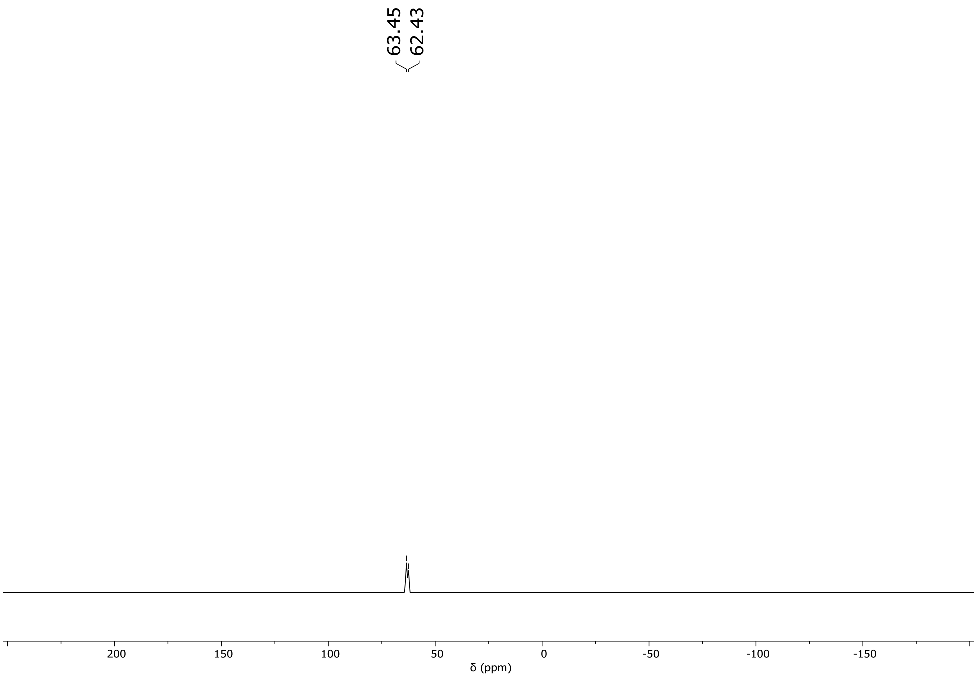


*Figure S 41 $^{31}P$ NMR (162 MHz, $CDCl_3$) spectrum of compound **AOOOOD**.*

# $AO_5D$

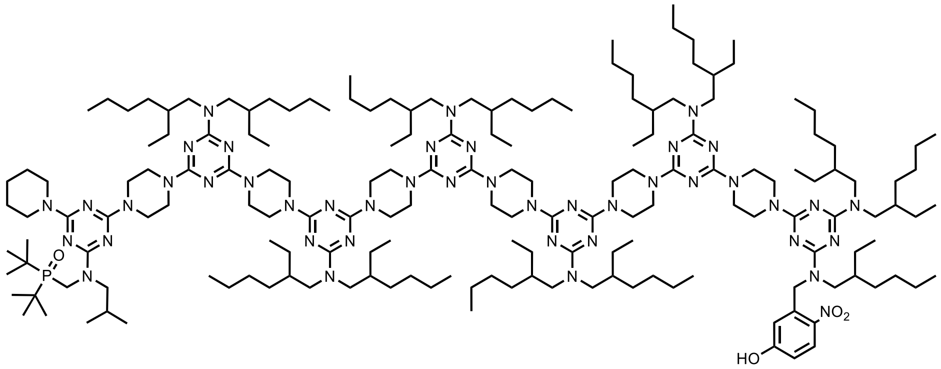

**¹H NMR** (400 MHz, $CDCl_3$) $\delta_H$ 8.08 (m, 1H), 7.10 (m, 1H), 6.65 (m, 1H), 5.10 (m, 2H), 4.45 (m, 2H), 3.50 (m, 80H), 2.20 (m, 1H), 1.85 - 1.65 (m, 19H), 1.25 (m, 122H), 0.90 (m, 84H).
**³¹P NMR** (162 MHz, $CDCl_3$) $\delta_p$ 61.59.
**HRMS (ES+):** calcd. for $[C_{174}H_{315}N_{43}O_4P+H]^+$ is 3201.5591, found 3102.5505 (2.80 ppm)
**UPLC trace** *UPLC Conditions*: The conditions of the UPLC method are as follows: Solvent A: Water +0.1% Formic acid; Solvent B: Tetrahydrofuran +0.1% Formic acid; Gradient of 0-4 minutes 30% - 100%B + 2 minute 100% B with re-equilibration time of 2 minutes. Flow rate: 0.4 ml/min; Column temperature of 40 °C; Injection volume of 2 µL. The signal was monitored at 254 nm.
**ESI-MS** Calculated Mass: 1552.9 [M+2H]2+, 1035.5 $[M+3H]^{3+}$, 776.6 $[M+4H]^{4+}$. Mass found (ESI+): 1552.9 [M+2H]2+, 1035.5 $[M+3H]^{3+}$, 776.6 $[M+4H]^{4+}$.

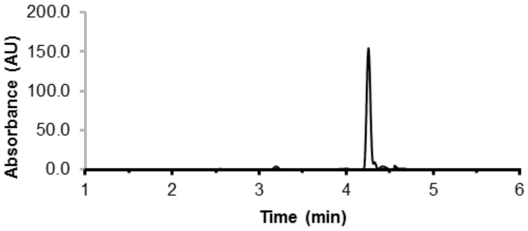


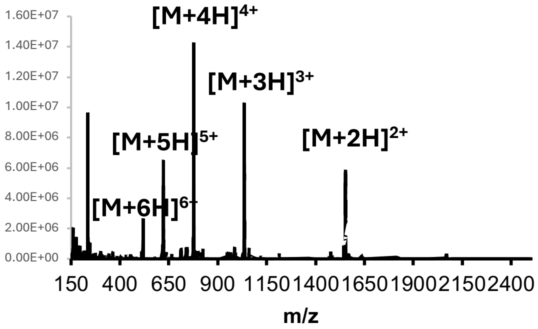


*Figure S 42 UPLC trace of **$AO_5D$** (UPLC Conditions: The conditions of the UPLC method are as follows: Solvent A: Water +0.1% Formic acid; Solvent B: Tetrahydrofuran +0.1% Formic acid; Gradient of 0-4 minutes 30% - 100%B + 2 minute 100% B with re-equilibration time of 2 minutes. Flow rate: 0.4 ml/min; Column temperature of 40 °C; Injection volume of 2 µL. The signal was monitored at 254 nm) and ESI-MS.*

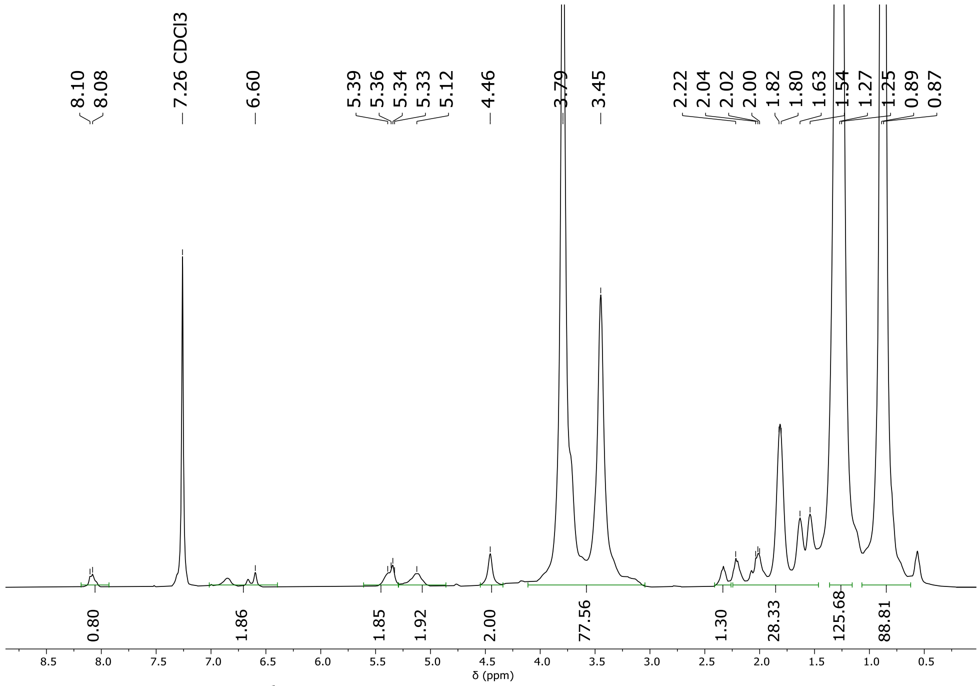


*Figure S 43 $^{1}$H NMR (400 MHz, $CDCl_3$) spectrum of compound* ***$AO_5D$***.

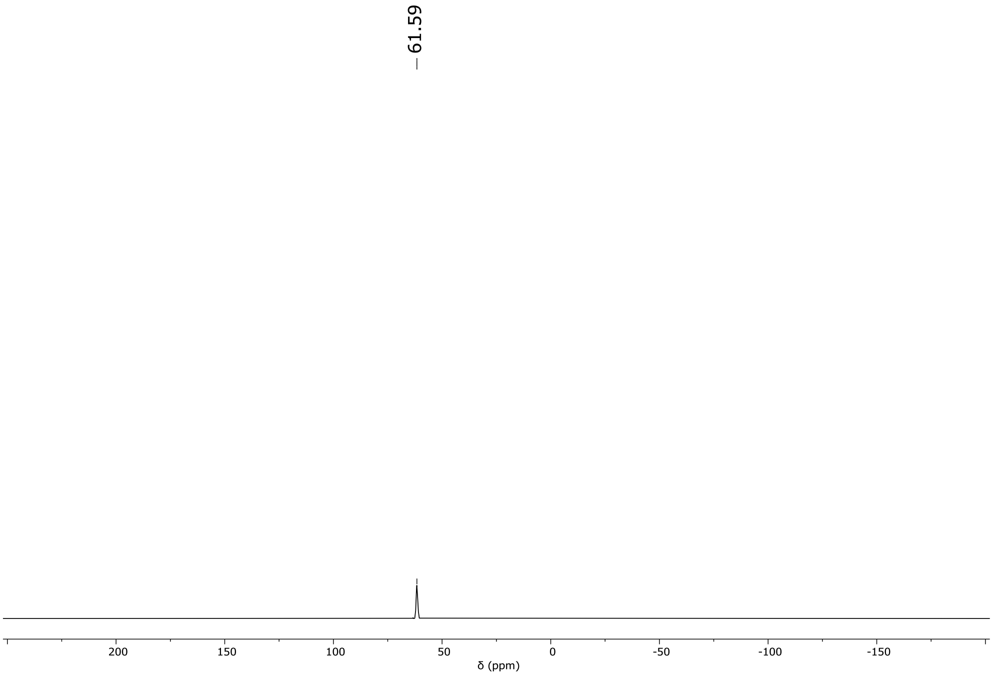


*Figure S 44 $^{31}$P NMR (162 MHz, $CDCl_3$) spectrum of compound* ***$AO_5D$***.

# AO$_6$D

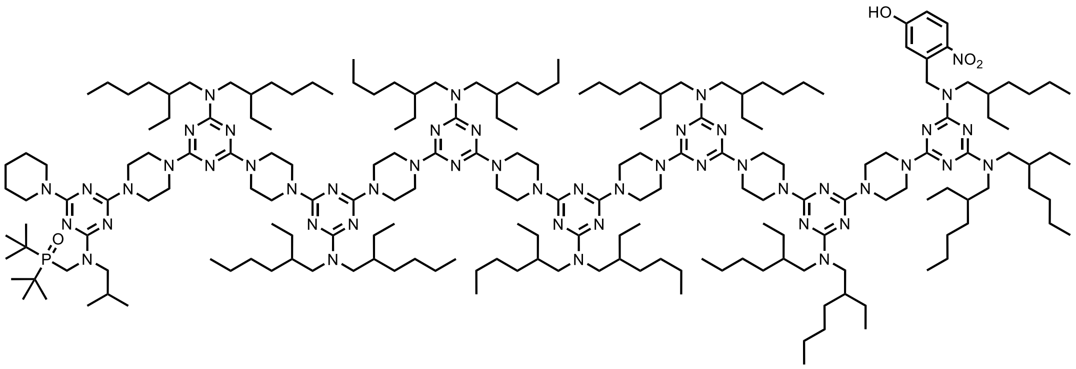

**$^1$H NMR** (400 MHz, CDCl$_3$) $\delta_H$ 8.08 (m, 1H), 7.10 (m, 1H), 6.65 (m, 1H), 5.10 (m, 2H), 4.45 (m, 2H), 3.50 (m, 92H), 2.20 (m, 1H), 1.85 - 1.65 (m, 21H), 1.25 (m, 138H), 0.90 (m, 96H).
**$^{31}$P NMR** (162 MHz, CDCl$_3$) $\delta_p$ 61.45.
**HRMS (ES+):** calcd. for $[C_{197}H_{356}N_{39}O_4P+H]^+$ is 3504.7979, found 3504,8976 (-28.4 ppm)
**UPLC trace** *UPLC Conditions*: The conditions of the UPLC method are as follows: Solvent A: Water +0.1% Formic acid; Solvent B: Tetrahydrofuran +0.1% Formic acid; Gradient of 0-4 minutes 30% - 100%B + 2 minute 100% B with re-equilibration time of 2 minutes. Flow rate: 0.4 ml/min; Column temperature of 40 °C; Injection volume of 2 μL. The signal was monitored at 254 nm.
**ESI-MS** Calculated Mass: 1753.1 $[M+2H]^{2+}$, 1168.2 $[M+3H]^{3+}$, 878.8 $[M+4H]^{4+}$. Mass found (ESI+): 1753.1 $[M+2H]^{2+}$, 1168.5 $[M+3H]^{3+}$, 878.8 $[M+4H]^{4+}$.

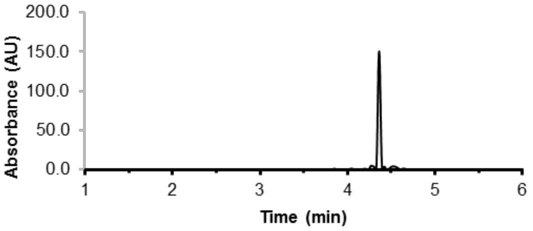


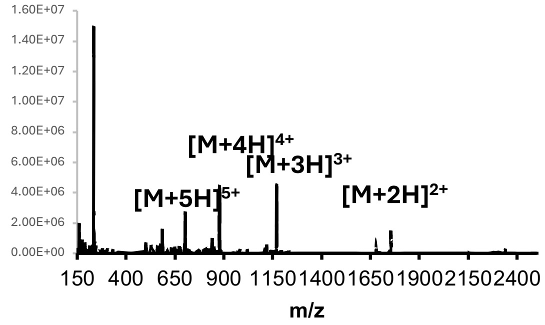


*Figure S 45 UPLC trace of **AO$_6$D** (UPLC Conditions: The conditions of the UPLC method are as follows: Solvent A: Water +0.1% Formic acid; Solvent B: Tetrahydrofuran +0.1% Formic acid; Gradient of 0-4 minutes 30% - 100%B + 2 minute 100% B with re-equilibration time of 2 minutes. Flow rate: 0.4 ml/min; Column temperature of 40 °C; Injection volume of 2 μL. The signal was monitored at 254 nm) and ESI-MS.*

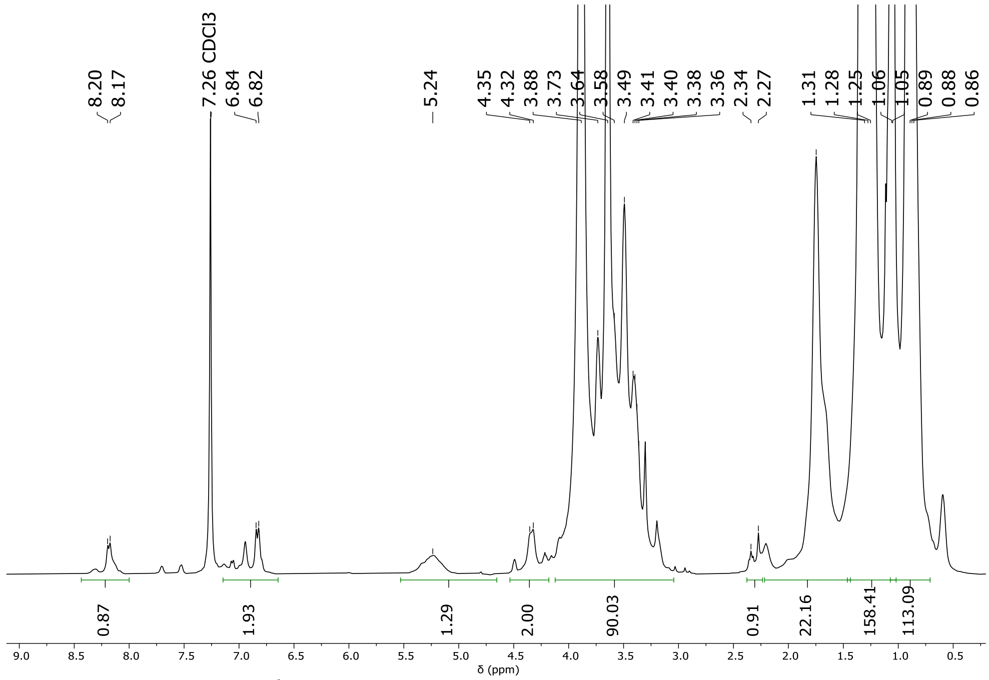


*Figure S 46 $^{1}$H NMR (400 MHz, $CDCl_3$) spectrum of compound* ***$AO_6D$***.

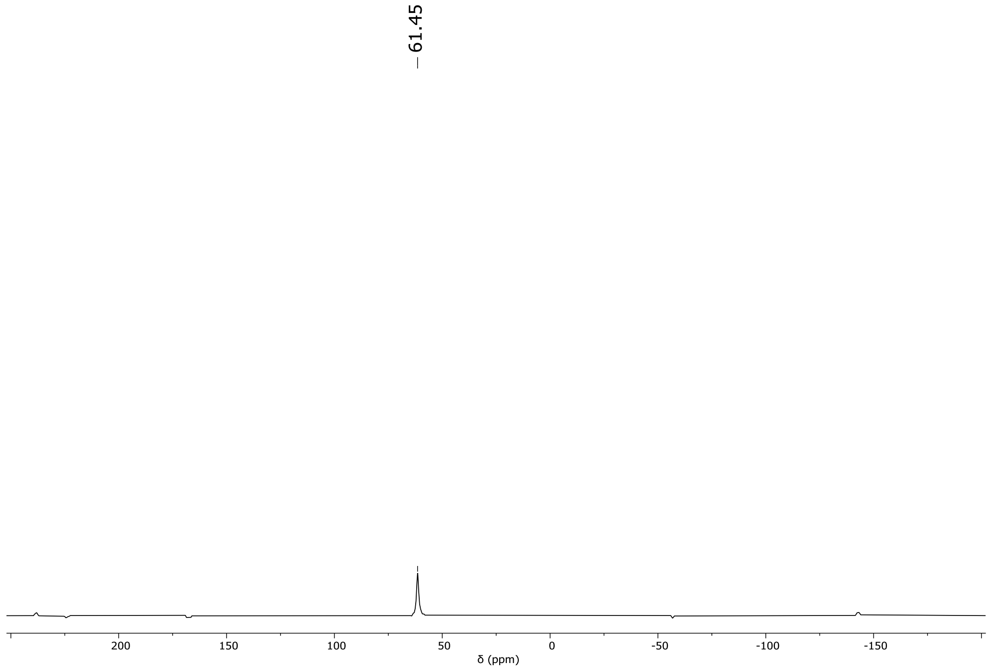


*Figure S 47 $^{31}$P NMR (162 MHz, $CDCl_3$) spectrum of compound* ***$AO_6D$***.

## AO$_7$D

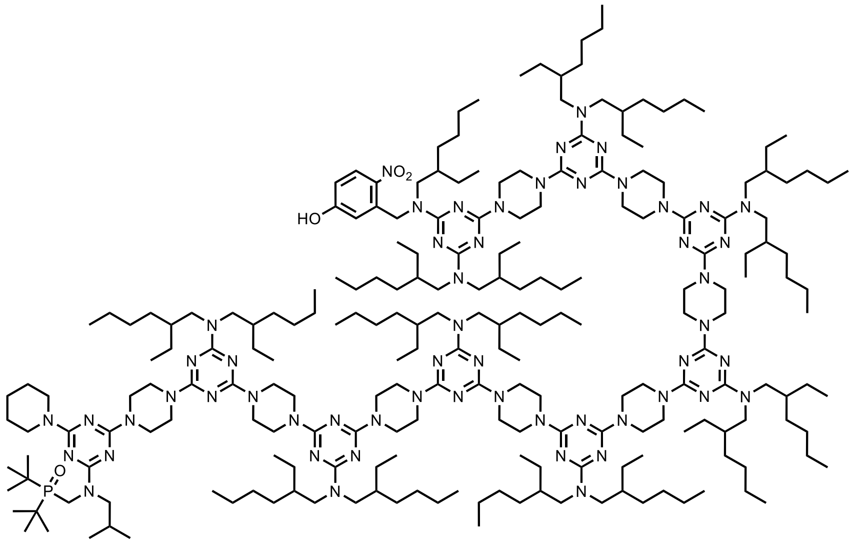

**$^1$H NMR** (400 MHz, $CDCl_3$) $\delta_H$ 8.08 (m, 1H), 7.10 (m, 1H), 6.65 (m, 1H), 5.10 (m, 2H), 4.45 (m, 2H), 3.50 (m, 104H), 2.20 (m, 1H), 1.85 - 1.65 (m, 23H), 1.25 (m, 154H), 0.90 (m, 108H).
**$^{31}$P NMR** (203 MHz, $CDCl_3$) $\delta_p$ 64.40.
**HRMS (ES+):** calcd. for $[C_{222}H_{398}N_{52}O_4P+H]^+$ is 1298.0883, found 1298.9813 (2.00 ppm)
**UPLC trace** *UPLC Conditions*: The conditions of the UPLC method are as follows: Solvent A: Water +0.1% Formic acid; Solvent B: Tetrahydrofuran +0.1% Formic acid; Gradient of 0-4 minutes 30% - 100%B + 2 minute 100% B with re-equilibration time of 2 minutes. Flow rate: 0.4 ml/min; Column temperature of 40 °C; Injection volume of 2 µL. The signal was monitored at 254 nm.
**ESI-MS** Calculated Mass: 1957.3 $[M+2H]^{2+}$, 1304.6 $[M+3H]^{3+}$, 977.9 $[M+4H]^{4+}$, 782.9 $[M+5H]^{5+\cdot}$ and found (ESI+): 1753.1 $[M+2H]^{2+}$, 1168.5 $[M+3H]^{3+}$, 878.8 $[M+4H]^{4+}$, 782.9 $[M+5H]^{5+\cdot}$

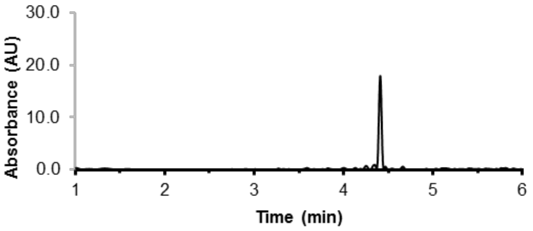


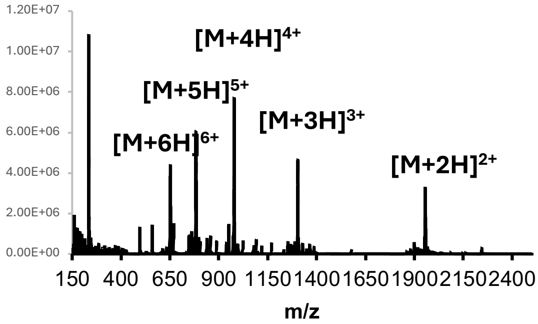


*Figure S 48 UPLC trace of **AO$_7$D** (UPLC Conditions: The conditions of the UPLC method are as follows: Solvent A: Water +0.1% Formic acid; Solvent B: Tetrahydrofuran +0.1% Formic acid; Gradient of 0-4 minutes 30% - 100%B + 2 minute 100% B with re-equilibration time of 2 minutes. Flow rate: 0.4 ml/min; Column temperature of 40 °C; Injection volume of 2 µL. The signal was monitored at 254 nm) and ESI-MS.*

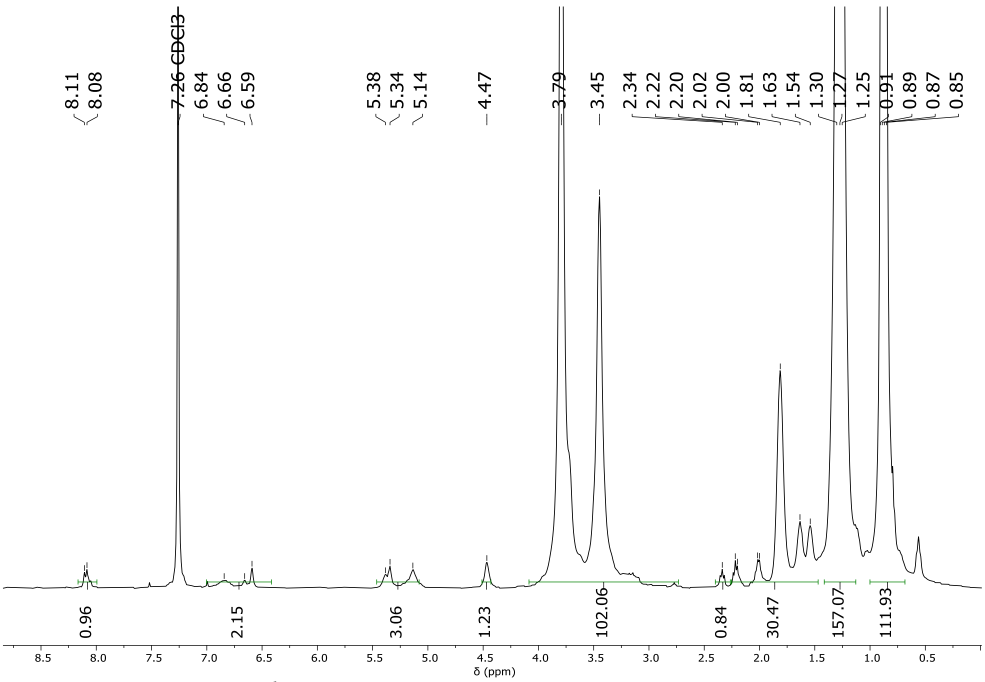


*Figure S 49 $^{1}$H NMR (400 MHz, CDCl$_3$) spectrum of compound **AO$_7$D**.*

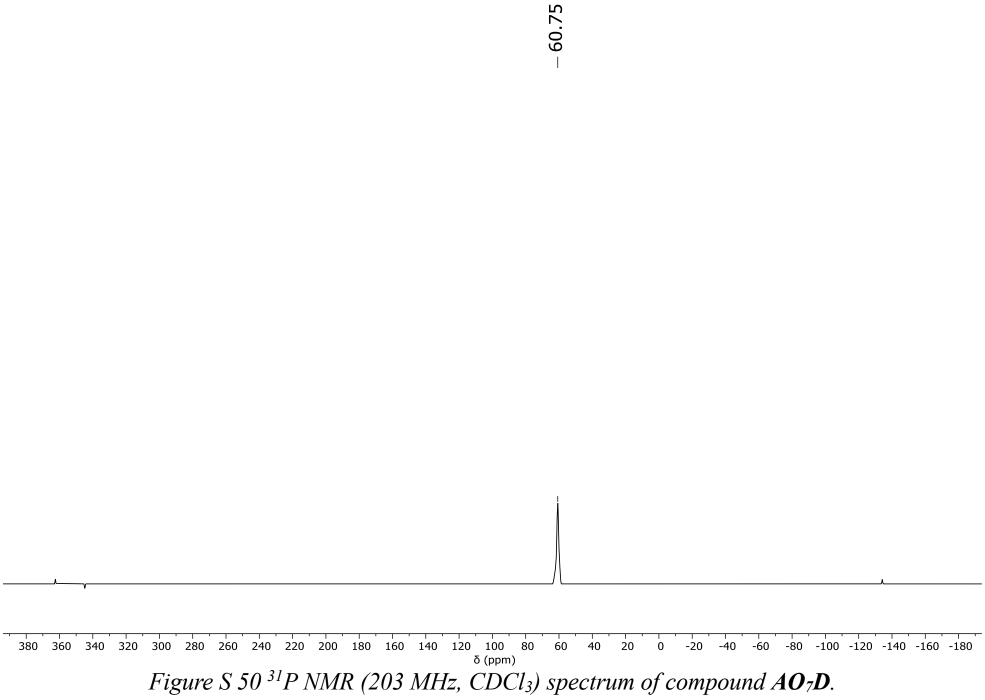


*Figure S 50 $^{31}$P NMR (203 MHz, CDCl$_3$) spectrum of compound **AO$_7$D**.*

## AO$_8$D

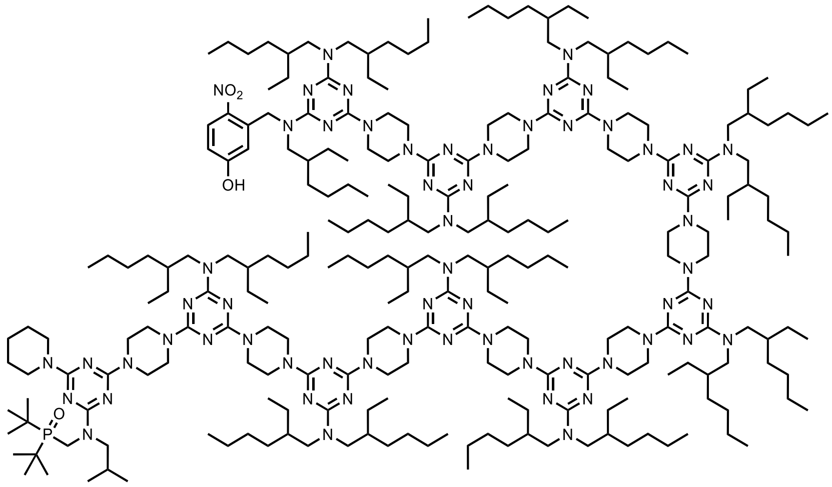

**$^{1}$H NMR** (400 MHz, CDCl$_3$) $\delta_H$ 8.08 (m, 1H), 7.10 (m, 1H), 6.65 (m, 1H), 5.10 (m, 2H), 4.45 (m, 2H), 3.50 (m, 116H), 2.20 (m, 1H), 1.85 - 1.65 (m, 25H), 1.25 (m, 170H), 0.90 (m, 120H).
**$^{31}$P NMR** (162 MHz, CDCl$_3$) $\delta_p$ 60.2.
**HRMS (ES+):** calcd. for $[C_{247}H_{447}N_{58}O_4P+H]^+$ is 4309.6246, found 4309.6216 (-3.10 ppm)
**UPLC trace** *UPLC Conditions*: The conditions of the UPLC method are as follows: Solvent A: Water +0.1% Formic acid; Solvent B: Tetrahydrofuran +0.1% Formic acid; Gradient of 0-4 minutes 30% - 100%B + 2 minute 100% B with re-equilibration time of 2 minutes. Flow rate: 0.4 ml/min; Column temperature of 40 °C; Injection volume of 2 µL. The signal was monitored at 254 nm.
**ESI-MS** Calculated Mass: 2156.7 $[M+2H]^{2+}$, 1426.1 $[M+3H]^{3+}$, 1078.9 $[M+4H]^{4+}$, 862.6 $[M+5H]^{5+\cdot}$ 719.8 $[M+6H]^{6+}$ and found (ESI+): 2156.7 $[M+2H]^{2+}$, 1426.1 $[M+3H]^{3+}$, 1079.0 $[M+4H]^{4+}$, 862.6 $[M+5H]^{5+}$, 719.8 $[M+6H]^{6+}$.

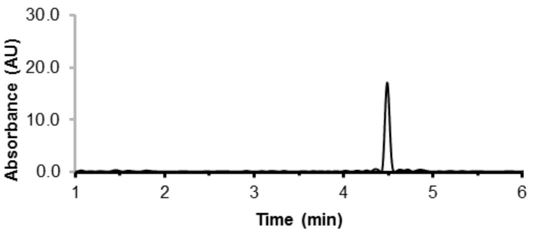


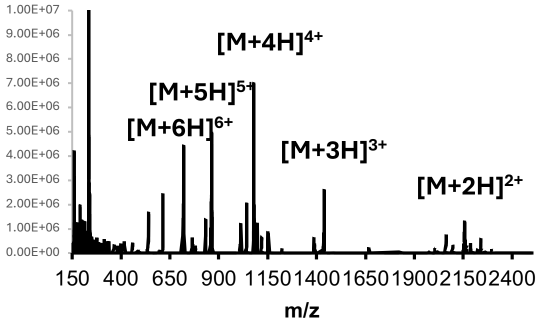


*Figure S 51 UPLC trace of **AO₈D** (UPLC Conditions: The conditions of the UPLC method are as follows: Solvent A: Water +0.1% Formic acid; Solvent B: Tetrahydrofuran +0.1% Formic acid; Gradient of 0-4 minutes 30% - 100%B + 2 minute 100% B with re-equilibration time of 2 minutes. Flow rate: 0.4 ml/min; Column temperature of 40 °C; Injection volume of 2 µL. The signal was monitored at 254 nm) and ESI-MS.*

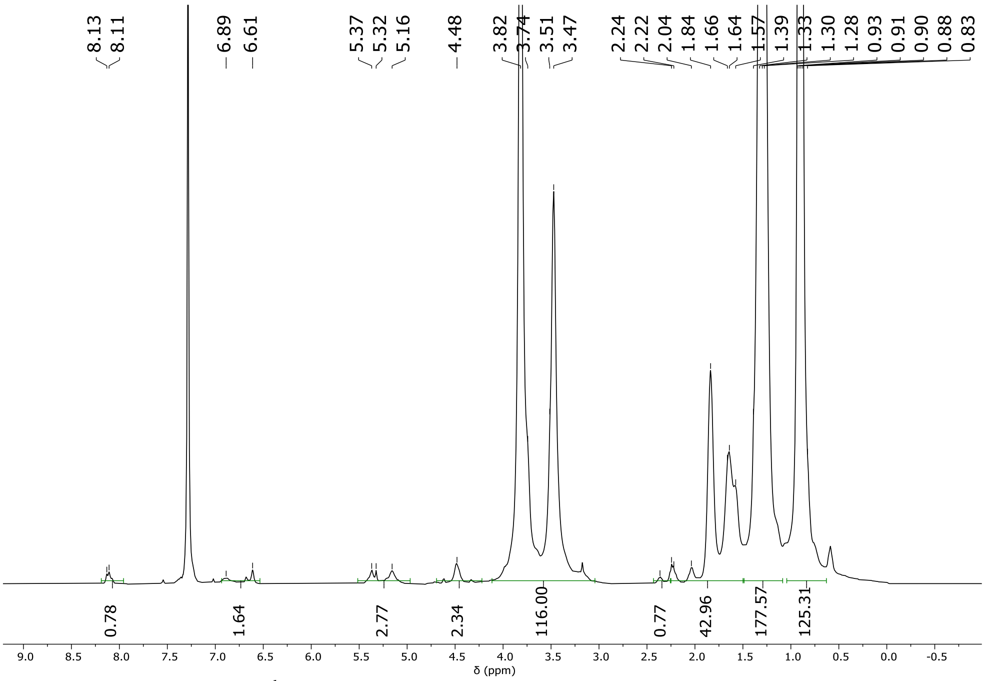


*Figure S 52 $^{1}$H NMR (400 MHz, $CDCl_3$) spectrum of compound* ***$AO_8D$***.

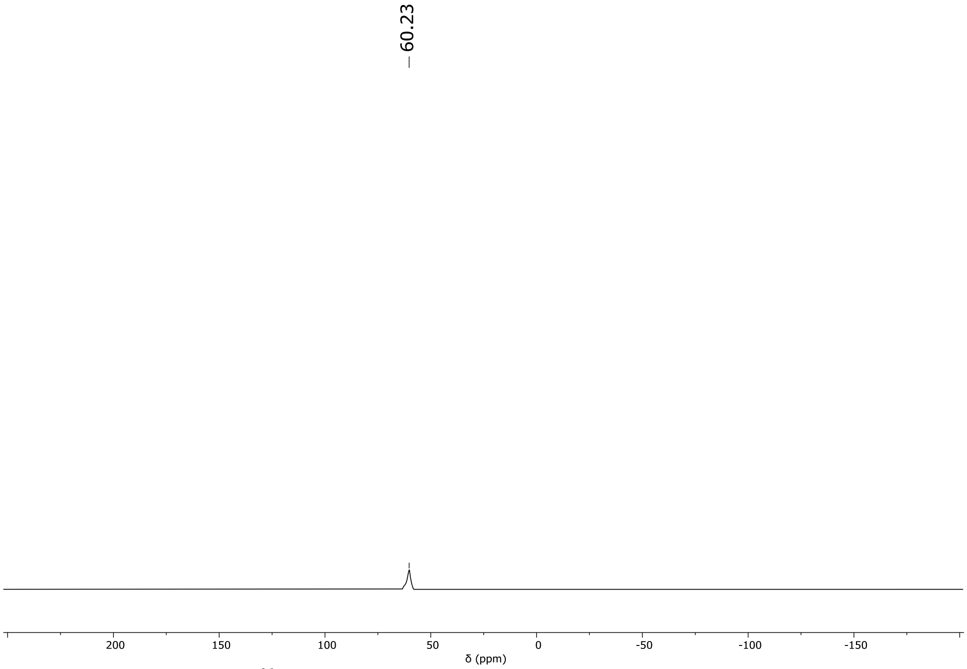


*Figure S 53 $^{31}$P NMR (162 MHz, $CDCl_3$) spectrum of compound* ***$AO_8D$***.

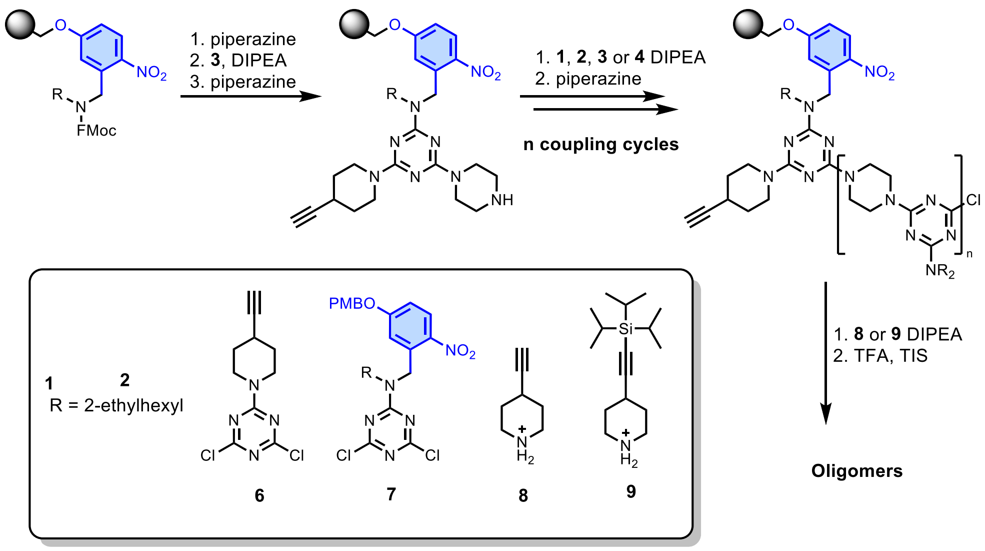


*Figure S 54 Reaction scheme summarizing the cycles in the automated solid phase synthesizer. The resin-bound amine is deprotected with piperazine; then subjected to repeated cycles of reacting with a dichloro building block and DIEPA, then with piperazine. In the last cycle, TIPS-protected or unprotected 4-ethylnyl piperidine is used instead of piperazine, then the oligomer is cleaved from the resin using TFA and TIS.*

# DAO3ADy

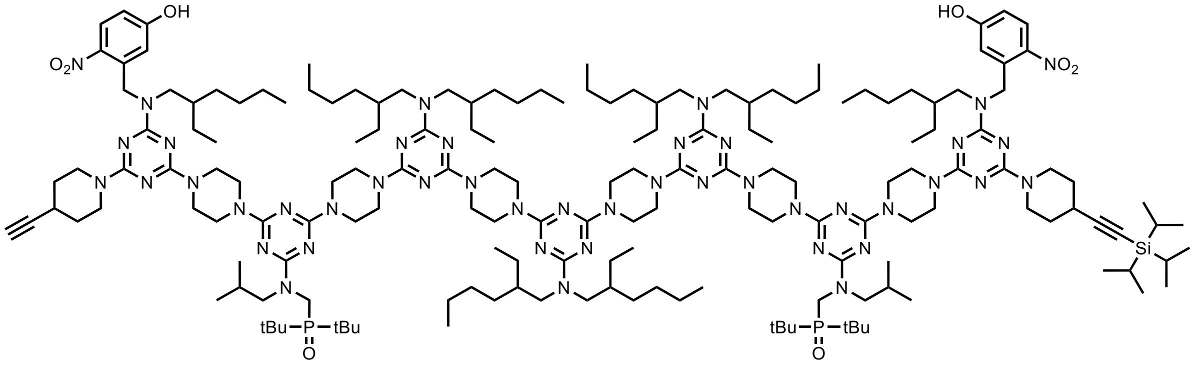


**$^1$H NMR** (400 MHz, $CDCl_3$) $\delta_H$ 8.08 (m, 2H), 6.88 (m, 2H), 6.65 (m, 2H), 5.50- 4.40 (m, 8H), 3.50 (m, 76H), 3.00 - 1.65 (m, 18H), 1.25 (m, 103H), 0.90 (m, 78H).
**$^{31}$P NMR** (162 MHz, $CDCl_3$) $\delta_P$ 62.8.
**HRMS (ES+):** calcd. for $[C_{172}H_{294}N_{44}O_8P_2Si+2H]^{2+}$ is 1598.1676, found 4598.1633 (-3.38 ppm)
**UPLC trace** *UPLC Conditions*: ACQUITY UPLC CSH C18 Column, 130Å, 1.7 µm, 2.1 mm X 50 mm, was used as the UPLC column. The conditions of the UPLC method are as follows: Solvent A: Acetonitrile +0.1% Formic acid; Solvent B: THF +0.1% Formic acid; Gradient of 0-1 minutes 0% B, 1-5 minutes 0% to 100% B + 1 minute 100% B with re-equilibration time of 1 minutes. Flow rate: 0.6 ml/min; Column temperature of 40°C; Injection volume of 2 µL. The signal was monitored at 254 nm.

**ESI-MS** Calculated Mass: 1598.15 $[M+2H]^{2+}$, 1065.77 $[M+3H]^{3+}$, 799.58 $[M+4H]^{4+}$, 639.86 $[M+5H]^{5+}$ and found (ESI+): 1598.15 $[M+2H]^{2+}$, 1065.75 $[M+3H]^{3+}$, 799.61 $[M+4H]^{4+}$, 639.85 $[M+5H]^{5+.}$

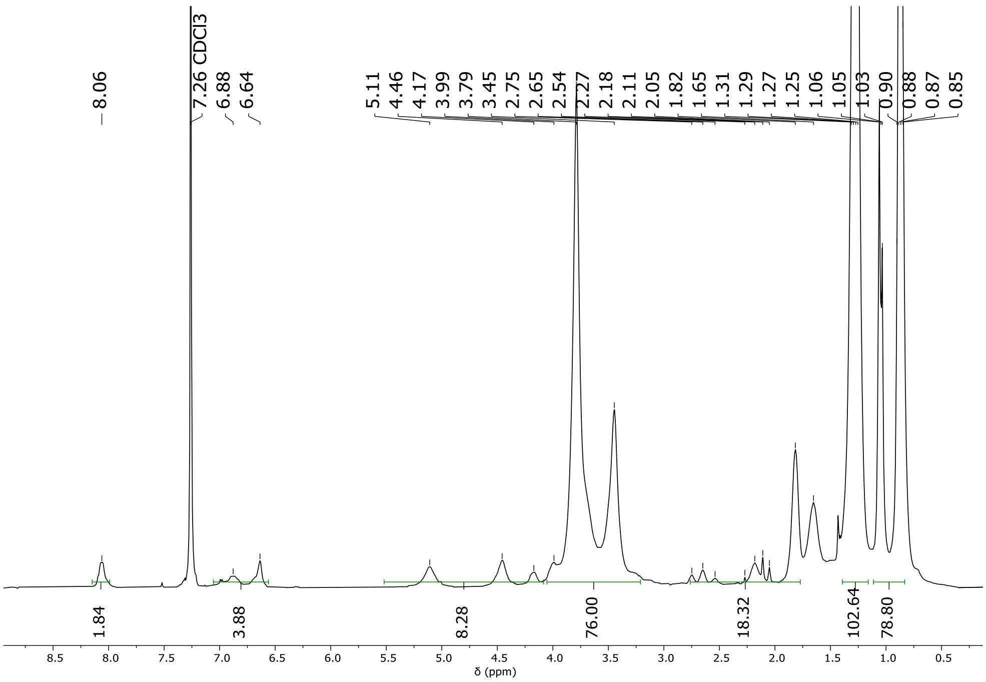


*Figure S 55 $^{1}H$ NMR (400 MHz, $CDCl_3$) spectrum of compound **$DAO_3AD$**.*

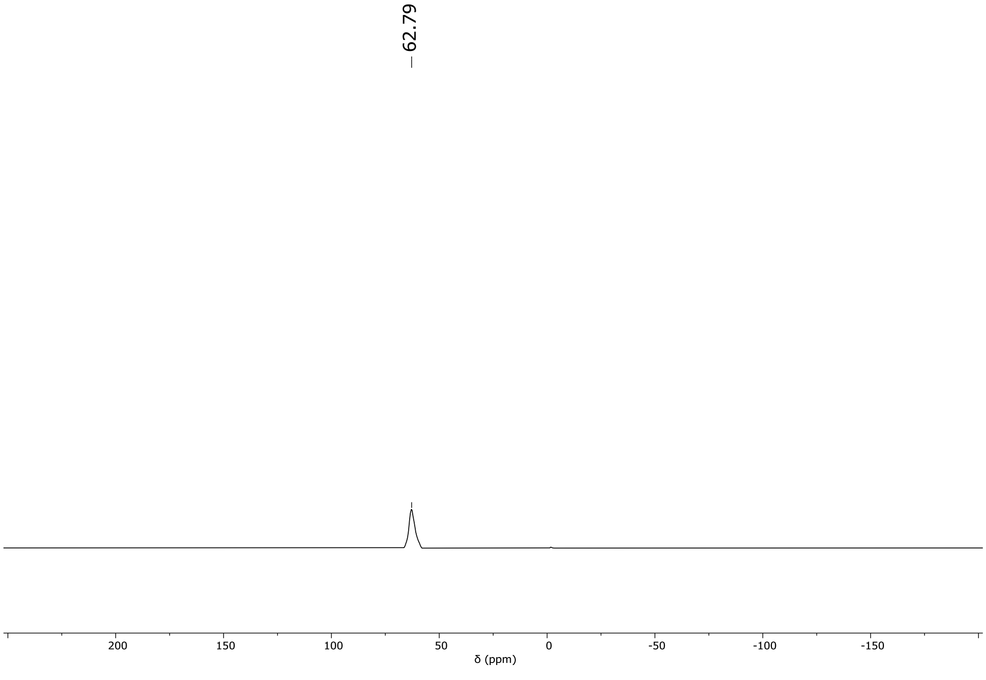


*Figure S 56 $^{31}P$ NMR (162 MHz, $CDCl_3$) spectrum of compound **$DAO_3AD$**.*

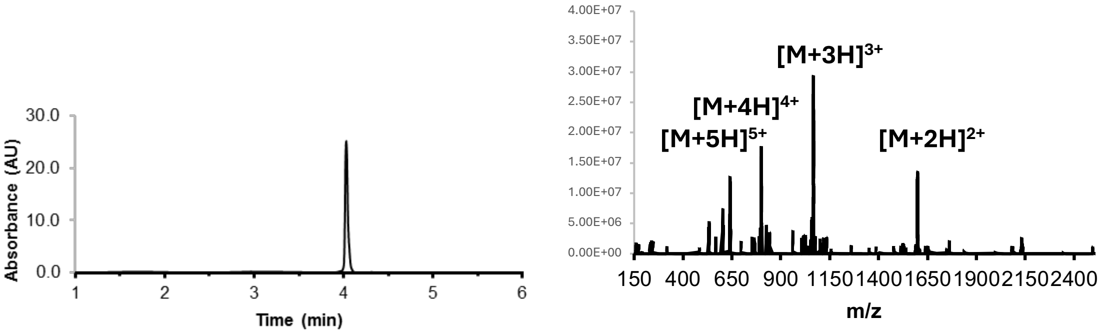


*Figure S 57 Figure 0-1 UPLC trace of* ***$DAO_3AD$*** ***(****UPLC Conditions: ACQUITY UPLC CSH C18 Column, 130Å, 1.7 µm, 2.1 mm X 50 mm, was used as the UPLC column. The conditions of the UPLC method are as follows: Solvent A: Acetonitrile +0.1% Formic acid; Solvent B: THF +0.1% Formic acid; Gradient of 0-1 minutes 0% B, 1-5 minutes 0% to 100% B + 1 minute 100% B with re-equilibration time of 1 minutes. Flow rate: 0.6 ml/min; Column temperature of 40°C; Injection volume of 2 µL. The signal was monitored at 254 nm) and ESI-MS.*

# DAO$_4$AD

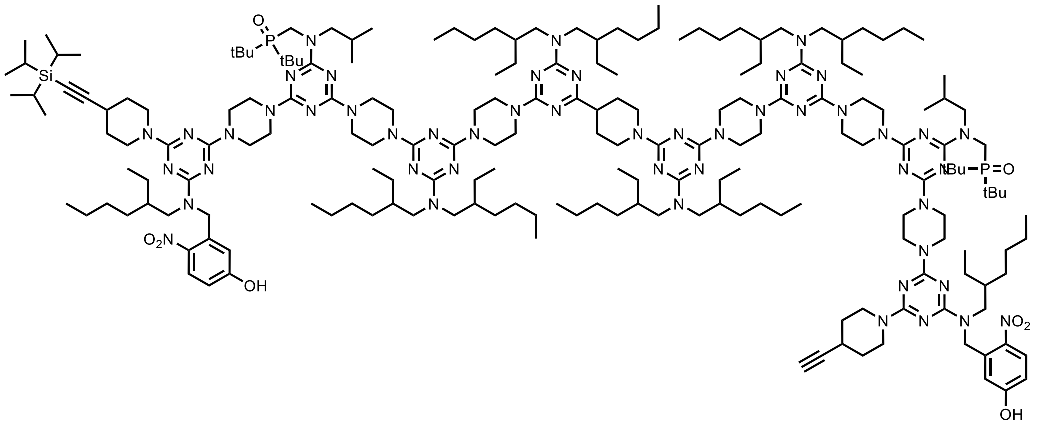

**$^{1}$H NMR** (400 MHz, CDCl$_3$) $\delta_H$ 8.08 (m, 2H), 6.88 (m, 2H), 6.65 (m, 2H), 5.50- 4.40 (m, 8H), 3.50 (m, 88H), 3.00 - 1.65 (m, 21H), 1.25 (m, 119H), 0.90 (m, 90H).
**$^{31}$P NMR** (162 MHz, CDCl$_3$) $\delta_p$ 62.7.
**HRMS (ES+):** calcd. for $[C_{198}H_{348}N_{50}O_8P_2Si+3H]^{3+}$ is 1199.8967, found 1199.8927 (-3.33 ppm).
**UPLC trace** *UPLC Conditions*: ACQUITY UPLC CSH C18 Column, 130Å, 1.7 µm, 2.1 mm X 50 mm, was used as the UPLC column. The conditions of the UPLC method are as follows: Solvent A: Acetonitrile +0.1% Formic acid; Solvent B: THF +0.1% Formic acid; Gradient of 0-1 minutes 0% B, 1-5 minutes 0% to 100% B + 1 minute 100% B with re-equilibration time of 1 minutes. Flow rate: 0.6 ml/min; Column temperature of 40°C; Injection volume of 2 µL. The signal was monitored at 254 nm.
**ESI-MS** Calculated Mass: 1798.84 $[M+2H]^{2+}$, 1199.56 $[M+3H]^{3+}$, 899.92 $[M+4H]^{4+}$ and found (ESI+): 1798.85 $[M+2H]^{2+}$, 1199.57 $[M+3H]^{3+}$, 899.98 $[M+4H]^{4+}$..

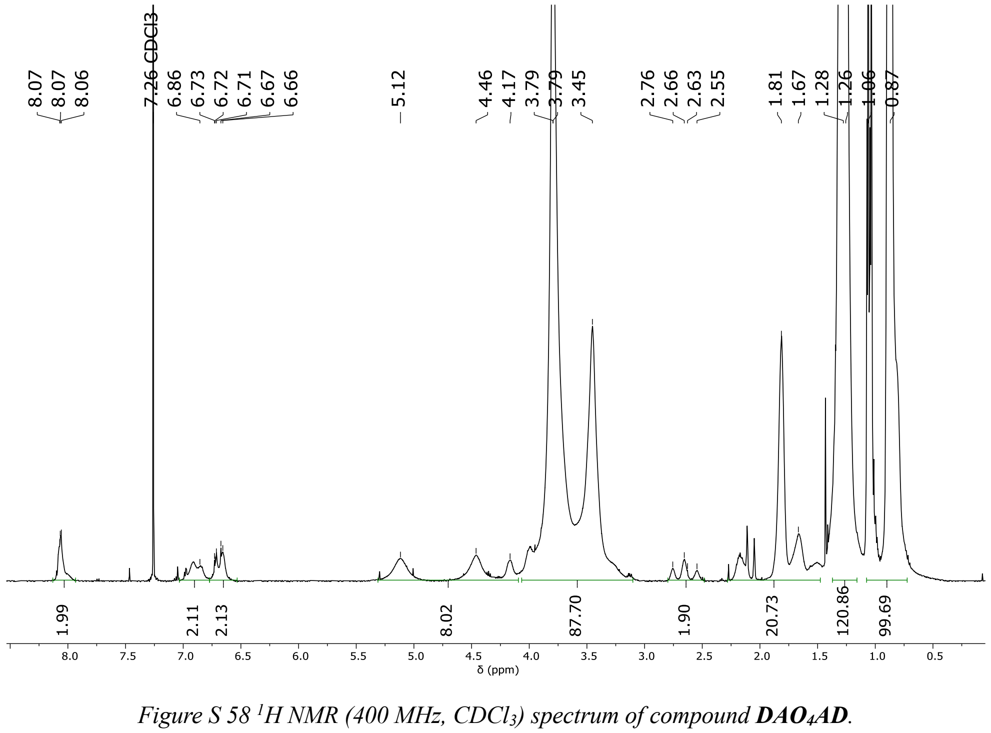


*Figure S 58 $^{1}$H NMR (400 MHz, CDCl$_3$) spectrum of compound **DAO$_4$AD**.*

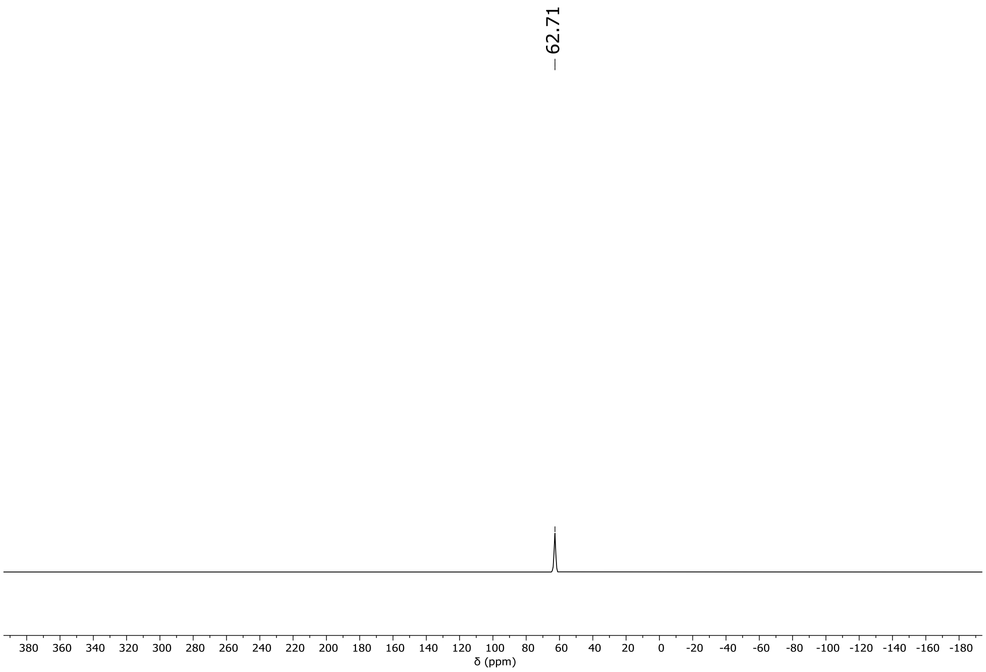


*Figure S 59 $^{31}P$ NMR (162 MHz, $CDCl_3$) spectrum of compound **$DAO_4AD$**.*

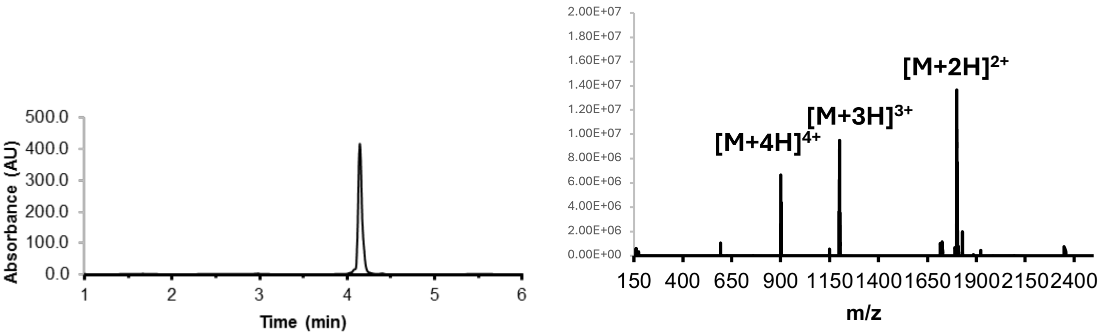


*Figure S 60 UPLC trace of **$DAO_4AD$** (UPLC Conditions ACQUITY UPLC CSH C18 Column, 130Å, 1.7 µm, 2.1 mm X 50 mm, was used as the UPLC column. The conditions of the UPLC method are as follows: Solvent A: Acetonitrile +0.1% Formic acid; Solvent B: THF +0.1% Formic acid; Gradient of 0-1 minutes 0% B, 1-5 minutes 0% to 100% B + 1 minute 100% B with re-equilibration time of 1 minutes. Flow rate: 0.6 ml/min; Column temperature of 40°C; Injection volume of 2 µL. The signal was monitored at 254 nm) and ESI-MS*

# ADO3AD

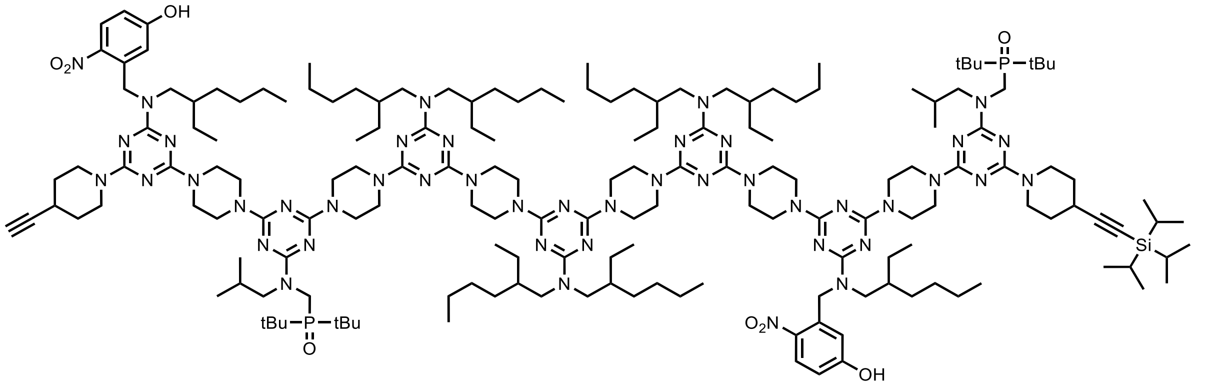

$^1$**H NMR** (400 MHz, $CDCl_3$) $\delta_H$ 8.08 (m, 2H), 6.88 (m, 2H), 6.65 (m, 2H), 5.50- 4.40 (m, 8H), 3.50 (m, 76H), 3.00 - 1.65 (m, 18H), 1.25 (m, 103H), 0.90 (m, 78H).
$^{31}$**P NMR** (162 MHz, $CDCl_3$) $\delta_p$ 62.2.
**HRMS (ES+):** calcd. for $[C_{172}H_{294}N_{44}O_8P_2Si+3H]^{3+}$ is 1065.7810, found 1065.7760 (-4.69 ppm).
**UPLC trace** *UPLC Conditions*: ACQUITY UPLC CSH C18 Column, 130Å, 1.7 µm, 2.1 mm X 50 mm, was used as the UPLC column. The conditions of the UPLC method are as follows: Solvent A: Acetonitrile +0.1% Formic acid; Solvent B: THF +0.1% Formic acid; Gradient of 0-1 minutes 0% B, 1-5 minutes 0% to 100% B + 1 minute 100% B with re-equilibration time of 1 minutes. Flow rate: 0.6 ml/min; Column temperature of 40°C; Injection volume of 2 µL. The signal was monitored at 254 nm.
**ESI-MS** Calculated Mass: 1598.15 $[M+2H]^{2+}$, 1065.77 $[M+3H]^{3+}$, 799.58 $[M+4H]^{4+}$, 639.86 $[M+5H]^{5+}$ and found (ESI+): 1598.15 $[M+2H]^{2+}$, 1065.75 $[M+3H]^{3+}$, 799.61 $[M+4H]^{4+}$, 639.85 $[M+5H]^{5+.}$

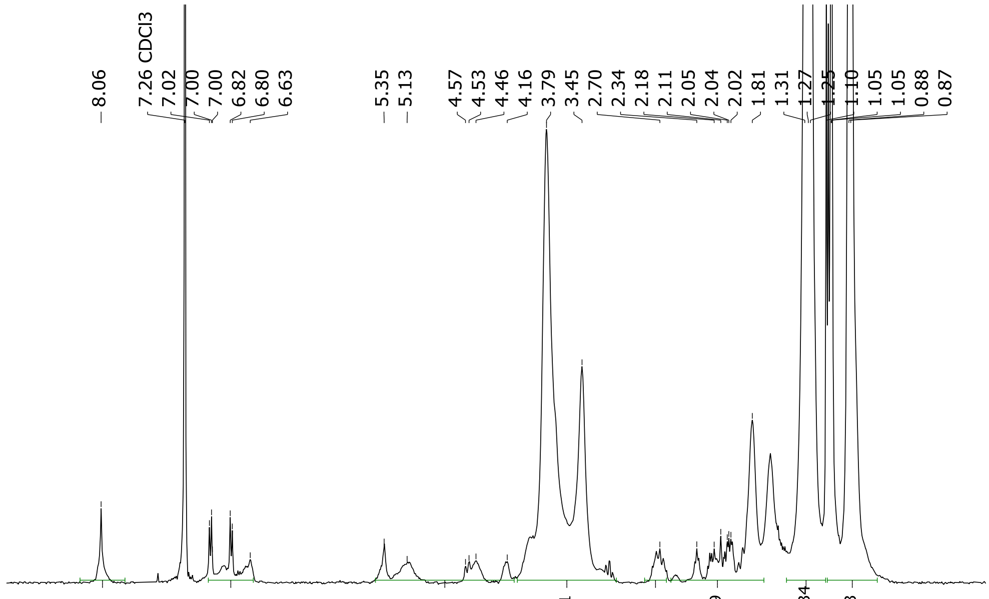

*Figure S 61 $^1$H NMR (400 MHz, $CDCl_3$) spectrum of compound* ***ADO3AD***.

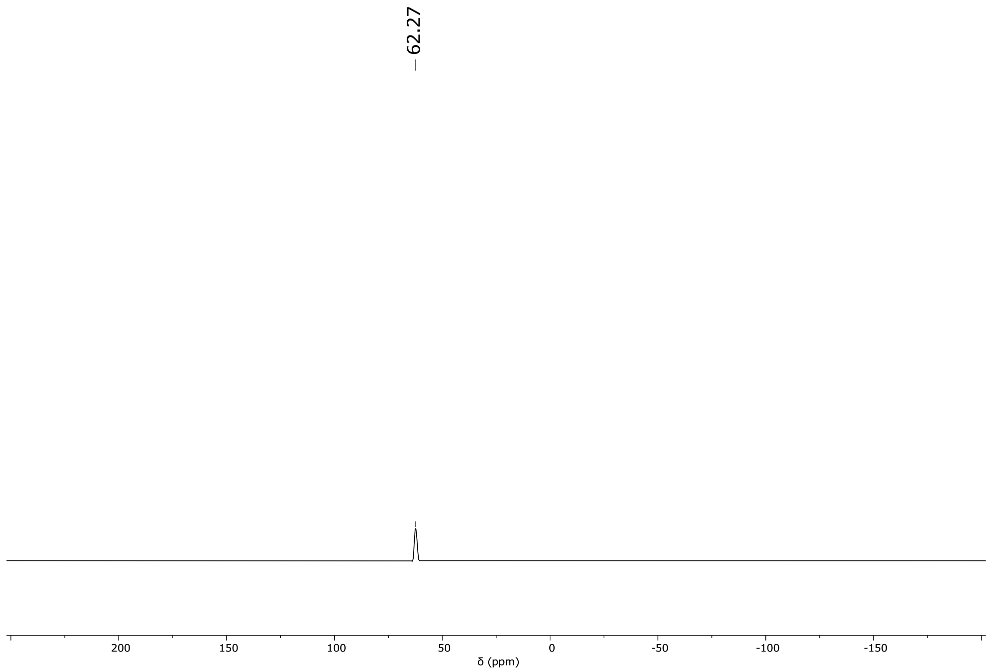


*Figure S 62 $^{31}P$ NMR (162 MHz, $CDCl_3$) spectrum of compound **$ADO_3AD$***

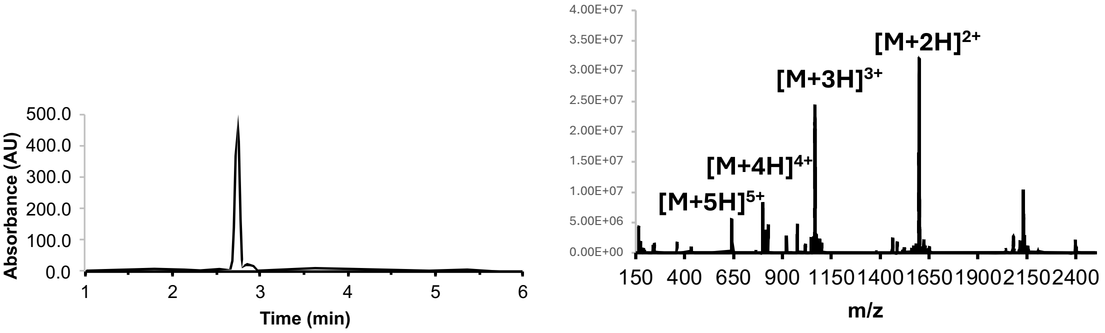


*Figure S 63 UPLC trace of **$ADO_3AD$** (UPLC Conditions: ACQUITY UPLC CSH C18 Column, 130Å, 1.7 µm, 2.1 mm X 50 mm, was used as the UPLC column. The conditions of the UPLC method are as follows: Solvent A: Acetonitrile +0.1% Formic acid; Solvent B: THF +0.1% Formic acid; Gradient of 0-1 minutes 0% B, 1-5 minutes 0% to 100% B + 1 minute 100% B with re-equilibration time of 1 minutes. Flow rate: 0.6 ml/min; Column temperature of 40°C; Injection volume of 2 µL. The signal was monitored at 254 nm) and ESI-MS.*

# ADO$_4$AD

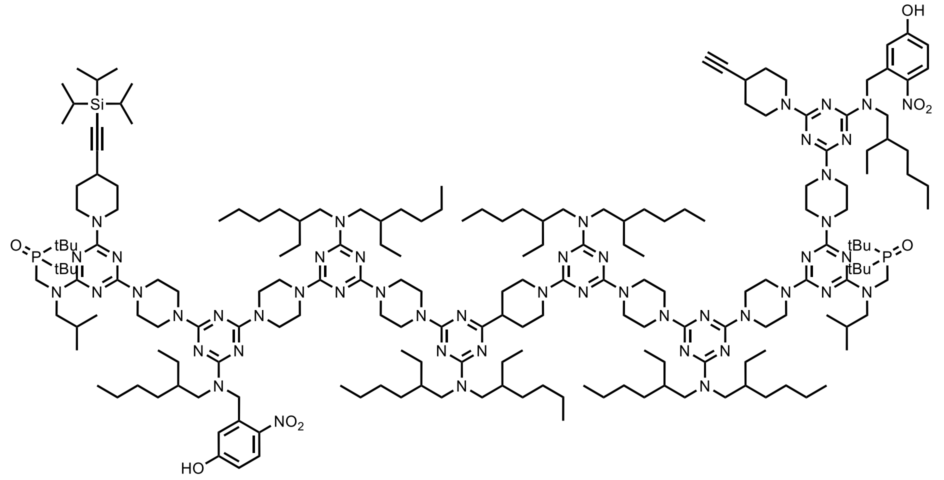

**$^{1}$H NMR** (400 MHz, $CDCl_3$) $\delta_H$ 8.08 (m, 2H), 6.88 (m, 2H), 6.65 (m, 2H), 5.50- 4.40 (m, 8H), 3.50 (m, 88H), 3.00 - 1.65 (m, 21H), 1.25 (m, 119H), 0.90 (m, 90H).
**$^{31}$P NMR** (162 MHz, $CDCl_3$) $\delta_p$ 63.3.
**HRMS (ES+):** calcd. for $[C_{198}H_{348}N_{50}O_8P_2Si+H]^+$ is 1199.8967, found 1199.8955 (-1.00 ppm).
**UPLC trace** *UPLC Conditions*: ACQUITY UPLC CSH C18 Column, 130Å, 1.7 µm, 2.1 mm X 50 mm, was used as the UPLC column. The conditions of the UPLC method are as follows: Solvent A: Acetonitrile +0.1% Formic acid; Solvent B: THF +0.1% Formic acid; Gradient of 0-1 minutes 0% B, 1-5 minutes 0% to 100% B + 1 minute 100% B with re-equilibration time of 1 minutes. Flow rate: 0.6 ml/min; Column temperature of 40°C; Injection volume of 2 µL. The signal was monitored at 254 nm.
**ESI-MS** Calculated Mass: 1798.84 $[M+2H]^{2+}$, 1199.56 $[M+3H]^{3+}$, 899.92 $[M+4H]^{4+}$ and found (ESI+): 1798.85 $[M+2H]^{2+}$, 1199.57 $[M+3H]^{3+}$, 899.98 $[M+4H]^{4+}$.

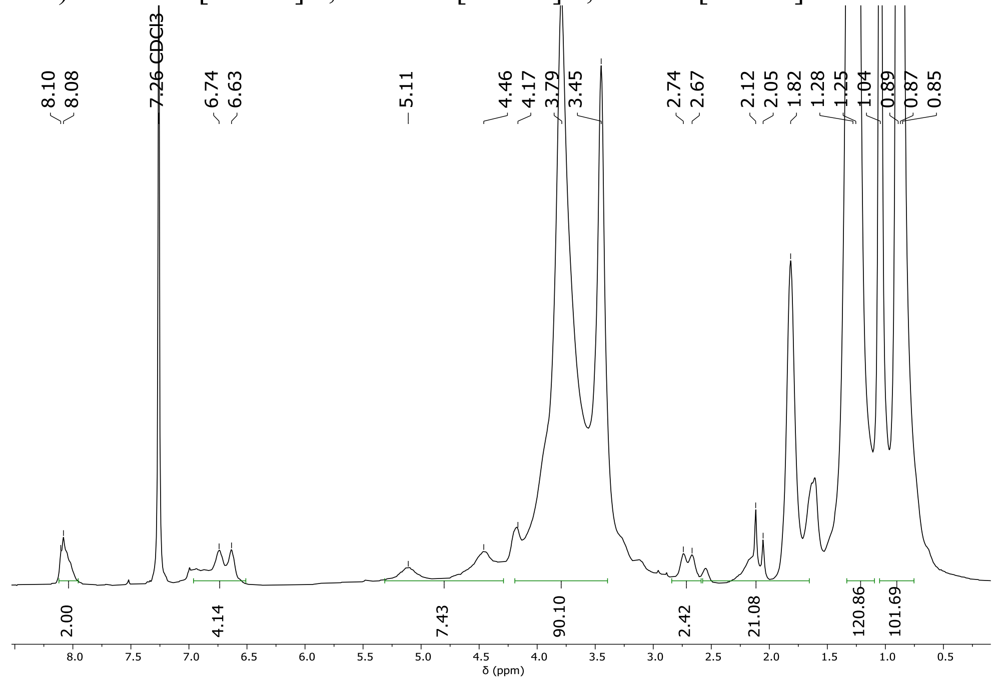


*Figure S 64 $^{1}$H NMR (400 MHz, $CDCl_3$) spectrum of compound **ADO$_4$AD**.*

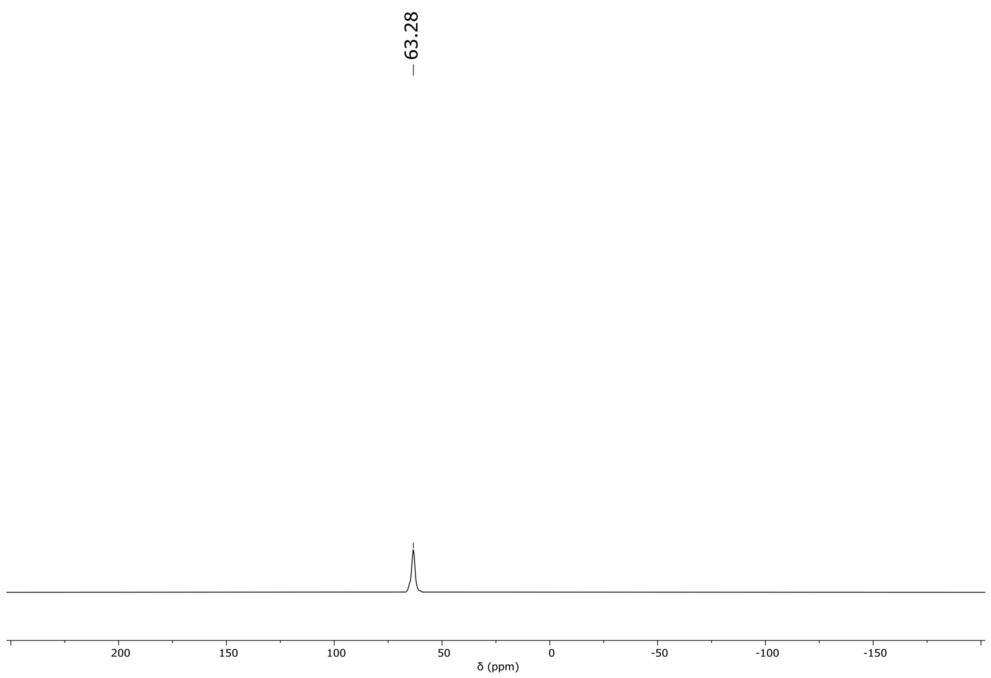


*Figure S 65 $^{31}$P NMR (162 MHz, CDCl$_3$) spectrum of compound **ADO$_4$AD**.*

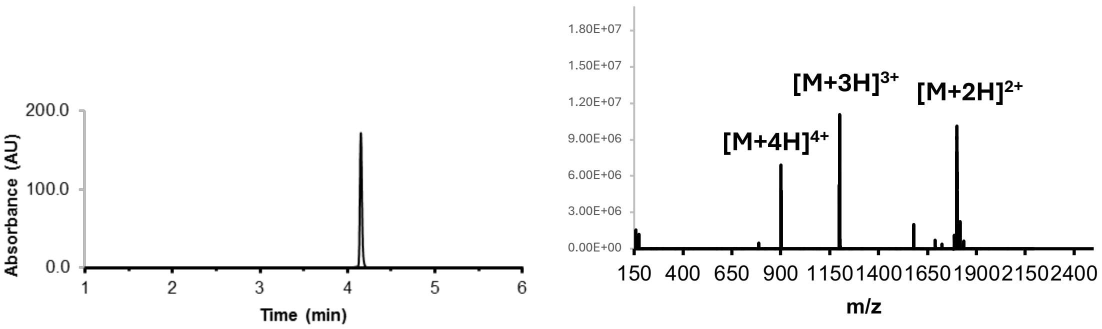


*Figure S 66 UPLC trace of **ADO$_4$AD (**UPLC Conditions: ACQUITY UPLC CSH C18 Column, 130Å, 1.7 µm, 2.1 mm X 50 mm, was used as the UPLC column. The conditions of the UPLC method are as follows: Solvent A: Acetonitrile +0.1% Formic acid; Solvent B: THF +0.1% Formic acid; Gradient of 0-1 minutes 0% B, 1-5 minutes 0% to 100% B + 1 minute 100% B with re-equilibration time of 1 minutes. Flow rate: 0.6 ml/min; Column temperature of 40°C; Injection volume of 2 µL. The signal was monitored at 254 nm) and ESI-MS.*

# ADO$_5$AD

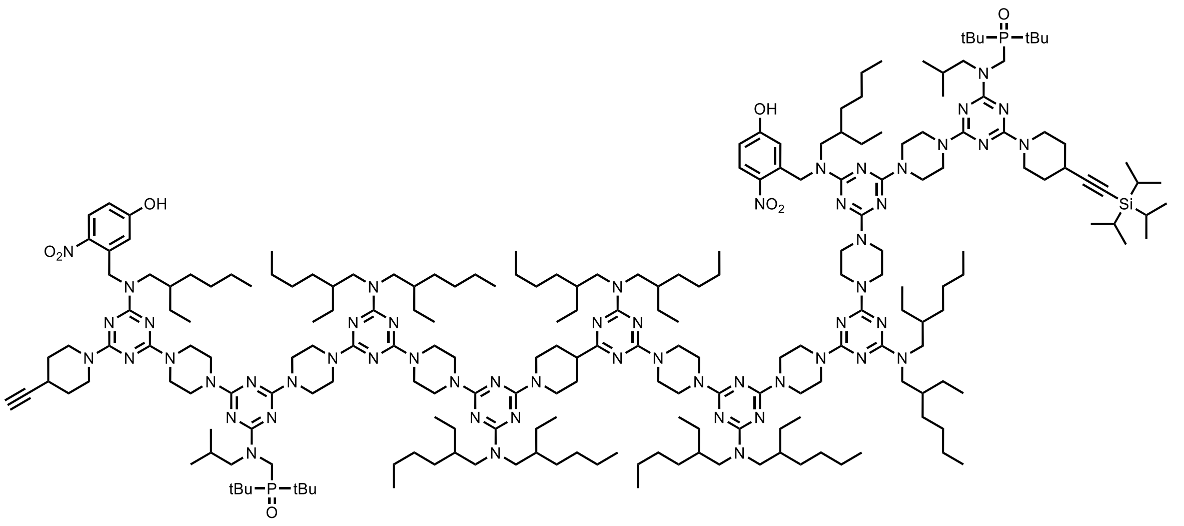


**$^1$H NMR** (400 MHz, $CDCl_3$) $\delta_H$ 8.08 (m, 2H), 6.88 (m, 2H), 6.65 (m, 2H), 5.50- 4.40 (m, 8H), 3.50 (m, 100H), 3.00 - 1.65 (m, 23H), 1.25 (m, 135H), 0.90 (m, 102H).
**$^{31}$P NMR** (162 MHz, $CDCl_3$) $\delta_p$ 62.8.
**HRMS (ES+):** calcd. for $[C_{219}H_{379}N_{55}O_8P_2Si+H]^+$ is 1334.0125, found 1334.0089 (-2.70 ppm).
**UPLC trace** *UPLC Conditions*: ACQUITY UPLC CSH C18 Column, 130Å, 1.7 µm, 2.1 mm X 50 mm, was used as the UPLC column. The conditions of the UPLC method are as follows: Solvent A: Acetonitrile +0.1% Formic acid; Solvent B: THF +0.1% Formic acid; Gradient of 0-1 minutes 0% B, 1-5 minutes 0% to 100% B + 1 minute 100% B with re-equilibration time of 1 minutes. Flow rate: 0.6 ml/min; Column temperature of 40°C; Injection volume of 2 µL. The signal was monitored at 254 nm.
**ESI-MS** Calculated Mass: 2000.51 $[M+2H]^{2+}$, 1334.01 $[M+3H]^{3+}$, 1000.76 $[M+4H]^{4+}$, 800.80 $[M+5H]^{5+}$ and found (ESI+): 2000.56 $[M+2H]^{2+}$, 1334.08 $[M+3H]^{3+}$, 1000.72 $[M+4H]^{4+}$, 800.83 $[M+5H]^{5+}$.

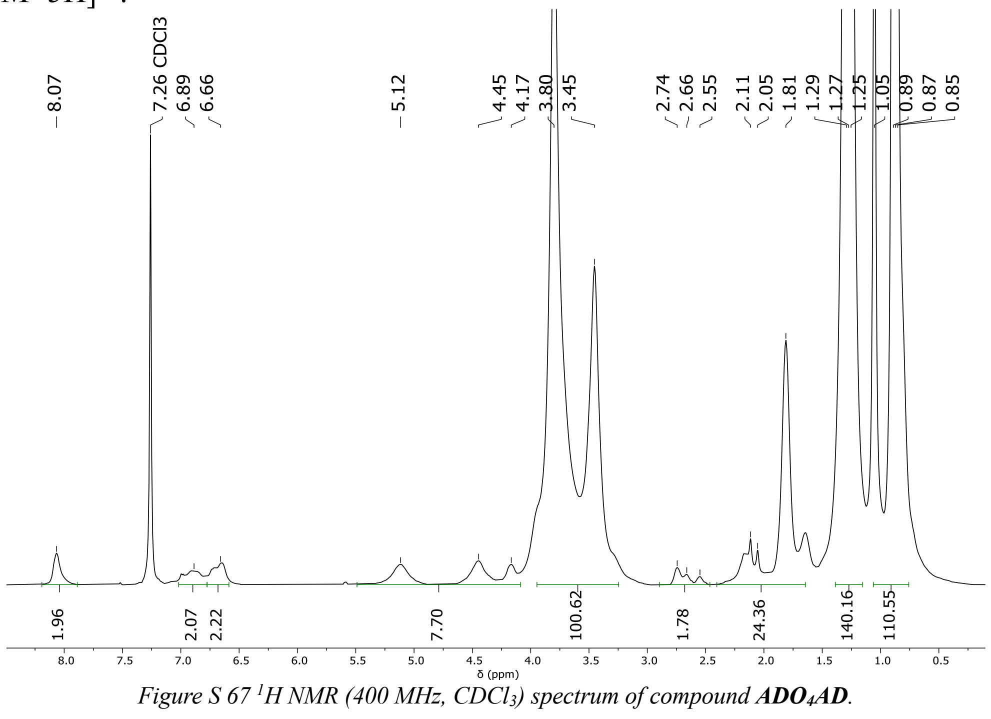


*Figure S 67 $^1$H NMR (400 MHz, $CDCl_3$) spectrum of compound* ***ADO$_4$AD***.

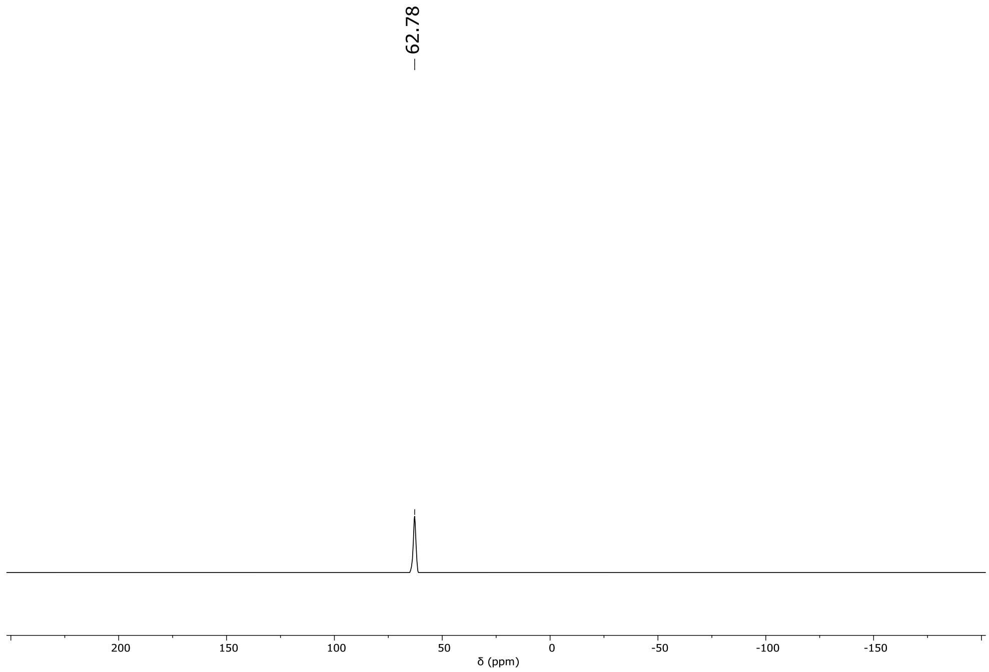


*Figure S 68 $^{31}$P NMR (162 MHz, CDCl$_3$)) spectrum of compound **ADO$_4$AD**.*

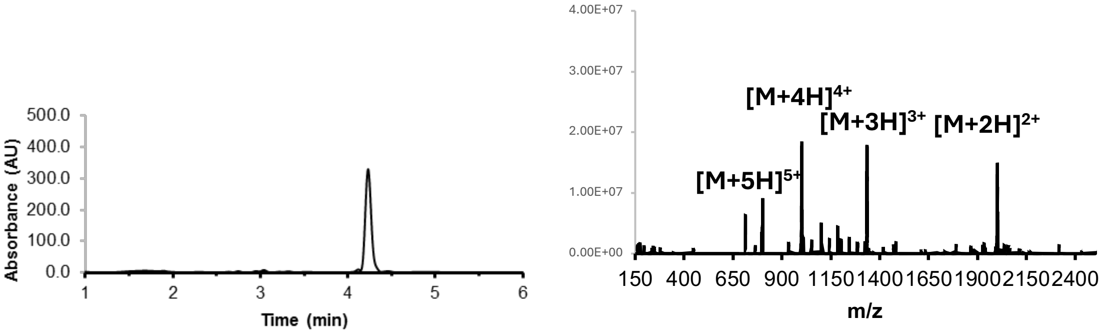


*Figure S 69 UPLC trace of **ADO$_4$AD** (UPLC Conditions: ACQUITY UPLC CSH C18 Column, 130Å, 1.7 µm, 2.1 mm X 50 mm, was used as the UPLC column. The conditions of the UPLC method are as follows: Solvent A: Acetonitrile +0.1% Formic acid; Solvent B: THF +0.1% Formic acid; Gradient of 0-1 minutes 0% B, 1-5 minutes 0% to 100% B + 1 minute 100% B with re-equilibration time of 1 minutes. Flow rate: 0.6 ml/min; Column temperature of 40°C; Injection volume of 2 µL. The signal was monitored at 254 nm) and ESI-MS.*

# X-ray structure of REMO 2-mer

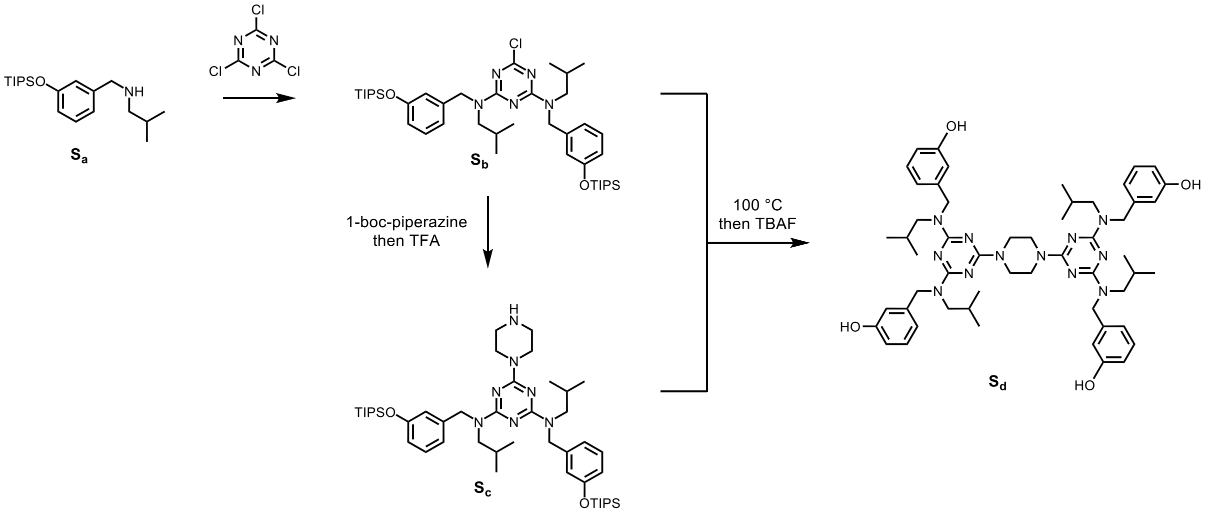


*Figure S 70 Synthetic route to compound* ***Sd*****.**

# Sa

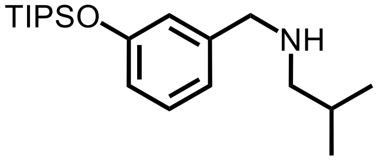


**Sa**

The synthesis of **Sa** was previously reported. [2]

## $S_b$

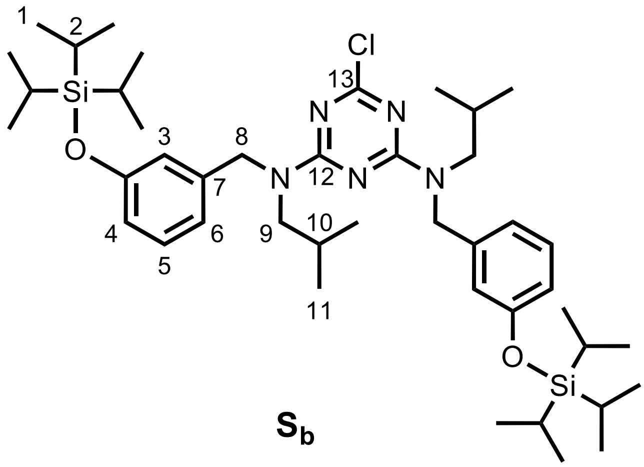


**$S_b$**

**$S_a$** (671 mg, 2.0 mmol, 2.5 eq) was dissolved in THF (10 mL). Cyanuric chloride (147 mg, 0.8 mmol, 1.0 eq) and DIPEA (1.4 mL, 8.0 mmol, 10.0 eq) were added and the solution was stirred overnight. The solvent was removed *in vacuo* and the residues were dissolved in EtOAc (30 mL). The solution was washed with citric acid solution (5% in water, 3 x 15 mL) and the combined aqueous phases were extracted with EtOAc (15 mL). The organic phase was dried over anhydrous magnesium sulfate. The crude was purified by flash column chromatography ($SiO_2$, 0 – 20% EtOAc in Pet. ether). **$S_b$** was obtained as a viscous colourless oil (523 mg, 0.668 mmol, 84%).

**$^1$H NMR** (500 MHz, $CDCl_3$**)** $\delta_H$ [7.18 – 7.03 (m, 2H, C5H)], [6.83 – 6.64 (m, 6H, C3H&C4H&C6H)], [4.80, 4.75 and 4.63 (s, 4H, C8H)], [3.40, 3.34, 3.25 and 3.15 (d, $J$ = 7.4 Hz, 4H, C9H)], [2.20 – 1.87 (m, 2H, C10H)], 1.26 – 1.16 (m, 6H, C2H), [1.09 – 1.03 (m, 36H, C1H)], [0.91, 0.89, 0.88 and 0.72 (d, $J$ = 6.7 Hz, 12H, C11H)];

**$^{13}$C NMR** (126 MHz, $CDCl_3$) $\delta_C$ 169.5 (C13), 165.8, 165.8 (C12), 156.4 (C-O), 139.3 (C7), 129.5 (C5), [120.7 – 118.4 (C3&C4&C6)], [53.9, 53.8, 53.7 and 53.6 (C9)], [50.5, 50.4, 50.3 and 50.2 (C8)], [27.3, 27.2, 27.0 and 26.9 (C10)], [20.5, 20.4, 20.3 and 20.3 (C11)], 18.0 (C1), 12.8 (C2);

**FT-IR (ATR):** $\nu_{max}$ /cm$^{-1}$ 2945, 2867, 1558, 1485, 1426, 1276, 1229, 1003, 981, 881, 823, 802, 779, 682;

**HRMS (ES+):** calcd. for $[C_{43}H_{73}N_5O_2Si_2Cl + H]^+$ is 782.4986, found 782.4951 (-4.4 ppm).

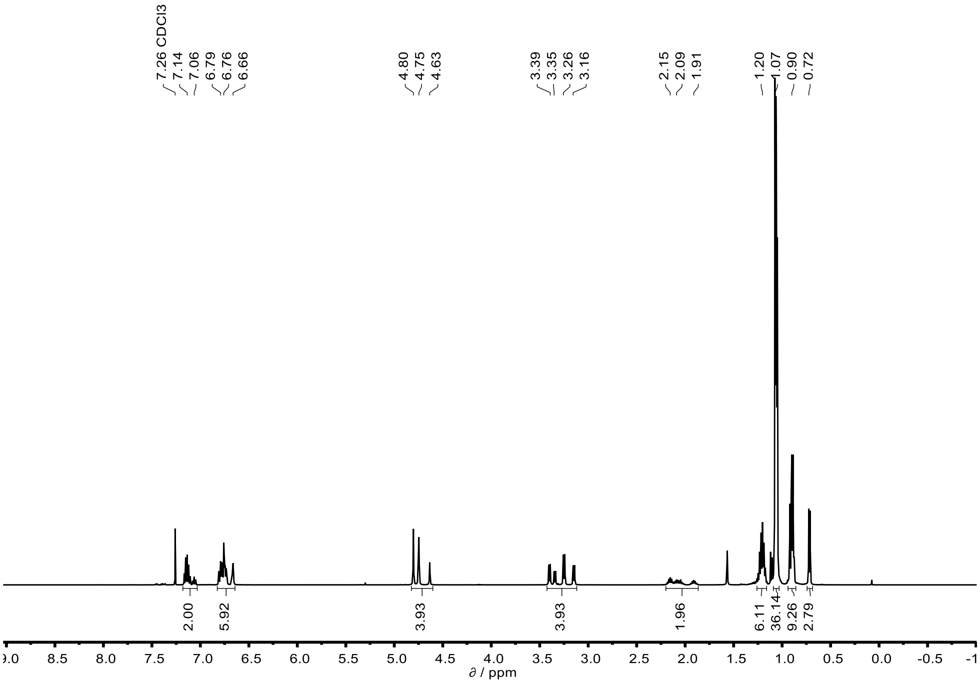

*Figure S 71 $^{1}H$ NMR spectrum (500 MHz, $CDCl_3$) of compound **$S_b$**.*

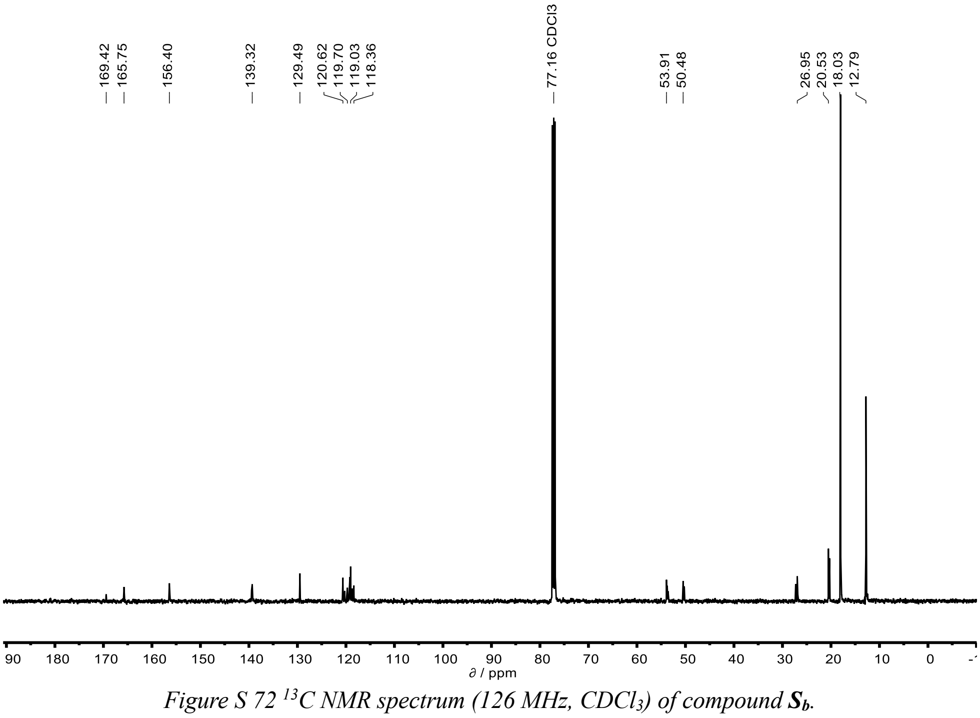

*Figure S 72 $^{13}C$ NMR spectrum (126 MHz, $CDCl_3$) of compound **$S_b$**.*

**S$_c$**

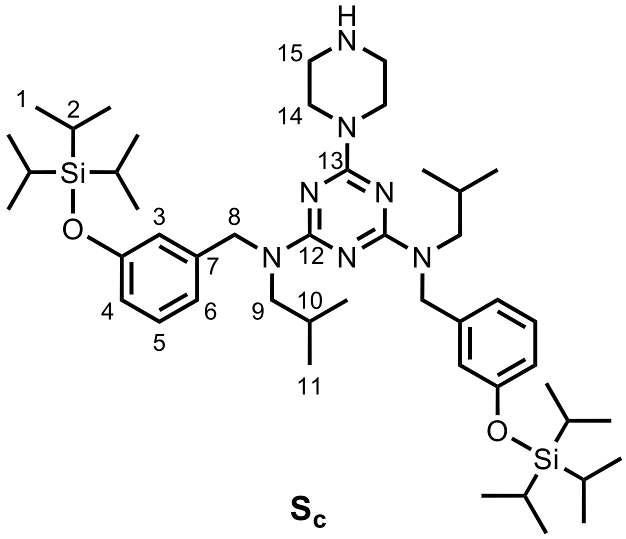


**S$_c$**

To a solution of **S$_b$** (414 mg, 0.528 mmol, 1.0 eq) in THF (4 mL) was added 1-boc-piperazine (394 mg, 2.12 mmol, 4.0 eq) and DIPEA (0.37 mL, 2.12 mmol, 4.0 eq). The solution was microwaved at 80 ºC for 4 hours. The solvent was removed *in vacuo* and the residues were dissolved in EtOAc (30 mL). The solution was washed with 0.1 M aqueous HCl solution (3 x 15 mL) and the combined aqueous phases were extracted with EtOAc (15 mL). The organic phase was dried over anhydrous magnesium sulfate and the solvent was removed *in vacuo.*

The crude from the previous step was dissolved in DCM (20 mL) and cooled to 0 ºC then TFA (1.2 mL) was added dropwise. The reaction mixture was stirred for 15 hours. The reaction mixture was diluted with DCM (15 mL) and washed with NaOH solution (1 M in water, 3 x 15 mL). The aqueous phases were extracted with DCM (15 mL) and the combined organic phases were dried over anhydrous magnesium sulfate and the solvent removed *in vacuo*. The crude was purified by flash column chromatography ($SiO_2$, 0 – 15% MeOH in DCM). **S$_c$** was obtained as a pale yellow oil (302 mg, 0.363 mmol, 69%).

**$^1$H NMR** (500 MHz, $CDCl_3$) $\delta_H$ [7.15 – 7.01 (m, 2H, C5H)], [6.85 – 6.67 (m, 6H,C3H&C4H&C6H)], [4.78, 4.74 and 4.68 (s, 4H, C8H)], [3.77, 3.71 and 3.65 (s, 4H, C14H)], [3.31, 3.27 and 3.18 (d, *J* = 7.3 Hz, 4H, C9H)], [2.90, 2.84 and 2.77 (m, 4H, C15H)], [2.24 – 1.88 (m, 2H, C10H)], 1.77 (s br, NH), 1.19 (m, 6H, C2H), 1.06 - 1.04 (m, 36H, C1H), [0.88 and 0.70 (m, 12H, C11H)];

**$^{13}$C NMR** (126 MHz, $CDCl_3$) $\delta_C$ 166.2 (C12), 165.5 (C13), 156.2 (C-O)], 141.3 (C7), 129.0 (C5), [120.4 - 118.0 (C3&C4&C6)], [53.9 and 53.8 (C9)], [50.2 and 50.0 (C8)], 46.1 (C14), 44.4 (C15), [27.6, 27.3 and 27.2 (C10)], [20.7, 20.5 and 20.4 (C11)], 18.1 (C1), 12.8 (C2);

**FT-IR (ATR):** $\nu_{max}$ /cm$^{-1}$ 2941, 2866, 1532, 1483, 1423, 1385, 1276, 982, 883, 826, 687;

**HRMS (ES+):** calcd. for $[C_{47}H_{81}N_7O_2Si_2 + H]^+$ is 832.6063, found 832.6022 (-4.9 ppm).

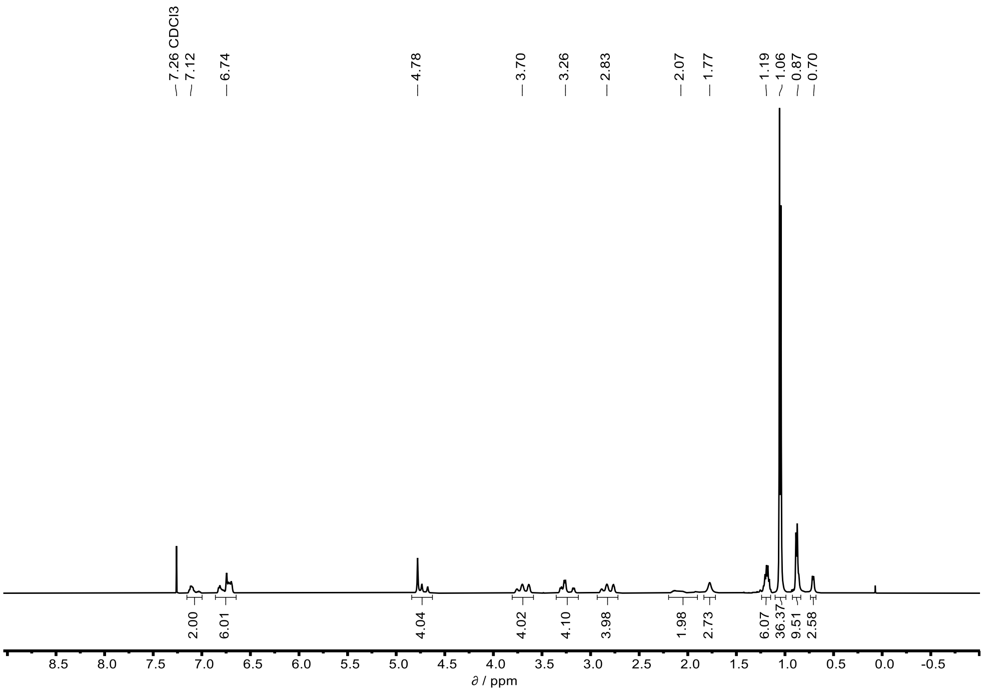


*Figure S 73 ¹H NMR spectrum (500 MHz, $CDCl_3$) of compound **S**$_c$.*

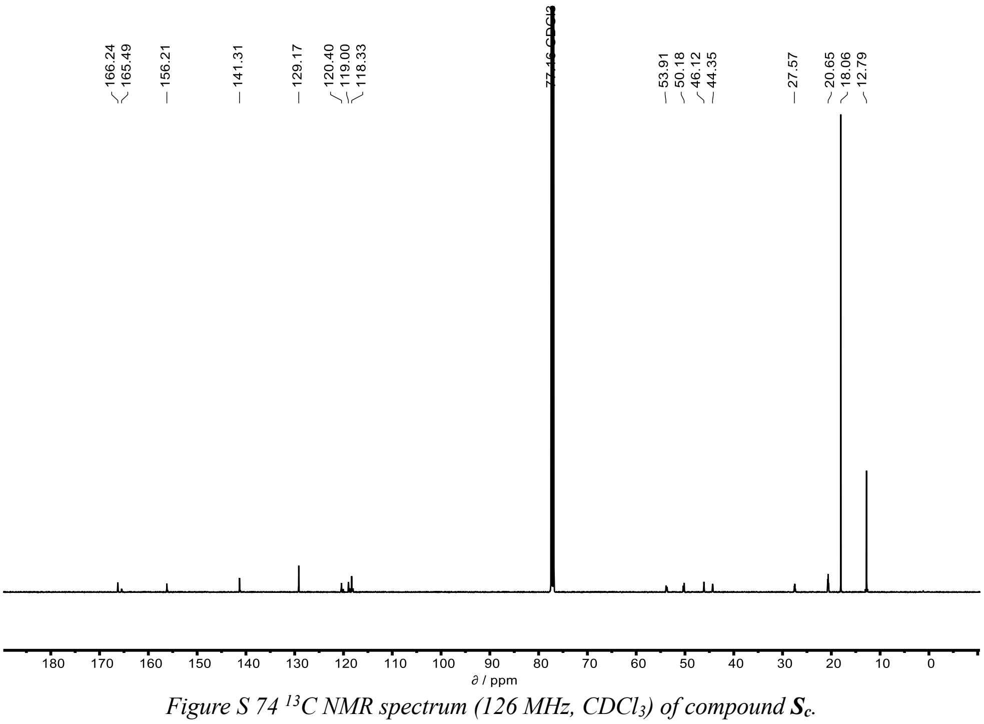


*Figure S 74 ¹³C NMR spectrum (126 MHz, $CDCl_3$) of compound **S**$_c$.*

**$S_d$**

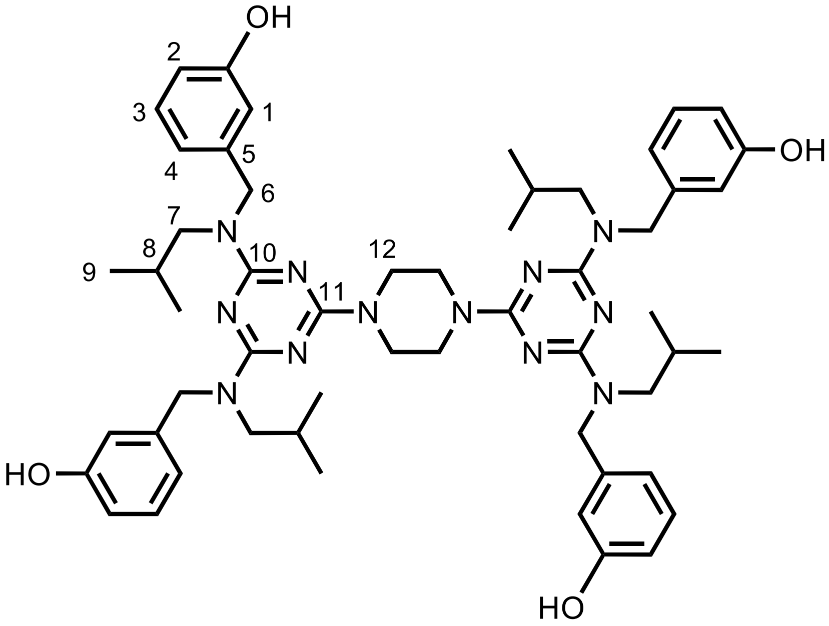


**$S_d$**

**$S_c$** (157 mg, 0.189 mmol, 2.0 eq), **$S_b$** (74 mg, 0.095 mmol, 1.0 eq) and DIPEA (0.10 mL, 0.57 mmol, 6.0 eq) were dissolved in THF (3 mL) and the solution was microwaved at 100 ºC for 6 hours. The solvent was removed *in vacuo* and the residues were dissolved in EtOAc (30 mL). The solution was washed with 0.1 M aqueous HCl solution (3 x 15 mL) and the combined aqueous phases were extracted with EtOAc (15 mL). The organic phase was dried over anhydrous magnesium sulfate and the solvent was removed *in vacuo.*

The crude was from the previous step was dissolved in THF (5 mL) and cooled to 0 ºC. TBAF (1 M in THF, 0.47 mL, 0.47 mmol, 5.0 eq) was added dropwise and the solution was stirred for 30 minutes. The solution was diluted with EtOAc (20 mL), washed with sat. ammonium chloride solution (2 x 10 mL), dried over anhydrous magnesium sulfate and the solvent removed *in vacuo*. The crude was purified by column chromatography ($SiO_2$, 0 – 25% EtOAc in DCM). **$S_d$** was obtained as a colourless oil (80 mg, 0.084 mmol, 89%).

**$^1$H NMR** (500 MHz, DMSO-$d_6$) $\delta_H$ [7.11 – 6.95 (m, 4H, C3H)], [6.70 – 6.54 (m, 12H, C1H&C2H&C4H)], [4.77 – 4.62 (m, 8H, C6H)], [3.79 – 3.57 (m, 8H, C12H)], [3.40 – 3.12 (m, 8H, C8H)], [2.17 – 1.84 (m, 4H, C8H)], [0.85 and 0.66 (m, 24H, C9H)];

**$^{13}$C NMR** (126 MHz, DMSO-$d_6$) $\delta_C$ 165.5 (C10), 164.7 (C11), 157.3 (C-O), 140.7 (C5), 129.2 (C3), [118.2, 117.5, 114.0, 113.7 and 113.4 (C1&C2&C4)], [53.4, 53.0 and 52.9 (C7)], [49.7, 49.4 and 49.3 (C6)], 42.5 (C12), [27.0, 26.8 and 26.6 (C8)], [20.4, 20.2 and 20.1 (C9)];

**FT-IR (ATR):** $\nu_{max}$ /cm$^{-1}$ 3315 (br), 2972, 1534, 1485, 1425, 1384, 1262, 1089, 1048, 881;

**HRMS (ES+):** calcd. for $[C_{54}H_{72}N_{12}O_4 + 2H]^{2+}$ is 477.2973, found 477.2957 (-3.3 ppm).

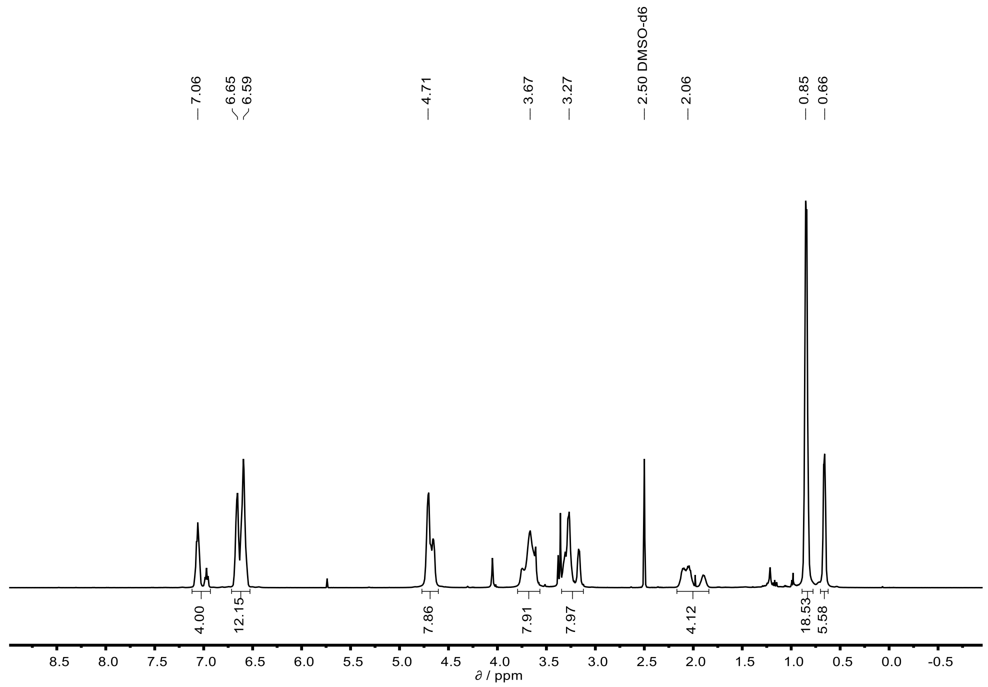


*Figure S 75 $^{1}$H NMR spectrum (500 MHz, DMSO-$d_6$) of compound $\mathbf{S}_d$.*

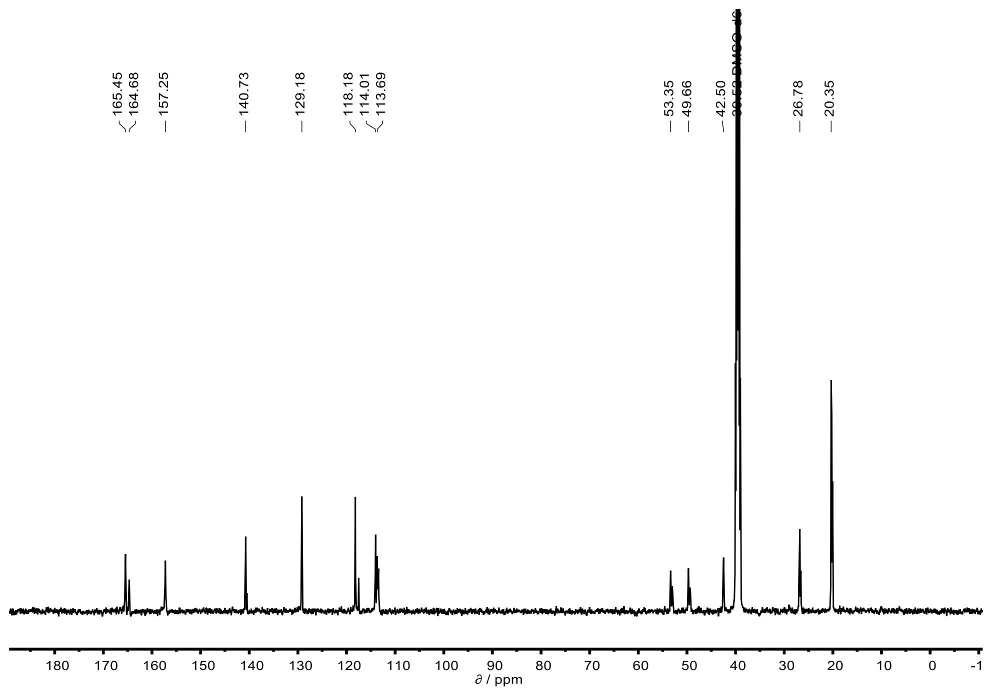


*Figure S 76 $^{13}$C NMR spectrum (126 MHz, DMSO-$d_6$) of compound $\mathbf{S}_d$.*

## Details of X-ray Crystallography

Single-crystal X-ray diffraction data for were collected on a Bruker D8-QUEST diffractometer, equipped with an Incoatec IμS Cu microsource (λ = 1.54178 Å) and a PHOTON-III detector. The temperature was maintained at 180(2) K. Data collection was performed using Bruker *APEX5*. Diffraction images were integrated using *SAINT*. Multi-scan absorption corrections were applied using *SADABS*. The structures were solved using *SHELXT* and refined using *SHELXL*.Molecular graphics were generated using Mercury. For compound $\mathbf{S_d}$, the *SQUEEZE* algorithm in *PLATON* was used to treat voids left by evaporation of dichloromethane molecules from the crystals.

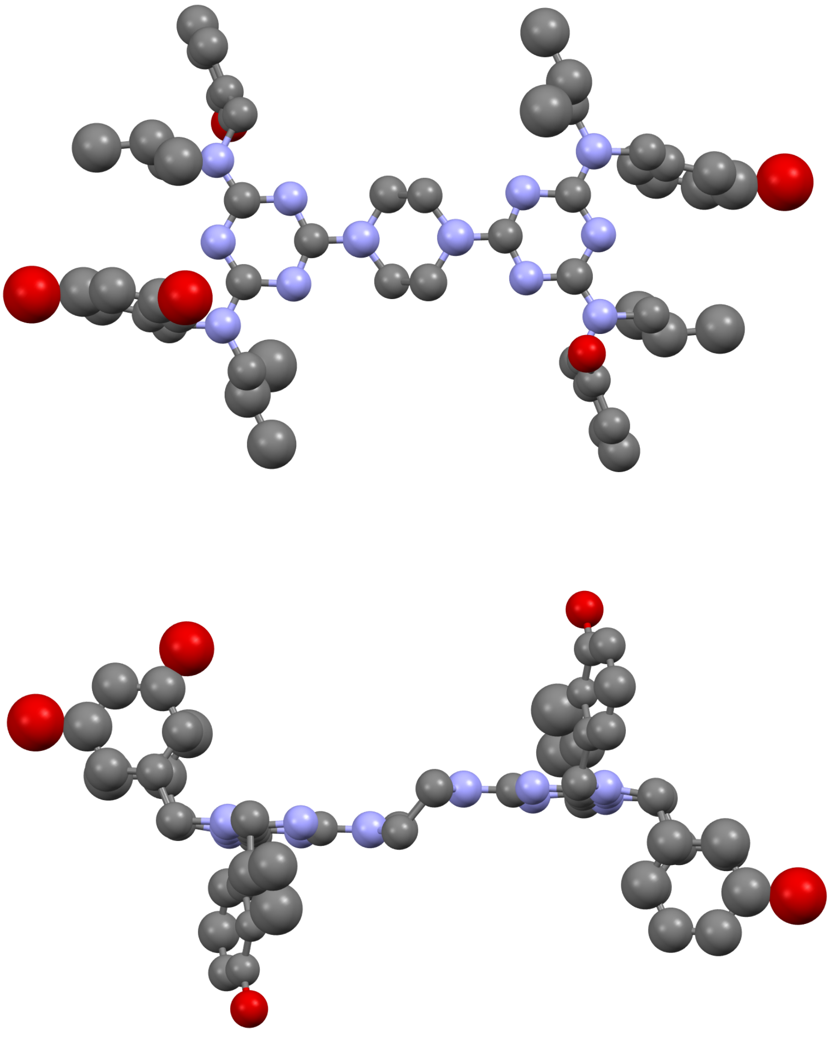

*Figure S 77 Top and side views of the molecular structure of* $\mathbf{S_d}$ *with displacement ellipsoids at 50% probability. H-atoms are omitted for clarity. A superposition of two orientations of the disordered phenol group is shown. Structure determined by Dr. Andrew Bond.*

*Table S 1 Crystal structure and refinement details for compound* ***$S_d$*** *and* ***9.***

| **Parameter** | **$S_d$** | **9** |
|---|---|---|
| Empirical formula | $C_{54}H_{72}N_{12}O_4$ | $C_{18}H_{32}F_3NO_2Si$ |
| CCDC Deposition Number | 2543578 | 2543579 |
| Formula weight | 953.23 | 379.53 |
| Temperature (K) | 180(2) | 180(2) |
| Crystal system | Monoclinic | Monoclinic |
| Space group | $P2_1/c$ | $P2_1/c$ |
| a (Å) | 17.386(14) | 22.132(3) |
| b (Å) | 9.761(8) | 8.3105(10) |
| c (Å) | 18.196(14) | 11.8637(13) |
| α (°) | 90 | 90 |
| β (°) | 94.57(2) | 92.366(7) |
| γ (°) | 90 | 90 |
| Volume ($Å^3$) | 3078(4) | 2180.2(4) |
| Z | 2 | 4 |
| Calc. density (g $cm^{-3}$) | 1.028 | 1.156 |
| F(000) | 1024 | 816 |
| Crystal size ($mm^3$) | 0.18 × 0.02 × 0.01 | 0.35 × 0.35 × 0.05 |
| Radiation | Cu Kα (λ = 1.54178 Å) | Cu Kα (λ = 1.54178 Å) |
| 2θ range for data collection (°) | 5.10–101.43 | 8.00-133.56 |
| Reflections collected | 12507 | 30090 |
| Independent reflections | 3184 | 3841 |
| Data / restraints / parameters | 3184 / 0 / 123 | 3841 / 45 / 254 |
| Goodness-of-fit on $F^2$ | 1.214 | 1.03 |
| Final R values [I > 2σ(I)] | $R_1$ = 0.1627 | $R_1$ = 0.0821, $wR_2$ = 0.2237 |
| Final R values (all data) | $R_1$ = 0.3703, $wR_2$ = 0.4111 | $R_1$ = 0.12353, $wR_2$ = 0.2636 |
| Largest diff. peak/hole (e $Å^{-3}$) | 0.551 / −0.303 | 0.396 / -0.322 |

# Titration data

## A•PFTB

The binding constant was measured by NMR titrations using a Bruker 400 MHz Avance III HD Smart Probe spectrometer. The host (**A**) was dissolved in $CD_2Cl_2$ at a known concentration. The guest (PFTB) was dissolved in the host solution and made to known concentration. 0.6 mL of host was added to an NMR tube and the spectrum was recorded. Aliquots of guest in host solution were added to the NMR tube and the $^{31}P$ and $^{1}H$ NMR spectra were recorded after each addition. The chemical shifts were monitored as a function of guest concentration and analysed using Musketeer (available from the GitHub repository, https://github.com/daniilS/Musketeer/releases). The changes in chemical shift were fit to a 1:1 binding isotherm. Errors are quoted as two standard deviations based on three different experiments.

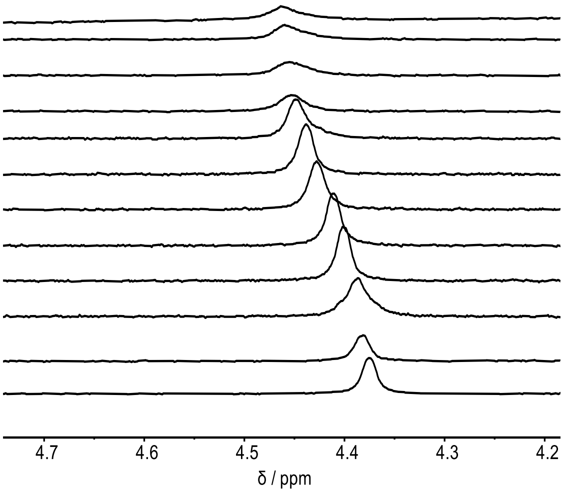


*Figure S 78 $^{1}H$ NMR titration of PFTB into **A** (0.67 mM) in deuterated-DCM at 298 K.*

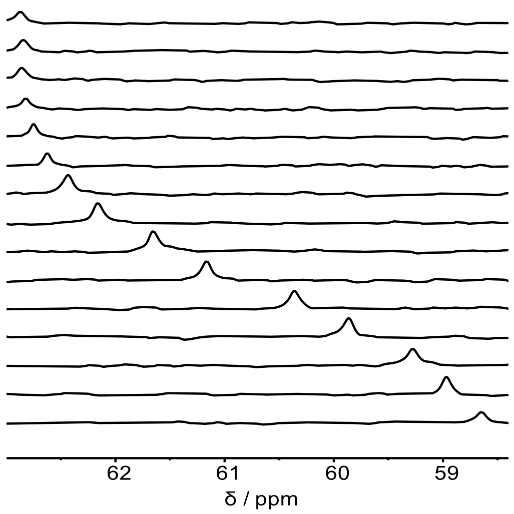


*Figure S 79 $^{31}$P NMR titration of PFTB into **A** (0.67 mM) in deuterated-DCM at 298 K.*

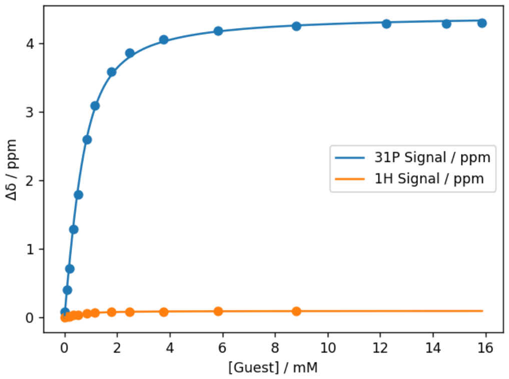


*Figure S 80 Fit of the change in $^{1}$H NMR and $^{31}$P NMR shift a 1:1 binding isotherm for the titration of PFTB into **A** (0.67 mM) in deuterated-DCM at 298 K.*

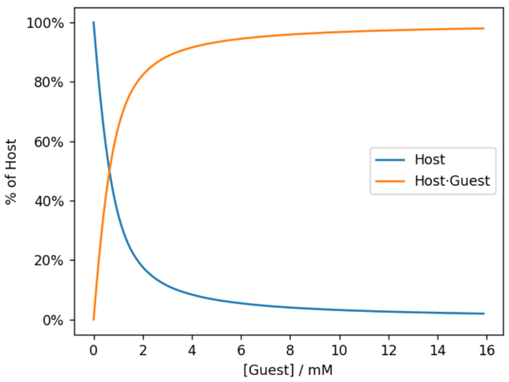


*Figure S 81 Speciation during the titration of PFTB into **A** (0.67 mM) in deuterated-DCM at 298 K.*

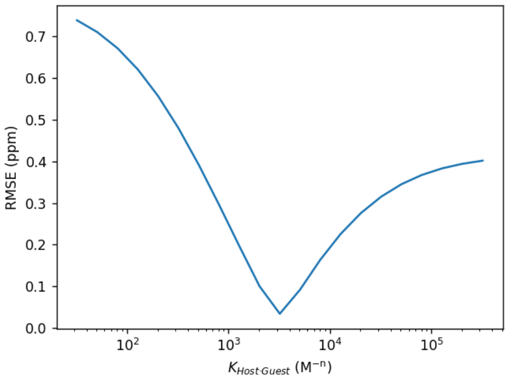


*Figure S 82 RMSE plot for the titration of PFTB into **A** (0.67 mM) in deuterated-DCM at 298 K.*

UV-vis titrations were carried out on an Agilent Cary 60 UV-Vis spectrophotometer, using standard titration protocols. A sample of the host (**D**, **DD**, **DOD**, **DO$_n$A**, **DAO$_n$AD** or **ADO$_n$AD**) was prepared at a known concentration in DCM (spectroscopic grade). The UV-vis spectrum of the free host (2 mL) was recorded. The guest (**A**, **AA**, **AOA** or PFTB) was dissolved in 2 mL of the host solution at a known concentration. Aliquots of the guest solution were successively added to the cuvette, and the UV-vis absorption spectrum was recorded after each addition. The UV-vis absorption spectra were analysed using Musketeer[1] to fit the changes in the absorption at fixed wavelengths to a 1:1 binding isotherm by optimizing the association constant and absorption of the free and bound host, or to a complex model by optimizing the effective molarity of folding and absorption of the free and bound 4-nitrophenol.

# A•D

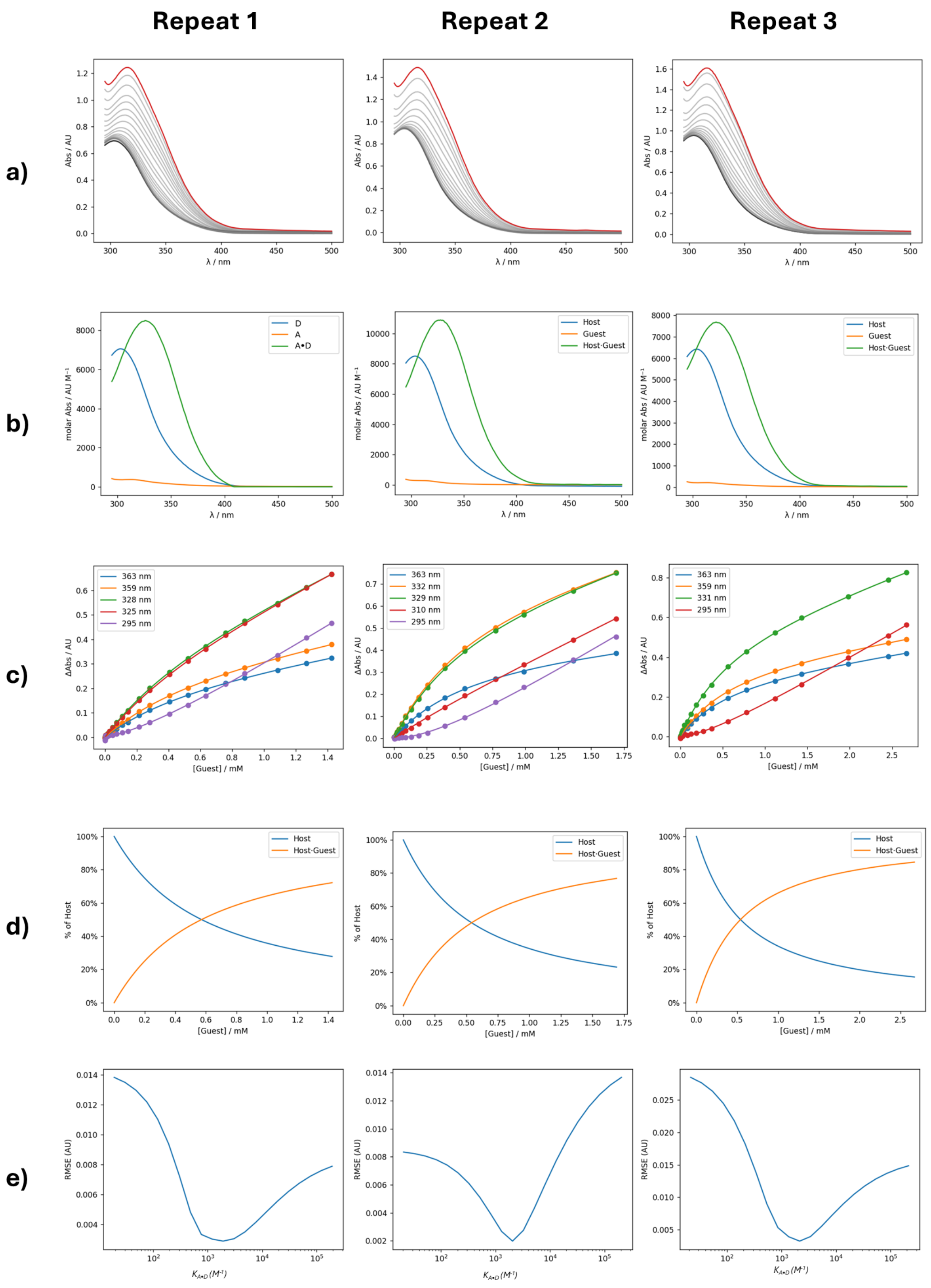


*Figure S 83 UV-Vis absorption titration of **A** into **D** (100 µM) in dichloromethane at 298 K. a) UV-Vis absorption spectra showing free **D** in black and final spectrum in red. b) Fitted spectra for **D** (host) and **A•D** (host•guest) c) Best fit of the change in UV-Vis absorbance at selected wavelengths to a 1:1 binding isotherm allowing for guest absorption. d) Calculated populations of different species containing **D**. e) Relationship between the RMSE between the experimental data and calculated spectra plotted as a function of the value of $K_{A•D}$.*

# AA•DD

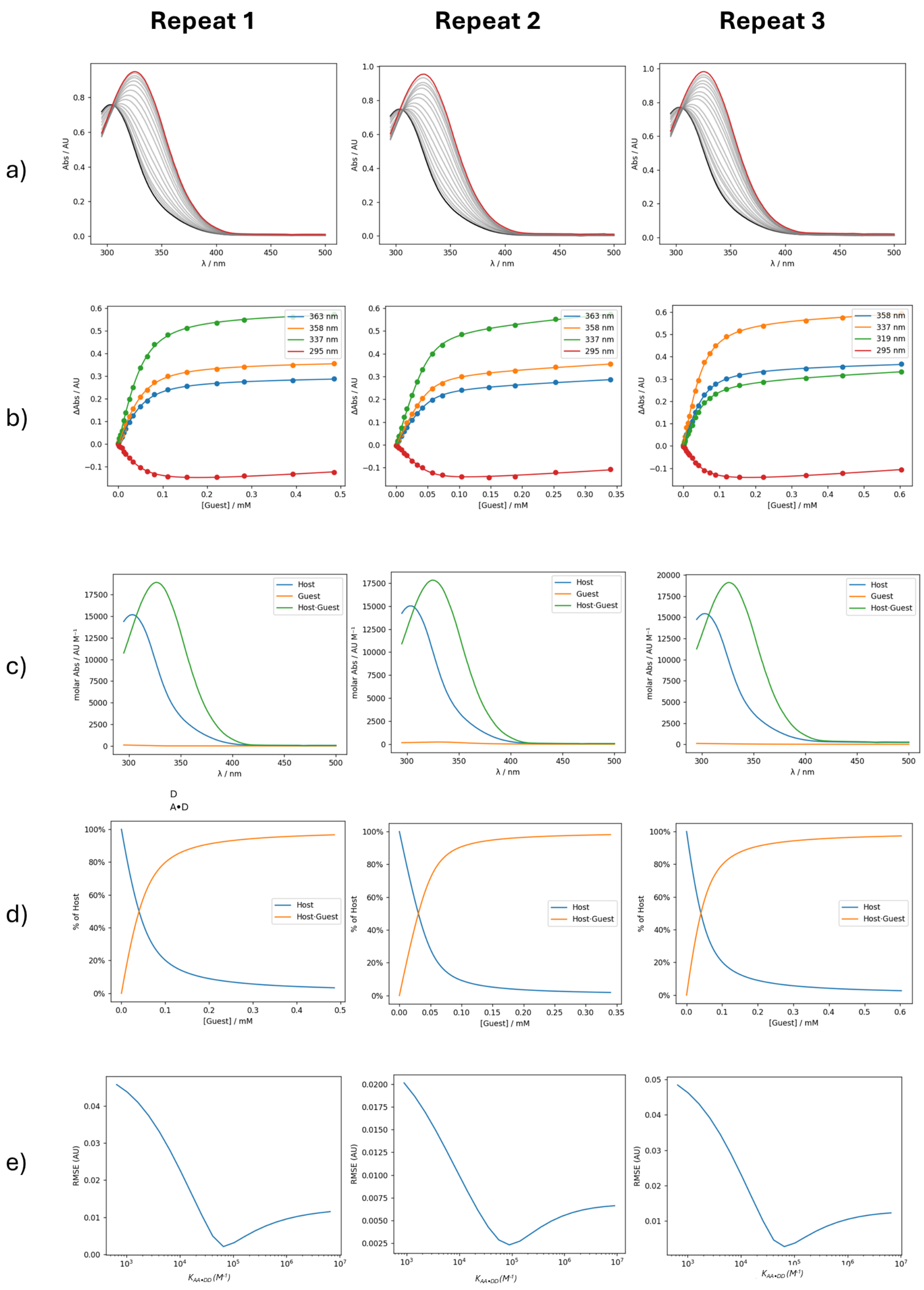


*Figure S 84 UV-Vis absorption titration of **AA** into **DD** (50 µM) in dichloromethane at 298 K. a) UV-Vis absorption spectra showing free **DD** in black and final spectrum in red. b) Fitted spectra for **DD** (host) and **AA•DD** (host•guest) c) Best fit of the change in UV-Vis absorbance at selected wavelengths to a 1:1 binding isotherm allowing for guest absorption. d) Calculated populations of different species containing **DD**. e) Relationship between the RMSE between the experimental data and calculated spectra plotted as a function of the value of* $K_{AA•DD}$.

# AOA•DOD

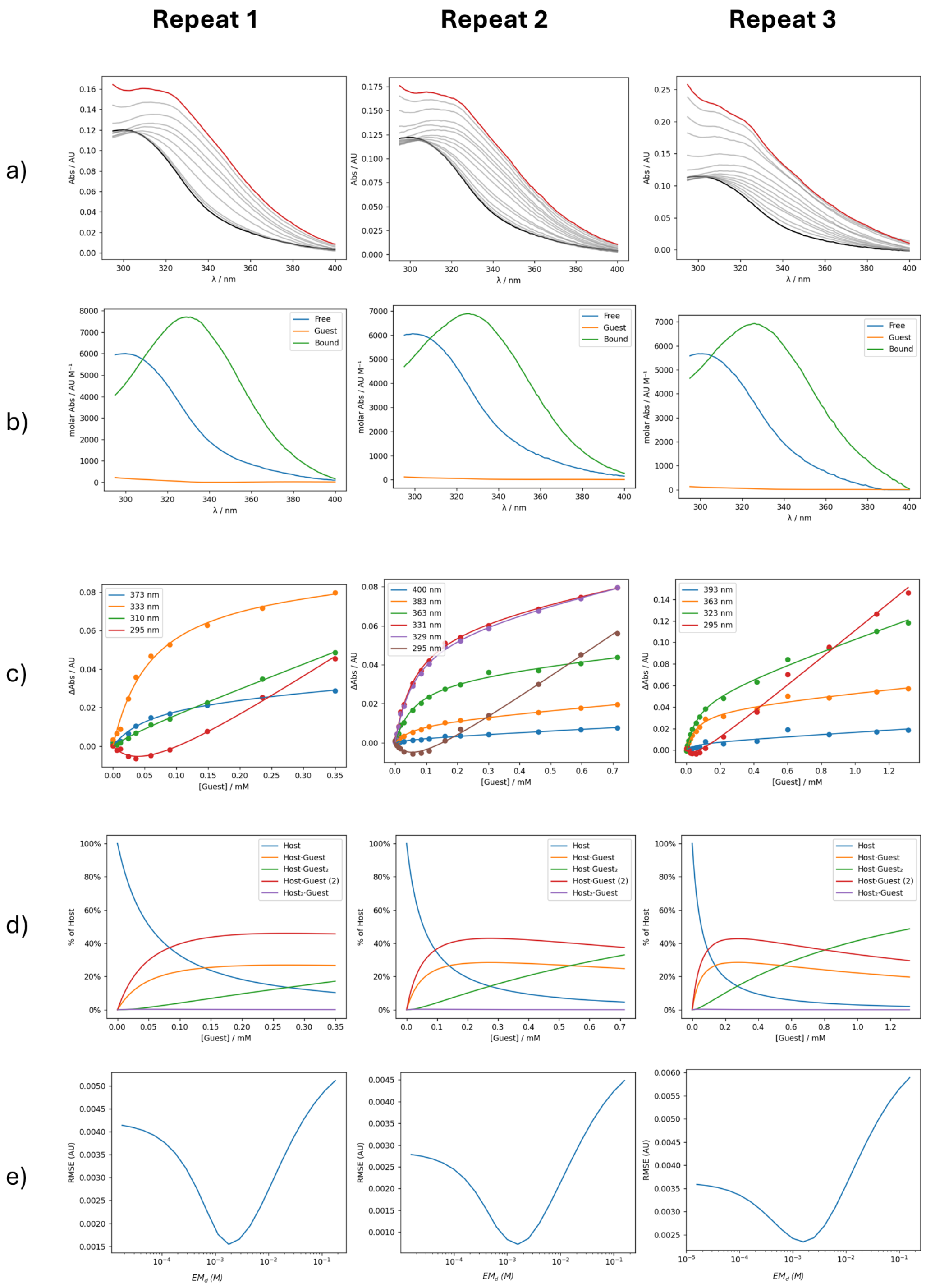


*Figure S 85 UV-Vis absorption titration of **AOA** into **DOD** (20 µM) in dichloromethane at 298 K. a) UV-Vis absorption spectra showing free **DOD** in black and final spectrum in red. b) Fitted spectra for **DOD** (host) and **AOA•DOD** (host•guest) c) Best fit of the change in UV-Vis absorbance at selected wavelengths to a 1:1 binding isotherm allowing for guest absorption. d) Calculated populations of different species containing **DOD**. e) Relationship between the RMSE between the experimental data and calculated spectra plotted as a function of the value of EM$_d$.*

# AO$_n$D•PFTB fitted to 1:1 binding isotherm

## AD•PFTB fitted to 1:1 binding isotherm

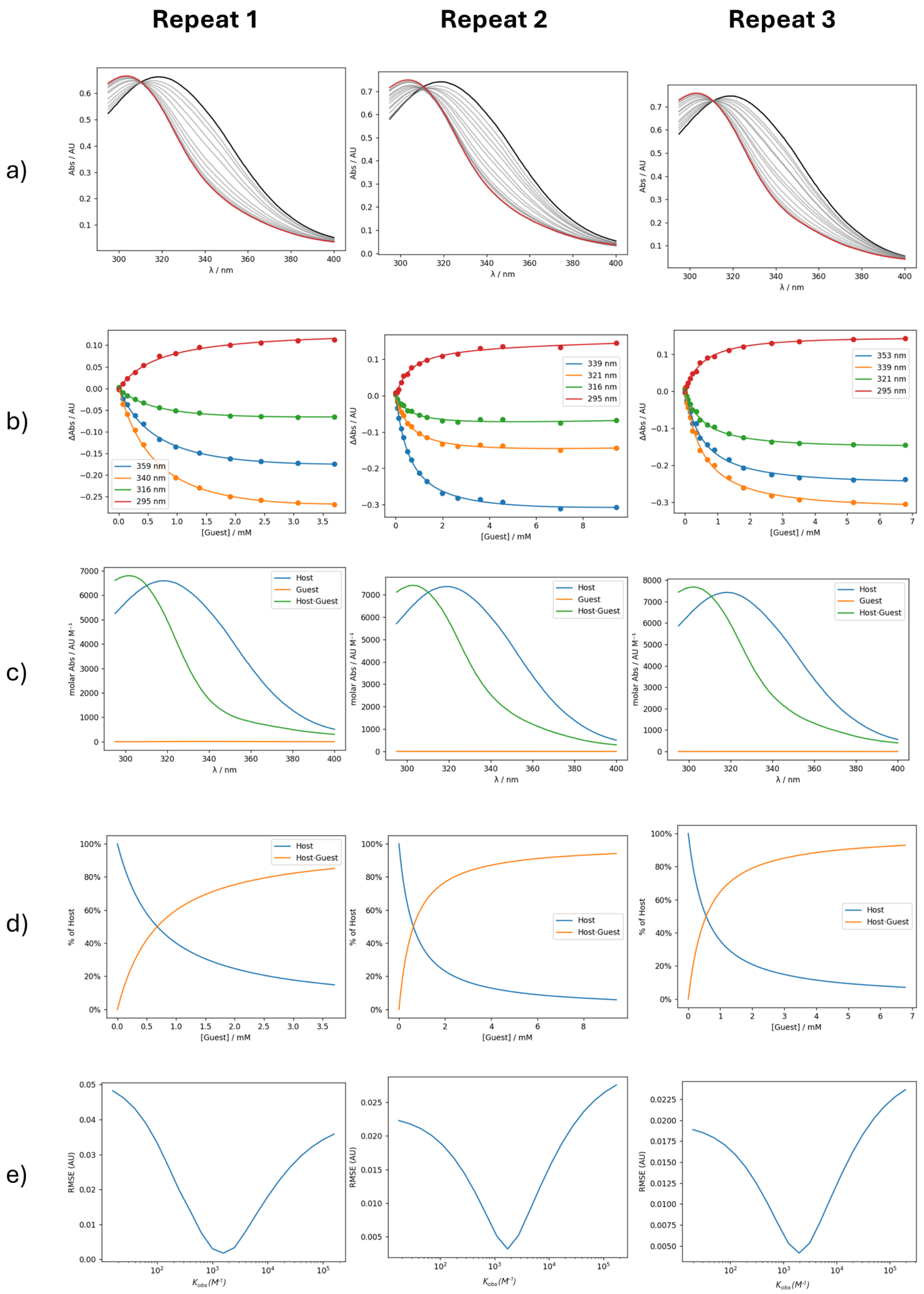


*Figure S 86 UV-Vis absorption titration of PFTB into **AD** (100 µM) in dichloromethane at 298 K. a) UV-Vis absorption spectra showing free **AD** in black and final spectrum in red. b) Fitted spectra for **AD** (host), **AD**•PFTB (host•guest) an PFTB (guest) c) Best fit of the change in UV-Vis absorbance at selected wavelengths to a 1:1 binding isotherm allowing for guest absorption. d) Calculated populations of different species containing **AD** and guest. e) Relationship between the RMSE between the experimental data and calculated spectra plotted as a function of the value of $K_{obs}$.*

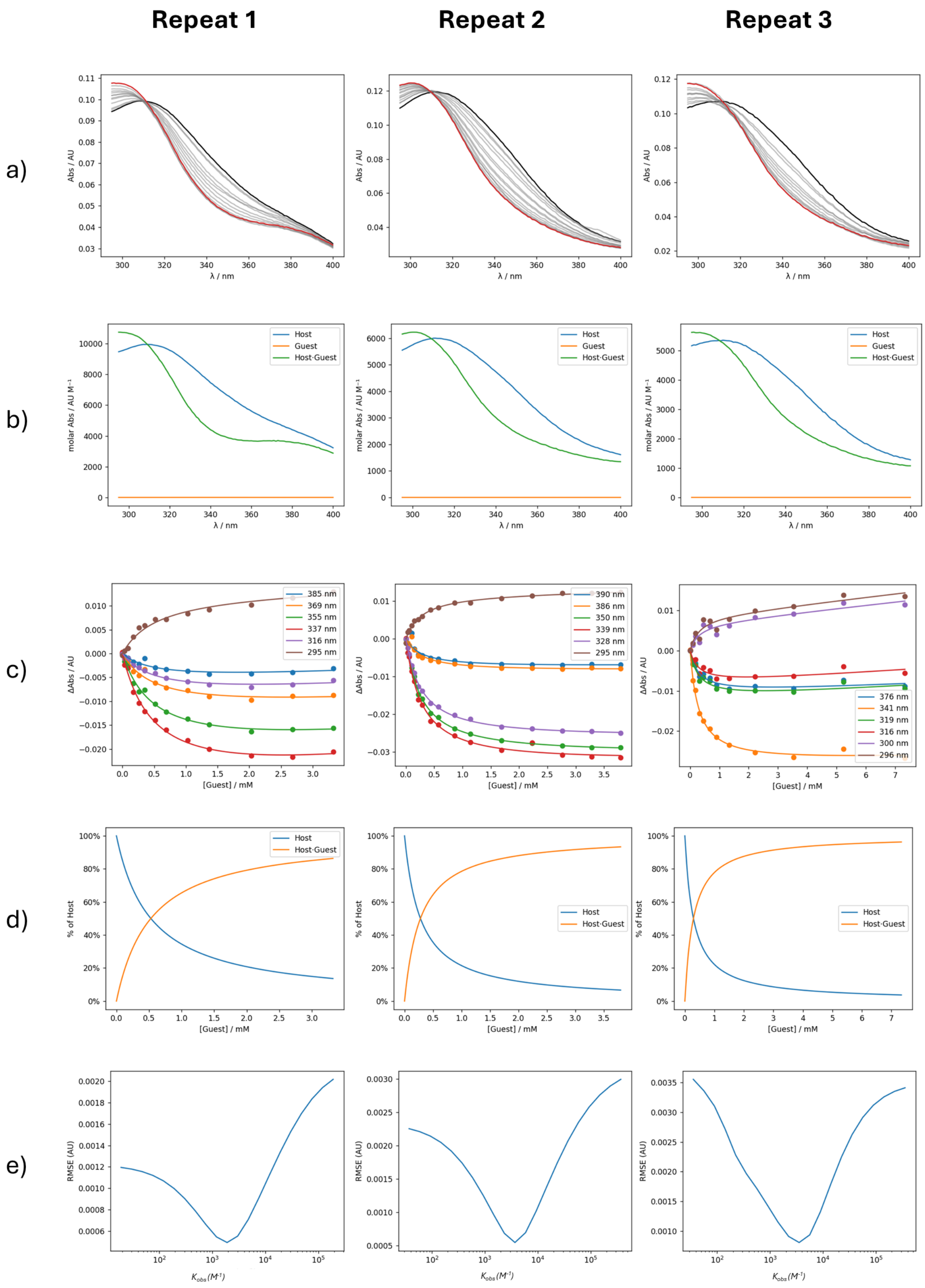


*Figure S 87 UV-Vis absorption titration of PFTB into **AD** (10 µM) in dichloromethane at 298 K. a) UV-Vis absorption spectra showing free **AD** in black and final spectrum in red. b) Fitted spectra for **AD** (host), **AD**•PFTB (host•guest) an PFTB (guest) c) Best fit of the change in UV-Vis absorbance at selected wavelengths to a 1:1 binding isotherm allowing for guest absorption. d) Calculated populations of different species containing **AD** and guest. e) Relationship between the RMSE between the experimental data and calculated spectra plotted as a function of the value of $K_{obs}$.*

# **AOD**•PFTB fitted to 1:1 binding isotherm

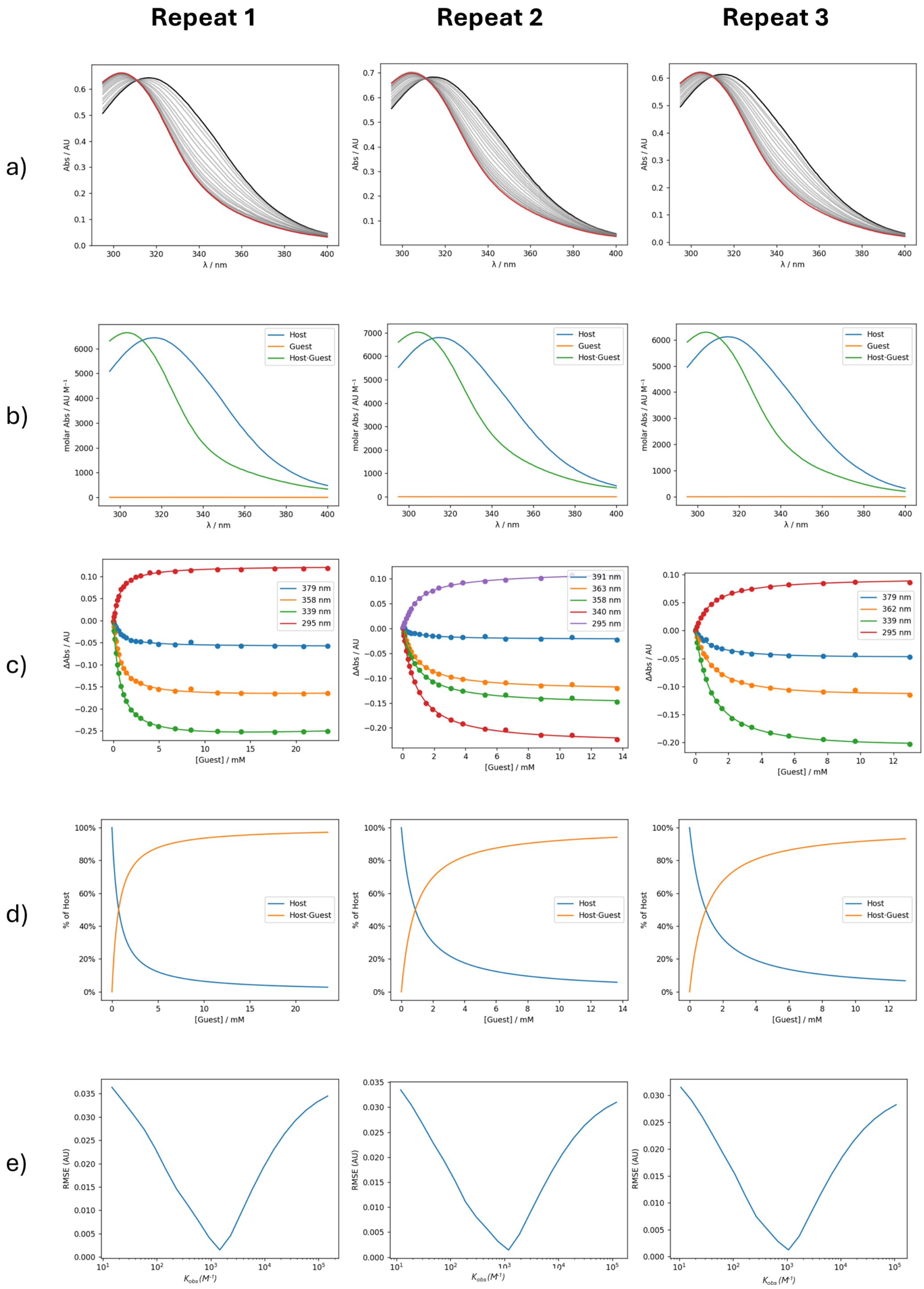


*Figure S 88 UV-Vis absorption titration of PFTB into **AOD** (100 μM) in dichloromethane at 298 K. a) UV-Vis absorption spectra showing free **AOD** in black and final spectrum in red. b) Fitted spectra for **AOD** (host), **AOD**•PFTB (host•guest) an PFTB (guest) c) Best fit of the change in UV-Vis absorbance at selected wavelengths to a 1:1 binding isotherm allowing for guest absorption. d) Calculated populations of different species containing **AOD** and guest. e) Relationship between the RMSE between the experimental data and calculated spectra plotted as a function of the value of $K_{obs}$.*

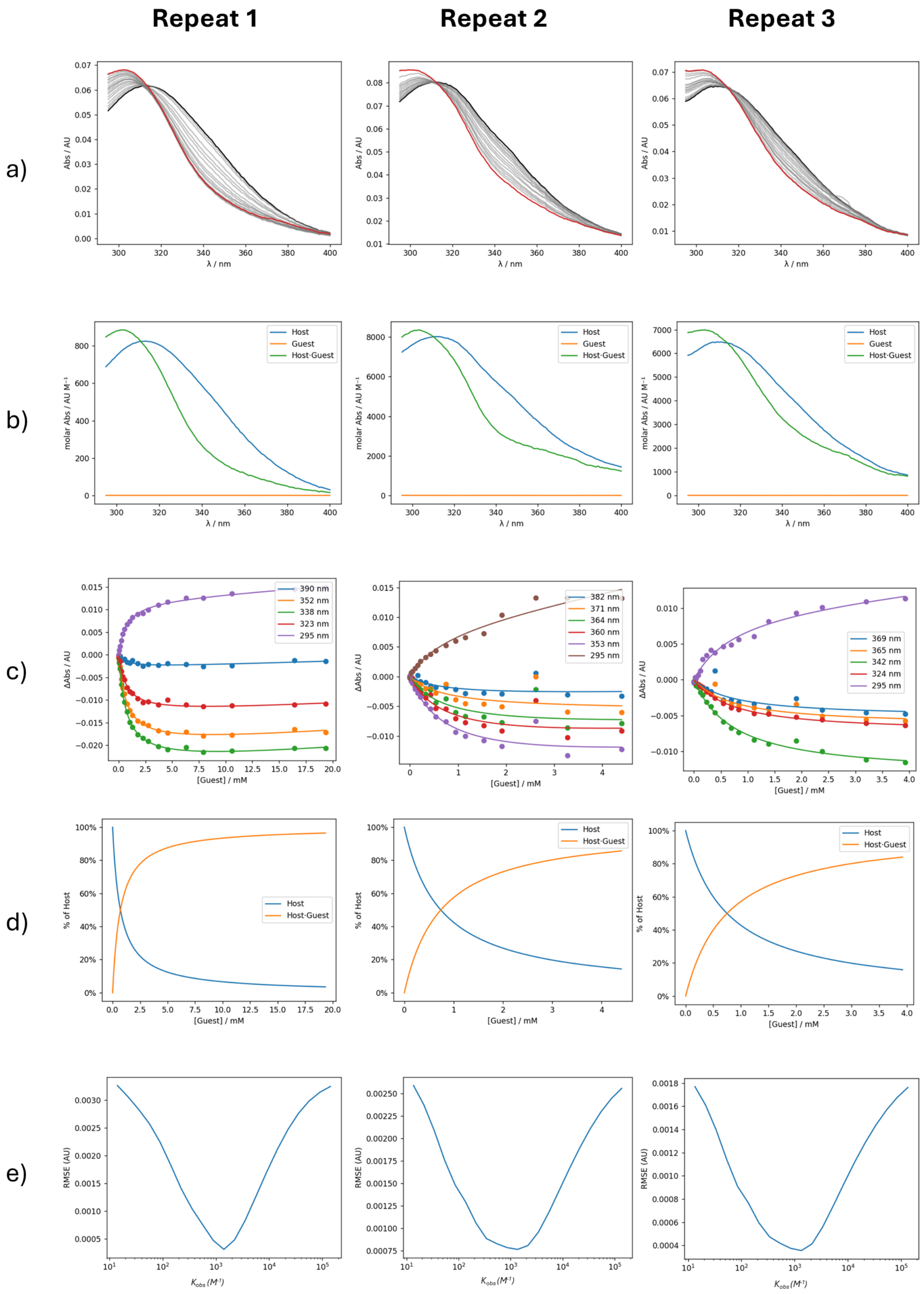


*Figure S 89 UV-Vis absorption titration of PFTB into **AOD** (10 µM) in dichloromethane at 298 K. a) UV-Vis absorption spectra showing free **AOD** in black and final spectrum in red. b) Fitted spectra for **AOD** (host), **AOD**•PFTB (host•guest) an PFTB (guest) c) Best fit of the change in UV-Vis absorbance at selected wavelengths to a 1:1 binding isotherm allowing for guest absorption. d) Calculated populations of different species containing **AOD** and guest. e) Relationship between the RMSE between the experimental data and calculated spectra plotted as a function of the value of $K_{obs}$.*

# **AOOD**•PFTB fitted to 1:1 binding isotherm

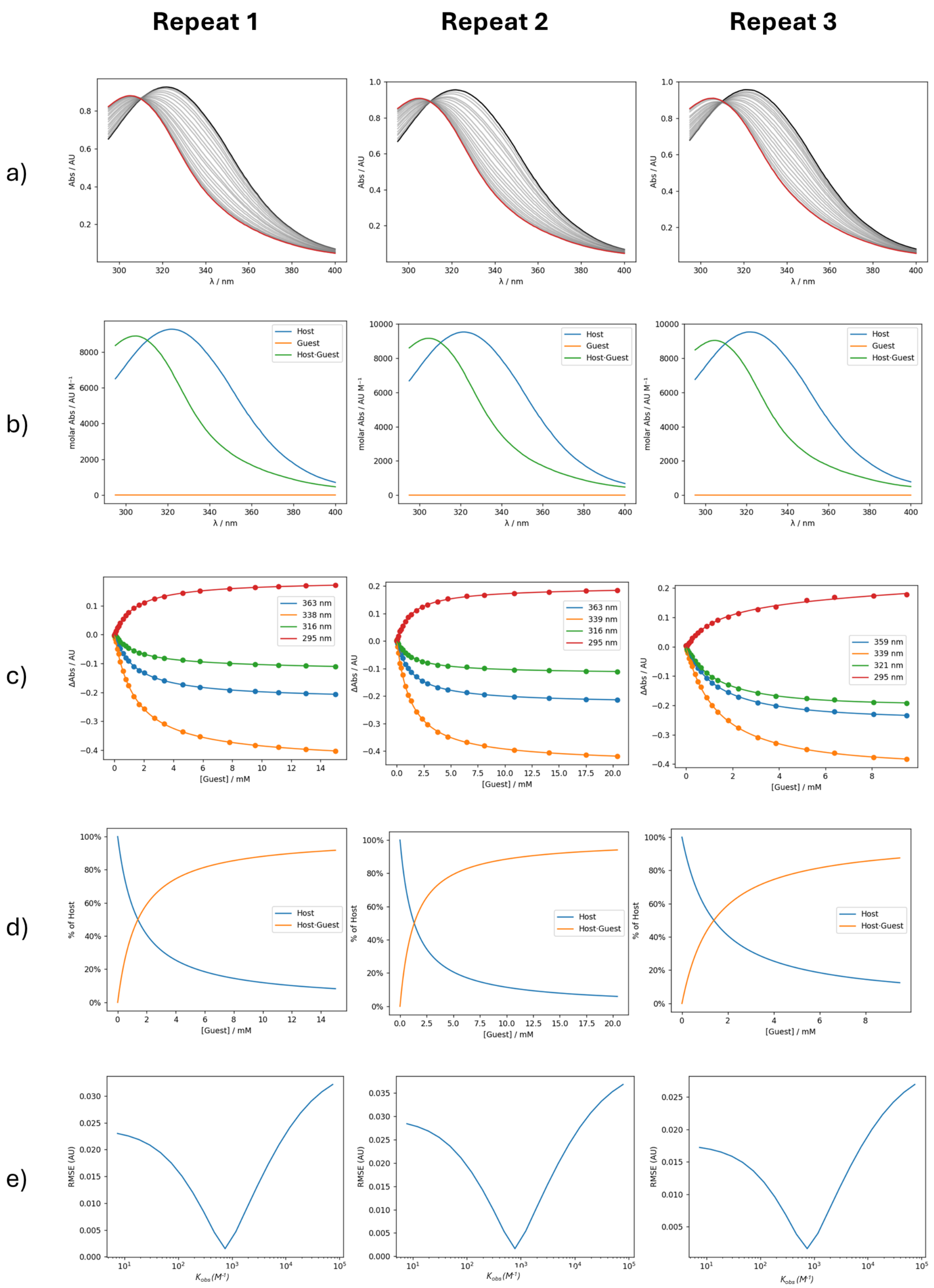


*Figure S 90 UV-Vis absorption titration of PFTB into **AOOD** (100 µM) in dichloromethane at 298 K. a) UV-Vis absorption spectra showing free **AOOD** in black and final spectrum in red. b) Fitted spectra for **AOOD** (host), **AOOD**•PFTB (host•guest) an PFTB (guest) c) Best fit of the change in UV-Vis absorbance at selected wavelengths to a 1:1 binding isotherm allowing for guest absorption. d) Calculated populations of different species containing **AOOD** and guest. e) Relationship between the RMSE between the experimental data and calculated spectra plotted as a function of the value of* $K_{obs}$.

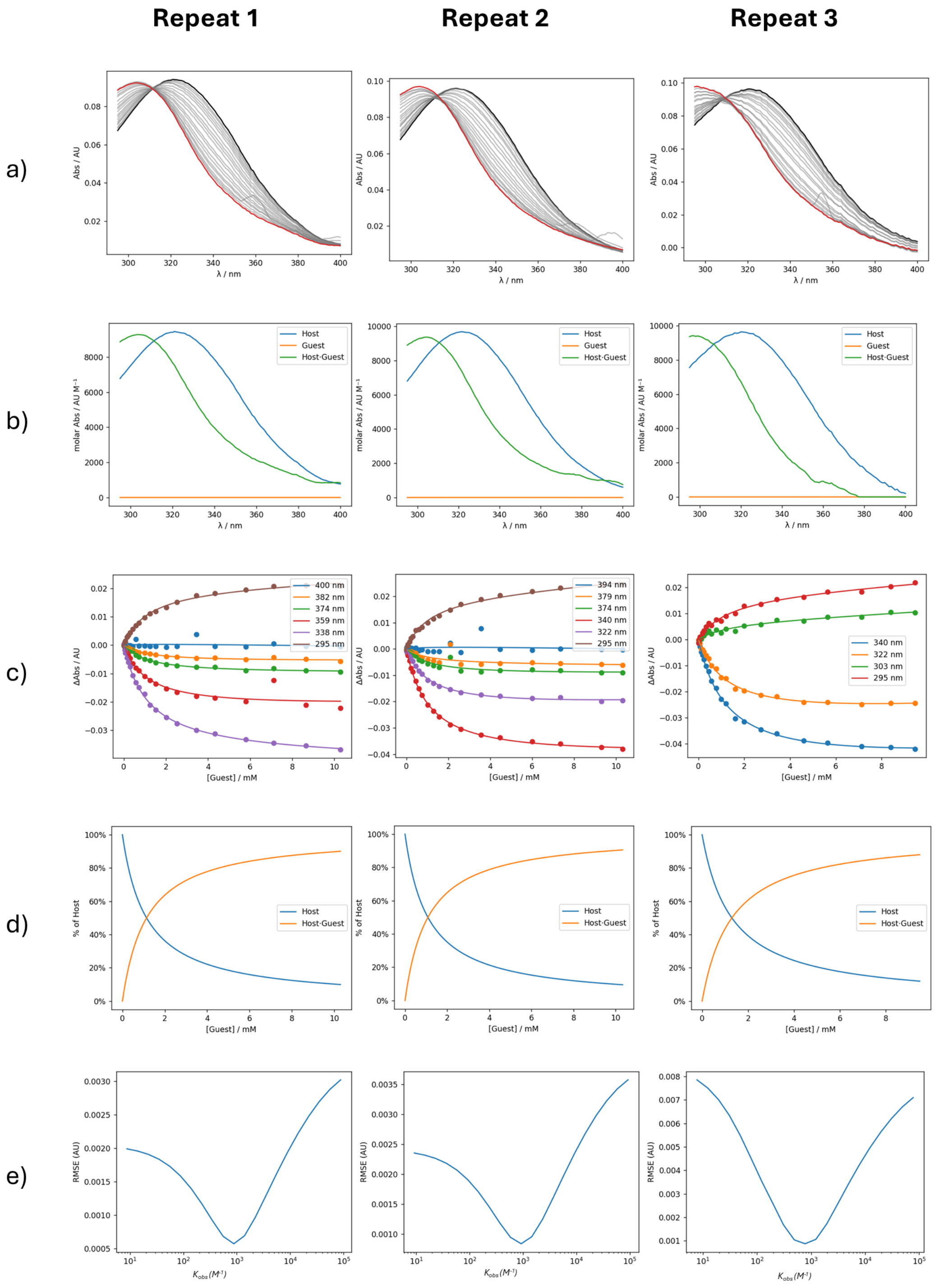


*Figure S 91 UV-Vis absorption titration of PFTB into **AOOD** (10 µM) in dichloromethane at 298 K. a) UV-Vis absorption spectra showing free **AOOD** in black and final spectrum in red. b) Fitted spectra for **AOOD** (host), **AOOD**•PFTB (host•guest) an PFTB (guest) c) Best fit of the change in UV-Vis absorbance at selected wavelengths to a 1:1 binding isotherm allowing for guest absorption d) Calculated populations of different species containing **AOOD** and guest. e) Relationship between the RMSE between the experimental data and calculated spectra plotted as a function of the value of $K_{obs}$.*

# **AOOOD**•PFTB fitted to 1:1 binding isotherm

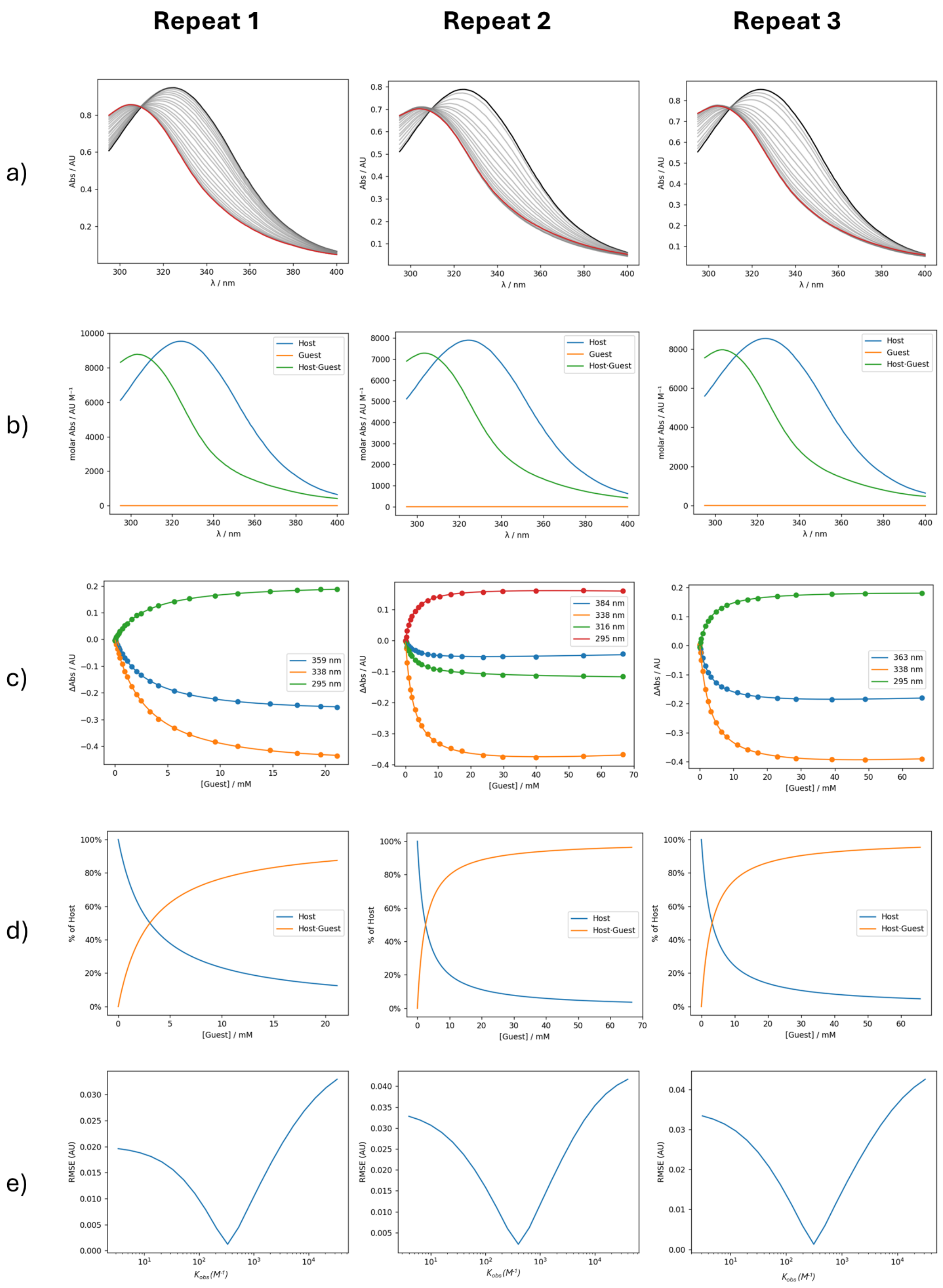


*Figure S 92 UV-Vis absorption titration of PFTB into* ***AOOOD*** *(100 µM) in dichloromethane at 298 K. a) UV-Vis absorption spectra showing free* ***AOOOD*** *in black and final spectrum in red. b) Fitted spectra for* ***AOOOD*** *(host),* ***AOOOD•****PFTB (host•guest) an PFTB (guest) c) Best fit of the change in UV-Vis absorbance at selected wavelengths to a 1:1 binding isotherm allowing for guest absorption. d) Calculated populations of different species containing* ***AOOOD*** *and guest. e) Relationship between the RMSE between the experimental data and calculated spectra plotted as a function of the value of* $K_{obs}$.

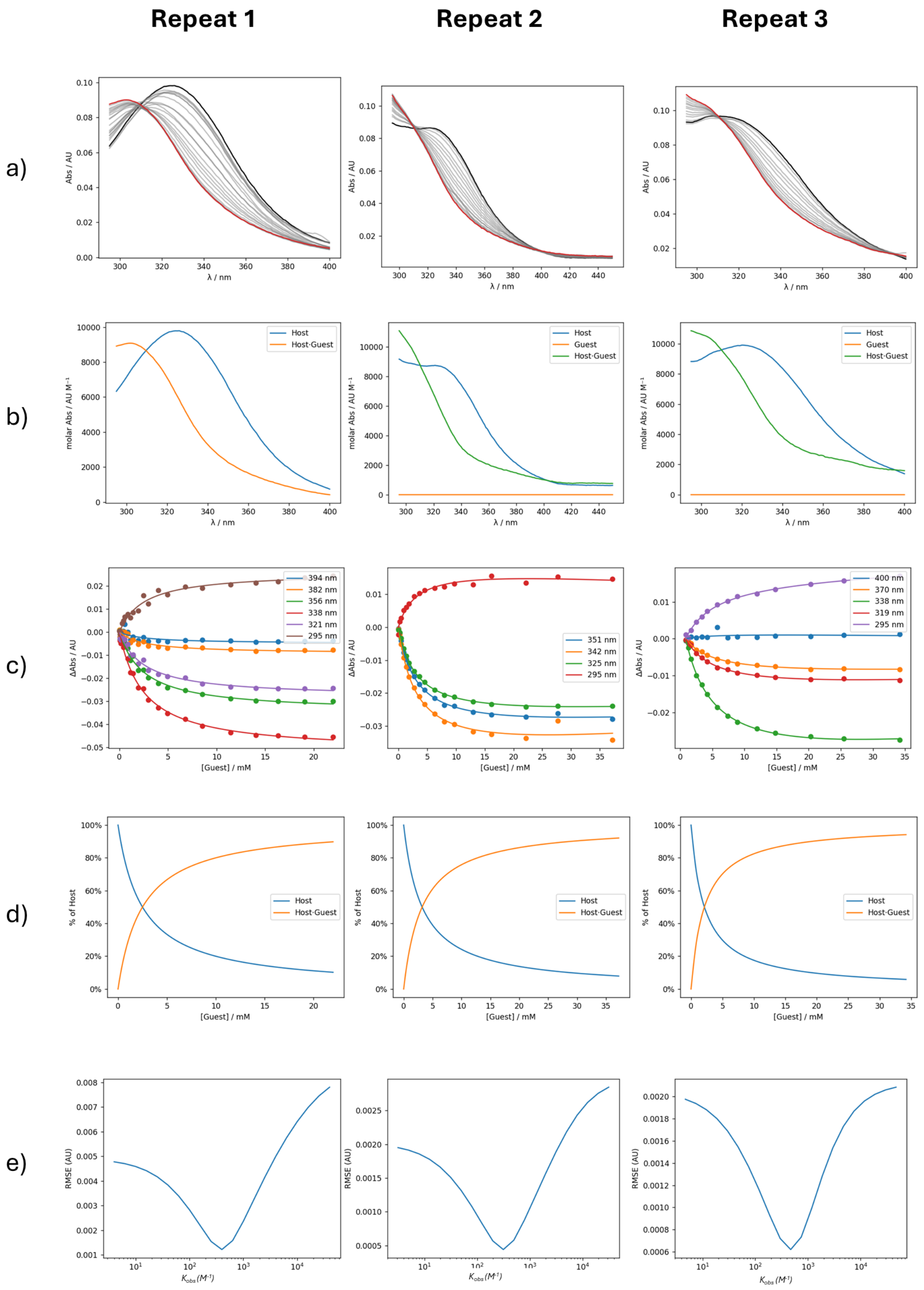


*Figure S 93 UV-Vis absorption titration of PFTB into **AOOOD** (10 µM) in dichloromethane at 298 K. a) UV-Vis absorption spectra showing free **AOOOD** in black and final spectrum in red. b) Fitted spectra for **AOOOD** (host), **AOOOD•**PFTB (host•guest) an PFTB (guest) c) Best fit of the change in UV-Vis absorbance at selected wavelengths to a 1:1 binding isotherm allowing for guest absorption. d) Calculated populations of different species containing **AOOOD** and guest. e) Relationship between the RMSE between the experimental data and calculated spectra plotted as a function of the value of* $K_{obs}$.

# **AOOOOD**•PFTB fitted to 1:1 binding isotherm

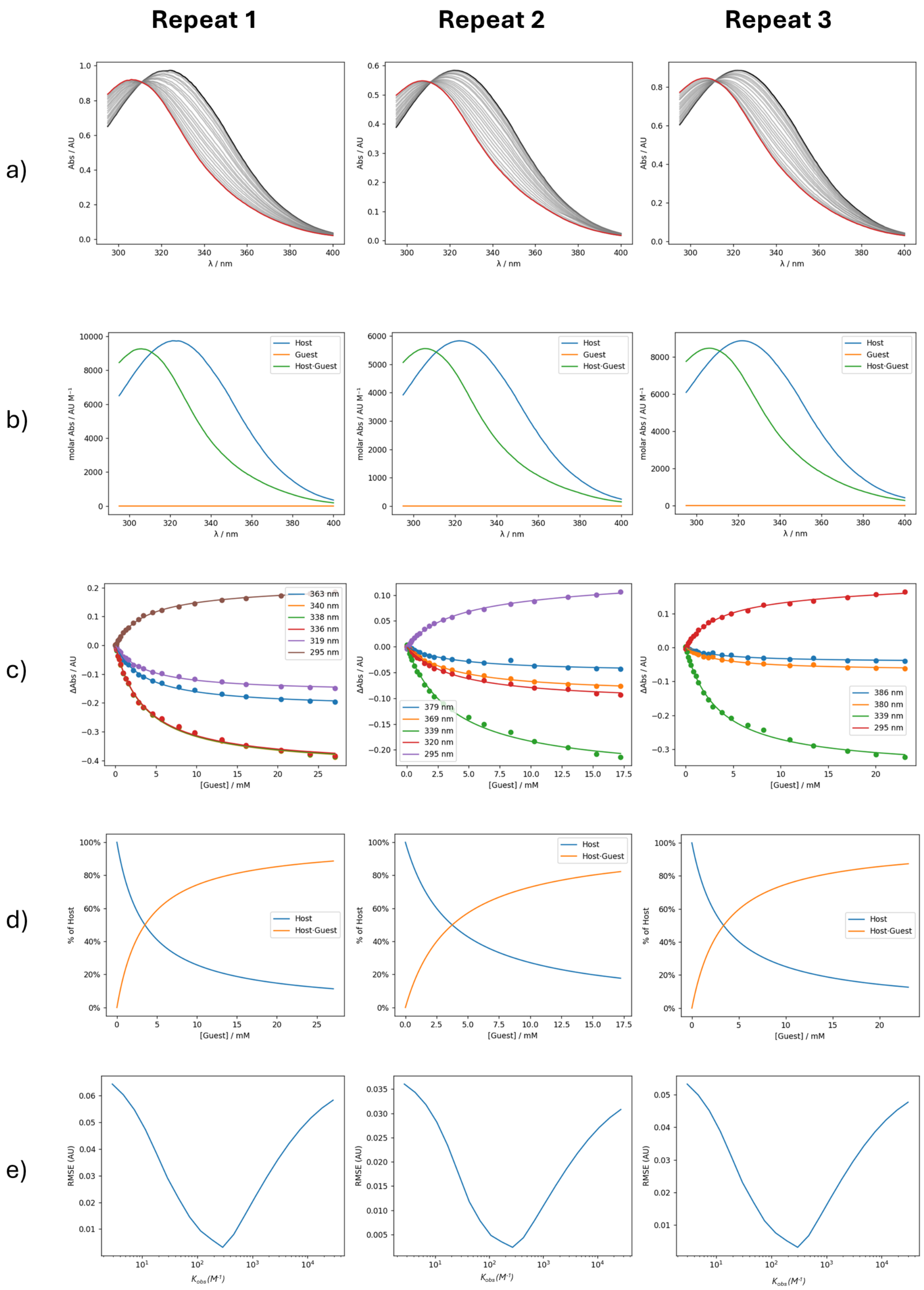


*Figure S 94 UV-Vis absorption titration of PFTB into **AOOOOD** (100 µM) in dichloromethane at 298 K. a) UV-Vis absorption spectra showing free **AOOOOD** in black and final spectrum in red. b) Fitted spectra for **AOOOOD** (host), **AOOOOD**•PFTB (host•guest) an PFTB (guest) c) Best fit of the change in UV-Vis absorbance at selected wavelengths to a 1:1 binding isotherm allowing for guest absorption. d) Calculated populations of different species containing **AOOOOD** and guest. e) Relationship between the RMSE between the experimental data and calculated spectra plotted as a function of the value of $K_{obs}$.*

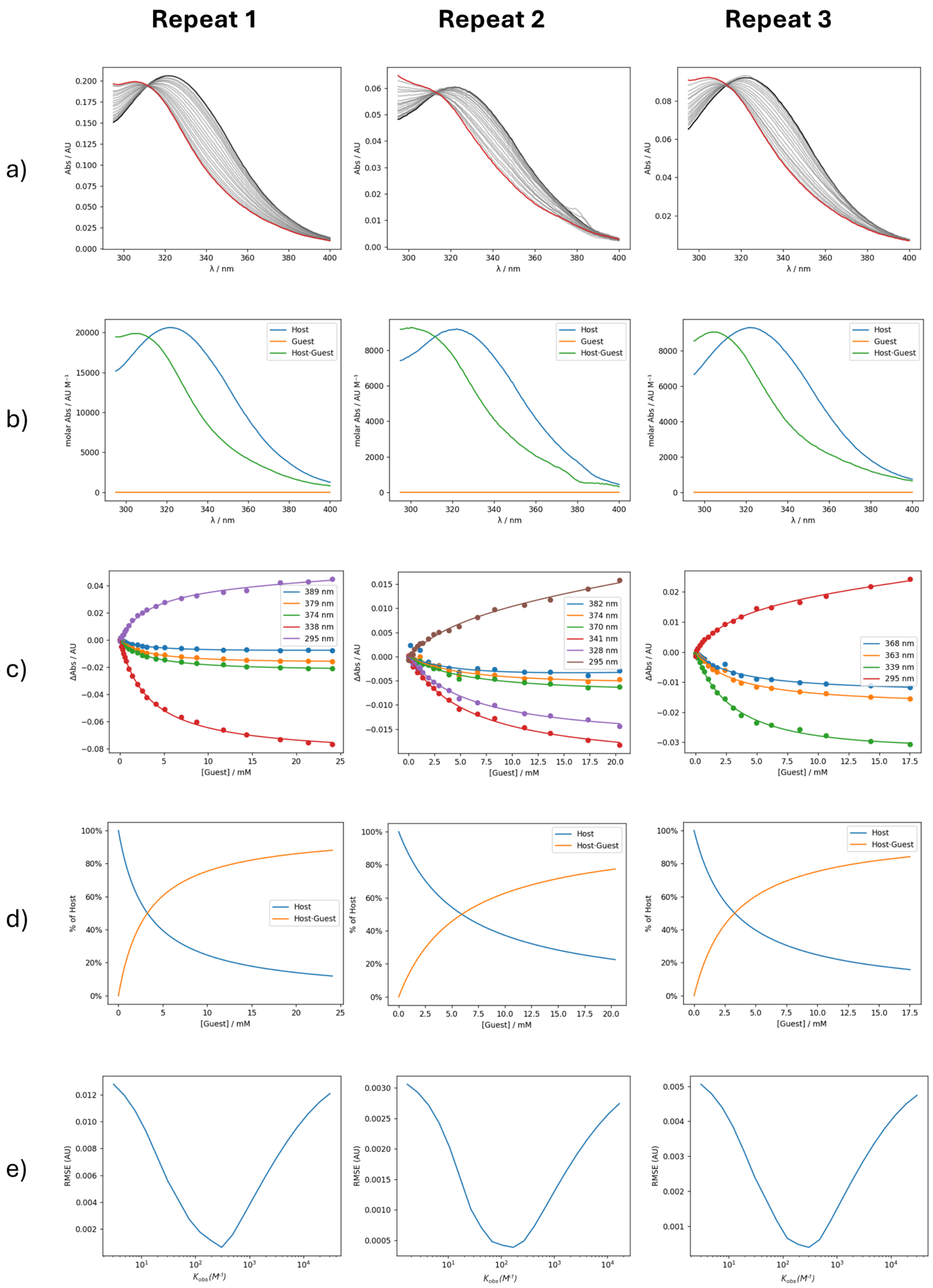


*Figure S 95 UV-Vis absorption titration of PFTB into* ***AOOOOD*** *(10 µM) in dichloromethane at 298 K. a) UV-Vis absorption spectra showing free* ***AOOOOD*** *in black and final spectrum in red. b) Fitted spectra for* ***AOOOOD*** *(host),* ***AOOOOD•****PFTB (host•guest) an PFTB (guest) c) Best fit of the change in UV-Vis absorbance at selected wavelengths to a 1:1 binding isotherm allowing for guest absorption. d) Calculated populations of different species containing* ***AOOOOD*** *and guest. e) Relationship between the RMSE between the experimental data and calculated spectra plotted as a function of the value of* $K_{obs}$.

# **AO$_5$D**•PFTB fitted to 1:1 binding isotherm

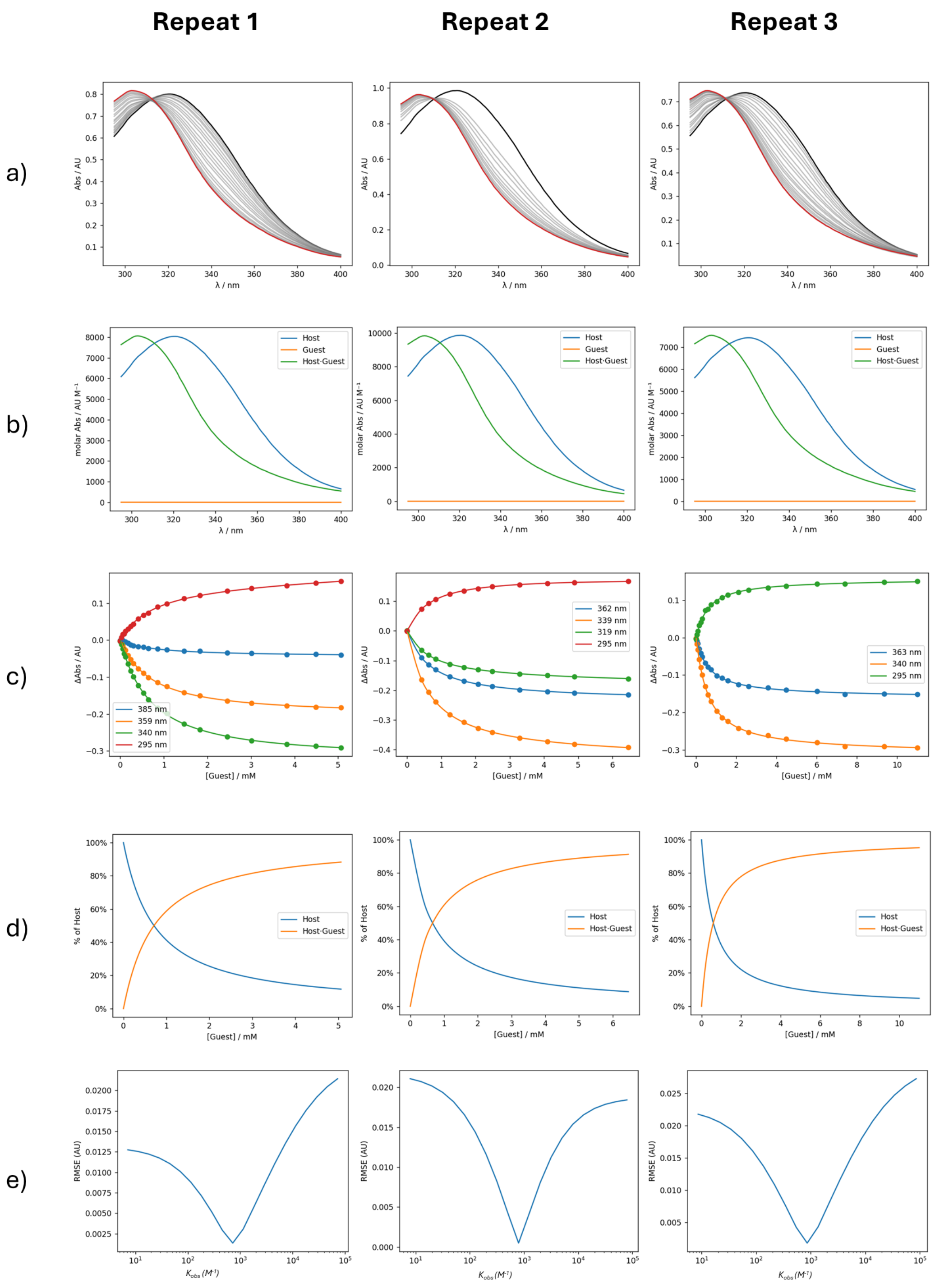


*Figure S 96 UV-Vis absorption titration of PFTB into* ***AO$_5$D*** *(100 µM) in dichloromethane at 298 K. a) UV-Vis absorption spectra showing free* ***AO$_5$D*** *in black and final spectrum in red. b) Fitted spectra for* ***AO$_5$D*** *(host),* ***AO$_5$D*** *•PFTB (host•guest) an PFTB (guest) c) Best fit of the change in UV-Vis absorbance at selected wavelengths to a 1:1 binding isotherm allowing for guest absorption. d) Calculated populations of different species containing* ***AO$_5$D*** *and guest. e) Relationship between the RMSE between the experimental data and calculated spectra plotted as a function of the value of* $K_{obs}$.

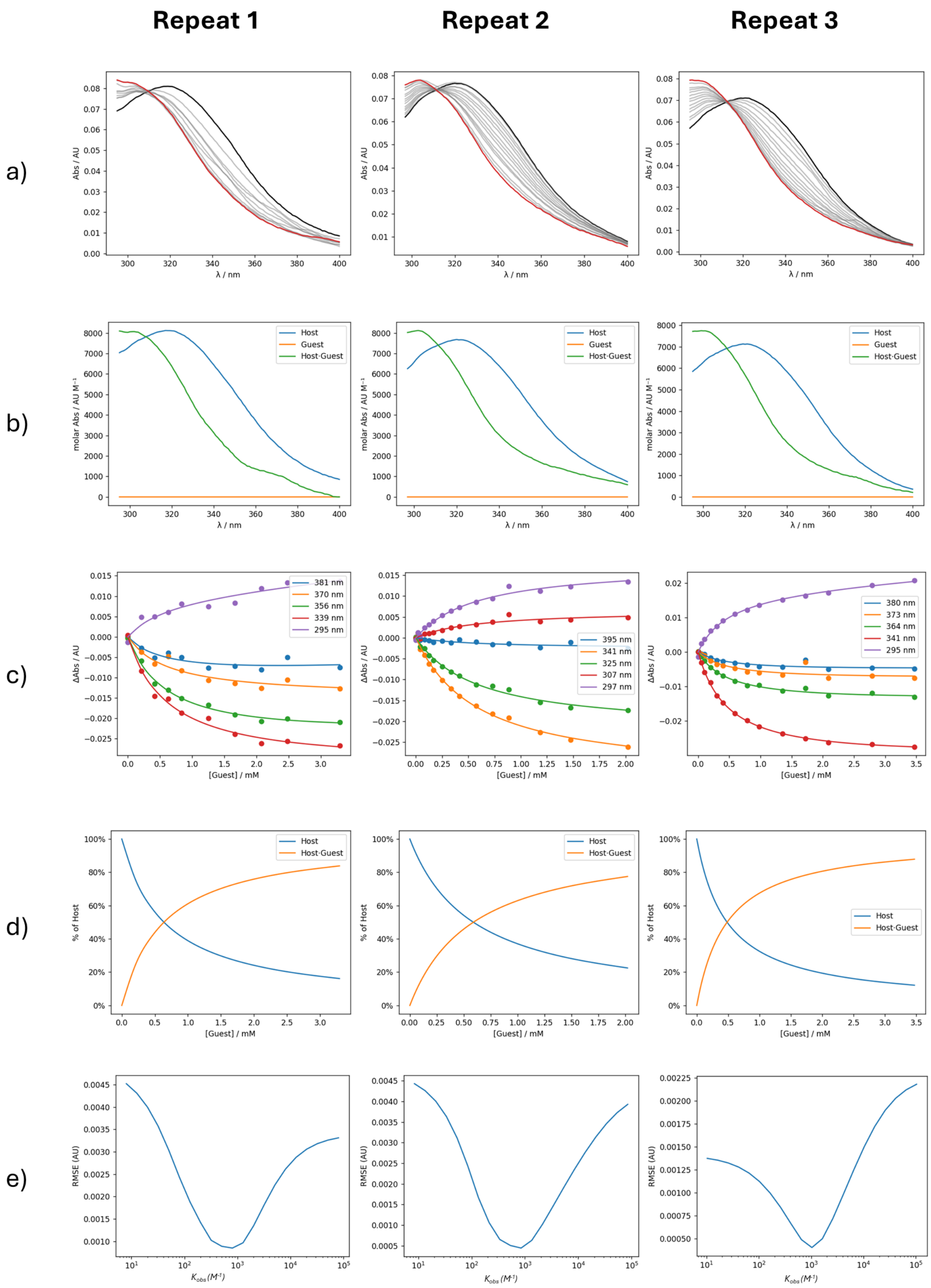


*Figure S 97 UV-Vis absorption titration of PFTB into* ***AO₅D*** *(10 µM) in dichloromethane at 298 K. a) UV-Vis absorption spectra showing free* ***AO₅D*** *in black and final spectrum in red. b) Fitted spectra for* ***AO₅D*** *(host),* ***AO₅D*** *•PFTB (host•guest) an PFTB (guest) c) Best fit of the change in UV-Vis absorbance at selected wavelengths to a 1:1 binding isotherm allowing for guest absorption. d) Calculated populations of different species containing* ***AO₅D*** *and guest. e) Relationship between the RMSE between the experimental data and calculated spectra plotted as a function of the value of* $K_{obs}$.

# $\mathbf{AO_6D}$•PFTB fitted to 1:1 binding isotherm

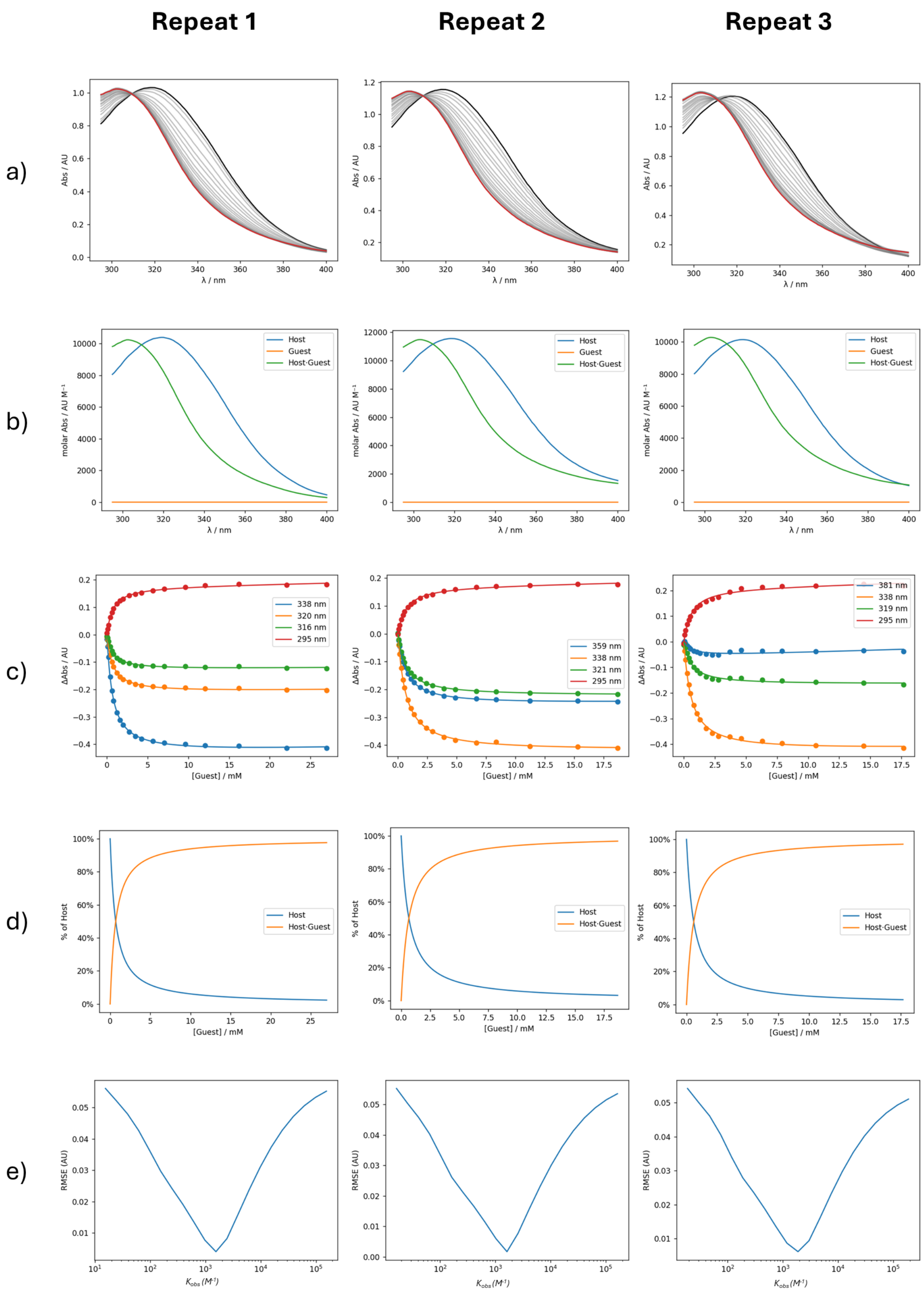


*Figure S 98 UV-Vis absorption titration of PFTB into* ***AO₆D*** *(100 µM) in dichloromethane at 298 K. a) UV-Vis absorption spectra showing free* ***AO₆D*** *in black and final spectrum in red. b) Fitted spectra for* ***AO₆D*** *(host),* ***AO₆D*** *•PFTB (host•guest) an PFTB (guest) c) Best fit of the change in UV-Vis absorbance at selected wavelengths to a 1:1 binding isotherm allowing for guest absorption. d)Calculated populations of different species containing* ***AO₆D*** *and guest. e) Relationship between the RMSE between the experimental data and calculated spectra plotted as a function of the value of* $K_{obs}$.

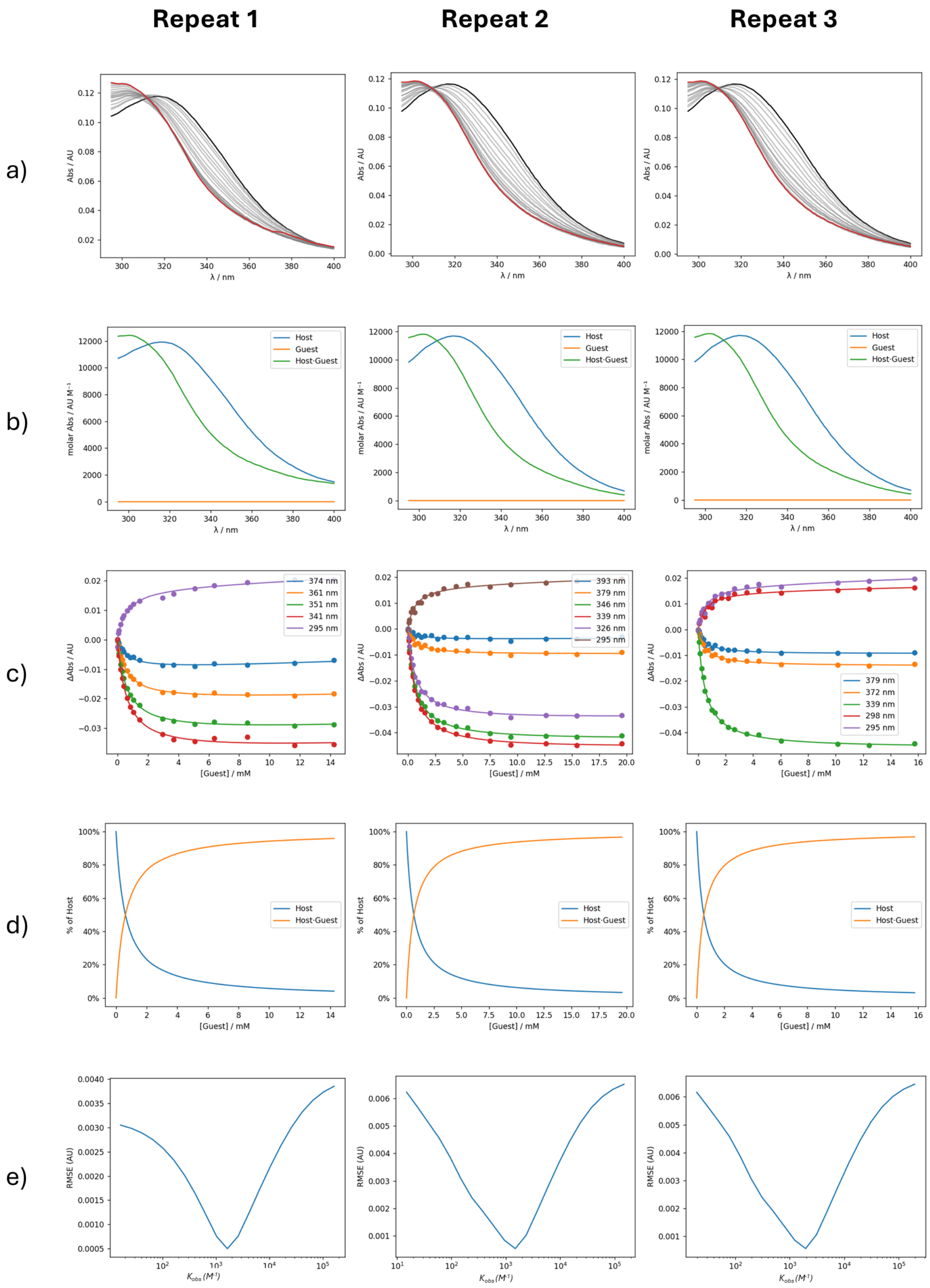


*Figure S 99 UV-Vis absorption titration of PFTB into **$AO_6D$** (10 µM) in dichloromethane at 298 K. a) UV-Vis absorption spectra showing free **$AO_6D$** in black and final spectrum in red. b) Fitted spectra for **$AO_6D$** (host), **$AO_6D$** •PFTB (host•guest) an PFTB (guest) c) Best fit of the change in UV-Vis absorbance at selected wavelengths to a 1:1 binding isotherm allowing for guest absorption. d) Calculated populations of different species containing **$AO_6D$** and guest. e) Relationship between the RMSE between the experimental data and calculated spectra plotted as a function of the value of $K_{obs}$.*

# $AO_7D$•PFTB fitted to 1:1 binding isotherm

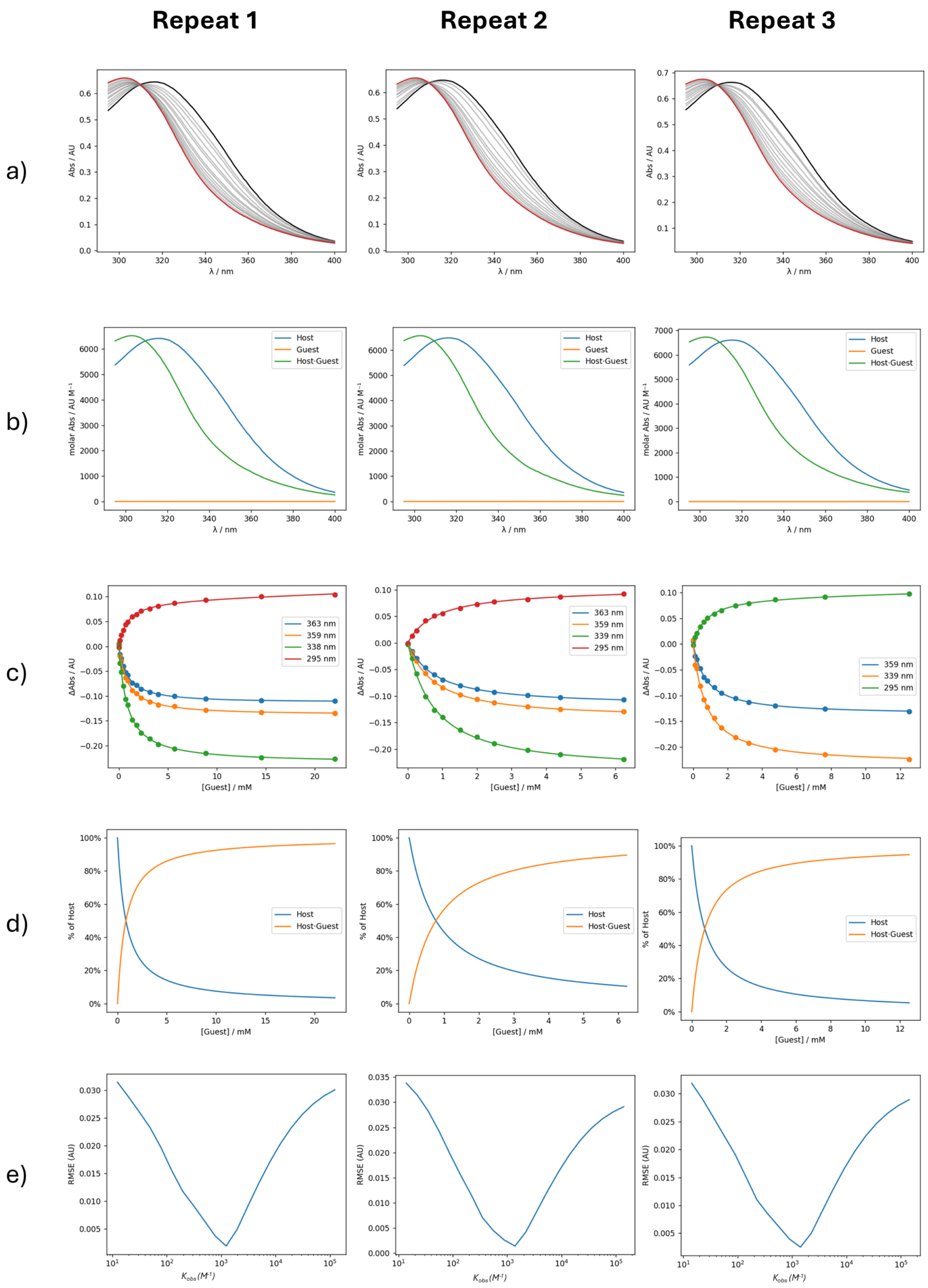


*Figure S 100 UV-Vis absorption titration of PFTB into **$AO_7D$** (100 µM) in dichloromethane at 298 K. a) UV-Vis absorption spectra showing free **$AO_7D$** in black and final spectrum in red. b) Fitted spectra for **$AO_7D$** (host), **$AO_7D$** •PFTB (host•guest) an PFTB (guest) c) Best fit of the change in UV-Vis absorbance at selected wavelengths to a 1:1 binding isotherm allowing for guest absorption. d) Calculated populations of different species containing **$AO_7D$** and guest. e) Relationship between the RMSE between the experimental data and calculated spectra plotted as a function of the value of $K_{obs}$.*

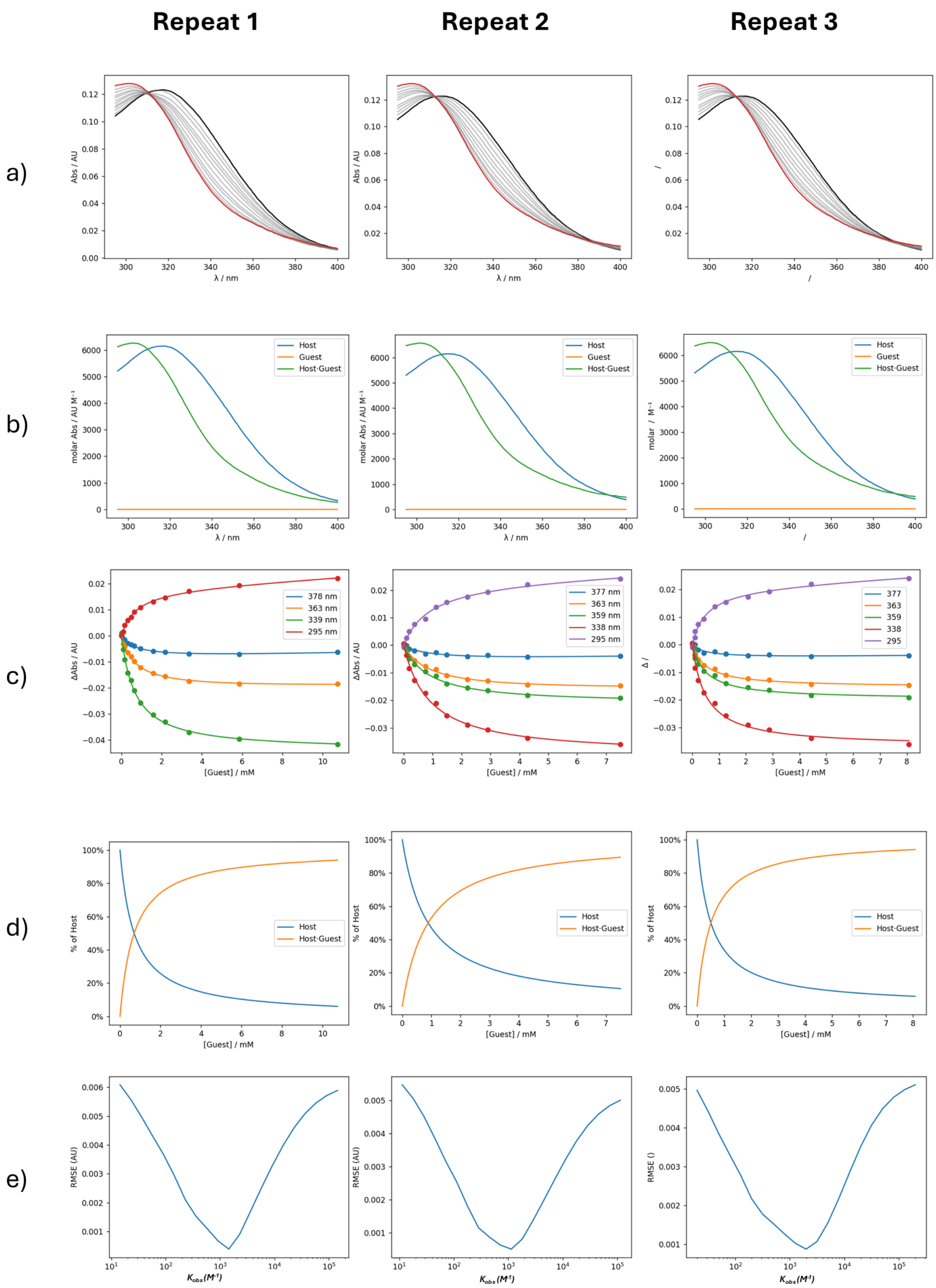


*Figure S 101 UV-Vis absorption titration of PFTB into* ***AO₇D*** *(10 µM) in dichloromethane at 298 K. a) UV-Vis absorption spectra showing free* ***AO₇D*** *in black and final spectrum in red. b) Fitted spectra for* ***AO₇D*** *(host),* ***AO₇D*** *•PFTB (host•guest) an PFTB (guest) c) Best fit of the change in UV-Vis absorbance at selected wavelengths to a 1:1 binding isotherm allowing for guest absorption. d) Calculated populations of different species containing* ***AO₇D*** *and guest. e) Relationship between the RMSE between the experimental data and calculated spectra plotted as a function of the value of* $K_{obs}$.

# **$AO_8D$**•PFTB fitted to 1:1 binding isotherm

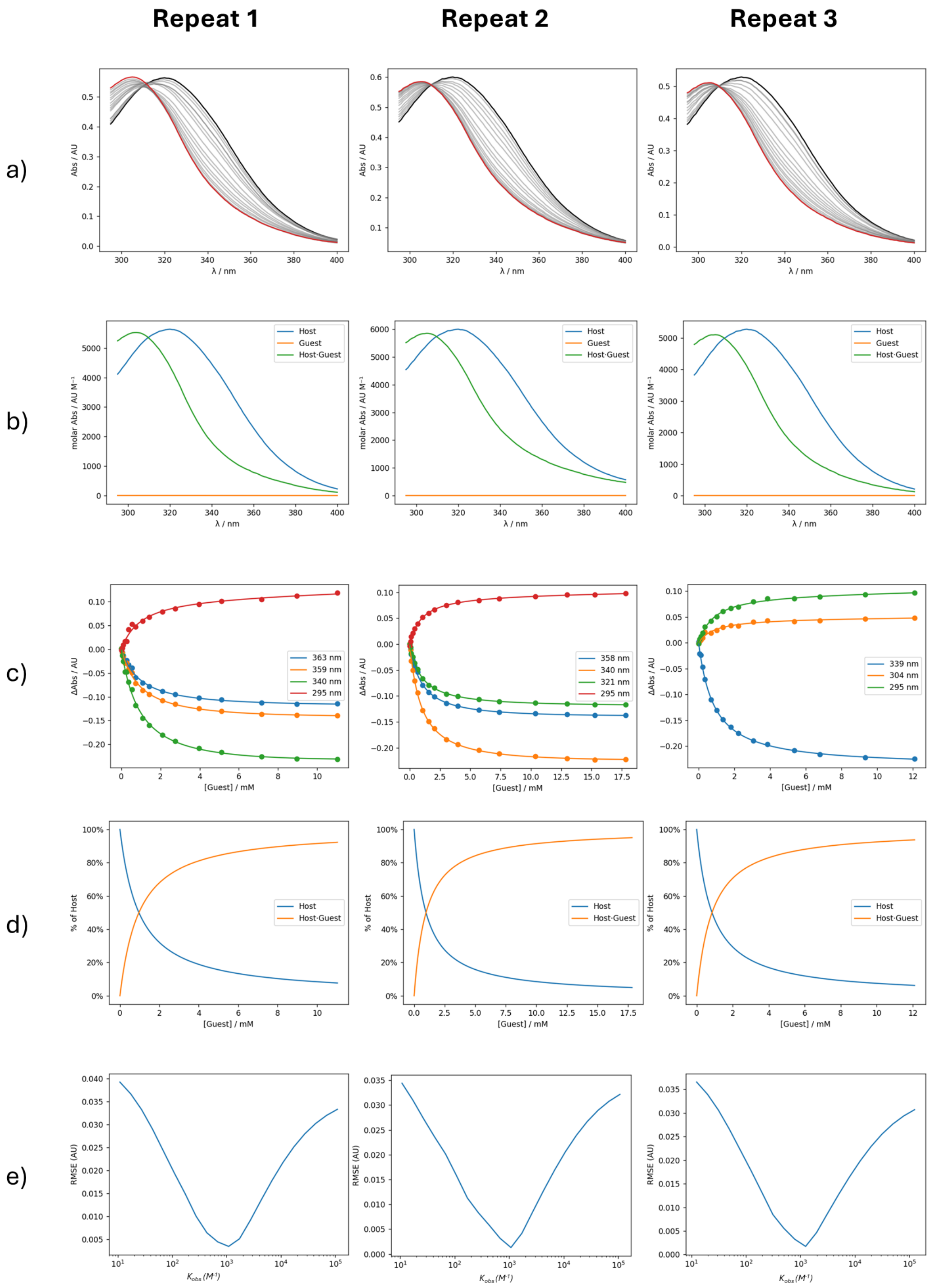


*Figure S 102 UV-Vis absorption titration of PFTB into **$AO_8D$** (100 μM) in dichloromethane at 298 K. a) UV-Vis absorption spectra showing free **$AO_8D$** vin black and final spectrum in red. b) Fitted spectra for **$AO_8D$** (host), **$AO_8D$** •PFTB (host•guest) an PFTB (guest) c) Best fit of the change in UV-Vis absorbance at selected wavelengths to a 1:1 binding isotherm allowing for guest absorption. d) Calculated populations of different species containing **$AO_8D$** and guest. e) Relationship between the RMSE between the experimental data and calculated spectra plotted as a function of the value of $K_{obs}$.*

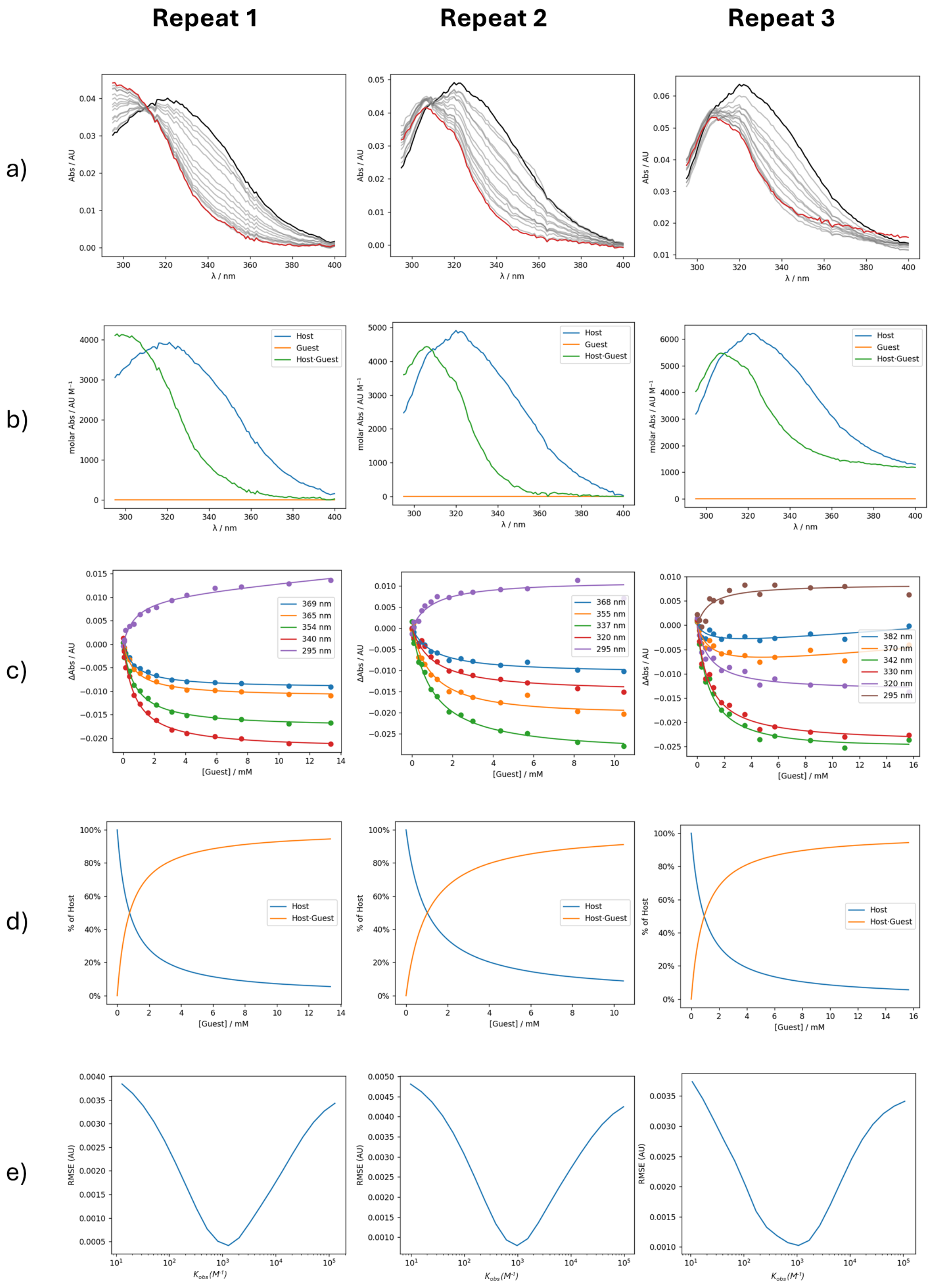


*Figure S 103 UV-Vis absorption titration of PFTB into **AO$_8$D** (10 µM) in dichloromethane at 298 K. a) UV-Vis absorption spectra showing free **AO$_8$D** vin black and final spectrum in red. b) ) Fitted spectra for **AO$_8$D** (host), **AO$_8$D** •PFTB (host•guest) an PFTB (guest) c) Best fit of the change in UV-Vis absorbance at selected wavelengths to a 1:1 binding isotherm allowing for guest absorption. d) Calculated populations of different species containing **AO$_8$D** and guest e) Relationship between the RMSE between the experimental data and calculated spectra plotted as a function of the value of $K_{obs}$.*

# $AO_nD$•PFTB fitted to complex models

## AD•PFTB fitted to duplex model

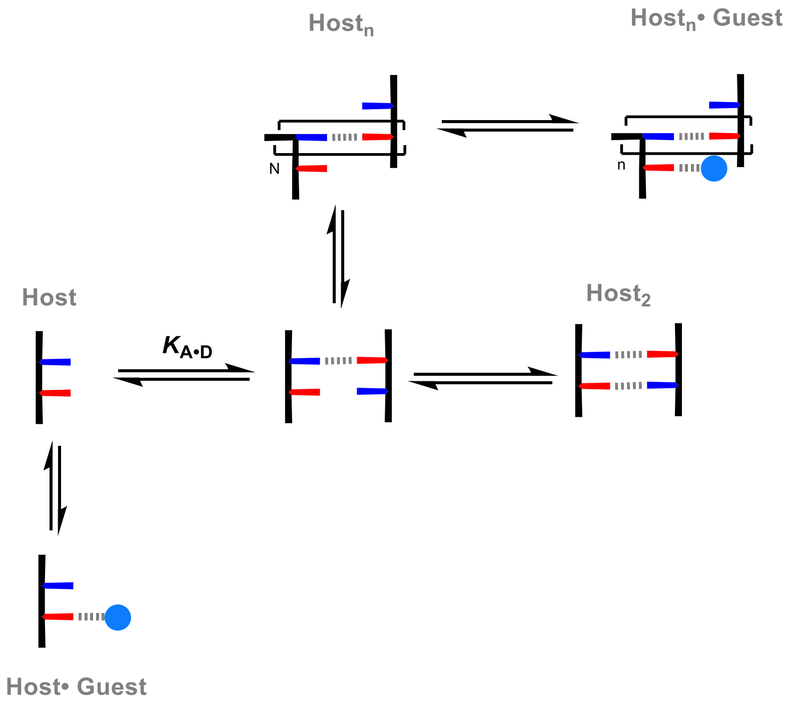


*Figure S 104 H-bonding equilibria between **AD** and PFTB (blue circle) and naming system used in the Musketeer fitting.*

*Table S 2 Summary of equations used in the Musketeer fit of PFTB titration into **AD**.*

| Species formed | Global K | Extinction coefficient as a function of extinction coefficients of free 4-nitrophenol ($\varepsilon_{free}$) and bound 4-nitrophenol ($\varepsilon_{bound}$) |
|---|---|---|
| Host | - | 1 $\varepsilon_{free}$ + 0 $\varepsilon_{bound}$ |
| Host•Guest | $K_d$ | 1 $\varepsilon_{free}$ + 0 $\varepsilon_{bound}$ |
| $Host_2$ | $0.5\ K_{A•D}\ EM_d$ | 0 $\varepsilon_{free}$ + 2 $\varepsilon_{bound}$ |
| $Host_n$ | $K_{A•D}{}^N$ | 1 $\varepsilon_{free}$ + N $\varepsilon_{bound}$ |
| $Host_n$•Guest | $K_{A•D}{}^{N+1}\ K_d$ | 1 $\varepsilon_{free}$ + N $\varepsilon_{bound}$ |

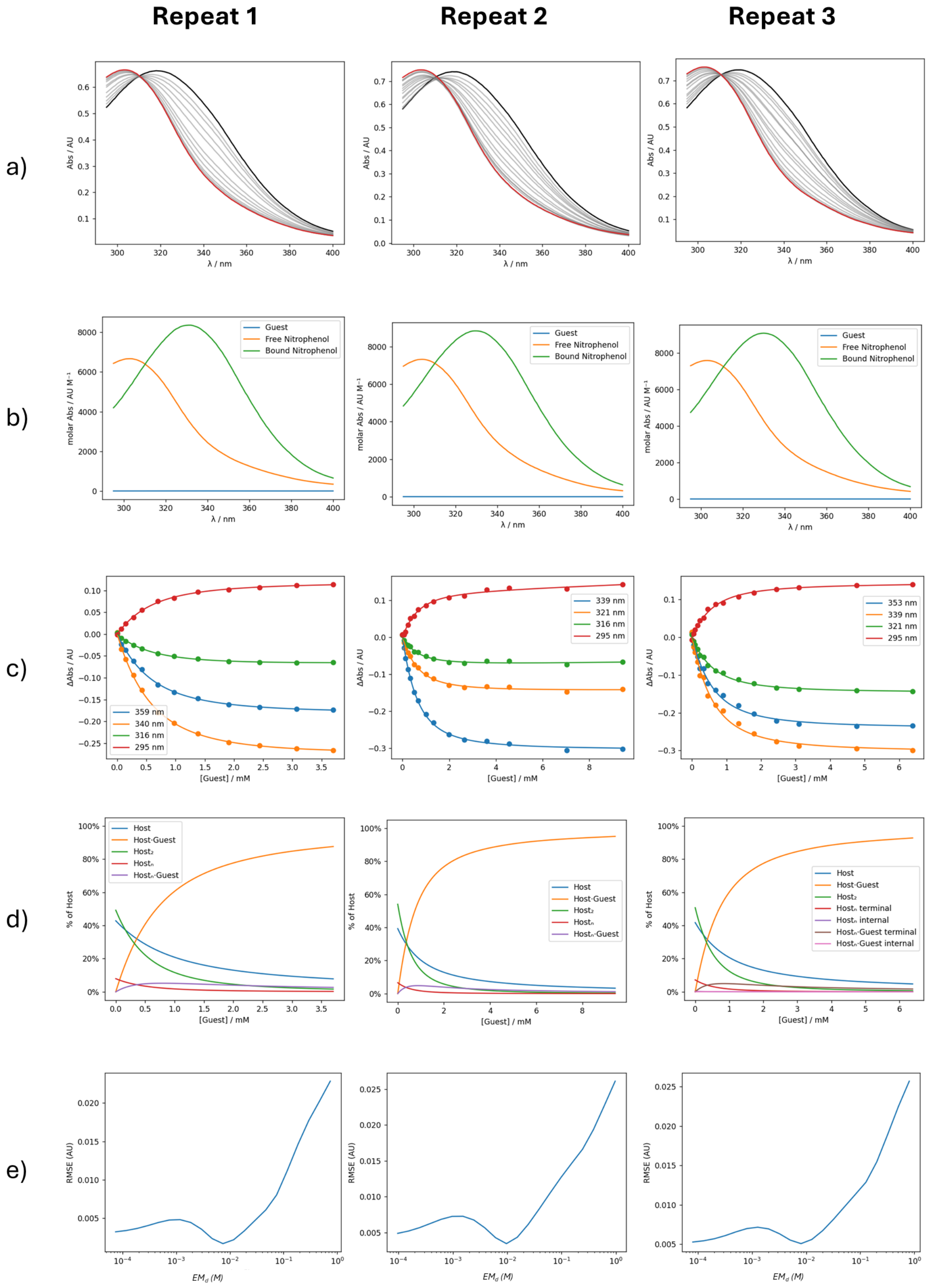


*Figure S 105 UV-Vis absorption denaturation of* ***AD*** *(100 µM) with PFTB (guest) in dichloromethane at 298 K. a) UV-Vis absorption spectra showing the starting spectrum in black and the final spectrum in red. b) Fitted spectra of free 4-nitrohenol, bound 4-nitrophenol and PFTB c) Best fit of the change in UV-Vis absorbance at selected wavelengths to an 5-species isotherm as described in the model in Figure S 104 and Table S 2 allowing for guest absorption. d) Calculated populations of different species containing* ***AD*** *and guest e) Relationship between the RMSE between the experimental data and calculated spectra plotted as a function of the value* $\mathbf{EM_d}$.

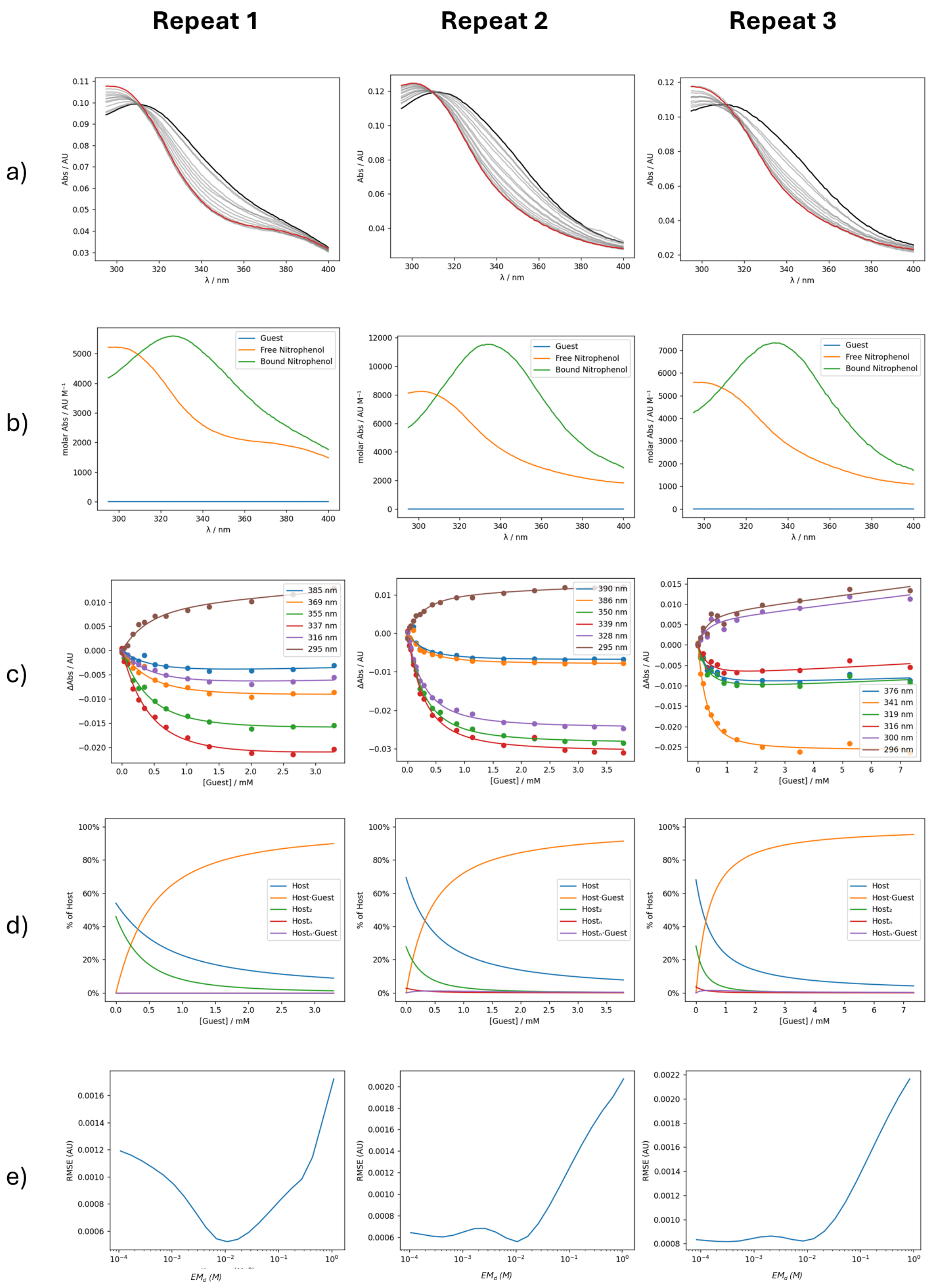


*Figure S 106 UV-Vis absorption denaturation of **AD** (10 µM) with PFTB (guest) in dichloromethane at 298 K. a) UV-Vis absorption spectra showing the starting spectrum in black and the final spectrum in red. b) Fitted spectra of free 4-nitrohenol, bound 4-nitrophenol and PFTB c) Best fit of the change in UV-Vis absorbance at selected wavelengths to an 5-species isotherm as described in the model in Figure S 104 and Table S 2 allowing for guest absorption. d) Calculated populations of different species containing **AD** and guest e) Relationship between the RMSE between the experimental data and calculated spectra plotted as a function of the value* $\boldsymbol{EM_d}$.

# **AOD**•PFTB fitted to duplex+folding model

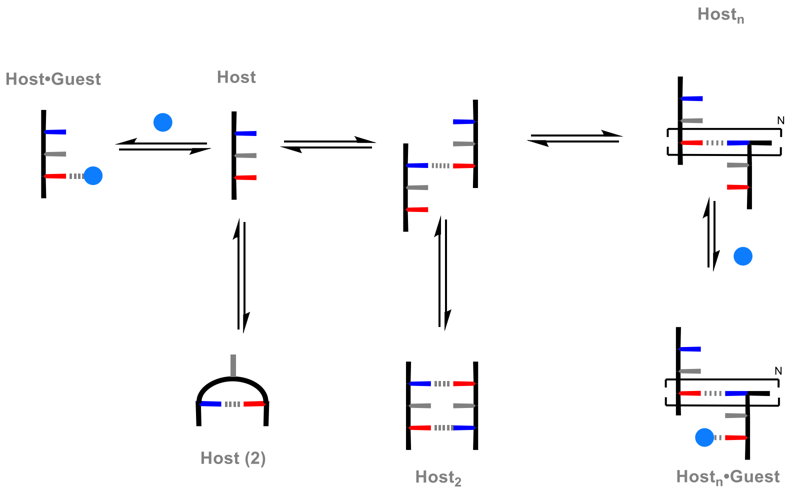


*Figure S 107 H-bonding equilibria between **AOD** and PFTB (blue circle) and naming system used in the Musketeer fitting.*

*Table S 3 Summary of equations used in the Musketeer fit of PFTB titration into **AOD.***

| Species formed | Global K | Extinction coefficient as a function of extinction coefficients of free 4-nitrophenol ($\varepsilon_{free}$) and bound 4-nitrophenol ($\varepsilon_{bound}$) |
|---|---|---|
| Host | - | 1 $\varepsilon_{free}$ + 0 $\varepsilon_{bound}$ |
| Host•Guest | $\boldsymbol{K_d}$ | 1 $\varepsilon_{free}$ + 0 $\varepsilon_{bound}$ |
| $Host_2$ | $\boldsymbol{0.5\ K_{A\bullet D}\ EM_d}$ | 0 $\varepsilon_{free}$ + 2 $\varepsilon_{bound}$ |
| Host (2) | $\boldsymbol{K_{A\bullet D}\ EM_f}$ | 0 $\varepsilon_{free}$ + 1 $\varepsilon_{bound}$ |
| $Host_n$ | $\boldsymbol{K_{A\bullet D}^{N}}$ | 1 $\varepsilon_{free}$ + N $\varepsilon_{bound}$ |
| $Host_n$•Guest | $\boldsymbol{K_{A\bullet D}^{N}\ K_d}$ | 1 $\varepsilon_{free}$ + N $\varepsilon_{bound}$ |

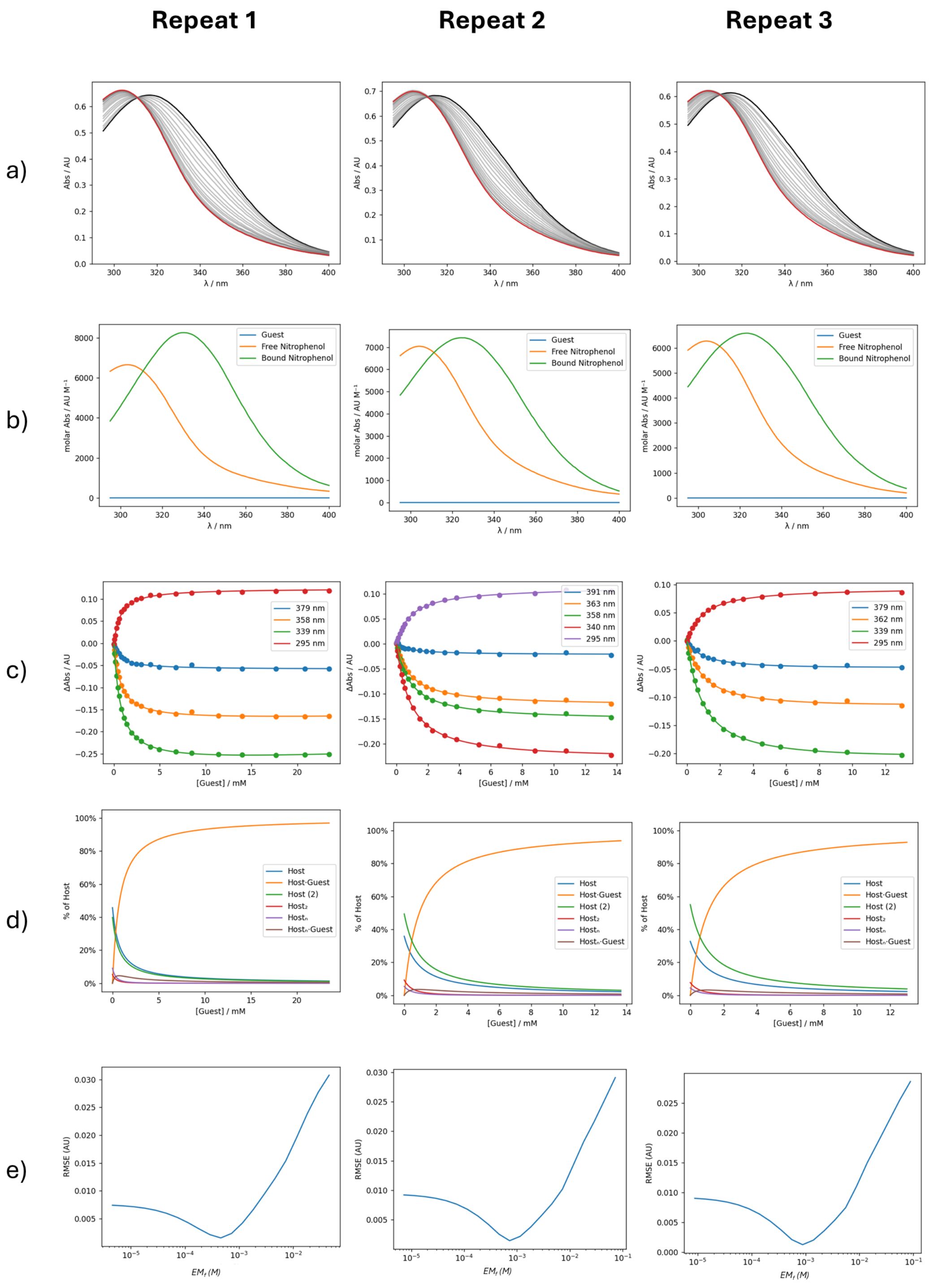


*Figure S 108 UV-Vis absorption denaturation of **AOD** (100 µM) with PFTB (guest) in dichloromethane at 298 K. a) UV-Vis absorption spectra showing the starting spectrum in black and the final spectrum in red. b) Fitted spectra of free 4-nitrohenol, bound 4-nitrophenol and PFTB  c) Best fit of the change in UV-Vis absorbance at selected wavelengths to an 6-species isotherm as described in the model in Figure S 107 and Table S 3 allowing for guest absorption. d) Calculated populations of different species containing **AOD** and guest e)  Relationship between the RMSE between the experimental data and calculated spectra plotted as a function of the value* $\boldsymbol{EM_f}$.

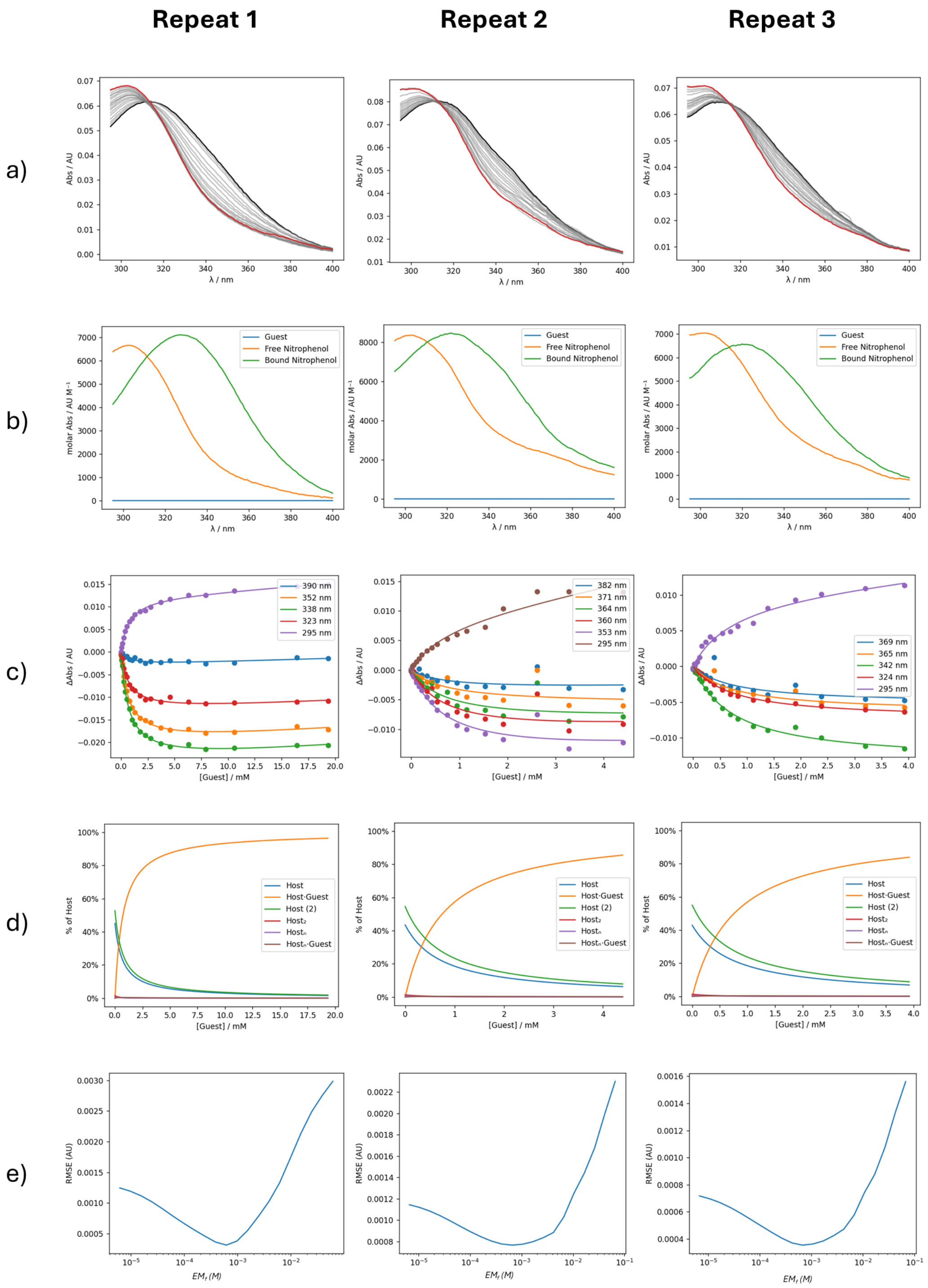


*Figure S 109 UV-Vis absorption denaturation of **AOD** (10 μM) with PFTB (guest) in dichloromethane at 298 K. a) UV-Vis absorption spectra showing the starting spectrum in black and the final spectrum in red. b) Fitted spectra of free 4-nitrohenol, bound 4-nitrophenol and PFTB c) Best fit of the change in UV-Vis absorbance at selected wavelengths to an 6-species isotherm as described in the model in Figure S 107 and Table S 3 allowing for guest absorption. d) Calculated populations of different species containing **AOD** and guest e) Relationship between the RMSE between the experimental data and calculated spectra plotted as a function of the value **EM**$_f$.*

# **AOOD**•PFTB fitted to folding model

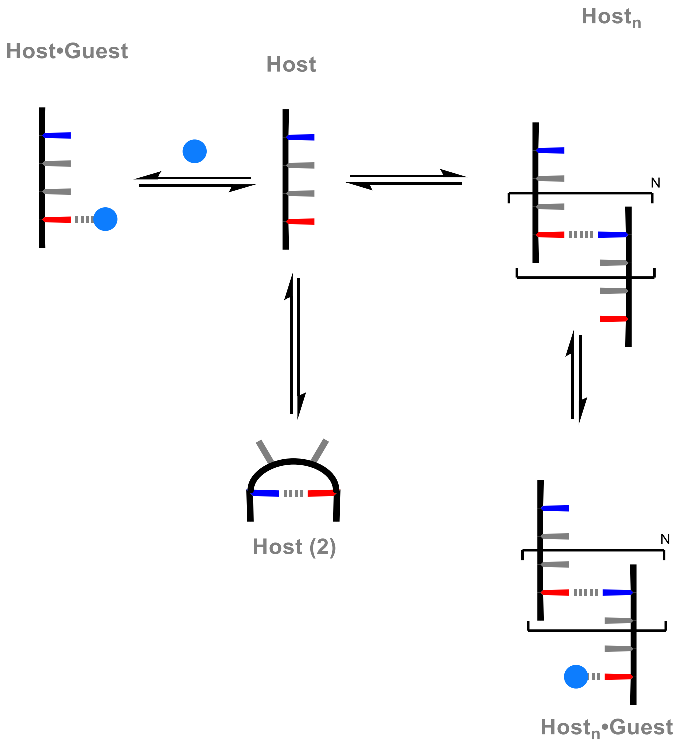


*Figure S 110 H-bonding equilibria between **AO$_n$D** (n = 2 to 8) and PFTB (blue circle) and naming system used in the Musketeer fitting, **AOOD** shown as example.*

*Table S 4 Summary of equations used in the Musketeer fit of PFTB titration into **AO$_n$D** (n = 2 to 8).*

| Species formed | Global K | Extinction coefficient as a function of extinction coefficients of free 4-nitrophenol ($\varepsilon_{free}$) and bound 4-nitrophenol ($\varepsilon_{bound}$) |
|---|---|---|
| Host | - | 1 $\varepsilon_{free}$ + 0 $\varepsilon_{bound}$ |
| Host•Guest | $K_d$ | 1 $\varepsilon_{free}$ + 0 $\varepsilon_{bound}$ |
| Host (2) | $K_{A\bullet D}\, EM_f$ | 0 $\varepsilon_{free}$ + 1 $\varepsilon_{bound}$ |
| Host$_n$ | $K_{A\bullet D}{}^N$ | 1 $\varepsilon_{free}$ + N $\varepsilon_{bound}$ |
| Host$_n$•Guest | $K_{A\bullet D}{}^N\, K_d$ | 1 $\varepsilon_{free}$ + N $\varepsilon_{bound}$ |

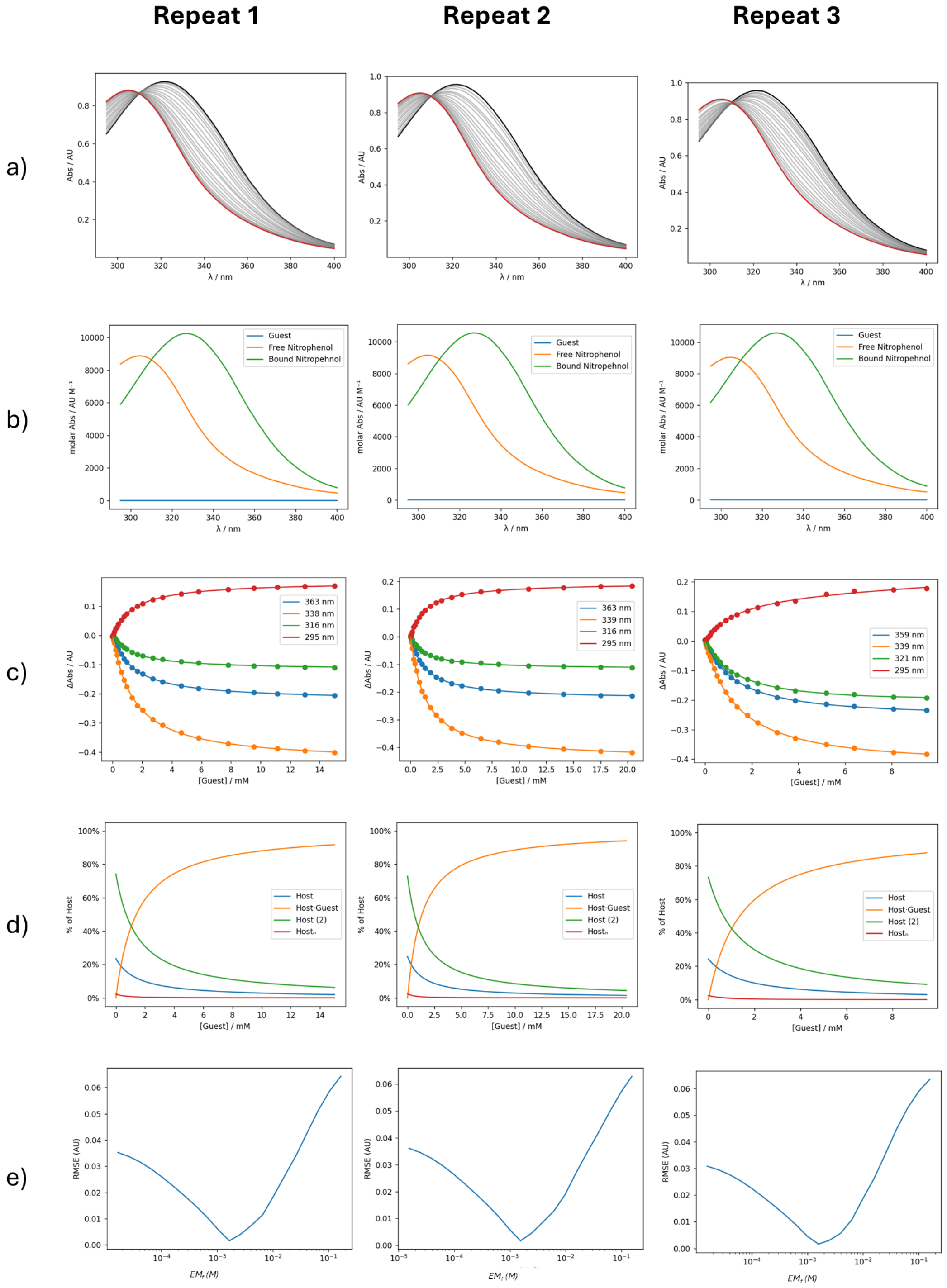


*Figure S 111 UV-Vis absorption denaturation of **AOOD** (100 µM) with PFTB (guest) in dichloromethane at 298 K. a) UV-Vis absorption spectra showing the starting spectrum in black and the final spectrum in red. b) Fitted spectra of free 4-nitrohenol, bound 4-nitrophenol and PFTB c) Best fit of the change in UV-Vis absorbance at selected wavelengths to an 5-species isotherm as described in the model in Figure S 110 and Table S 4 allowing for guest absorption. d) Calculated populations of different species containing **AOOD** and guest e) Relationship between the RMSE between the experimental data and calculated spectra plotted as a function of the value **EM$_f$**.*

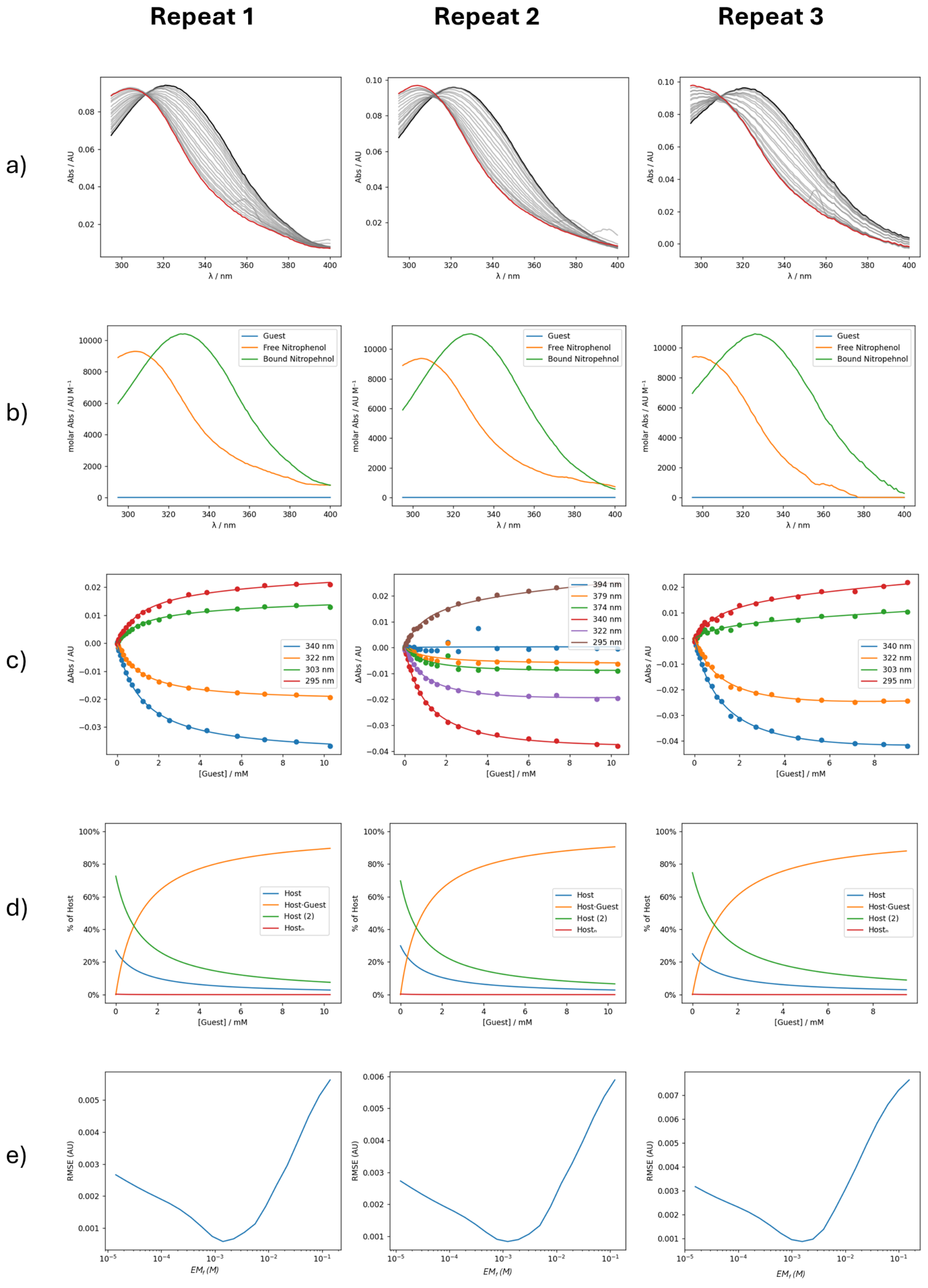


*Figure S 112 UV-Vis absorption denaturation of **AOOD** (10 µM) with PFTB (guest) in dichloromethane at 298 K. a) UV-Vis absorption spectra showing the starting spectrum in black and the final spectrum in red. b) Fitted spectra of free 4-nitrohenol, bound 4-nitrophenol and PFTB c) Best fit of the change in UV-Vis absorbance at selected wavelengths to an 5-species isotherm as described in the model in Figure S 110 and Table S 4 allowing for guest absorption. d) Calculated populations of different species containing **AOOD** and guest e) Relationship between the RMSE between the experimental data and calculated spectra plotted as a function of the value* $\boldsymbol{EM_f}$.

# **AOOOD**•PFTB fitted to folding model

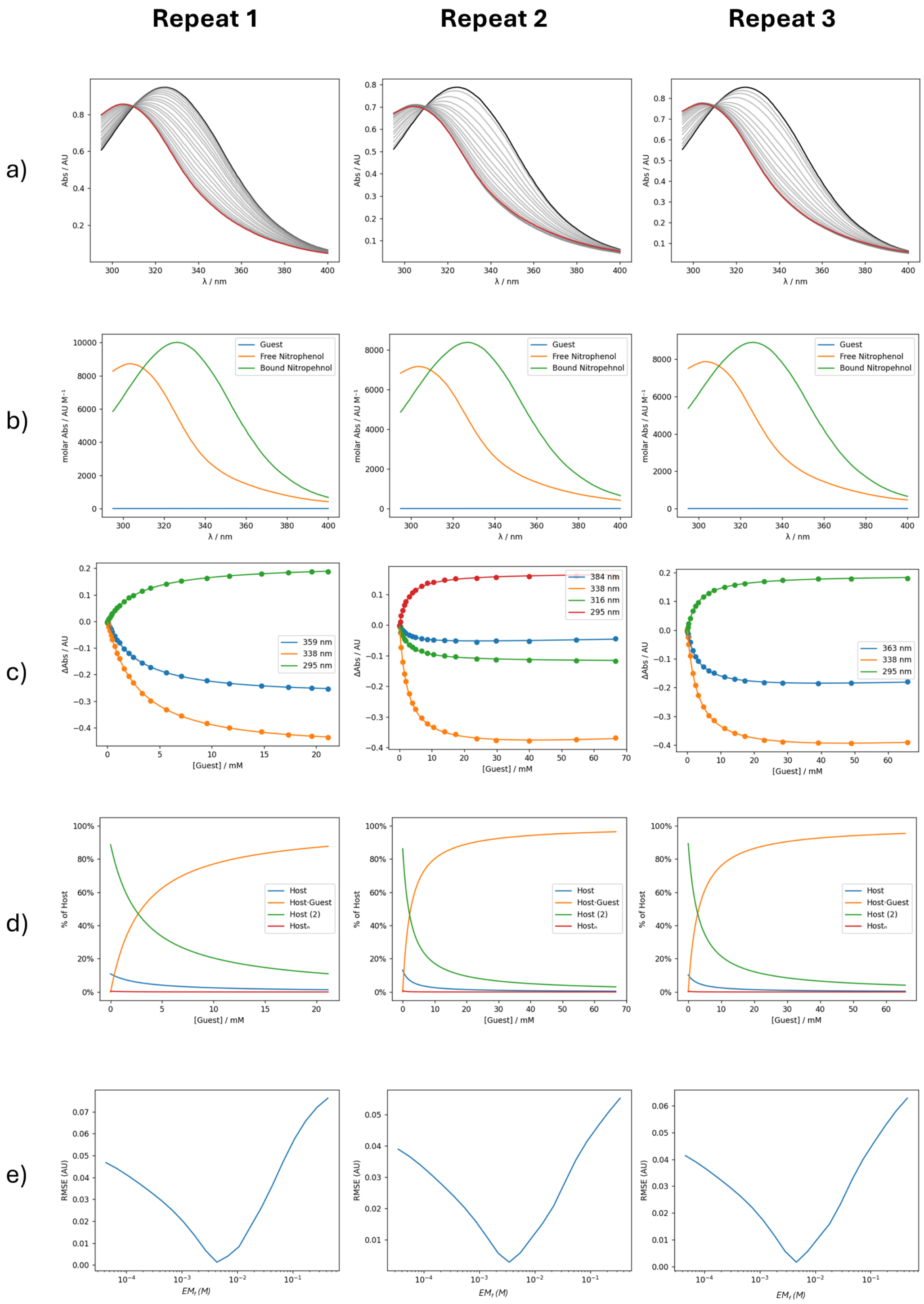


*Figure S 113 UV-Vis absorption denaturation of **AOOOD** (100 µM) with PFTB (guest) in dichloromethane at 298 K. a) UV-Vis absorption spectra showing the starting spectrum in black and the final spectrum in red. b) Fitted spectra of free 4-nitrohenol, bound 4-nitrophenol and PFTB c) Best fit of the change in UV-Vis absorbance at selected wavelengths to an 5-species isotherm as described in the model in Figure S 110 and Table S 4 allowing for guest absorption. d) Calculated populations of different species containing **AOOOD** and guest e) Relationship between the RMSE between the experimental data and calculated spectra plotted as a function of the value* $\boldsymbol{EM_f}$.

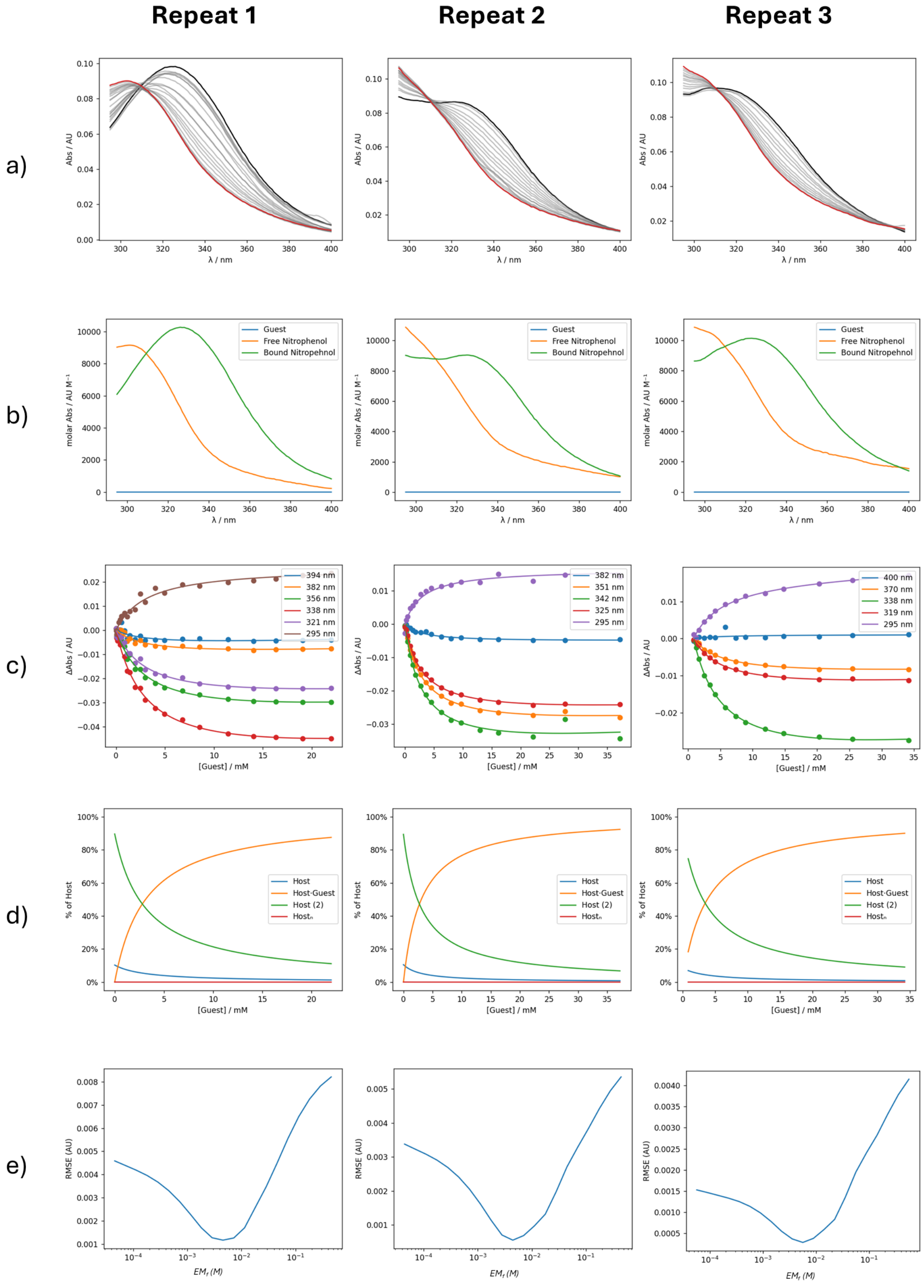


*Figure S 114 UV-Vis absorption denaturation of **AOOOD** (10 µM) with PFTB (guest) in dichloromethane at 298 K. a) UV-Vis absorption spectra showing the starting spectrum in black and the final spectrum in red. b) Fitted spectra of free 4-nitrohenol, bound 4-nitrophenol and PFTB c) Best fit of the change in UV-Vis absorbance at selected wavelengths to an 5-species isotherm as described in the model in Figure S 110 and Table S 4 allowing for guest absorption. d) Calculated populations of different species containing **AOOOD** and guest e) Relationship between the RMSE between the experimental data and calculated spectra plotted as a function of the value $\boldsymbol{EM_f}$.*

# **AOOOOD**•PFTB fitted to folding model

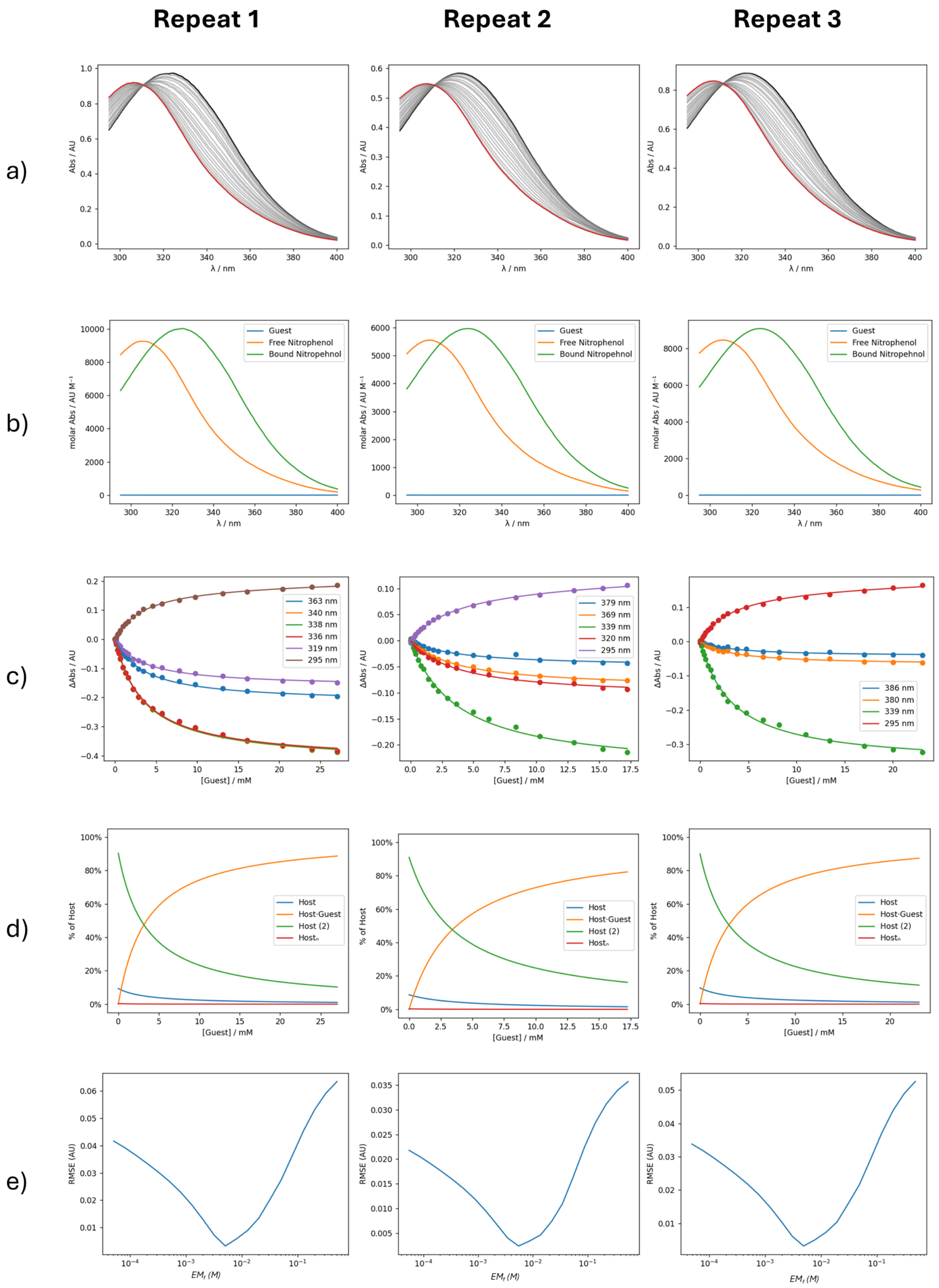


*Figure S 115 UV-Vis absorption denaturation of **AOOOOD** (100 µM) with PFTB (guest) in dichloromethane at 298 K. a) UV-Vis absorption spectra showing the starting spectrum in black and the final spectrum in red. b) Fitted spectra of free 4-nitrohenol, bound 4-nitrophenol and PFTB c) Best fit of the change in UV-Vis absorbance at selected wavelengths to an 5-species isotherm as described in the model in Figure S 110 and Table S 4 allowing for guest absorption. d) Calculated populations of different species containing **AOOOOD** and guest e) Relationship between the RMSE between the experimental data and calculated spectra plotted as a function of the value **EM**$_f$.*

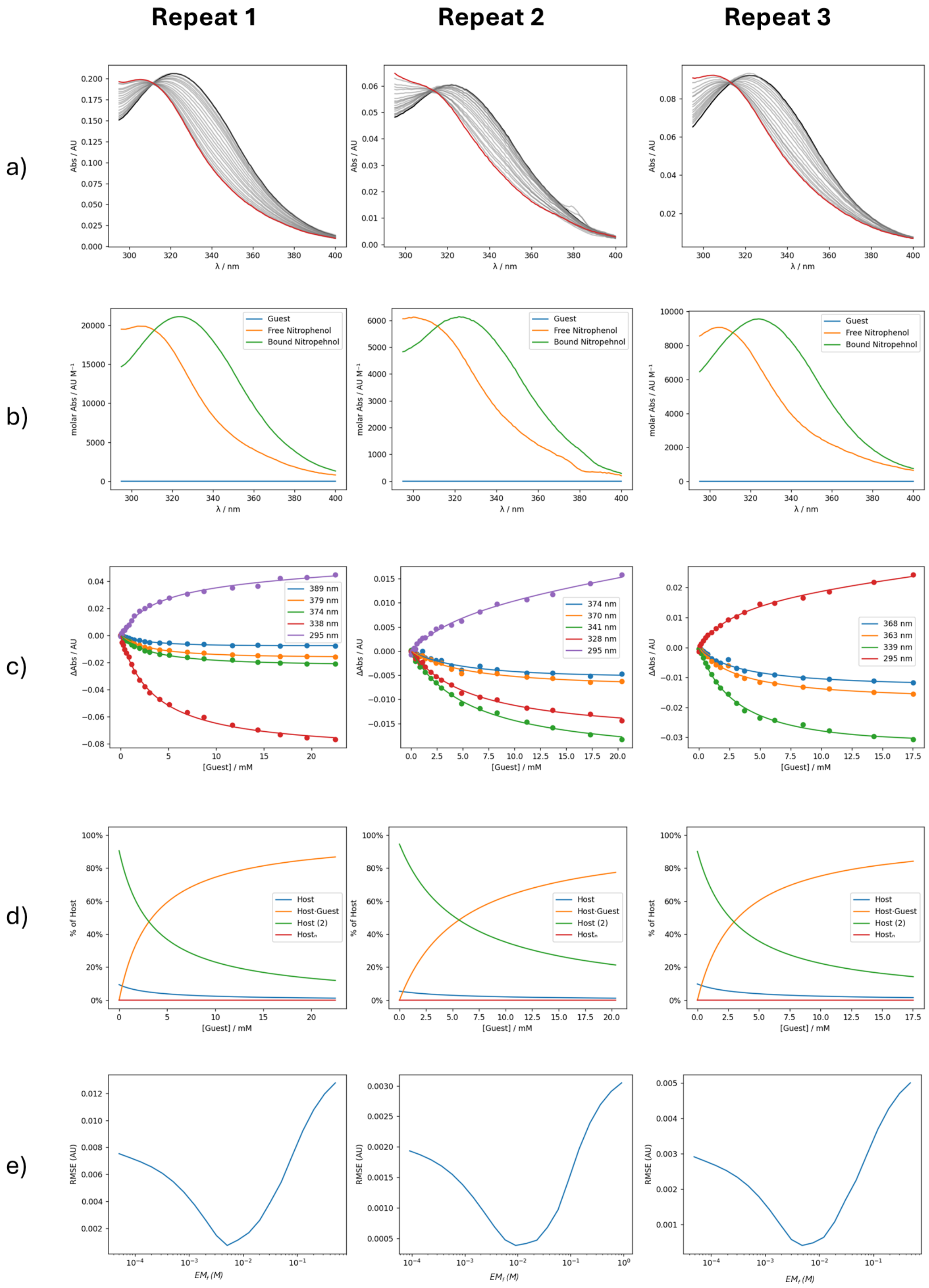


*Figure S 116 UV-Vis absorption denaturation of **AOOOOD** (10 µM) with PFTB (guest) in dichloromethane at 298 K. a) UV-Vis absorption spectra showing the starting spectrum in black and the final spectrum in red. b) Fitted spectra of free 4-nitrohenol, bound 4-nitrophenol and PFTB  c) Best fit of the change in UV-Vis absorbance at selected wavelengths to an 5-species isotherm as described in the model in Figure S 110 and Table S 4 allowing for guest absorption. d) Calculated populations of different species containing **AOOOOD** and guest e)  Relationship between the RMSE between the experimental data and calculated spectra plotted as a function of the value $\mathbf{EM_f}$.*

# **AO$_5$D**•PFTB fitted to folding model

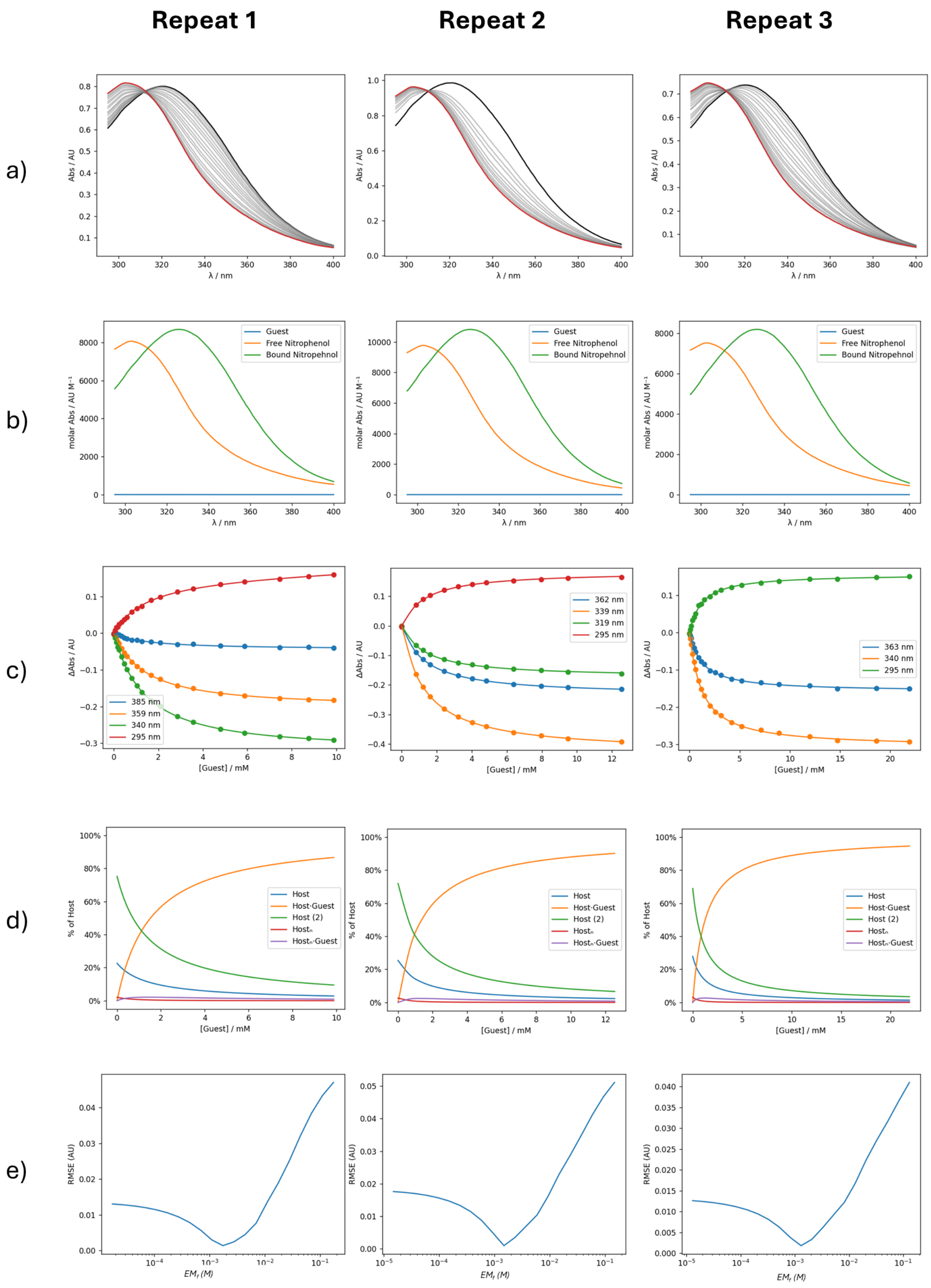


*Figure S 117 UV-Vis absorption denaturation of **AO$_5$D** (100 µM) with PFTB (guest) in dichloromethane at 298 K. a) UV-Vis absorption spectra showing the starting spectrum in black and the final spectrum in red. b) Fitted spectra of free 4-nitrohenol, bound 4-nitrophenol and PFTB c) Best fit of the change in UV-Vis absorbance at selected wavelengths to an 5-species isotherm as described in the model in Figure S 110 and Table S 4 allowing for guest absorption. d) Calculated populations of different species containing **AO$_5$D** and guest e) Relationship between the RMSE between the experimental data and calculated spectra plotted as a function of the value **EM$_f$**.*

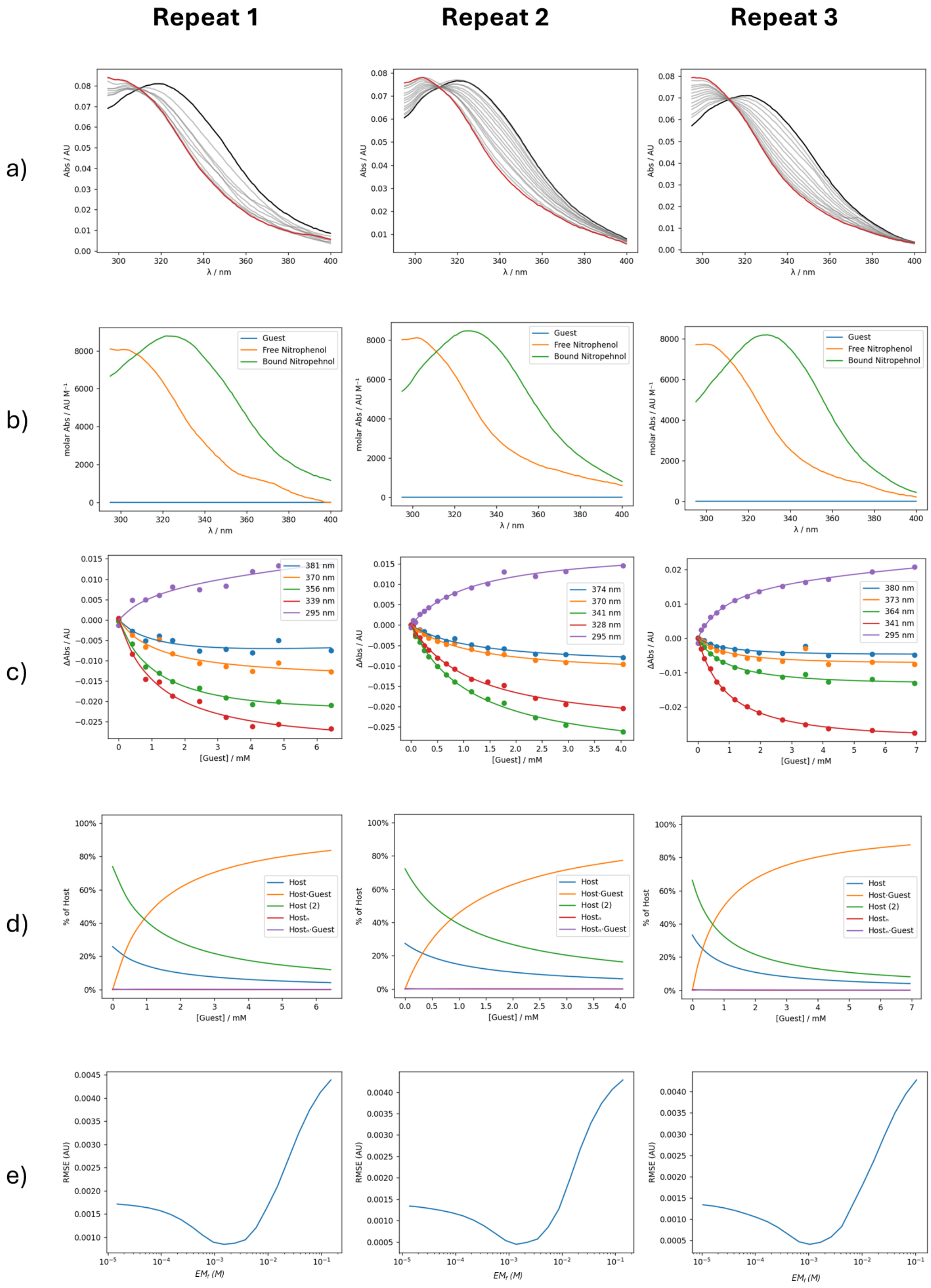


*Figure S 118 UV-Vis absorption denaturation of* ***AO$_5$D*** *(10 µM) with PFTB (guest) in dichloromethane at 298 K. a) UV-Vis absorption spectra showing the starting spectrum in black and the final spectrum in red. b) Fitted spectra of free 4-nitrohenol, bound 4-nitrophenol and PFTB c) Best fit of the change in UV-Vis absorbance at selected wavelengths to an 5-species isotherm as described in the model in Figure S 110 and Table S 4 allowing for guest absorption. d) Calculated populations of different species containing* ***AO$_5$D*** *and guest e) Relationship between the RMSE between the experimental data and calculated spectra plotted as a function of the value* ***EM$_f$****.*

# AO$_6$D•PFTB fitted to folding model

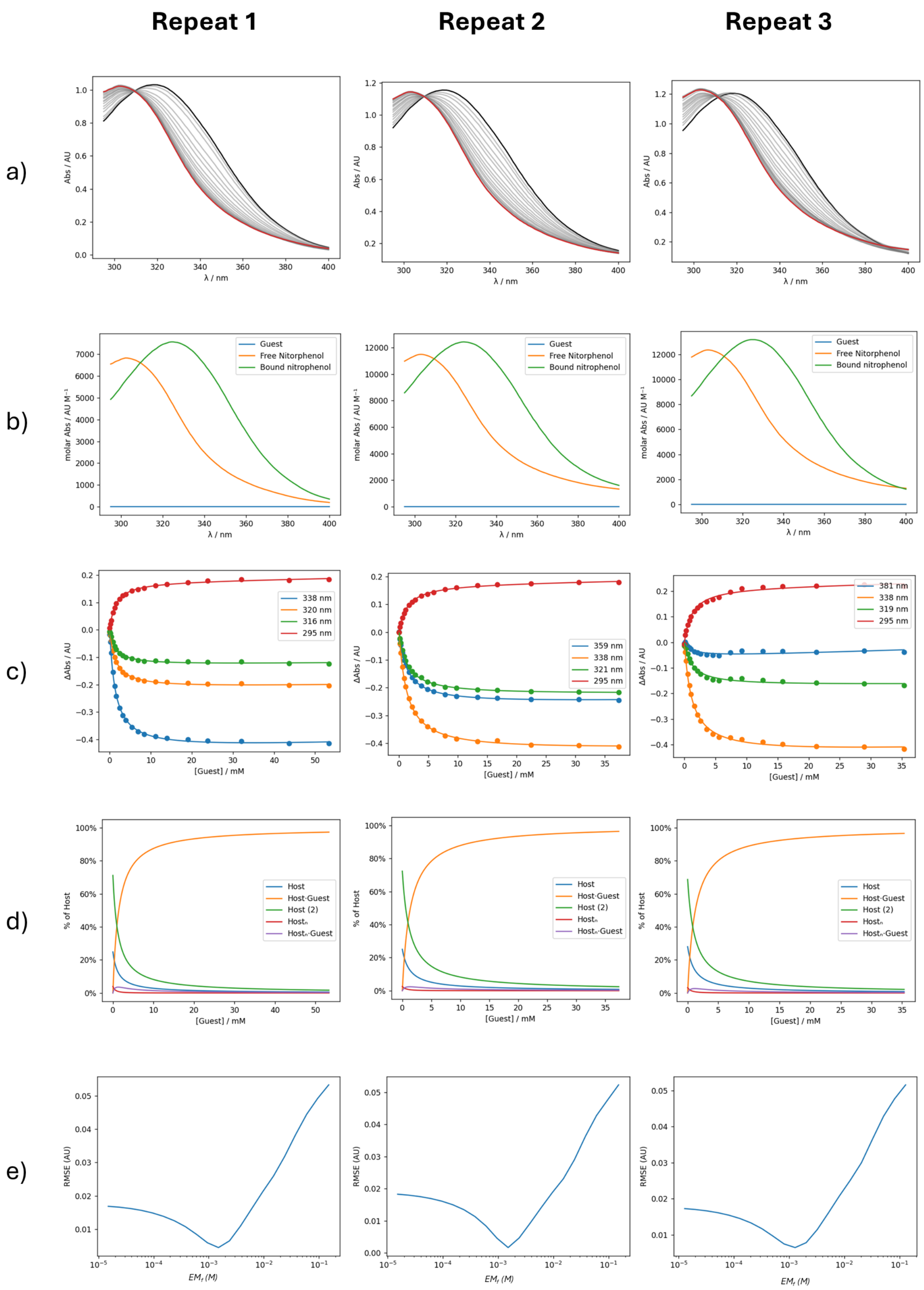


*Figure S 119 UV-Vis absorption denaturation of **AO$_6$D** (100 µM) with PFTB (guest) in dichloromethane at 298 K. a) UV-Vis absorption spectra showing the starting spectrum in black and the final spectrum in red. b) Fitted spectra of free 4-nitrohenol, bound 4-nitrophenol and PFTB c) Best fit of the change in UV-Vis absorbance at selected wavelengths to an 5-species isotherm as described in the model in Figure S 110 and Table S 4 allowing for guest absorption. d) Calculated populations of different species containing **AO$_6$D** and guest e) Relationship between the RMSE between the experimental data and calculated spectra plotted as a function of the value **EM$_f$**.*

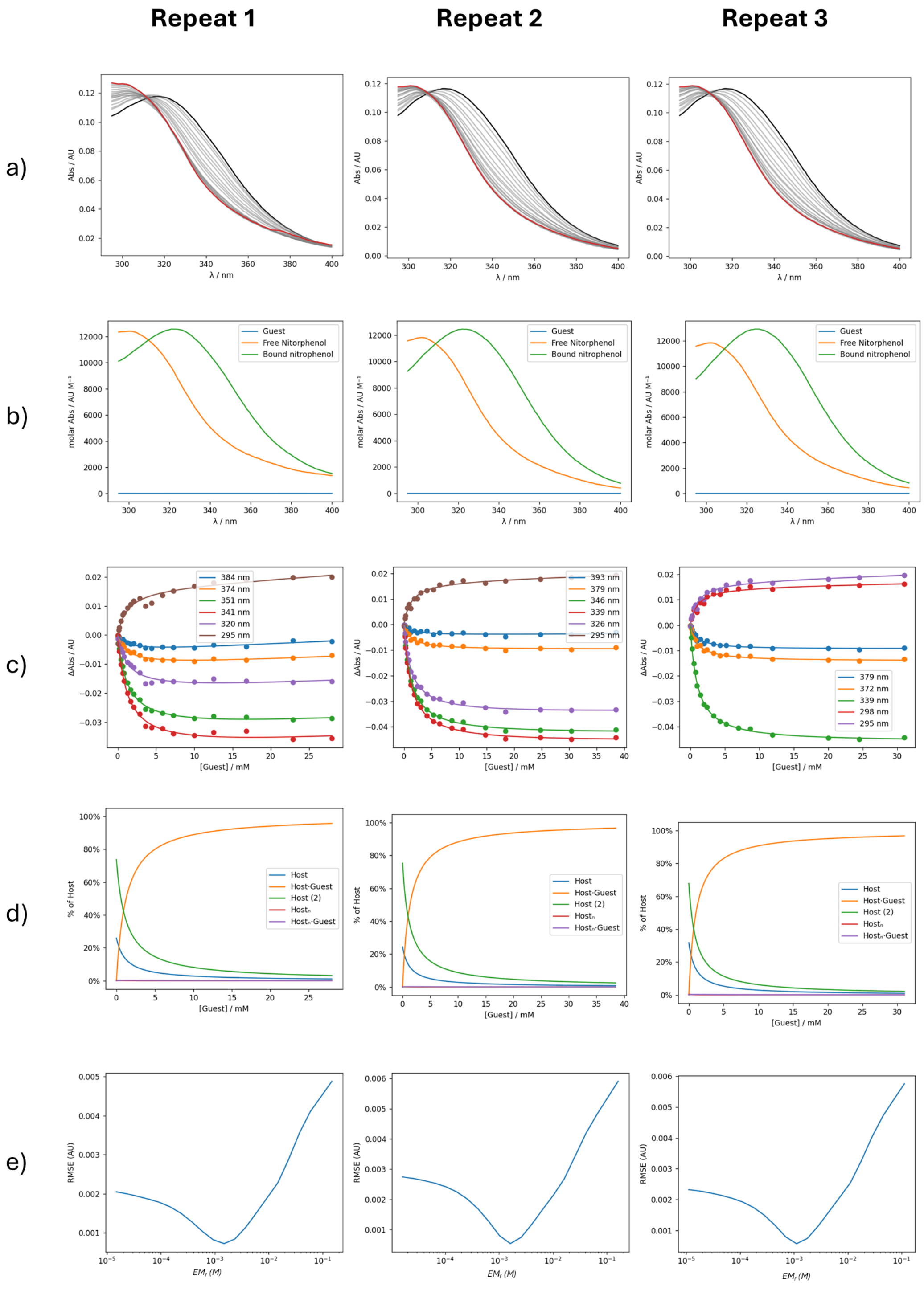


*Figure S 120 UV-Vis absorption denaturation of* ***AO₆D*** *(10 µM) with PFTB (guest) in dichloromethane at 298 K. a) UV-Vis absorption spectra showing the starting spectrum in black and the final spectrum in red. b) Fitted spectra of free 4-nitrohenol, bound 4-nitrophenol and PFTB c) Best fit of the change in UV-Vis absorbance at selected wavelengths to an 5-species isotherm as described in the model in Figure S 110 and Table S 4 allowing for guest absorption. d) Calculated populations of different species containing* ***AO₆D*** *and guest e) Relationship between the RMSE between the experimental data and calculated spectra plotted as a function of the value* ***EM$_f$***.

# $\mathbf{AO_7D}$•PFTB fitted to folding model

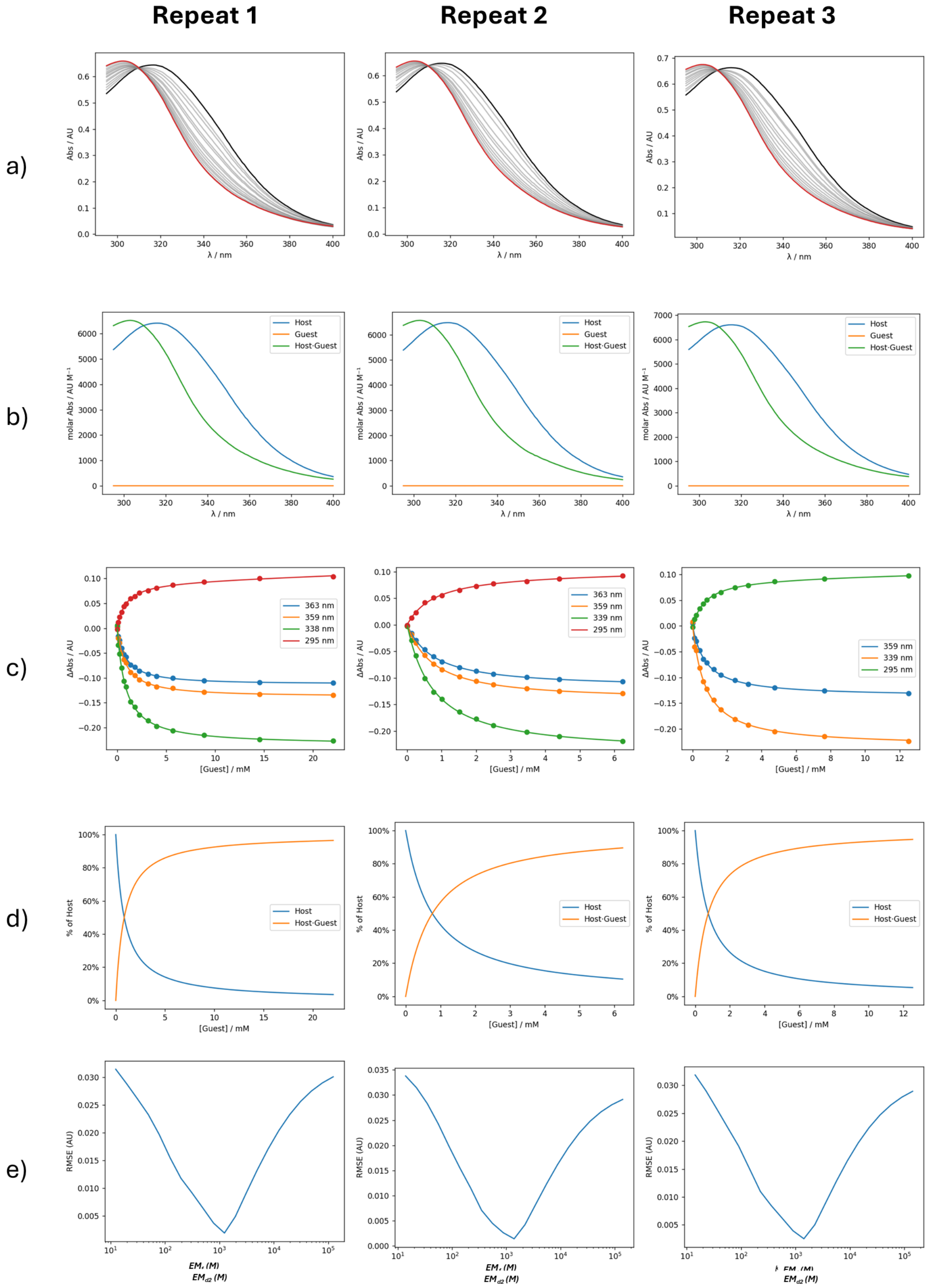


*Figure S 121 UV-Vis absorption denaturation of **$AO_7D$** (100 µM) with PFTB (guest) in dichloromethane at 298 K. a) UV-Vis absorption spectra showing the starting spectrum in black and the final spectrum in red. b) Fitted spectra of free 4-nitrohenol, bound 4-nitrophenol and PFTB c) Best fit of the change in UV-Vis absorbance at selected wavelengths to an 5-species isotherm as described in the model in Figure S 110 and Table S 4 allowing for guest absorption. d) Calculated populations of different species containing **$AO_6D$** and guest e) Relationship between the RMSE between the experimental data and calculated spectra plotted as a function of the value **$EM_f$**.*

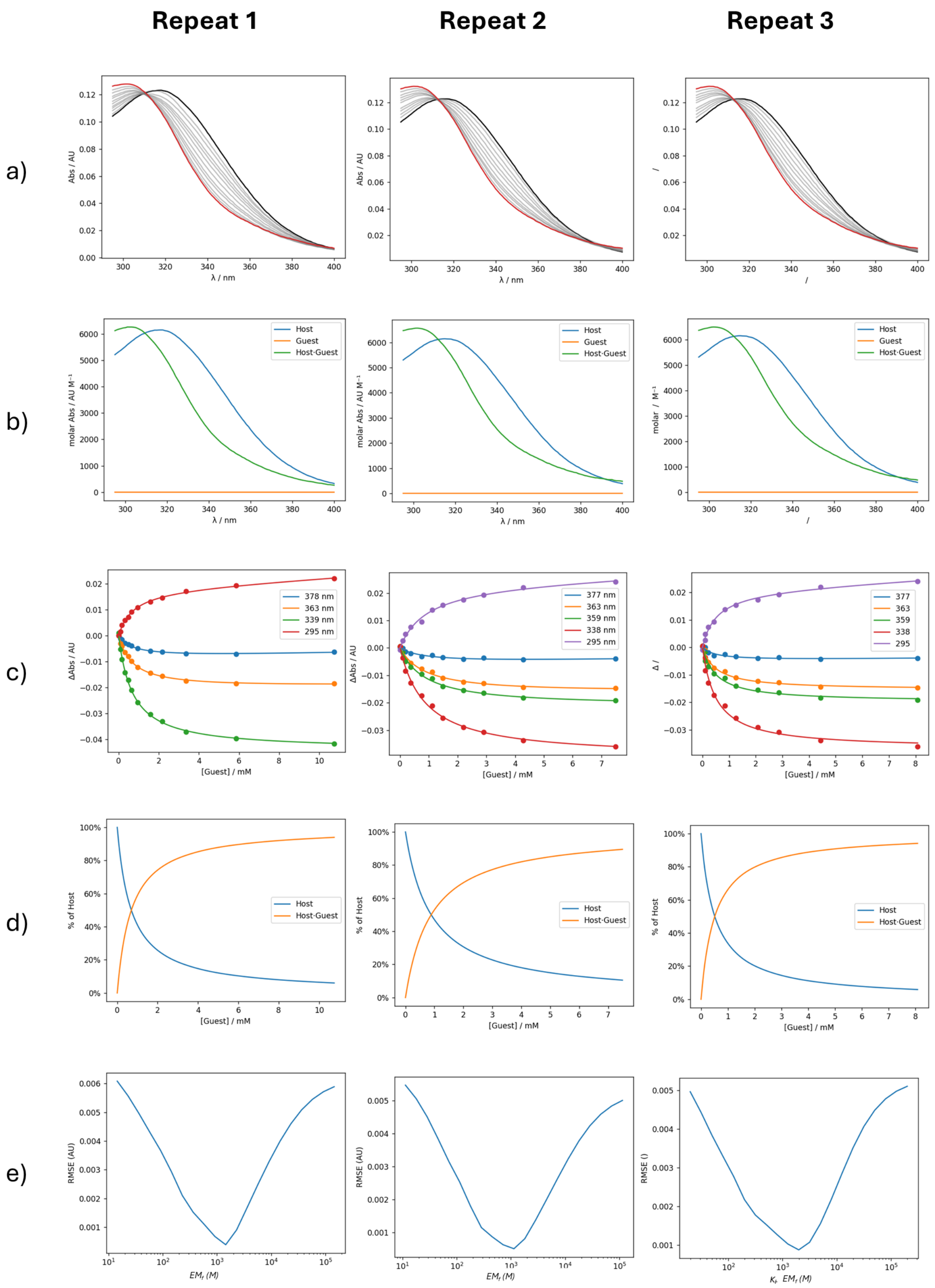


*Figure S 122 UV-Vis absorption denaturation of **$AO_7D$** (10 µM) with PFTB (guest) in dichloromethane at 298 K. a) UV-Vis absorption spectra showing the starting spectrum in black and the final spectrum in red. b) Fitted spectra of free 4-nitrohenol, bound 4-nitrophenol and PFTB c) Best fit of the change in UV-Vis absorbance at selected wavelengths to an 5-species isotherm as described in the model in Figure S 110 and Table S 4 allowing for guest absorption. d) Calculated populations of different species containing **$AO_7D$** and guest e) Relationship between the RMSE between the experimental data and calculated spectra plotted as a function of the value **$EM_f$**.*

# **AO$_8$D**•PFTB fitted to folding model

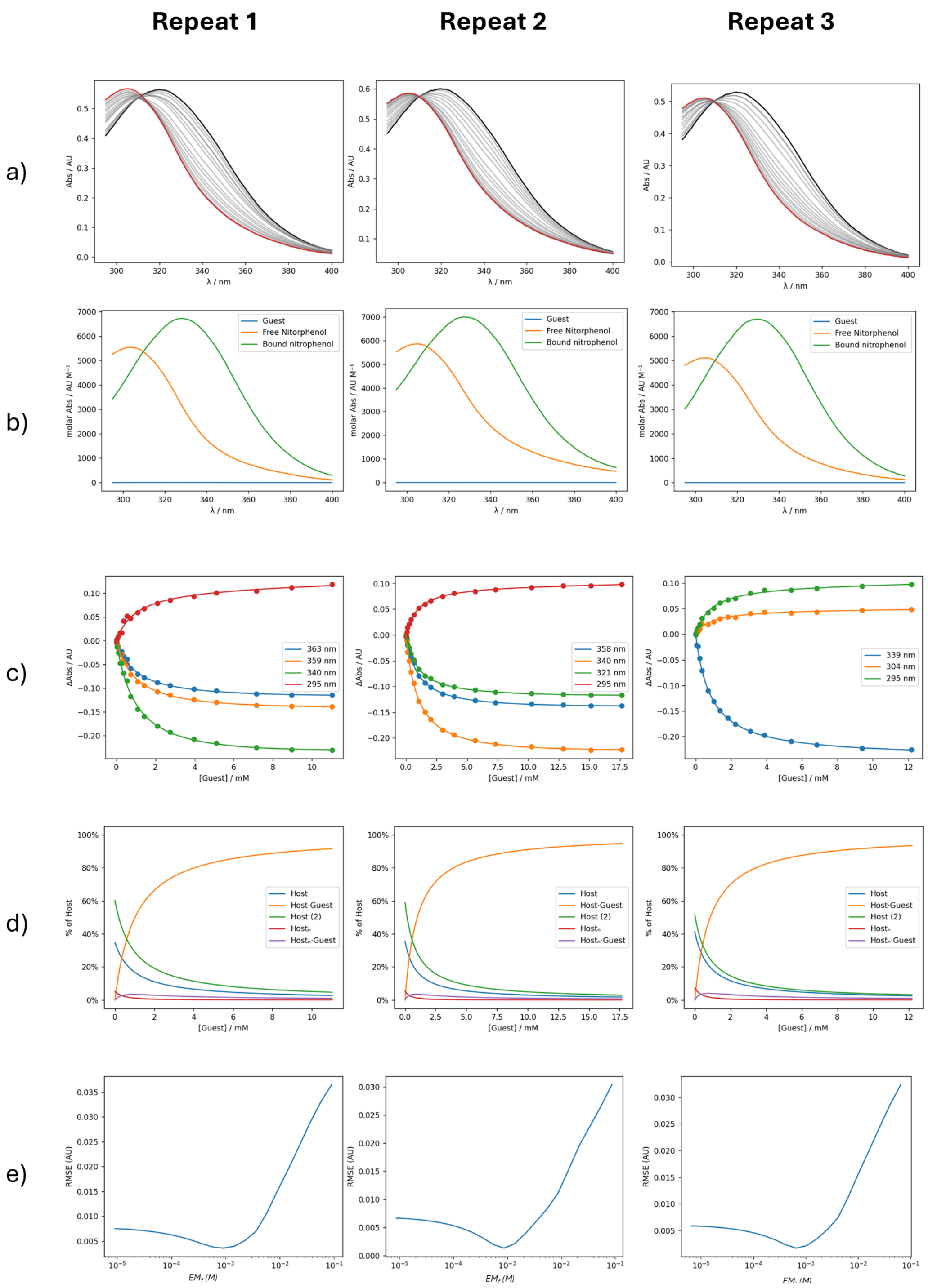


*Figure S 123 UV-Vis absorption denaturation of **AO$_8$D** (100 µM) with PFTB (guest) in dichloromethane at 298 K. a) UV-Vis absorption spectra showing the starting spectrum in black and the final spectrum in red. b) Fitted spectra of free 4-nitrohenol, bound 4-nitrophenol and PFTB c) Best fit of the change in UV-Vis absorbance at selected wavelengths to an 5-species isotherm as described in the model in Figure S 110 and Table S 4 allowing for guest absorption. d) Calculated populations of different species containing **AO$_8$D** and guest e) Relationship between the RMSE between the experimental data and calculated spectra plotted as a function of the value **EM$_f$**.*

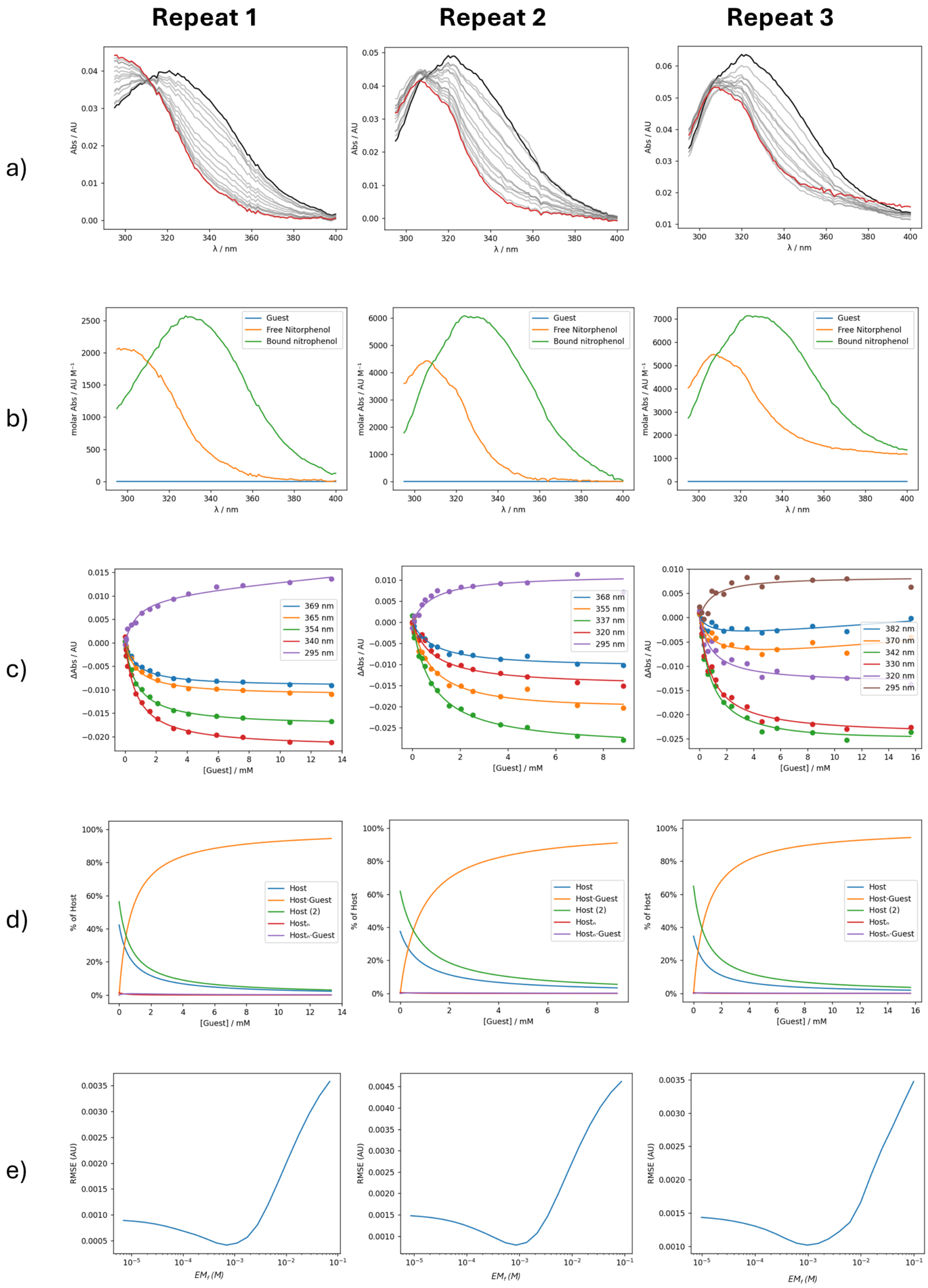


*Figure S 124 UV-Vis absorption denaturation of* ***AO$_8$D*** *(10 µM) with PFTB (guest) in dichloromethane at 298 K. a) UV-Vis absorption spectra showing the starting spectrum in black and the final spectrum in red. b) Fitted spectra of free 4-nitrohenol, bound 4-nitrophenol and PFTB c) Best fit of the change in UV-Vis absorbance at selected wavelengths to an 5-species isotherm as described in the model in Figure S 110 and Table S 4 allowing for guest absorption. d) Calculated populations of different species containing* ***AO$_8$D*** *and guest e) Relationship between the RMSE between the experimental data and calculated spectra plotted as a function of the value* ***EM$_f$****.*

# **DAO$_n$AD**•PFTB and **ADO$_n$AD**•PFTB fitted to 1:1 binding isotherm

## **DAO$_3$AD**•PFTB fitted to 1:1 binding isotherm

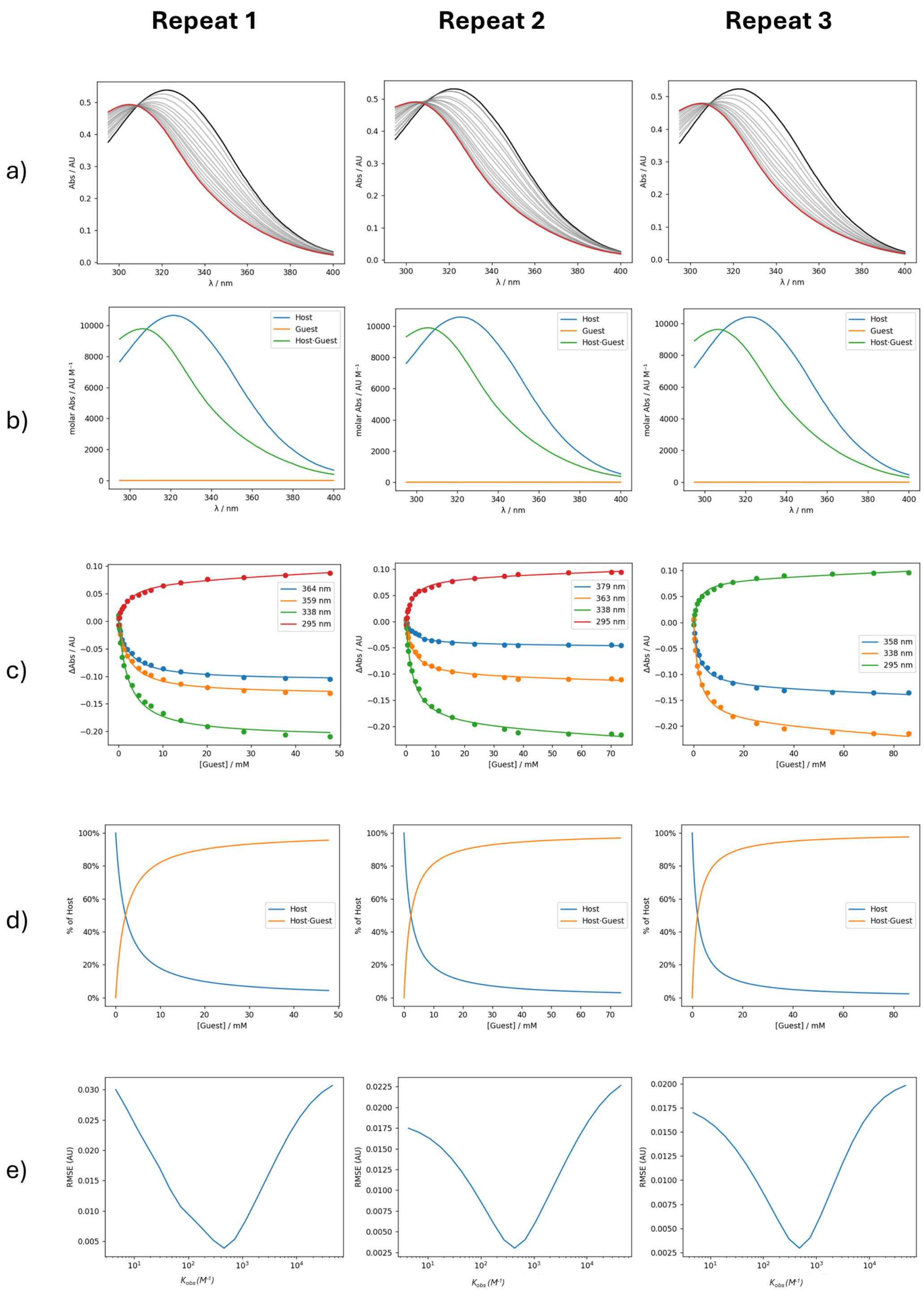


*Figure S 125 UV-Vis absorption titration of PFTB into **DAO$_3$AD** (50 µM) in dichloromethane at 298 K. a) UV-Vis absorption spectra showing free **DAO$_3$AD** in black and final spectrum in red. b) Fitted spectra for **DAO$_3$AD** (host), **DAO$_3$AD** •PFTB (host•guest) an PFTB (guest) c) Best fit of the change in UV-Vis absorbance at selected wavelengths to a 1:1 binding isotherm allowing for guest absorption. d) Calculated populations of different species containing **DAO$_3$AD** and guest. e) Relationship between the RMSE between the experimental data and calculated spectra plotted as a function of the value of $K_{obs}$.*

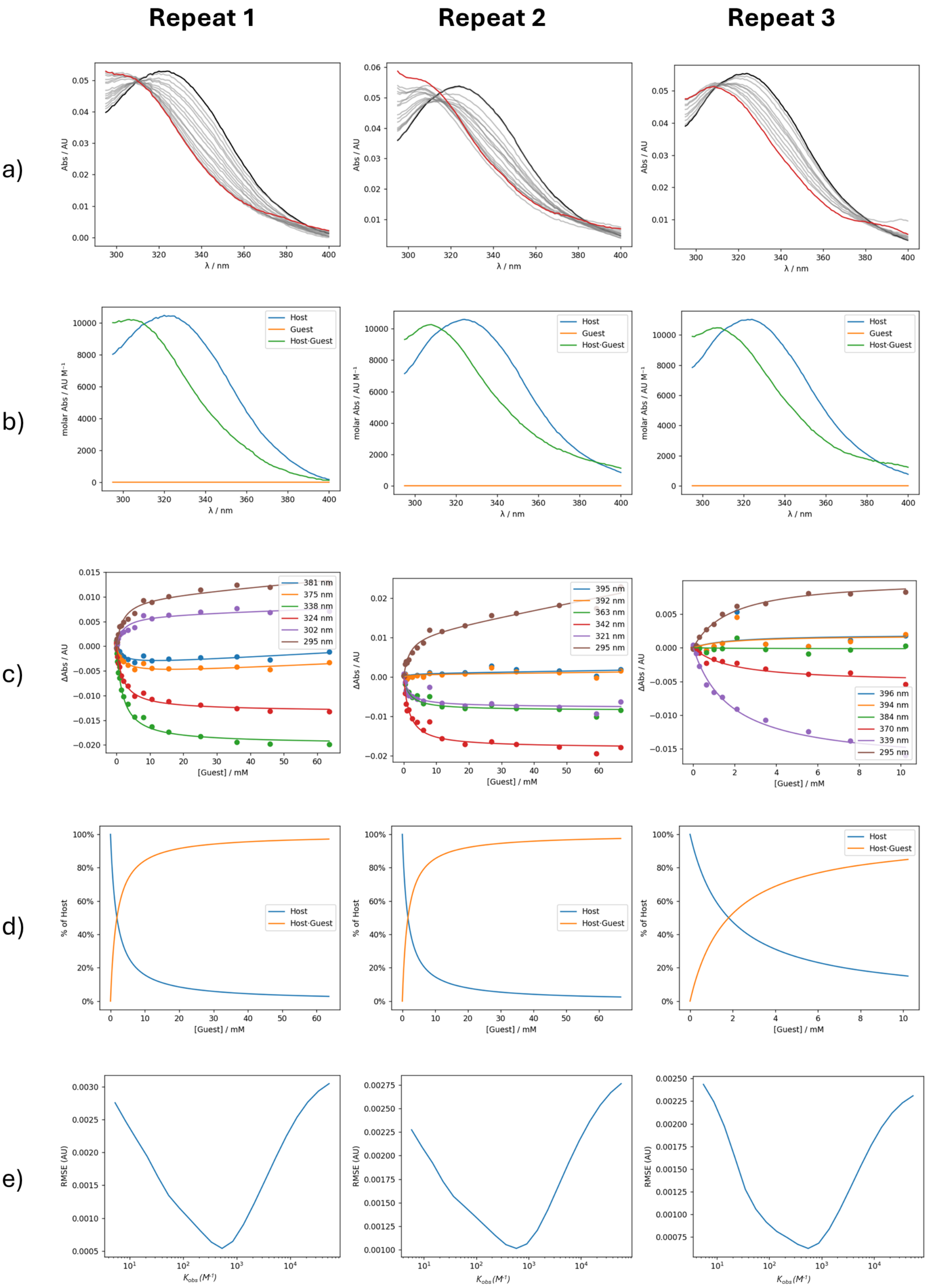


*Figure S 126 UV-Vis absorption titration of PFTB into* ***DAO$_3$AD*** *(5 μM) in dichloromethane at 298 K. a) UV-Vis absorption spectra showing free* ***DAO$_3$AD*** *in black and final spectrum in red. b) Fitted spectra for* ***DAO$_3$AD*** *(host),* ***DAO$_3$AD*** *•PFTB (host•guest) an PFTB (guest) c) Best fit of the change in UV-Vis absorbance at selected wavelengths to a 1:1 binding isotherm allowing for guest absorption. d) Calculated populations of different species containing* ***DAO$_3$AD*** *and guest. e) Relationship between the RMSE between the experimental data and calculated spectra plotted as a function of the value of $K_{obs}$.*

# **DAO$_4$AD**•PFTB fitted to 1:1 binding isotherm

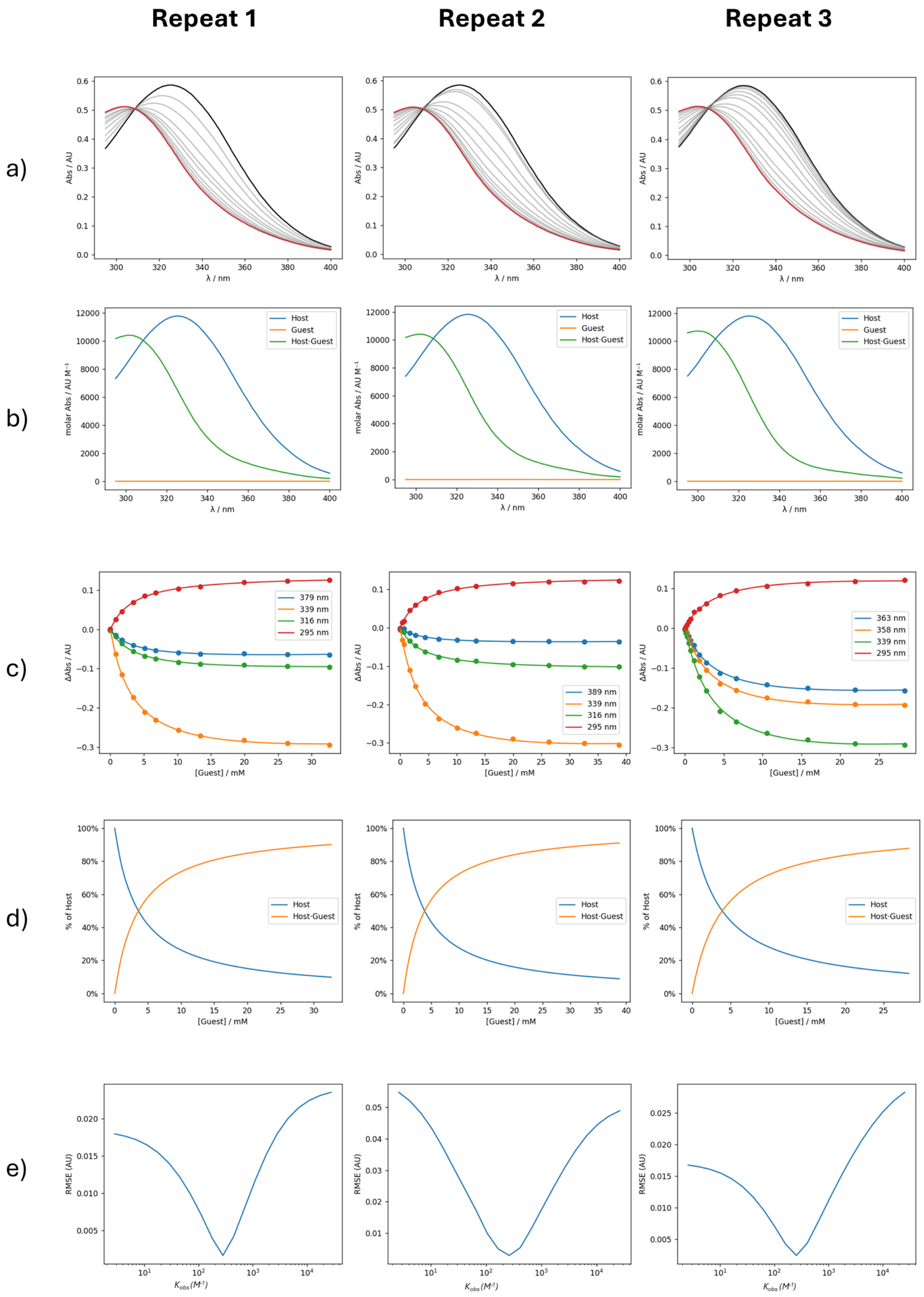


*Figure S 127 UV-Vis absorption titration of PFTB into **DAO$_4$AD** (50 µM) in dichloromethane at 298 K. a) UV-Vis absorption spectra showing free **DAO4AD** in black and final spectrum in red. b) Fitted spectra for **DAO$_4$AD** (host), **DAO$_4$AD** •PFTB (host•guest) an PFTB (guest) c) Best fit of the change in UV-Vis absorbance at selected wavelengths to a 1:1 binding isotherm allowing for guest absorption. d) Calculated populations of different species containing **DAO$_4$AD** and guest. e) Relationship between the RMSE between the experimental data and calculated spectra plotted as a function of the value of $K_{obs}$.*

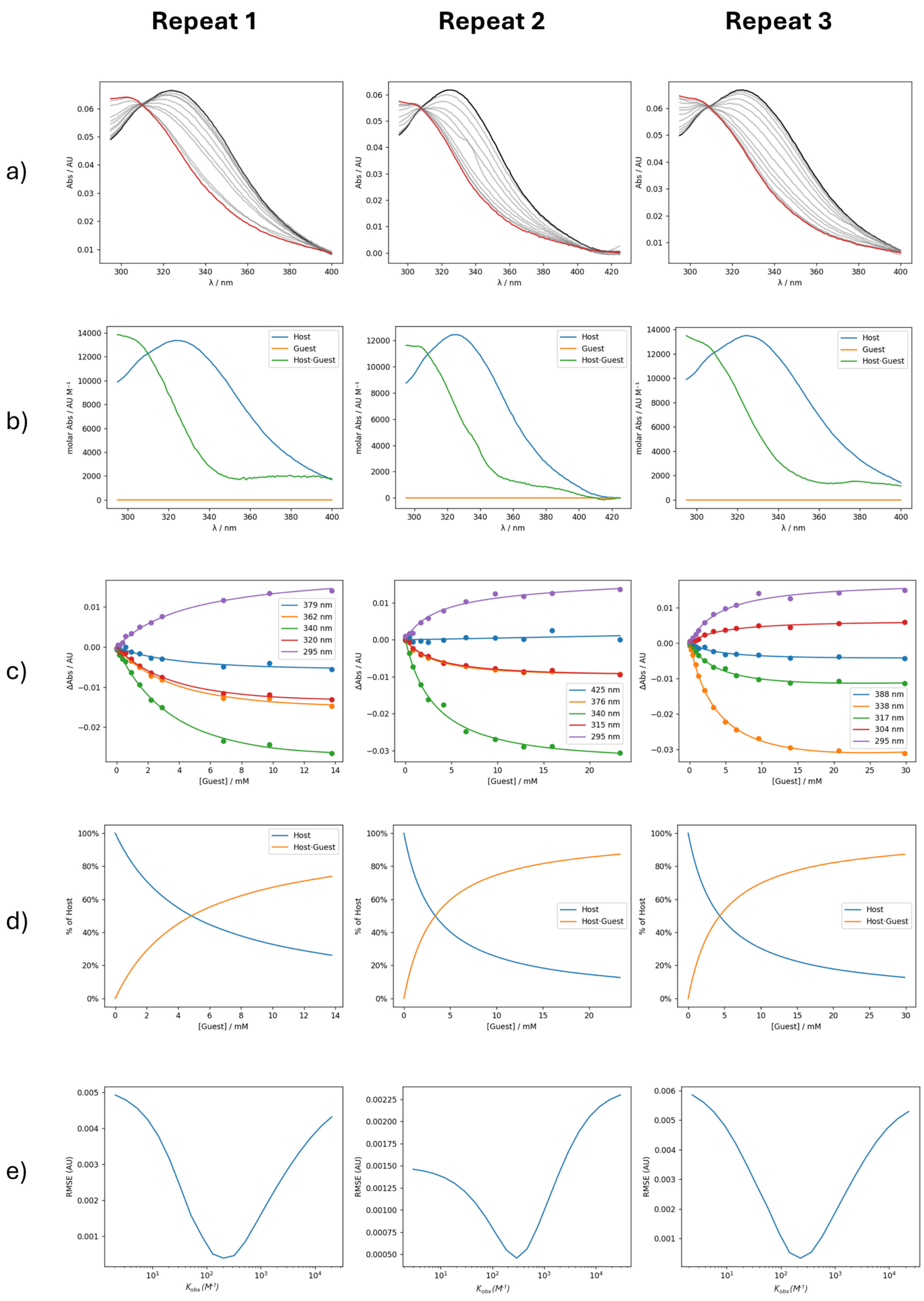


*Figure S 128 UV-Vis absorption titration of PFTB into **DAO$_4$AD** (5 µM) in dichloromethane at 298 K. a) UV-Vis absorption spectra showing free **DAO4AD** in black and final spectrum in red. b) Fitted spectra for **DAO$_4$AD** (host), **DAO$_4$AD** •PFTB (host•guest) an PFTB (guest) c) Best fit of the change in UV-Vis absorbance at selected wavelengths to a 1:1 binding isotherm allowing for guest absorption. d) Calculated populations of different species containing **DAO$_4$AD** and guest. e) Relationship between the RMSE between the experimental data and calculated spectra plotted as a function of the value of $K_{obs}$.*

# **ADO$_3$AD•**PFTB fitted to 1:1 binding isotherm

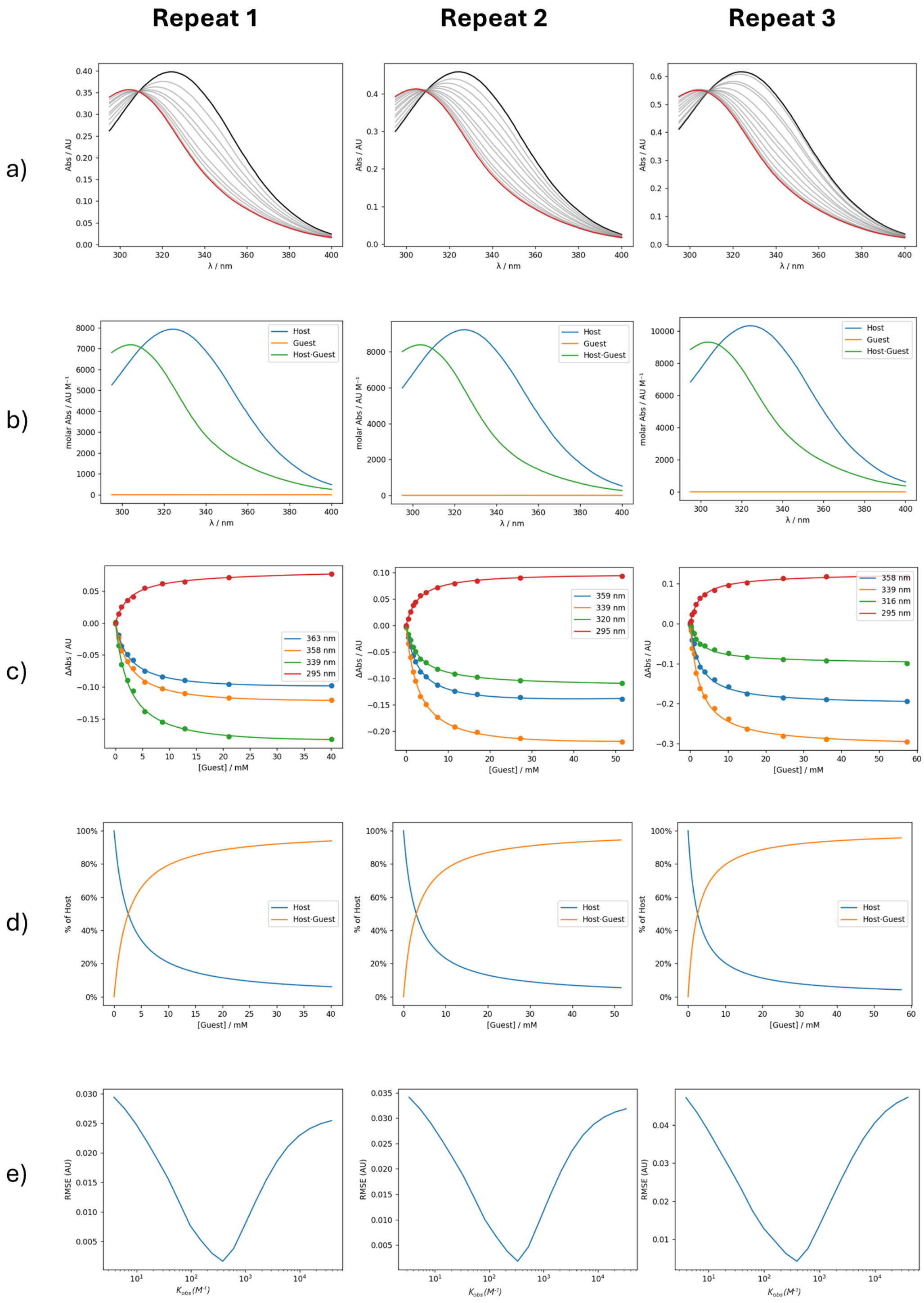


*Figure S 129 UV-Vis absorption titration of PFTB into **ADO$_3$AD** (50 µM) in dichloromethane at 298 K. a) UV-Vis absorption spectra showing free **ADO$_3$AD** in black and final spectrum in red. b) Fitted spectra for **ADO$_3$AD** (host), **ADO$_3$AD** •PFTB (host•guest) an PFTB (guest) c) Best fit of the change in UV-Vis absorbance at selected wavelengths to a 1:1 binding isotherm allowing for guest absorption. d) Calculated populations of different species containing **ADO$_3$AD** and guest. e) Relationship between the RMSE between the experimental data and calculated spectra plotted as a function of the value of $K_{obs}$.*

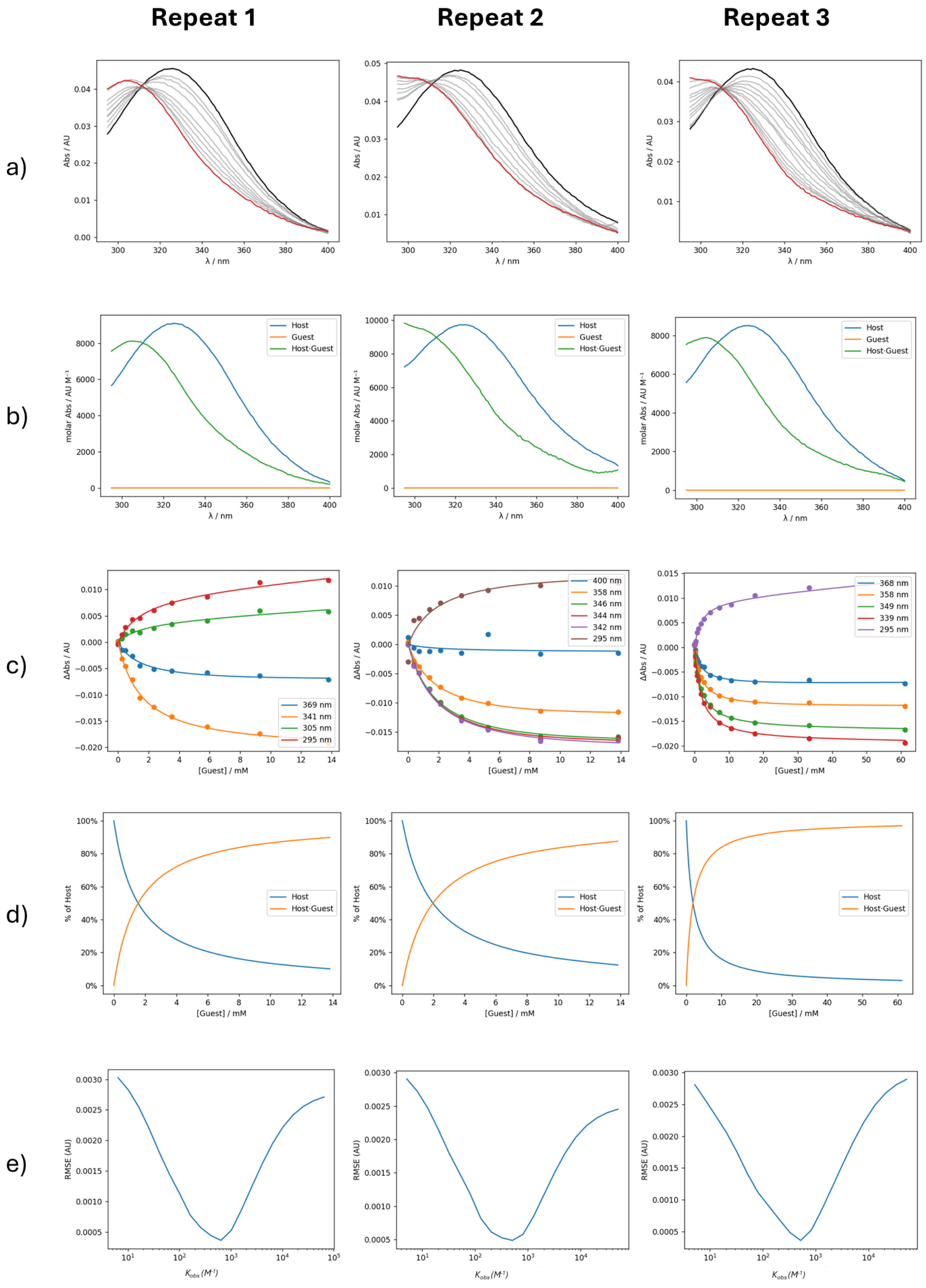


*Figure S 130 UV-Vis absorption titration of PFTB into **ADO₃AD** (5 µM) in dichloromethane at 298 K. a) UV-Vis absorption spectra showing free **ADO₃AD** in black and final spectrum in red. b) Fitted spectra for **ADO₃AD** (host), **ADO₃AD** •PFTB (host•guest) an PFTB (guest) c) Best fit of the change in UV-Vis absorbance at selected wavelengths to a 1:1 binding isotherm allowing for guest absorption. e) Relationship between the RMSE between the experimental data and calculated spectra plotted as a function of the value of $K_{obs}$.*

# **ADO$_4$AD**•PFTB fitted to 1:1 binding isotherm

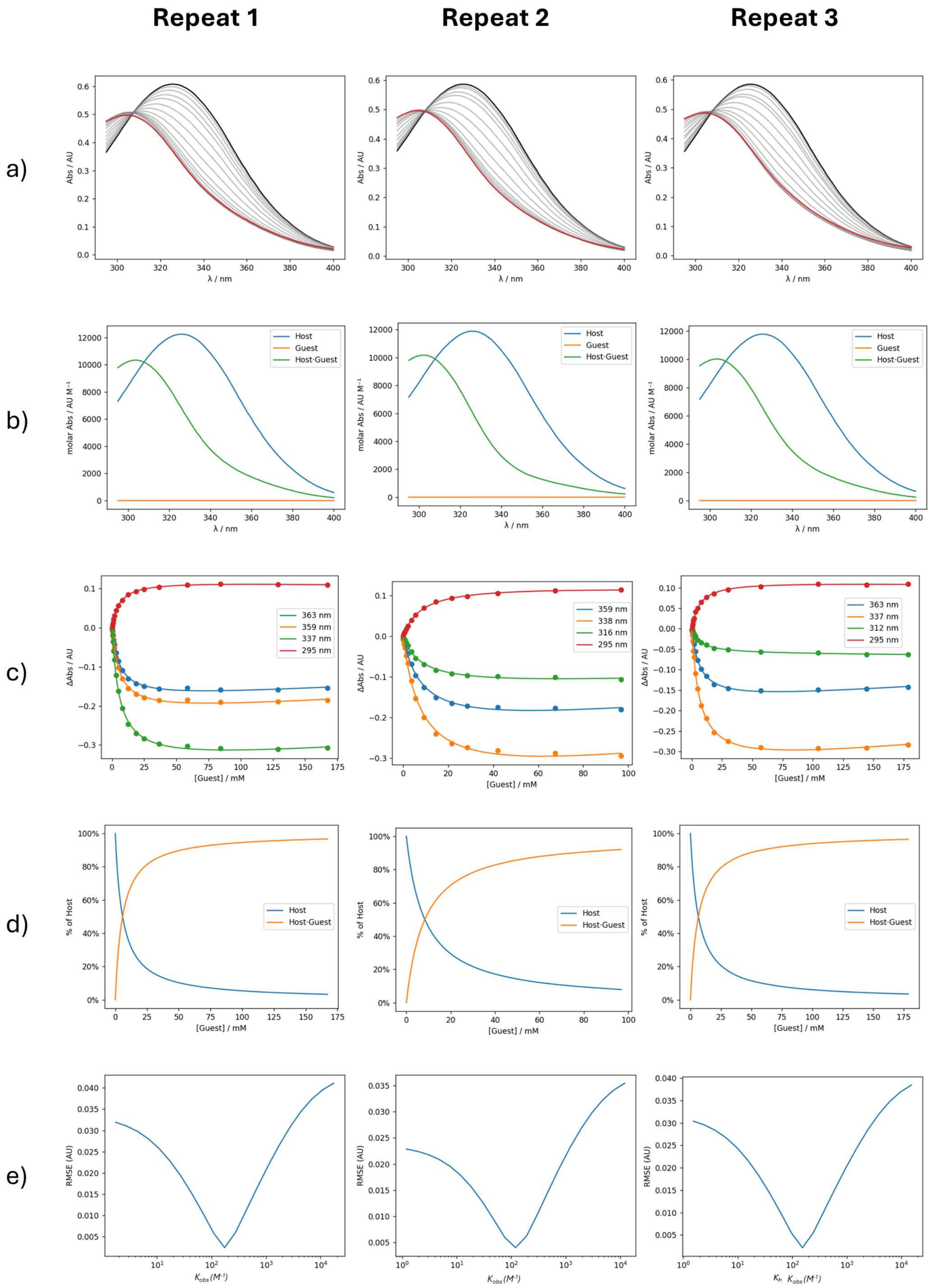


*Figure S 131 UV-Vis absorption titration of PFTB into* ***ADO$_4$AD*** *(50 µM) in dichloromethane at 298 K. a) UV-Vis absorption spectra showing free* ***ADO$_4$AD*** *in black and final spectrum in red. b) Fitted spectra for* ***ADO$_4$AD*** *(host),* ***ADO$_4$AD*** *•PFTB (host•guest) an PFTB (guest) c) Best fit of the change in UV-Vis absorbance at selected wavelengths to a 1:1 binding isotherm allowing for guest absorption. d) Calculated populations of different species containing* ***ADO$_4$AD*** *and guest. e) Relationship between the RMSE between the experimental data and calculated spectra plotted as a function of the value of $K_{obs}$.*

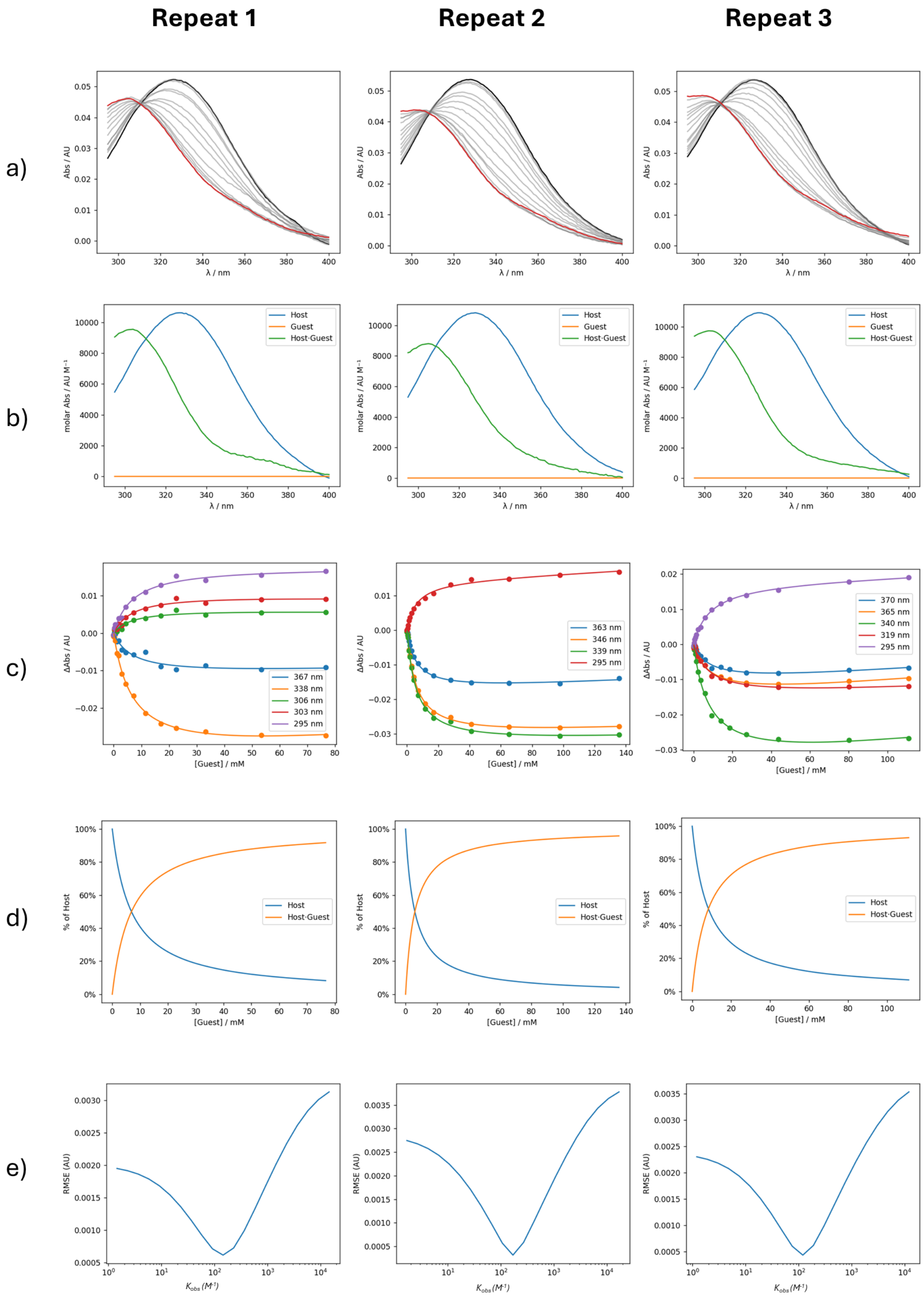


*Figure S 132 UV-Vis absorption titration of PFTB into **ADO$_4$AD** (5 µM) in dichloromethane at 298 K. a) UV-Vis absorption spectra showing free **ADO$_4$AD** in black and final spectrum in red. b) Fitted spectra for **ADO$_4$AD** (host), **ADO$_4$AD** •PFTB (host•guest) an PFTB (guest) c) Best fit of the change in UV-Vis absorbance at selected wavelengths to a 1:1 binding isotherm allowing for guest absorption. d) Calculated populations of different species containing **ADO$_4$AD** and guest. e) Relationship between the RMSE between the experimental data and calculated spectra plotted as a function of the value of $K_{obs}$.*

# ADO$_5$AD•PFTB fitted to 1:1 binding isotherm

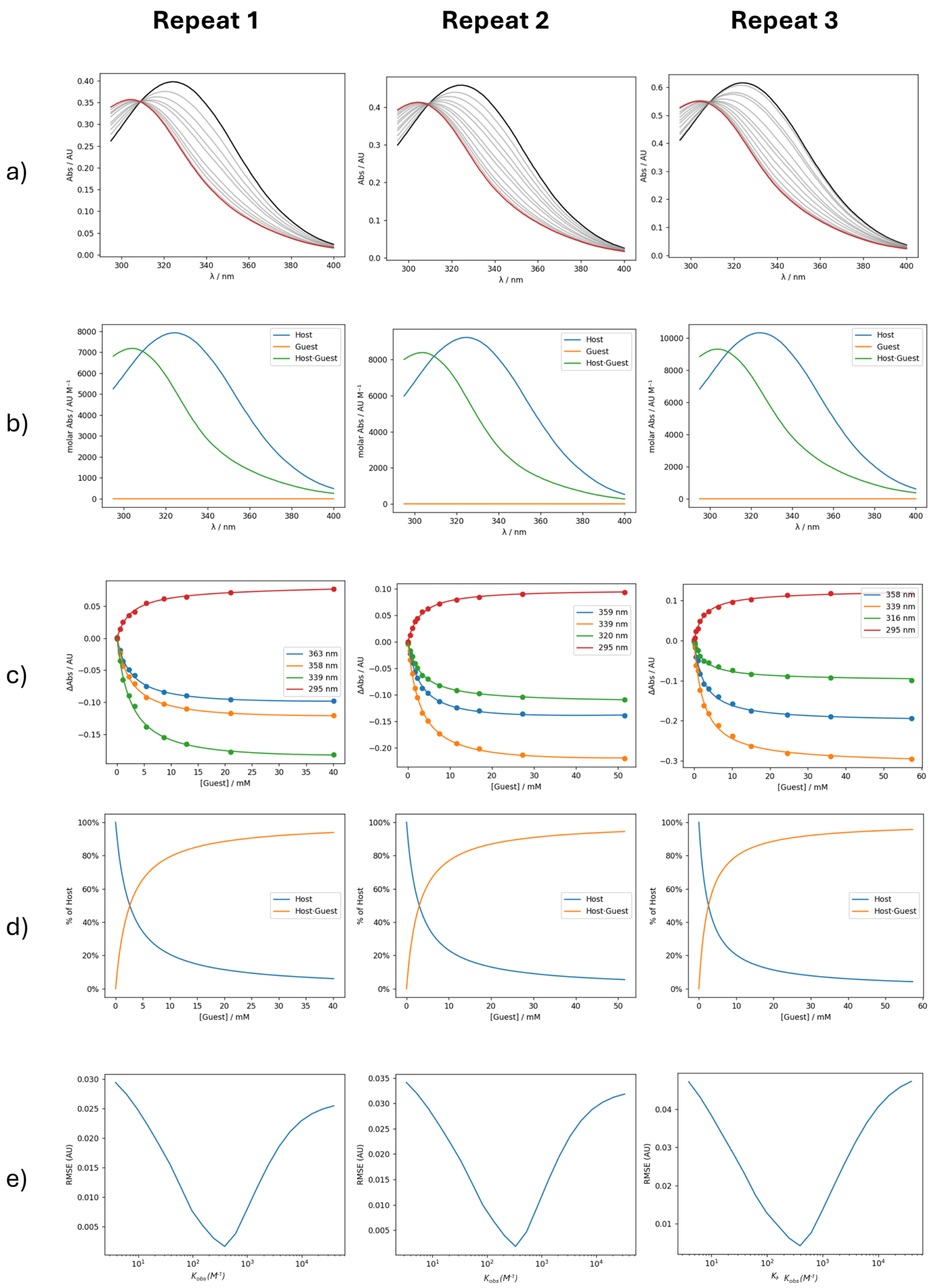


*Figure S 133 UV-Vis absorption titration of PFTB into* ***ADO$_5$AD*** *(50 µM) in dichloromethane at 298 K. a) UV-Vis absorption spectra showing free* ***ADO$_5$AD*** *in black and final spectrum in red. b) Fitted spectra for* ***ADO$_5$AD*** *(host),* ***ADO$_5$AD*** *•PFTB (host•guest) an PFTB (guest) c) Best fit of the change in UV-Vis absorbance at selected wavelengths to a 1:1 binding isotherm allowing for guest absorption. d) Calculated populations of different species containing* ***ADO$_5$AD*** *and guest. e) Relationship between the RMSE between the experimental data and calculated spectra plotted as a function of the value of* $K_{obs}$.

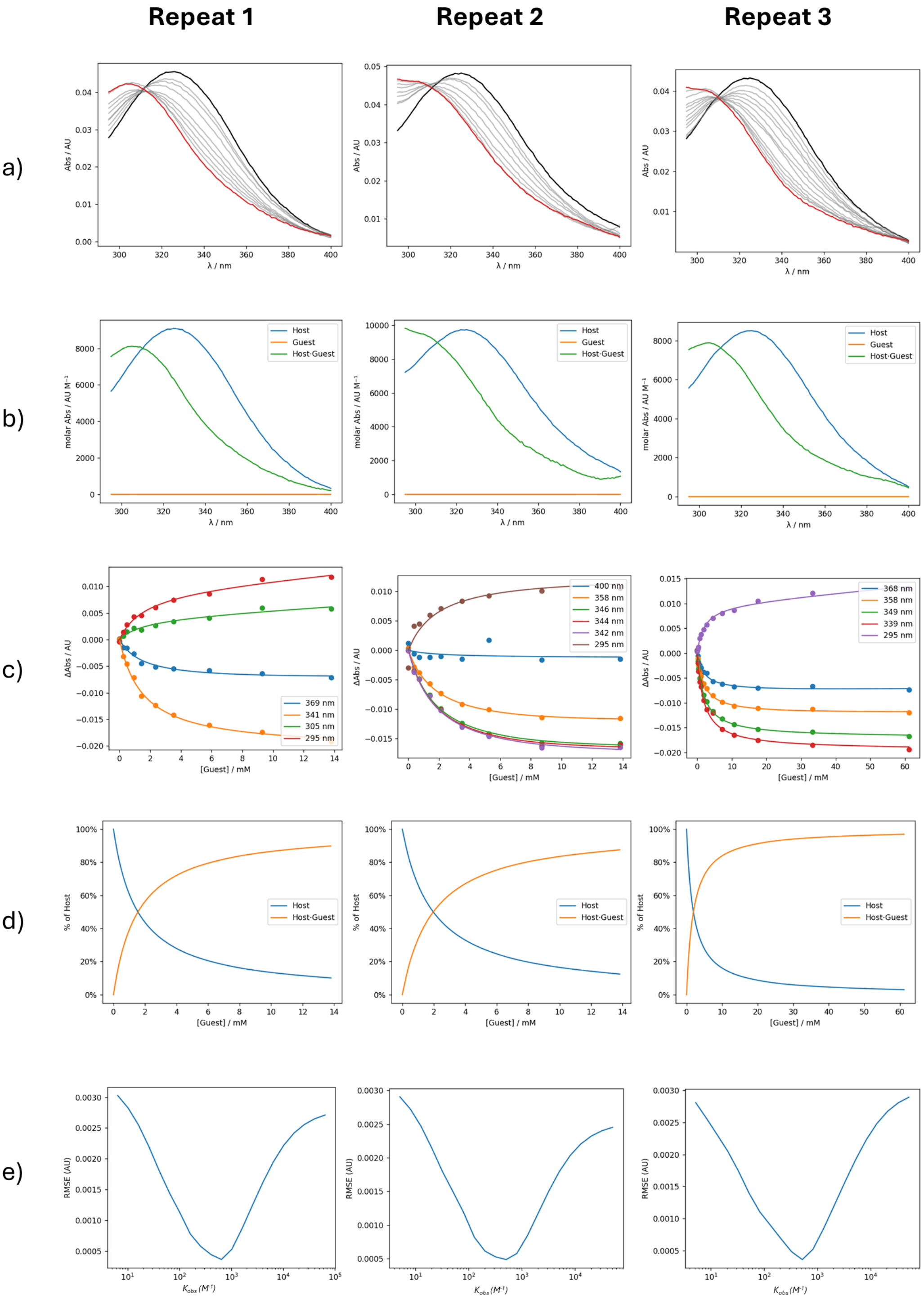


*Figure S 134 UV-Vis absorption titration of PFTB into* ***ADO$_5$AD*** *(5 µM) in dichloromethane at 298 K. a) UV-Vis absorption spectra showing free* ***ADO$_5$AD*** *in black and final spectrum in red. b) Fitted spectra for* ***ADO$_5$AD*** *(host),* ***ADO$_5$AD*** *•PFTB (host•guest) an PFTB (guest) c) Best fit of the change in UV-Vis absorbance at selected wavelengths to a 1:1 binding isotherm allowing for guest absorption. d) Calculated populations of different species containing* ***ADO$_5$AD*** *and guest. e) Relationship between the RMSE between the experimental data and calculated spectra plotted as a function of the value of $K_{obs}$.*

# **DAO$_n$AD**•PFTB and **ADO$_n$AD**•PFTB fitted to duplex model

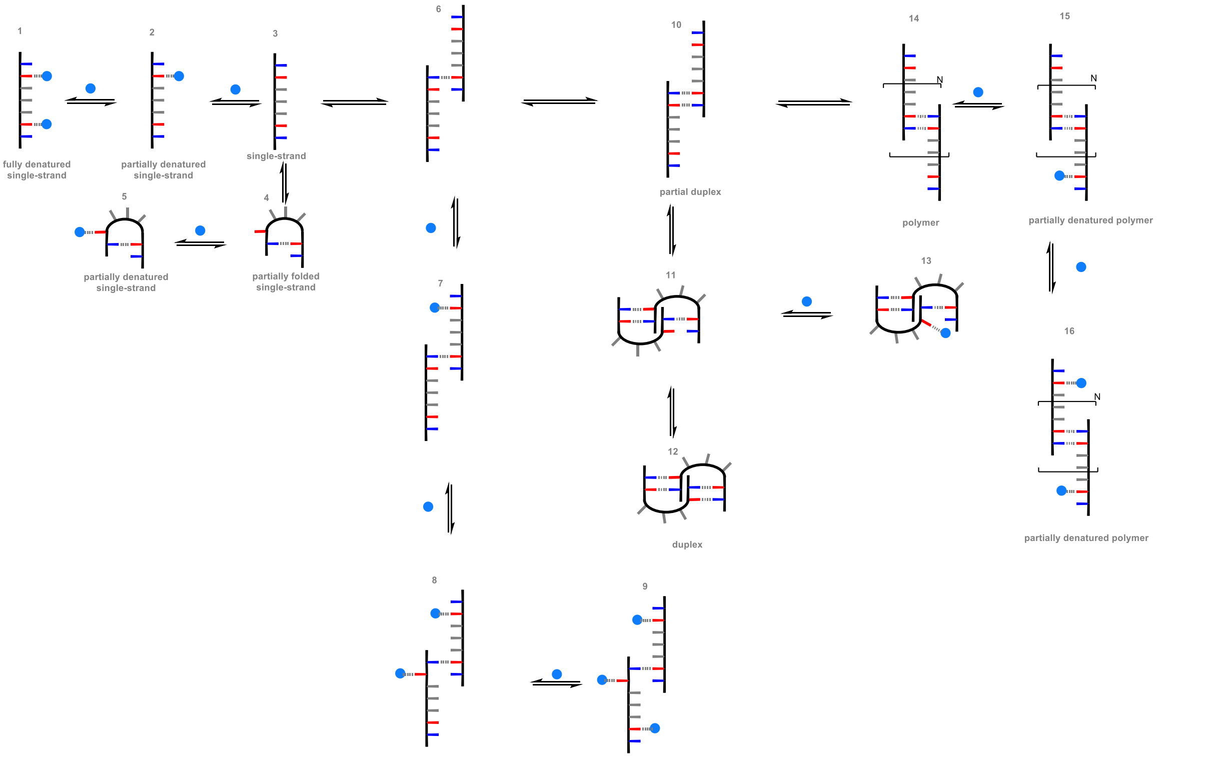


*Figure S 135 The duplex model: H-bonding equilibria between* ***DAO$_n$AD*** *and* ***ADO$_n$AD*** *(n = 2 to 8) and PFTB (blue circle) allowing for no full folding and only duplex formation, and naming system used in the Musketeer fitting. Only one isomer for each species shown.* ***DAO$_3$AD*** *shown as example.*

*Table S 5 Summary of equations used in the Musketeer fit of PFTB titration into* $\mathbf{DAO_nAD}$ *and* $\mathbf{ADO_nAD}$ *) using the duplex model allowing for no full folding and only duplex formation.*

| Species formed | Global K | Extinction coefficient as a function of extinction coefficients of free 4-nitrophenol ($\varepsilon_{free}$) and bound 4-nitrophenol ($\varepsilon_{bound}$) |
|---|---|---|
| 1 | - | 2 $\varepsilon_{free}$ + 0 $\varepsilon_{bound}$ |
| 2 | 2 $K_d$ | 2 $\varepsilon_{free}$ + 0 $\varepsilon_{bound}$ |
| 3 | $K_d^2$ | 2 $\varepsilon_{free}$ + 0 $\varepsilon_{bound}$ |
| 4 | $K_{A\bullet D}$ $(EM_{f1} + EM_{f2})$ | 1 $\varepsilon_{free}$ + 1 $\varepsilon_{bound}$ |
| 5 | $K_{A\bullet D}$ $(EM_{f1} + EM_{f2})$ $K_d$ | 1 $\varepsilon_{free}$ + 1 $\varepsilon_{bound}$ |
| 6 | 8 $K_{A\bullet D}$ | 3 $\varepsilon_{free}$ + 1 $\varepsilon_{bound}$ |
| 7 | 24 $K_{A\bullet D}$ $K_d$ | 3 $\varepsilon_{free}$ + 1 $\varepsilon_{bound}$ |
| 8 | 24 $K_{A\bullet D}$ $K_d^2$ | 3 $\varepsilon_{free}$ + 1 $\varepsilon_{bound}$ |
| 9 | 8 $K_{A\bullet D}$ $K_d^3$ | 3 $\varepsilon_{free}$ + 1 $\varepsilon_{bound}$ |
| 10 | 4 $K_{A\bullet D}^2$ $EM_d$ | 2 $\varepsilon_{free}$ + 2 $\varepsilon_{bound}$ |
| 11 | 8 $K_{A\bullet D}^3$ $EM_d EM_{d2}$ | 1 $\varepsilon_{free}$ + 3 $\varepsilon_{bound}$ |
| 12 | 2 $K_{A\bullet D}^4$ $EM_d^{\,2} EM_{d2}$ | 0 $\varepsilon_{free}$ + 4 $\varepsilon_{bound}$ |
| 13 | 8 $K_{A\bullet D}^3$ $EM_d EM_{d2}$ $K_d$ | 1 $\varepsilon_{free}$ + 3 $\varepsilon_{bound}$ |
| 14 | 2 $K_{A\bullet D}^{4N}$ $EM_d^{2N}$ | 2 $\varepsilon_{free}$ + 2N $\varepsilon_{bound}$ |
| 15 | 4 $K_{A\bullet D}^{4N}$ $EM_d^{2N}$ $K_d$ | 2 $\varepsilon_{free}$ + 2N $\varepsilon_{bound}$ |
| 16 | 2 $K_{A\bullet D}^{4N}$ $EM_d^{2N}$ $K_d^2$ | 2 $\varepsilon_{free}$ + 2N $\varepsilon_{bound}$ |

# **DAO$_3$AD**•PFTB fitted to duplex model

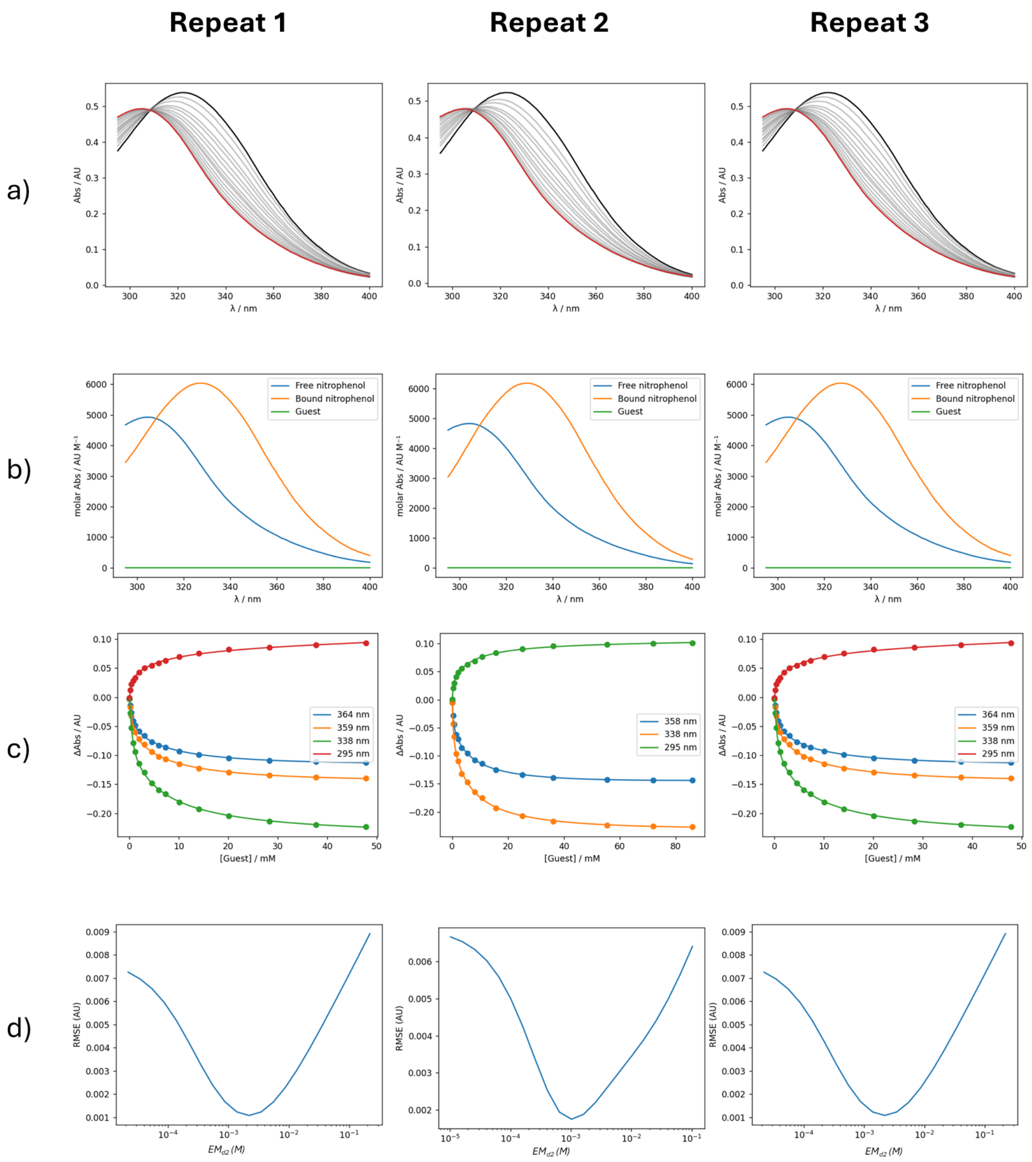


*Figure S 136 UV-Vis absorption denaturation of* ***DAO$_3$AD*** *(50 µM) with PFTB (guest) in dichloromethane at 298 K. a) UV-Vis absorption spectra showing the starting spectrum in black and the final spectrum in red. b) Fitted spectra of free 4-nitrohenol, bound 4-nitrophenol and PFTB c) Best fit of the change in UV-Vis absorbance at selected wavelengths to an 16-species isotherm as described in the duplex model in Figure S 135 and Table S 5 allowing for guest absorption. d) Relationship between the RMSE between the experimental data and calculated spectra plotted as a function of the value of* ***$EM_{d2}$****.*

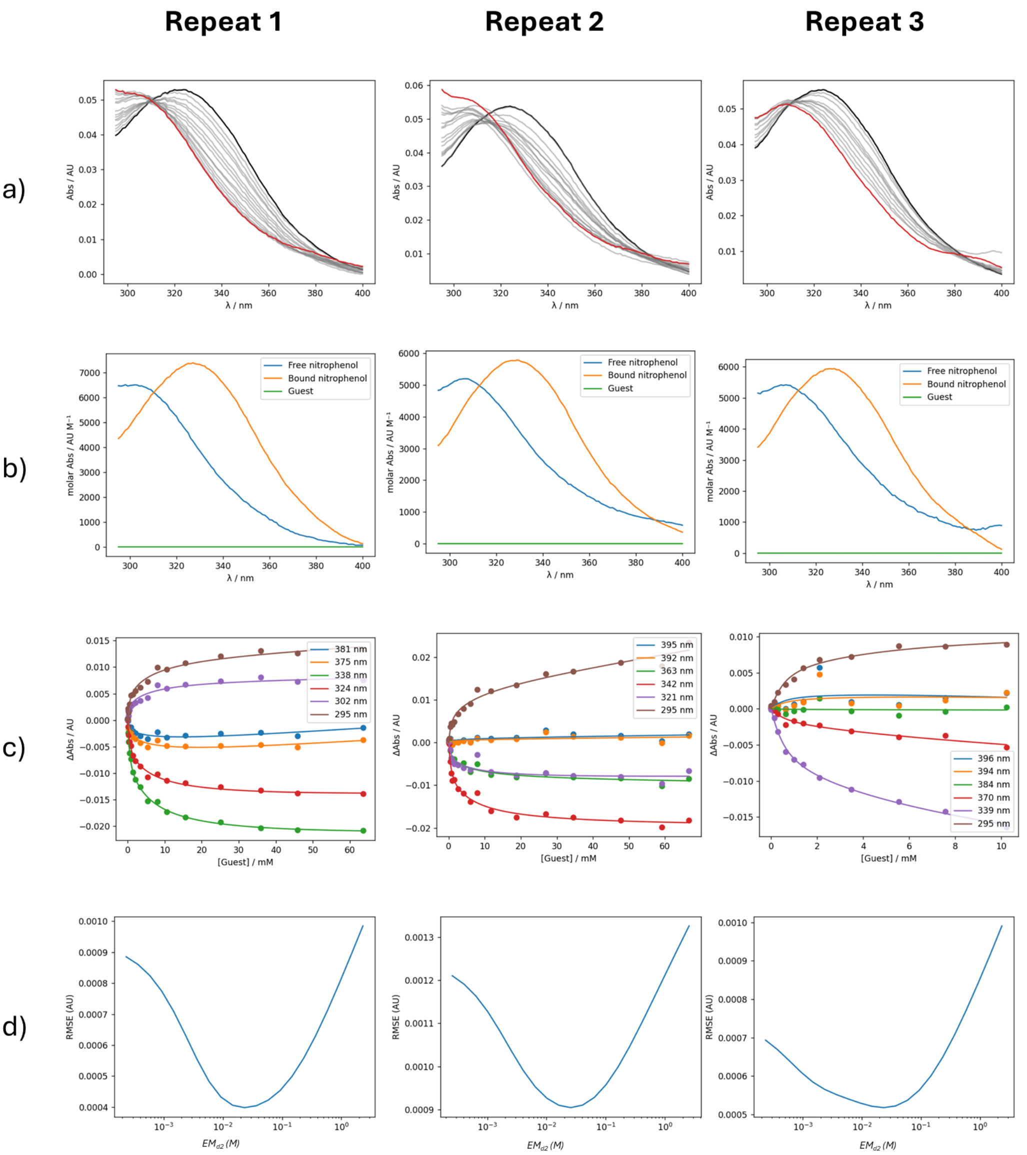


*Figure S 137 UV-Vis absorption denaturation of **$DAO_3AD$** (5 µM) with PFTB (guest) in dichloromethane at 298 K. a) UV-Vis absorption spectra showing the starting spectrum in black and the final spectrum in red. b) Fitted spectra of free 4-nitrohenol, bound 4-nitrophenol and PFTB c) Best fit of the change in UV-Vis absorbance at selected wavelengths to an 16-species isotherm as described in the duplex model in Figure S 136 and Table S 5 allowing for guest absorption. d) Relationship between the RMSE between the experimental data and calculated spectra plotted as a function of the value of **$EM_{d2}$**.*

# **DAO$_4$AD**•PFTB fitted to duplex model

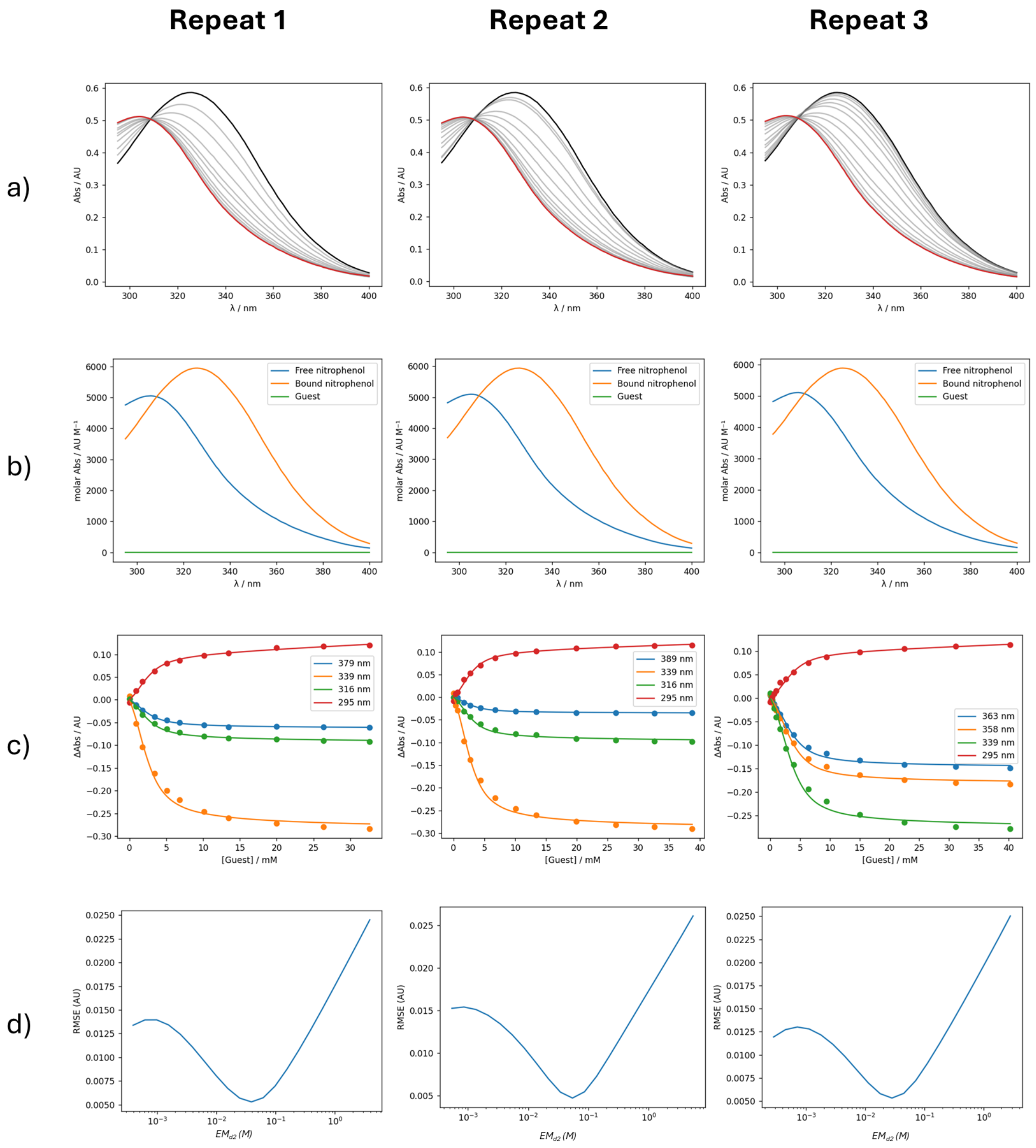


*Figure S 138 UV-Vis absorption denaturation of* ***DAO$_4$AD*** *(50 µM) with PFTB (guest) in dichloromethane at 298 K. a) UV-Vis absorption spectra showing the starting spectrum in black and the final spectrum in red. b) Fitted spectra of free4-nitrohenol, bound 4-nitrophenol and PFTB c) Best fit of the change in UV-Vis absorbance at selected wavelengths to an 16-species isotherm as described in the duplex model in Figure S 135 and Table S 5 allowing for guest absorption. d) Relationship between the RMSE between the experimental data and calculated spectra plotted as a function of the value of* ***EM$_{d2}$****.*

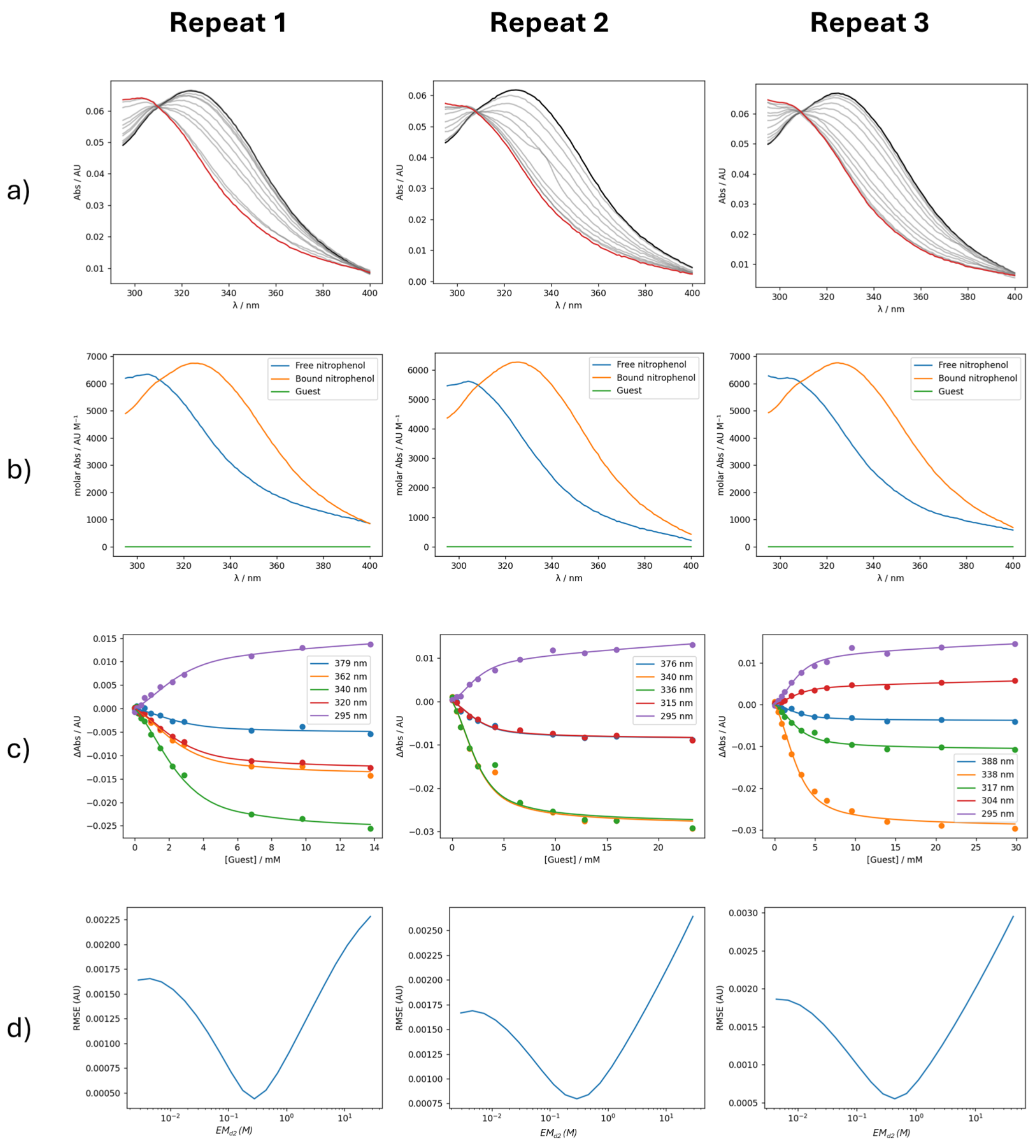


*Figure S 139 UV-Vis absorption denaturation of **DAO$_4$AD** (50 µM) with PFTB (guest) in dichloromethane at 298 K. a) UV-Vis absorption spectra showing the starting spectrum in black and the final spectrum in red. b) Fitted spectra of free 4-nitrohenol, bound 4-nitrophenol and PFTB c) Best fit of the change in UV-Vis absorbance at selected wavelengths to an 16-species isotherm as described in the duplex model in Figure S 136 and Table S 5 allowing for guest absorption. d) Relationship between the RMSE between the experimental data and calculated spectra plotted as a function of the value of **EM$_{d2}$**.*

# **ADO$_3$AD**•PFTB fitted to duplex model

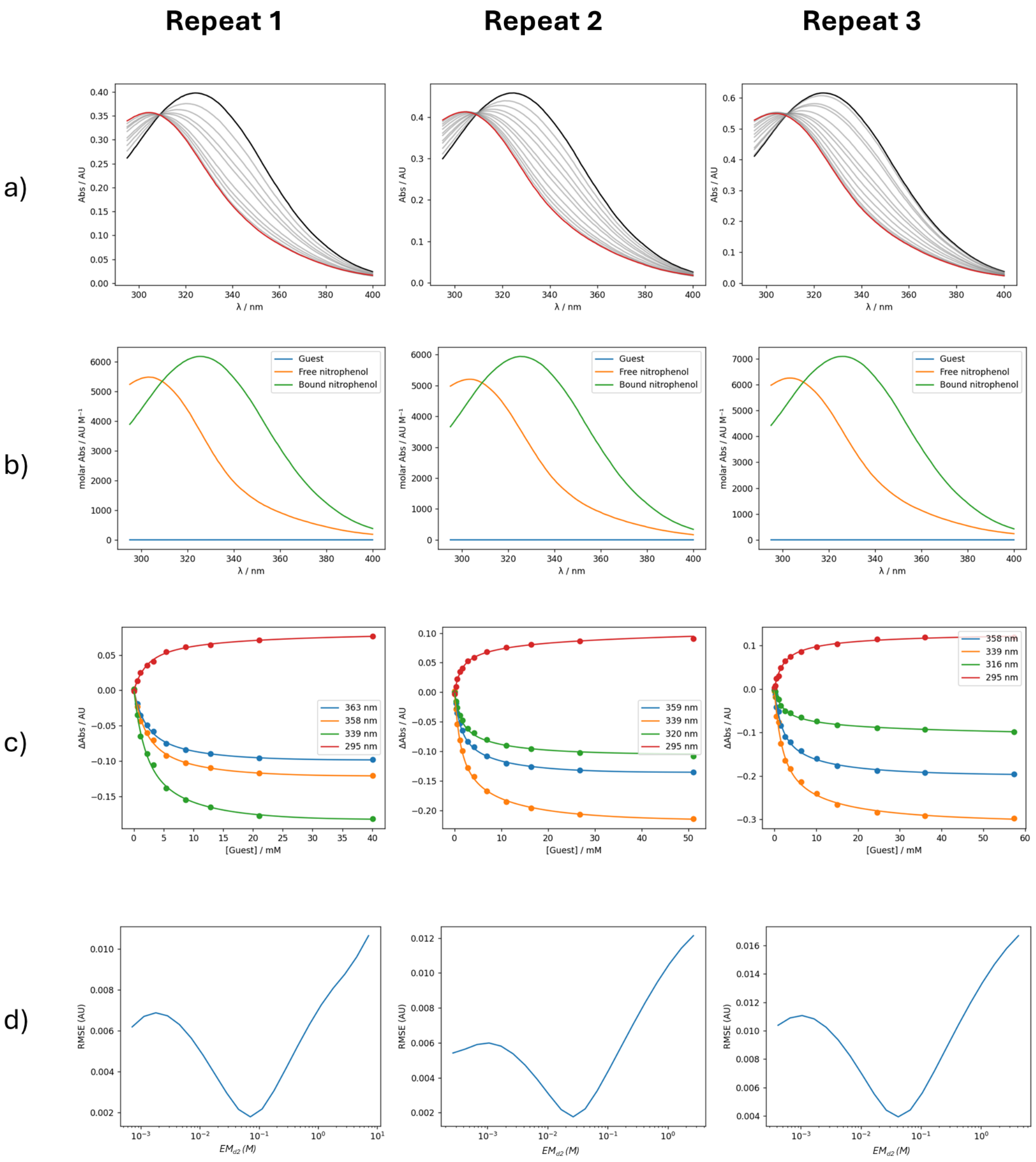


*Figure S 140 UV-Vis absorption denaturation of **ADO$_3$AD** (50 µM) with PFTB (guest) in dichloromethane at 298 K. a) UV-Vis absorption spectra showing the starting spectrum in black and the final spectrum in red. b) Fitted spectra of free 4-nitrohenol, bound 4-nitrophenol and PFTB c) Best fit of the change in UV-Vis absorbance at selected wavelengths to an 16-species isotherm as described in the duplex model in Figure S 135 and Table S 5 allowing for guest absorption. d) Relationship between the RMSE between the experimental data and calculated spectra plotted as a function of the value of **EM$_{d2}$**.*

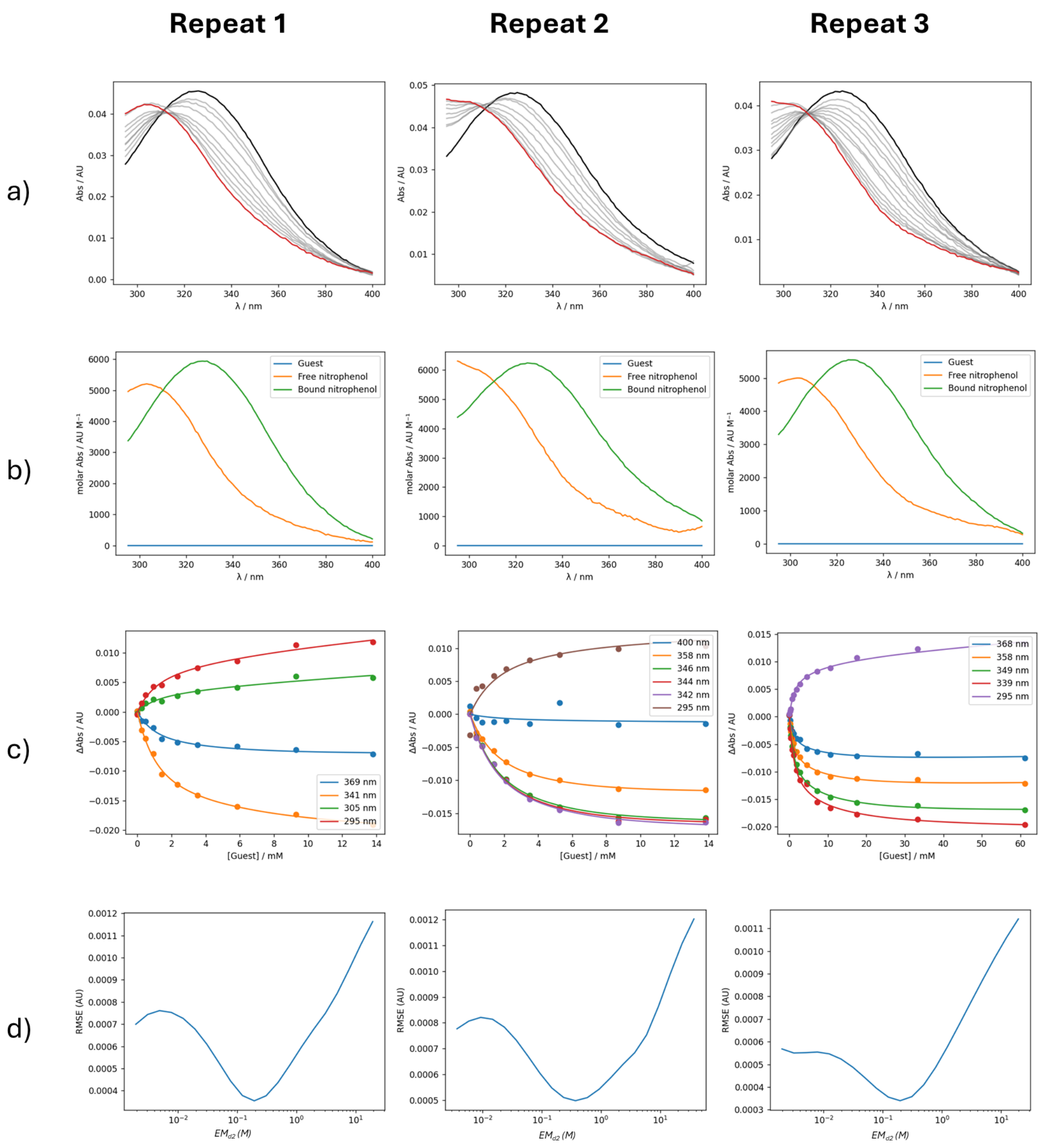


*Figure S 141 UV-Vis absorption denaturation of* ***$ADO_3AD$*** *(5 µM) with PFTB (guest) in dichloromethane at 298 K. a) UV-Vis absorption spectra showing the starting spectrum in black and the final spectrum in red. b) Fitted spectra of free 4-nitrohenol, bound 4-nitrophenol and PFTB c) Best fit of the change in UV-Vis absorbance at selected wavelengths to an 16-species isotherm as described in the duplex model in Figure S 135 and Table S 5 allowing for guest absorption. d) Relationship between the RMSE between the experimental data and calculated spectra plotted as a function of the value of* ***$EM_{d2}$***.

# **ADO$_4$AD**•PFTB fitted to duplex model

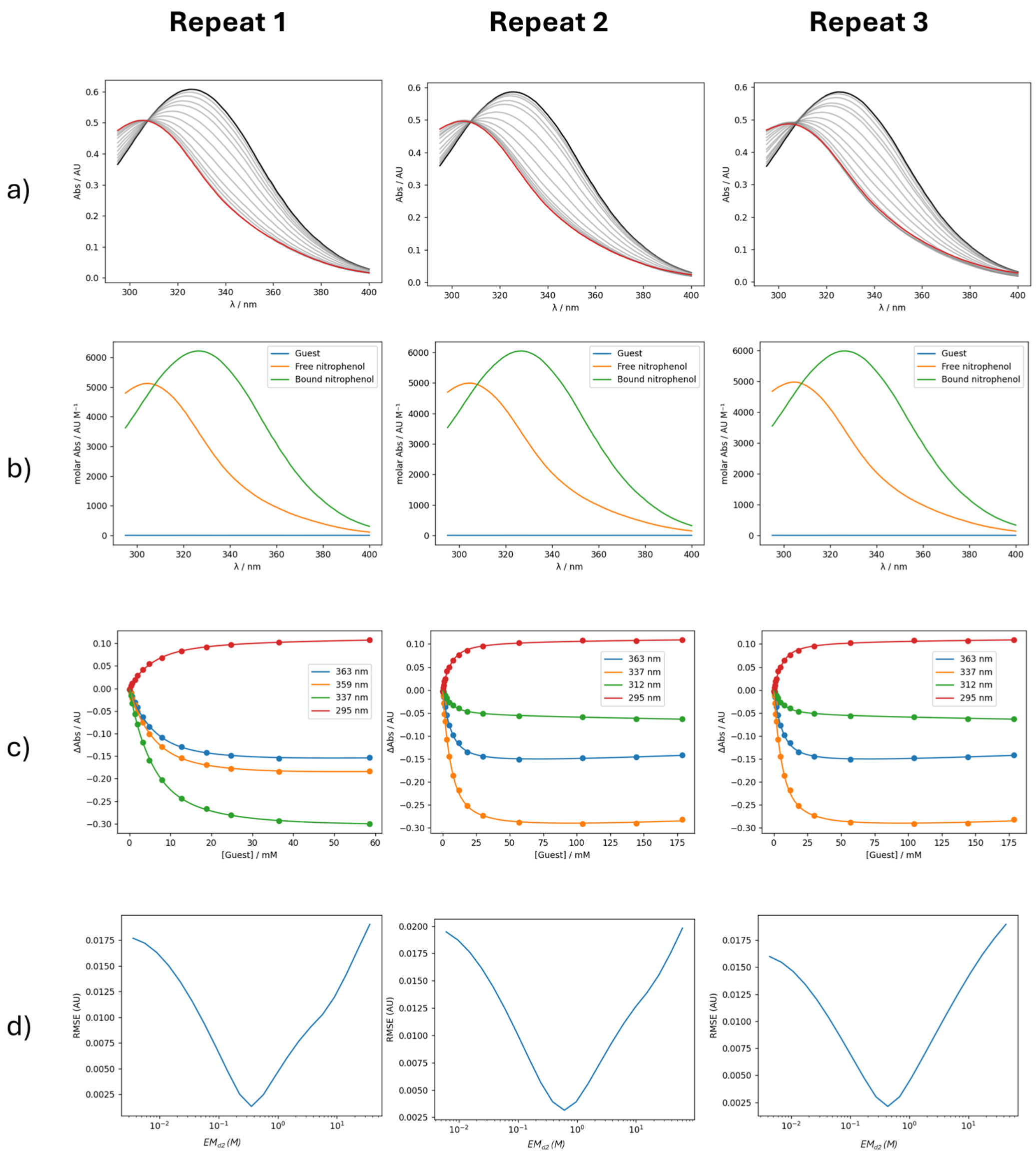


*Figure S 142 UV-Vis absorption denaturation of **ADO$_4$AD** (50 µM) with PFTB (guest) in dichloromethane at 298 K. a) UV-Vis absorption spectra showing the starting spectrum in black and the final spectrum in red. b) Fitted spectra of free 4-nitrohenol, bound 4-nitrophenol and PFTB c) Best fit of the change in UV-Vis absorbance at selected wavelengths to an 16-species isotherm as described in the duplex model in Figure S 135 and Table S 5 allowing for guest absorption. d) Relationship between the RMSE between the experimental data and calculated spectra plotted as a function of the value of* $\boldsymbol{EM_{d2}}$.

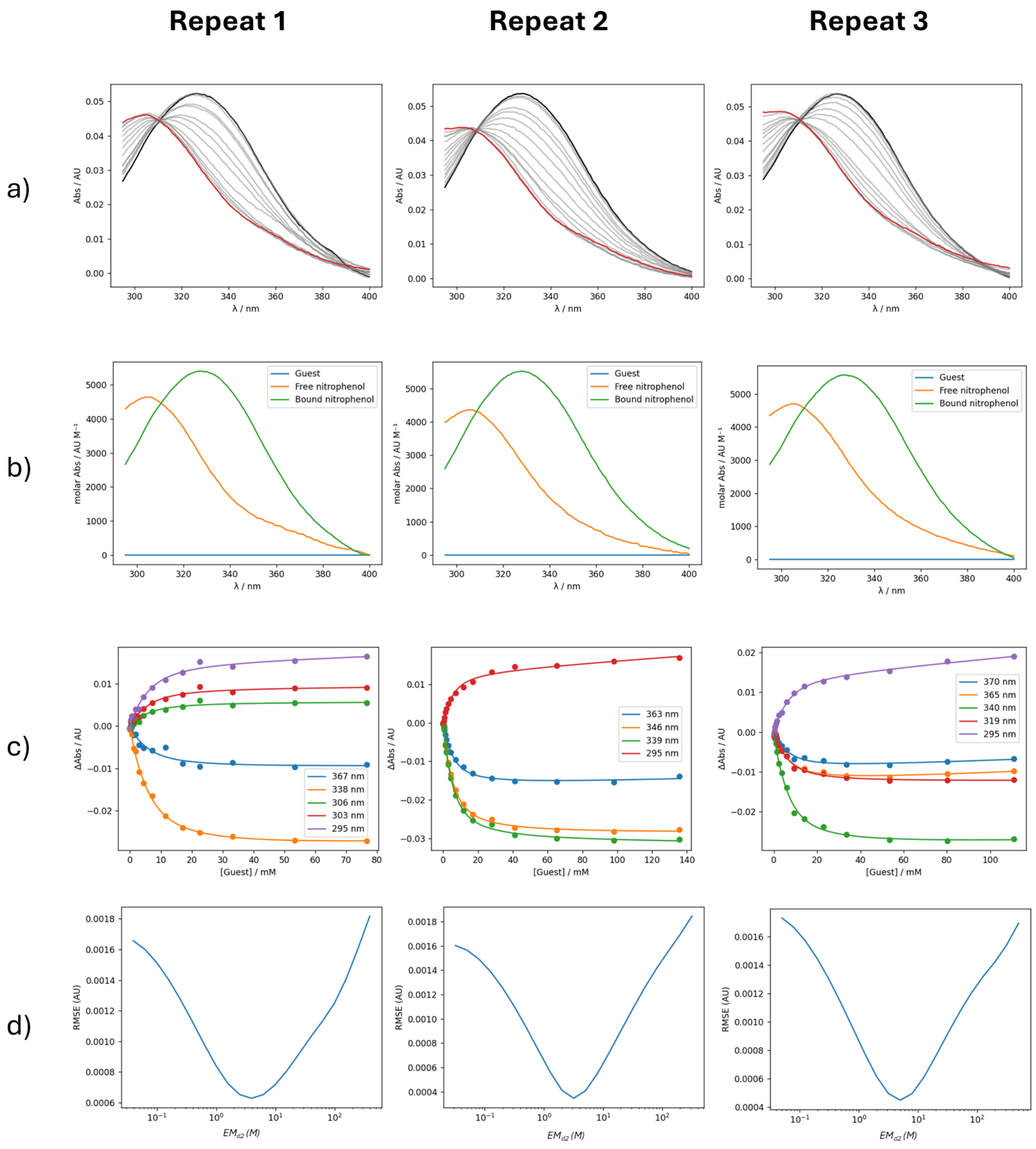


*Figure S 143 UV-Vis absorption denaturation of* ***ADO$_4$AD*** *(5 µM) with PFTB (guest) in dichloromethane at 298 K. a) UV-Vis absorption spectra showing the starting spectrum in black and the final spectrum in red. b) Fitted spectra of free 4-nitrohenol, bound 4-nitrophenol and PFTB c)Best fit of the change in UV-Vis absorbance at selected wavelengths to an 16-species isotherm as described in the duplex model in Figure S 135 and Table S 5 allowing for guest absorption. d) Relationship between the RMSE between the experimental data and calculated spectra plotted as a function of the value of* ***$EM_{d2}$****.*

# ADO₅AD•PFTB fitted to duplex model

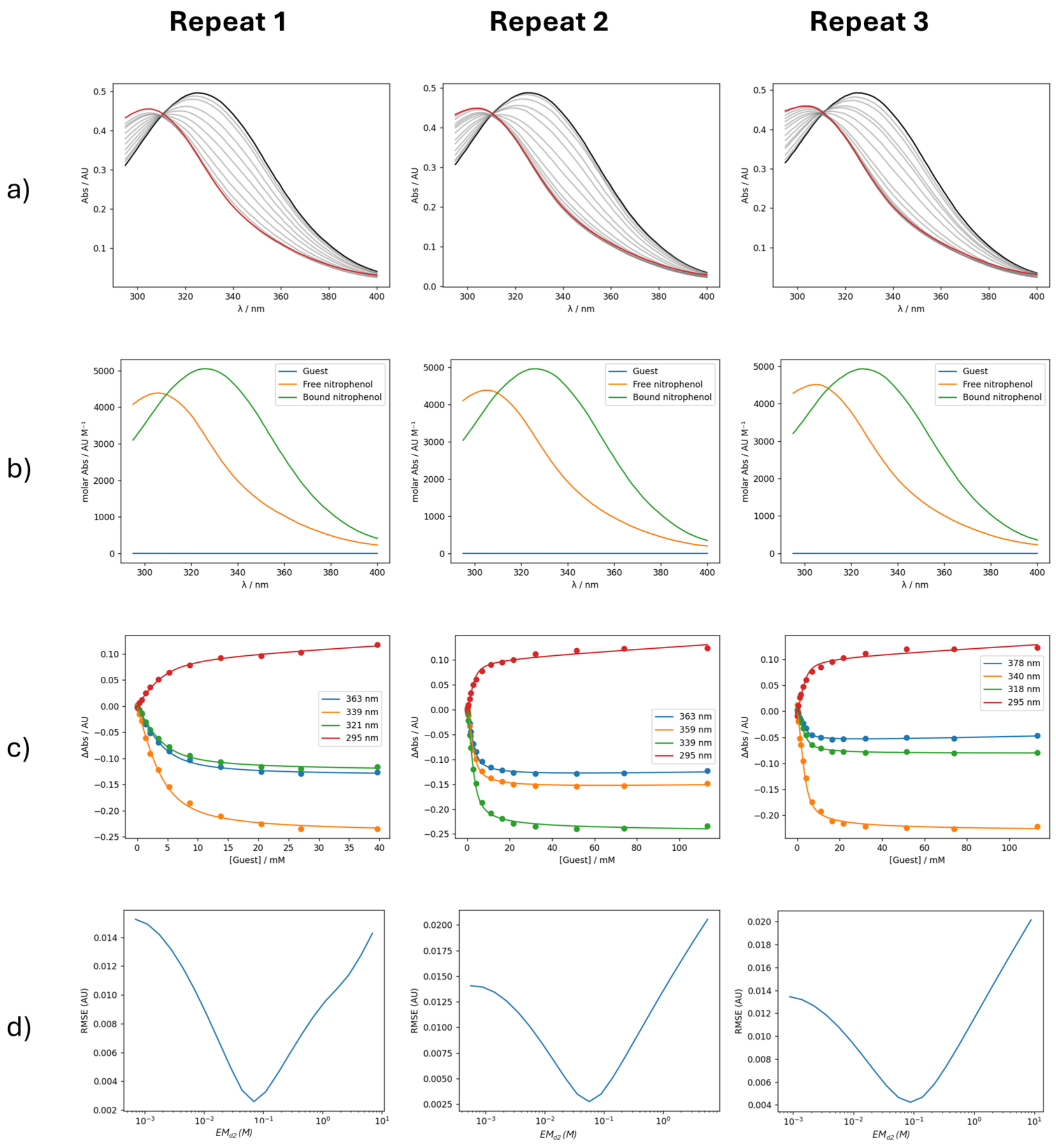


*Figure S 144 UV-Vis absorption denaturation of **$ADO_5AD$** (50 µM) with PFTB (guest) in dichloromethane at 298 K. a) UV-Vis absorption spectra showing the starting spectrum in black and the final spectrum in red. b) Fitted spectra of free 4-nitrohenol, bound 4-nitrophenol and PFTB c) Best fit of the change in UV-Vis absorbance at selected wavelengths to an 16-species isotherm as described in the duplex model in Figure S 136 and Table S 5 allowing for guest absorption. d) Relationship between the RMSE between the experimental data and calculated spectra plotted as a function of the value of **$EM_{d2}$**.*

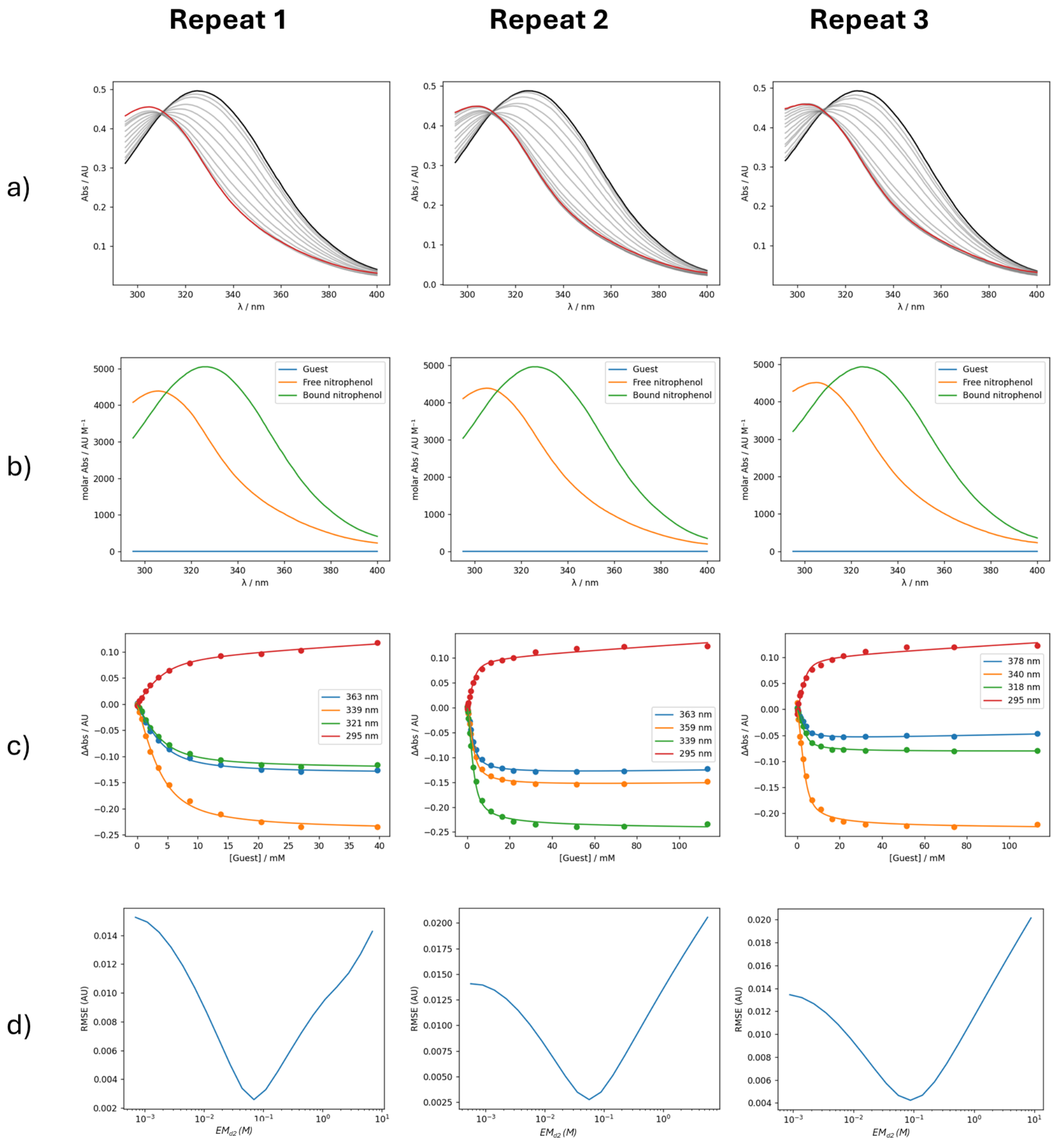


*Figure S 145 UV-Vis absorption denaturation of **ADO$_5$AD** (5 μM) with PFTB (guest) in dichloromethane at 298 K. a) UV-Vis absorption spectra showing the starting spectrum in black and the final spectrum in red. b) Fitted spectra of free 4-nitrohenol, bound 4-nitrophenol and PFTB c) Best fit of the change in UV-Vis absorbance at selected wavelengths to an 16-species isotherm as described in the duplex model in Figure S 135 and Table S 5 allowing for guest absorption. d) Relationship between the RMSE between the experimental data and calculated spectra plotted as a function of the value of **EM$_{d2}$**.*

# **DAO$_n$AD**•PFTB and **ADO$_n$AD**•PFTB fitted to folding model

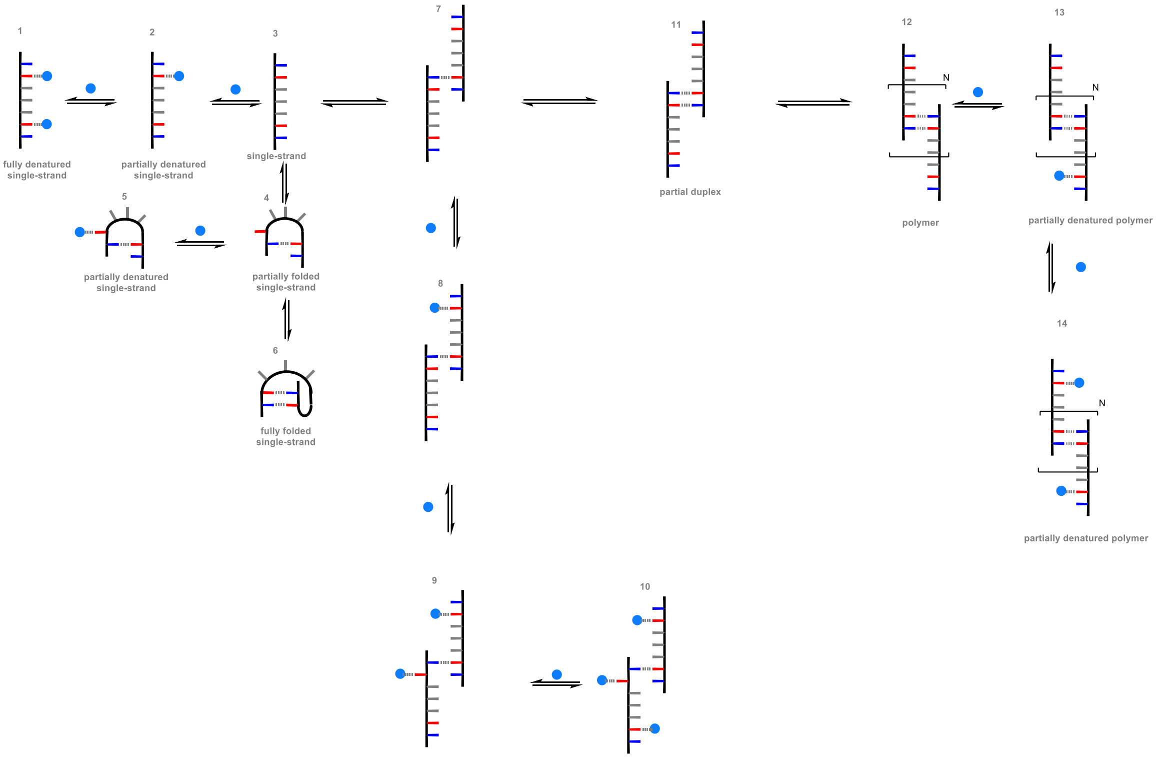


*Figure S 146 H-bonding equilibria between **DAO$_n$AD** and **ADO$_n$AD** (n = 2 to 8) and PFTB (blue circle) allowing for full folding and no duplex formation, and naming system used in the Musketeer fitting. Only one isomer for each species shown. **DAO$_3$AD** shown as example.*

*Table S 6 Summary of equations used in the Musketeer fit of PFTB titration into **DAO$_n$AD** and **ADO$_n$AD**) allowing for full folding and no duplex formation.*

| Species formed | Global K | Extinction coefficient as a function of extinction coefficients of free 4-nitrophenol ($\varepsilon_{free}$) and bound 4-nitrophenol ($\varepsilon_{bound}$) |
|---|---|---|
| 1 | - | $2\ \varepsilon_{free} + 0\ \varepsilon_{bound}$ |
| 2 | $2\ \boldsymbol{K_d}$ | $2\ \varepsilon_{free} + 0\ \varepsilon_{bound}$ |
| 3 | $\boldsymbol{K_d^2}$ | $2\ \varepsilon_{free} + 0\ \varepsilon_{bound}$ |
| 4 | $\boldsymbol{K_{A\bullet D}\ (EM_{f1} + EM_{f2})}$ | $1\ \varepsilon_{free} + 1\ \varepsilon_{bound}$ |
| 5 | $\boldsymbol{K_{A\bullet D}\ (EM_{f1} + EM_{f2})\ K_d}$ | $1\ \varepsilon_{free} + 1\ \varepsilon_{bound}$ |
| 6[1] | $\boldsymbol{K_{A\bullet D}{}^2\ (EM_{f1} + EM_{f2})\ EM_f'}$ <br> $\boldsymbol{= K_{A\bullet D}{}^2\ EM_{f1}\ EM_{f2}\ \alpha}$ | $0\ \varepsilon_{free} + 2\ \varepsilon_{bound}$ |
| 7 | $8\ \boldsymbol{K_{A\bullet D}}$ | $3\ \varepsilon_{free} + 1\ \varepsilon_{bound}$ |
| 8 | $24\ \boldsymbol{K_{A\bullet D}\ K_d}$ | $3\ \varepsilon_{free} + 1\ \varepsilon_{bound}$ |
| 9 | $24\ \boldsymbol{K_{A\bullet D}\ K_d^2}$ | $3\ \varepsilon_{free} + 1\ \varepsilon_{bound}$ |
| 10 | $8\ \boldsymbol{K_{A\bullet D}\ K_d^3}$ | $3\ \varepsilon_{free} + 1\ \varepsilon_{bound}$ |
| 11 | $4\ \boldsymbol{K_{A\bullet D}{}^2\ EM_d}$ | $2\ \varepsilon_{free} + 2\ \varepsilon_{bound}$ |
| 12 | $2\ \boldsymbol{K_{A\bullet D}{}^{4N}\ EM_d{}^{2N}}$ | $2\ \varepsilon_{free} + 2N\ \varepsilon_{bound}$ |
| 13 | $4\ \boldsymbol{K_{A\bullet D}{}^{4N}\ EM_d{}^{2N}\ K_d}$ | $2\ \varepsilon_{free} + 2N\ \varepsilon_{bound}$ |
| 14 | $2\ \boldsymbol{K_{A\bullet D}{}^{4N}\ EM_d{}^{2N}\ K_d^2}$ | $2\ \varepsilon_{free} + 2N\ \varepsilon_{bound}$ |

[1] The stability of species 6 can either be defined in terms of stepwise effective molarities ($\boldsymbol{EM_{f1}}$ or $\boldsymbol{EM_{f2}}$ for the first intramolecular base-pairs, and $\boldsymbol{EM_f'}$ for the second) or in terms of the cooperativity parameter for formation of the doubly H-bonded species ($\alpha$). The equations in the table define the mathematical relationship between $\boldsymbol{EM_f'}$ and $\alpha$.

# **DAO$_3$AD**•PFTB fitted to folding model

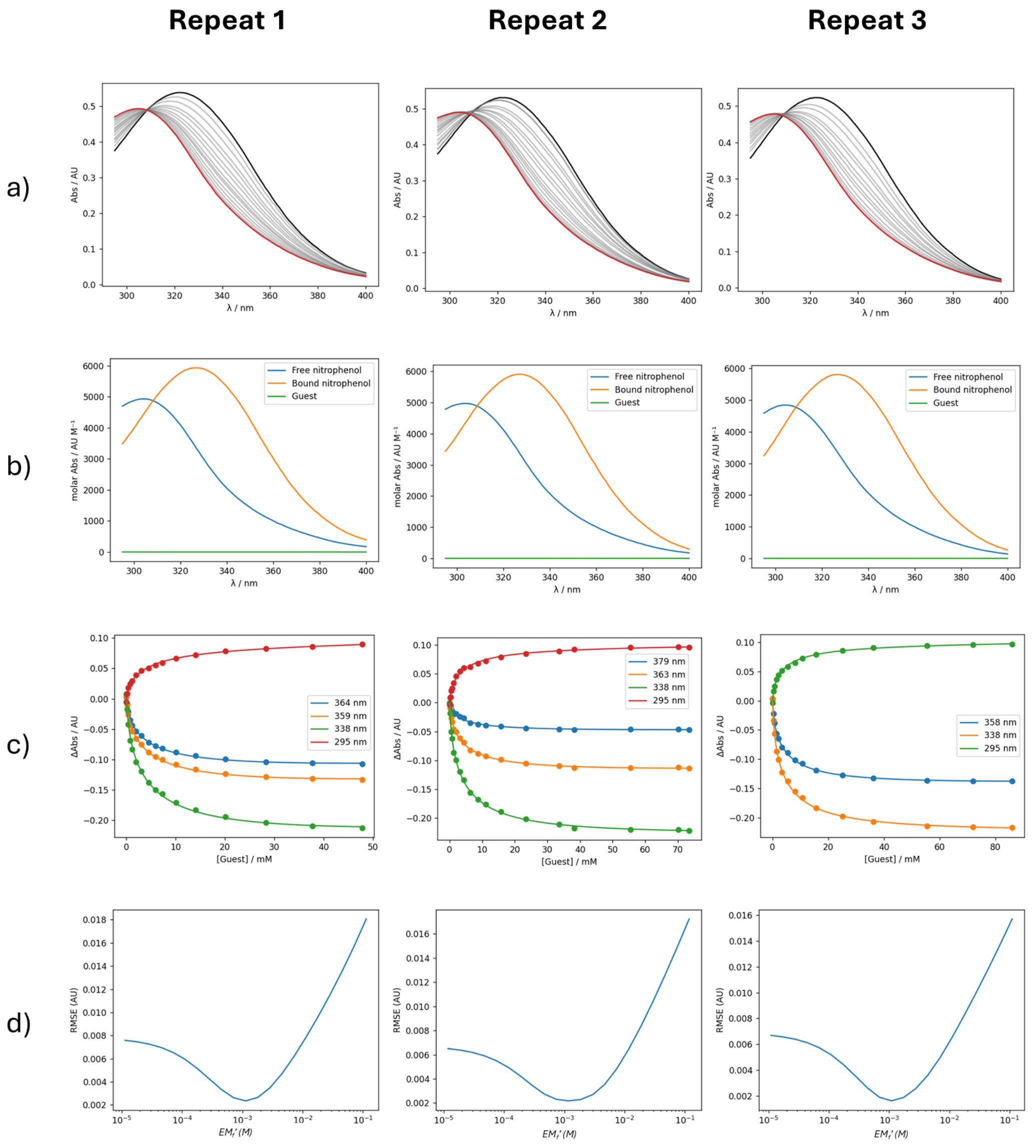


*Figure S 147 UV-Vis absorption denaturation of **DAO$_3$AD** (50 µM) with PFTB (guest) in dichloromethane at 298 K. a) UV-Vis absorption spectra showing the starting spectrum in black and the final spectrum in red. b) Best fit of the change in UV-Vis absorbance at selected wavelengths to an 14-species isotherm as described in the duplex model in Figure S 146 and Table S 6 allowing for guest absorption. c) Fitted spectra of free 4-nitrohenol, bound 4-nitrophenol and PFTB d) Relationship between the RMSE between the experimental data and calculated spectra plotted as a function of the value of **EM$_f$'**.*

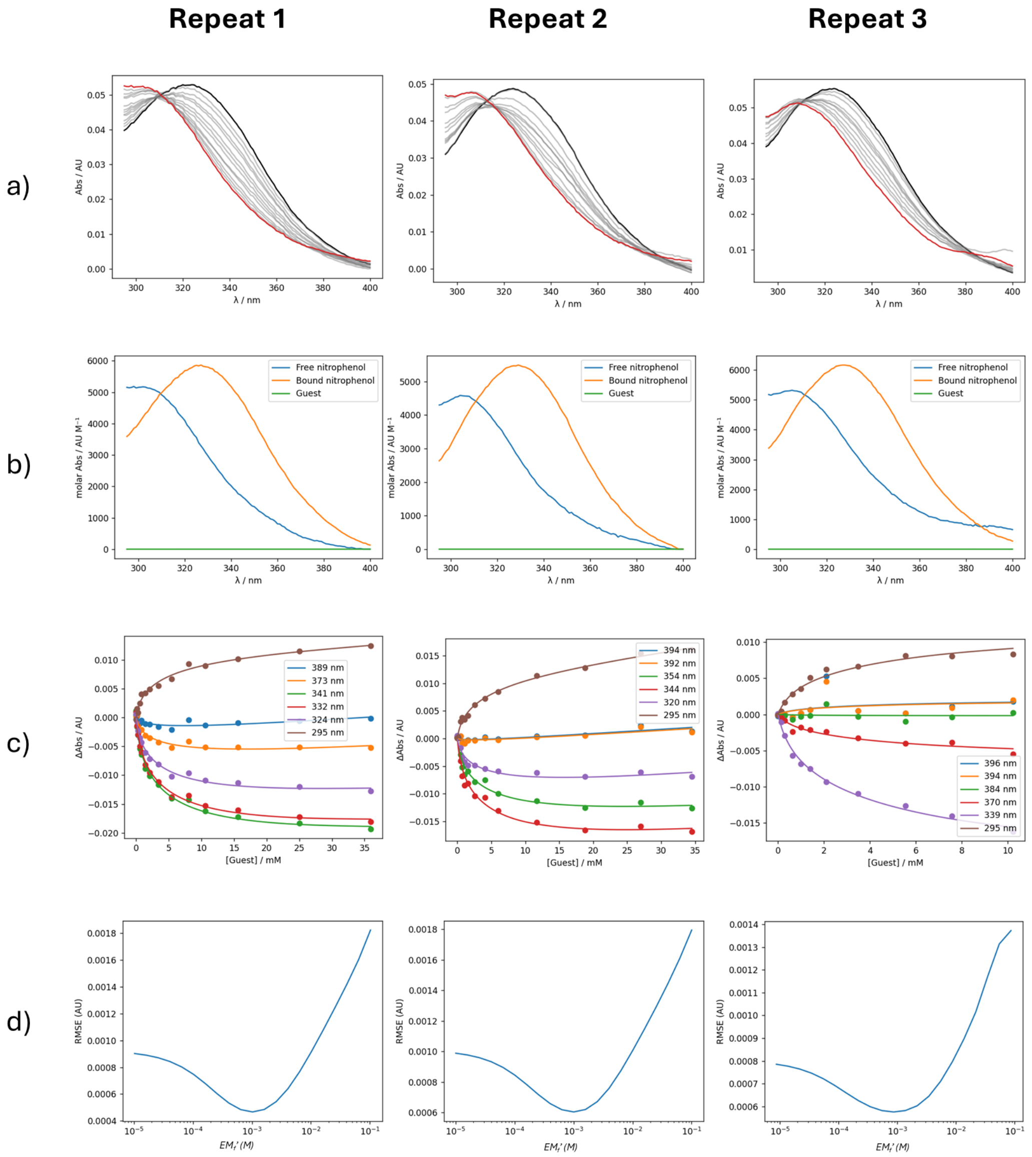


*Figure S 148 UV-Vis absorption denaturation of* ***DAO$_3$AD*** *(5 µM) with PFTB (guest) in dichloromethane at 298 K. a) UV-Vis absorption spectra showing the starting spectrum in black and the final spectrum in red. b) Best fit of the change in UV-Vis absorbance at selected wavelengths to an 14-species isotherm as described in the duplex model in Figure S 146 and Table S 6 allowing for guest absorption. c) Fitted spectra of free 4-nitrohenol, bound 4-nitrophenol and PFTB d) Relationship between the RMSE between the experimental data and calculated spectra plotted as a function of the value of* ***EM$_f$'****.*

*Table S 7 Summary of results of fitting data of the titrations of PFTB into **DAO$_3$AD** to different models.*

| Model | Parameter | [oligomer] = 50 µM | [oligomer] = 5 µM | Conclusions |
| --- | --- | --- | --- | --- |
| duplex | $\boldsymbol{EM_{d2}}$ | 1.4 ± 0.7 mM | 24 ± 1 mM | $\boldsymbol{EM_{d2}}$ should not be concentration-dependent, so the model is incorrect |
| folding | $\boldsymbol{EM_{f}'}$ | 1.1 ± 0.04 mM | 0.9 ± 0.09 mM | $\boldsymbol{EM_{f}'}$ is not concentration-dependent, so the model is correct |

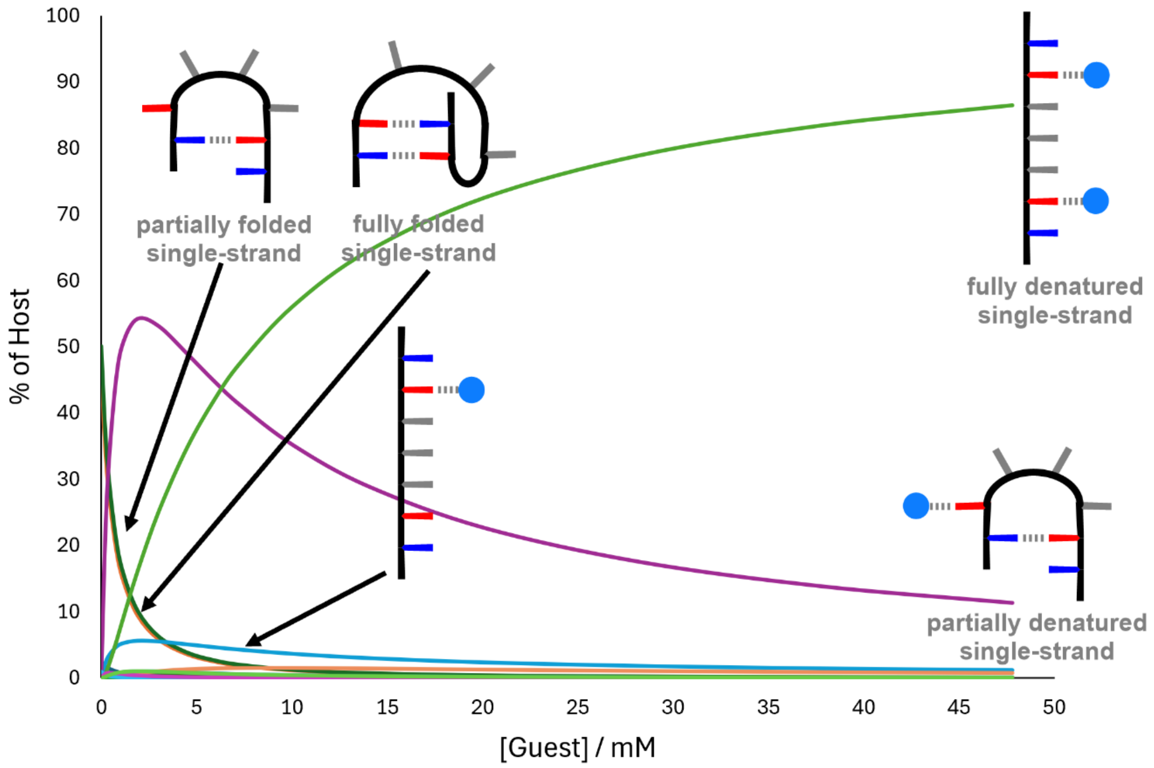


*Figure S 149 Calculated populations of different species containing **DAO$_3$AD** (only populated species shown) using the folding model.*

# **DAO$_4$AD**•PFTB fitted to folding model

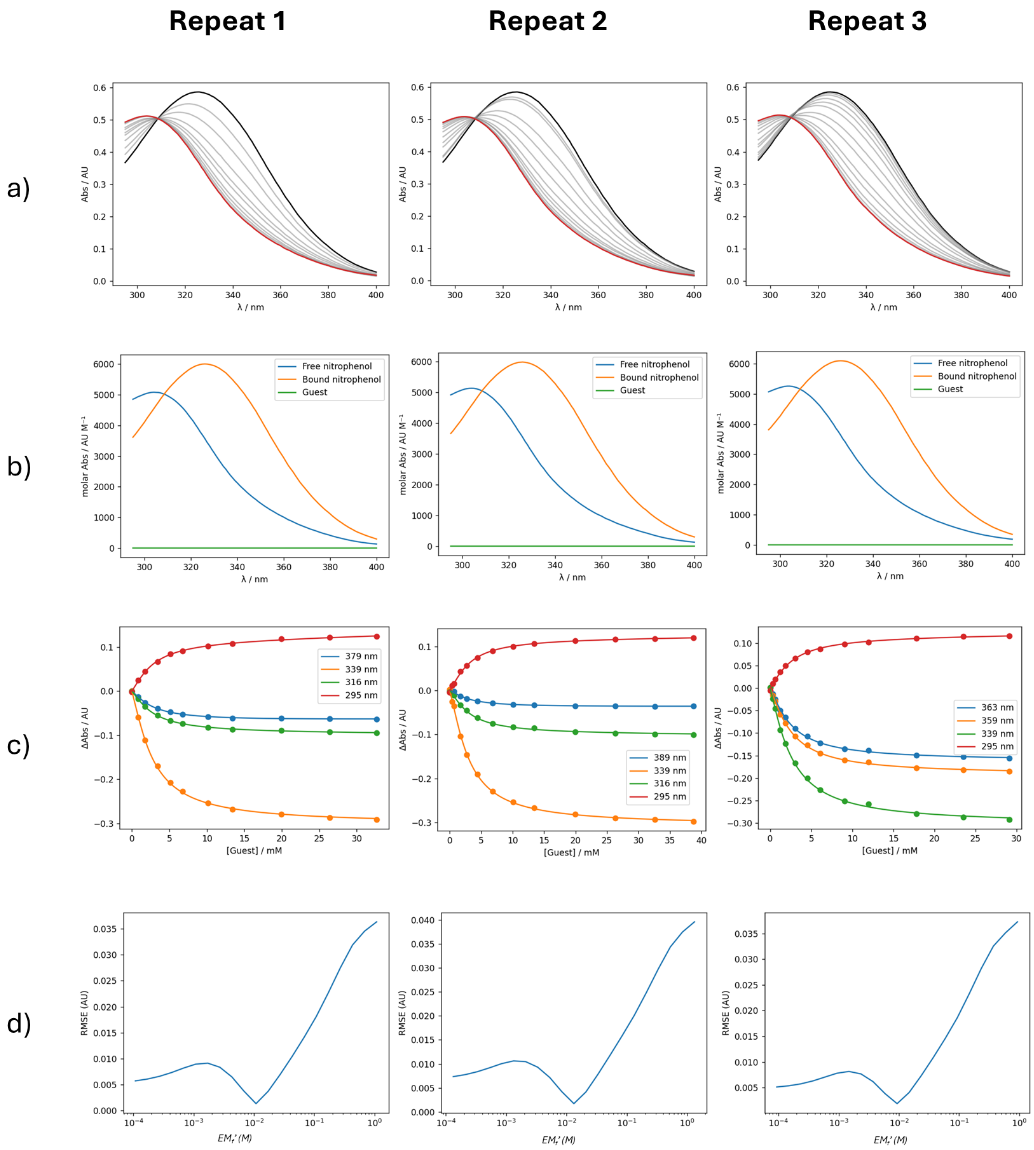


*Figure S 150 UV-Vis absorption denaturation of **DAO$_4$AD** (50 µM) with PFTB (guest) in dichloromethane at 298 K. a) UV-Vis absorption spectra showing the starting spectrum in black and the final spectrum in red. b) Best fit of the change in UV-Vis absorbance at selected wavelengths to an 14-species isotherm as described in the duplex model in Figure S 146 and Table S 6 allowing for guest absorption. c) Fitted spectra of free 4-nitrohenol, bound 4-nitrophenol and PFTB d) Relationship between the RMSE between the experimental data and calculated spectra plotted as a function of the value of **$EM_f$'**.*

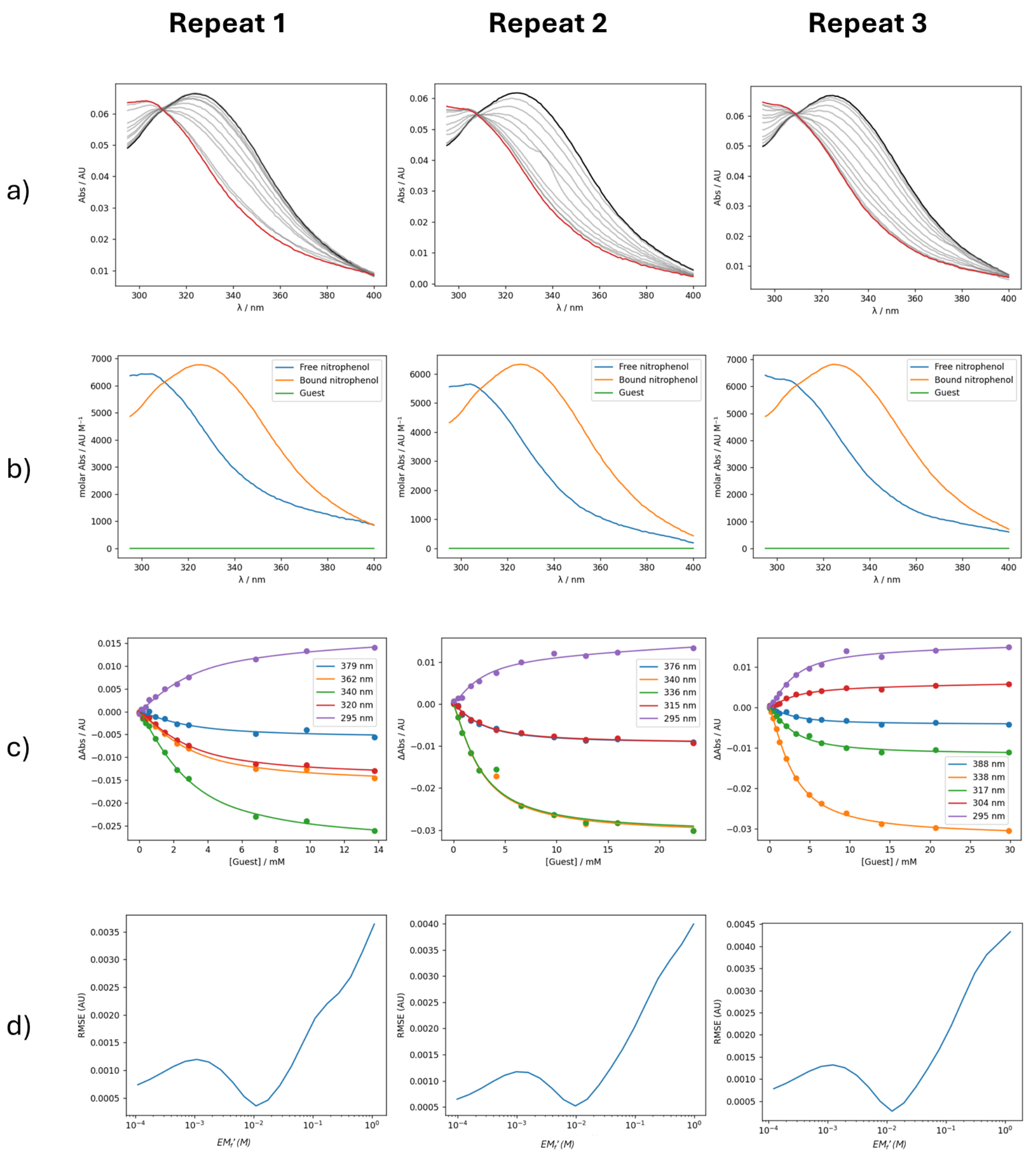


*Figure S 151 UV-Vis absorption denaturation of* ***DAO$_4$AD*** *(5 µM) with PFTB (guest) in dichloromethane at 298 K. a) UV-Vis absorption spectra showing the starting spectrum in black and the final spectrum in red. b) Best fit of the change in UV-Vis absorbance at selected wavelengths to an 14-species isotherm as described in the duplex model in Figure S 146 and Table S 6 allowing for guest absorption. c) Fitted spectra of free 4-nitrohenol, bound 4-nitrophenol and PFTB d) Relationship between the RMSE between the experimental data and calculated spectra plotted as a function of the value of* ***EM$_f$'****.*

*Table S 8 Summary of results of fitting data of the titrations of PFTB into **DAO$_4$AD** to different models.*

| Model | Parameter | [oligomer] = 50 µM | [oligomer] = 5 µM | Conclusions |
|---|---|---|---|---|
| duplex | $EM_{d2}$ | 40 ± 10 mM | 340 ± 90 mM | $EM_{d2}$ should not be concentration-dependent, so the model is incorrect |
| folding | $EM_f$' | 11 ± 2 mM | 11 ± 1 mM | $EM_f$' is not concentration-dependent, so the model is correct |

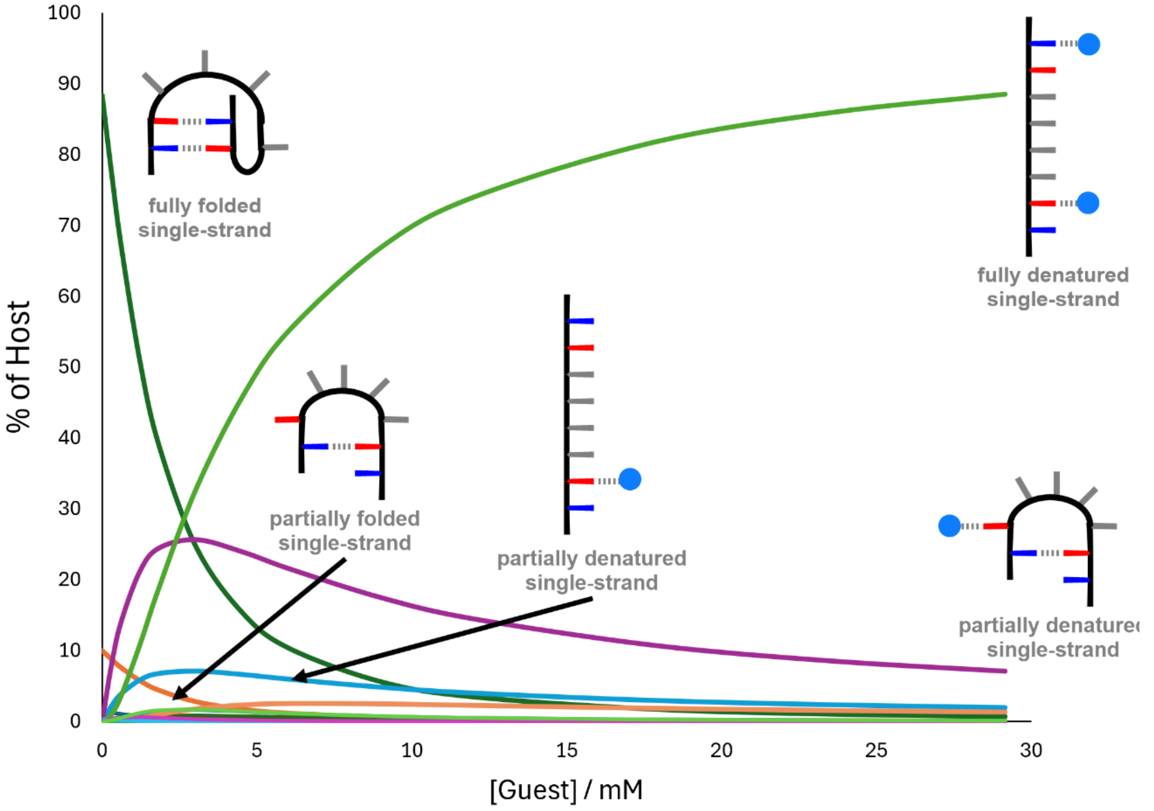


*Figure S 152 Calculated populations of different species containing **DAO$_4$AD** (only populated species shown) using the folding model.*

## **ADO₃AD•**PFTB fitted to folding model

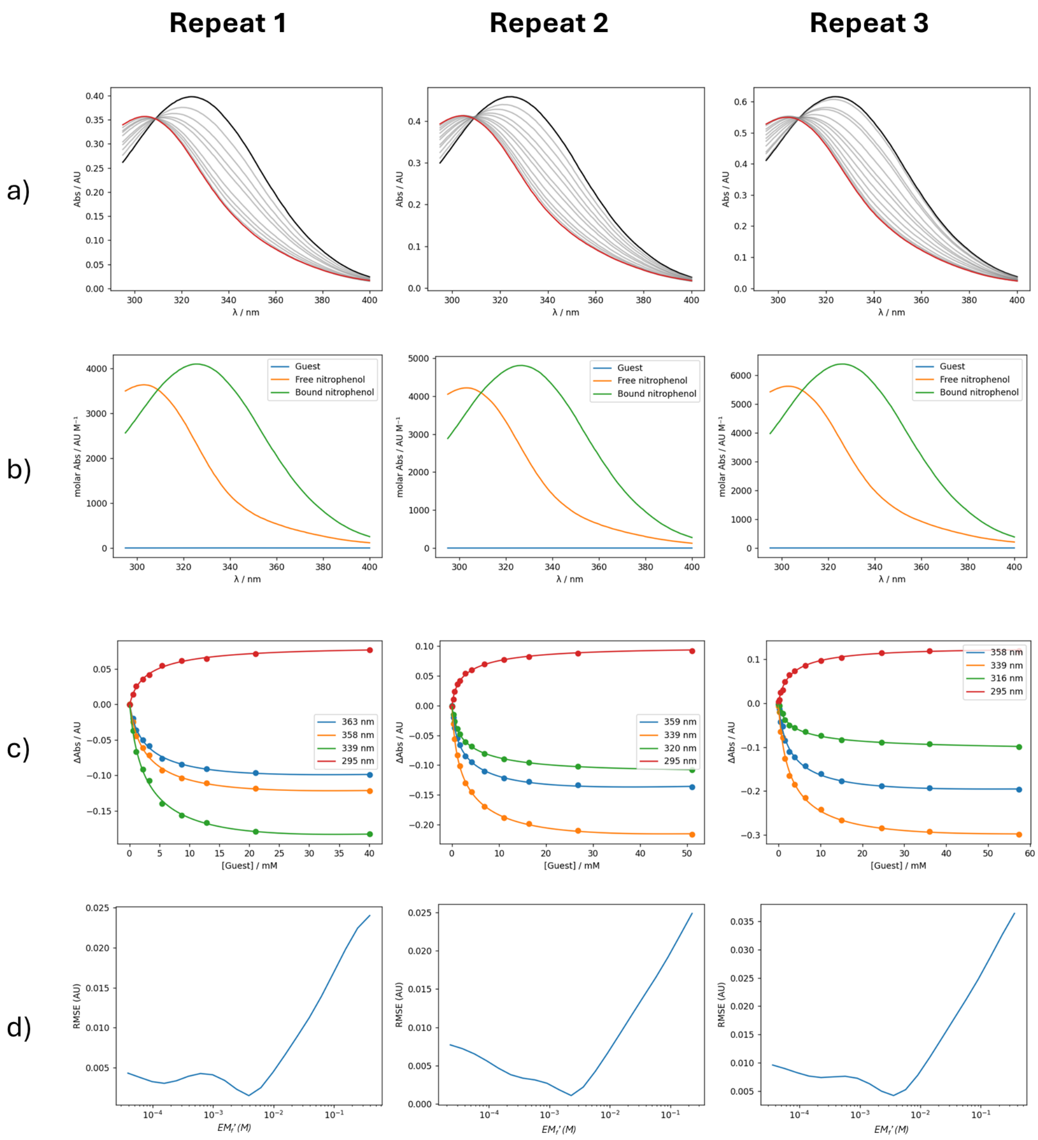


*Figure S 153 UV-Vis absorption denaturation of* ***ADO$_3$AD*** *(50 µM) with PFTB (guest) in dichloromethane at 298 K. a) UV-Vis absorption spectra showing the starting spectrum in black and the final spectrum in red. b) Best fit of the change in UV-Vis absorbance at selected wavelengths to an 14-species isotherm as described in the duplex model in Figure S 146 and Table S 6 allowing for guest absorption. c) Fitted spectra of free 4-nitrohenol, bound 4-nitrophenol and PFTB d) Relationship between the RMSE between the experimental data and calculated spectra plotted as a function of the value of* ***EM$_f$'****.*

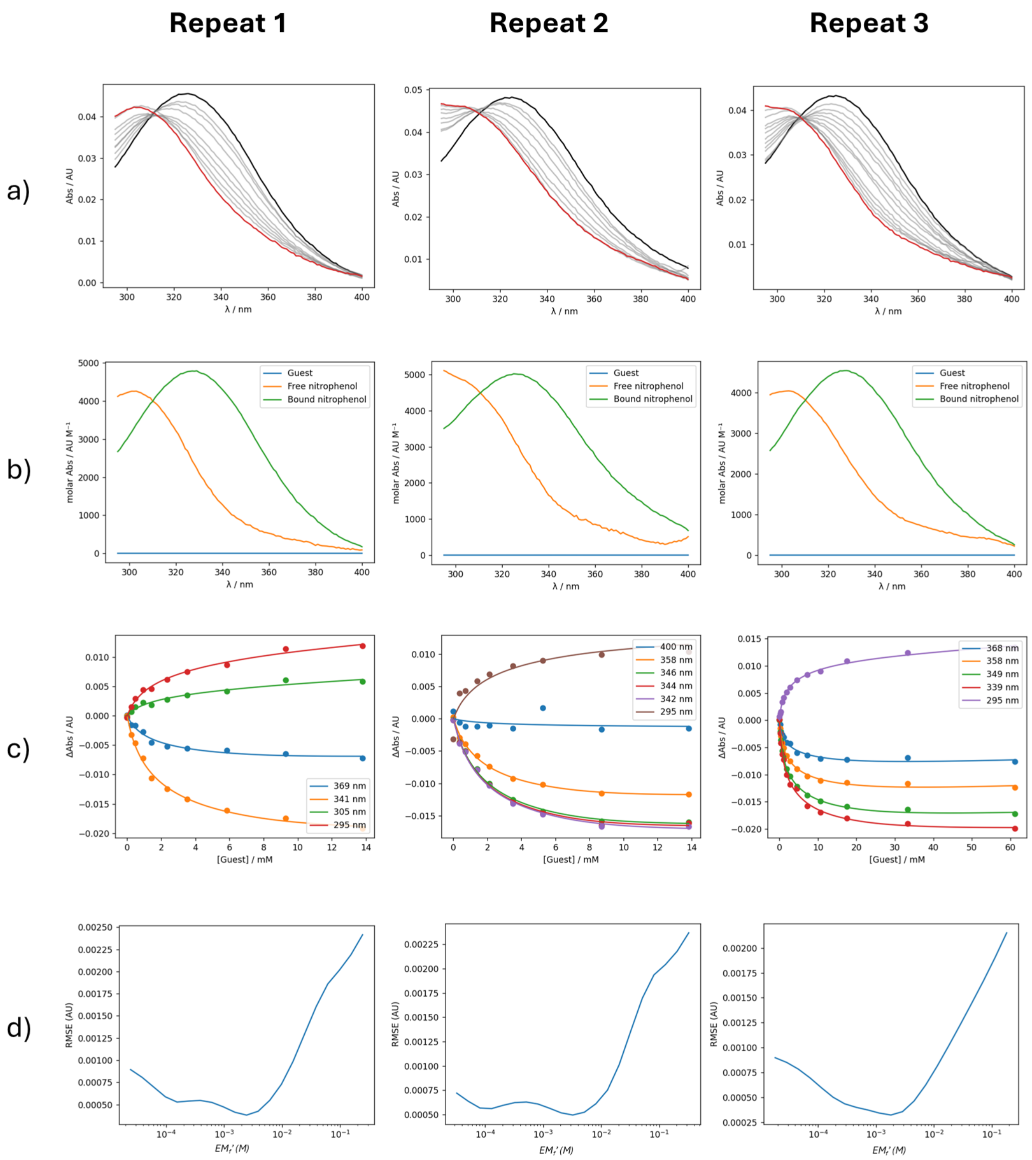


*Figure S 154 UV-Vis absorption denaturation of* ***ADO$_3$AD*** *(5 μM) with PFTB (guest) in dichloromethane at 298 K. a) UV-Vis absorption spectra showing the starting spectrum in black and the final spectrum in red. b) Best fit of the change in UV-Vis absorbance at selected wavelengths to an 14-species isotherm as described in the duplex model in Figure S 146 and Table S 6 allowing for guest absorption. c) Fitted spectra of free 4-nitrohenol, bound 4-nitrophenol and PFTB d) Relationship between the RMSE between the experimental data and calculated spectra plotted as a function of the value of* ***$EM_f$'****.*

*Table S 9 Summary of results of fitting data of the titrations of PFTB into **$ADO_3AD$** to different models*

| Model | Parameter | [oligomer] = 50 µM | [oligomer] = 5 µM | Conclusions |
|---|---|---|---|---|
| duplex | $EM_{d2}$ | 50 ± 20 mM | 220 ± 60 mM | $EM_{d2}$ should not be concentration-dependent, so the model is incorrect |
| folding | $EM_f$' | 3.0 ± 0.9 mM | 2.5 ± 0.7 mM | $EM_f$' is not concentration-dependent, so the model is correct |

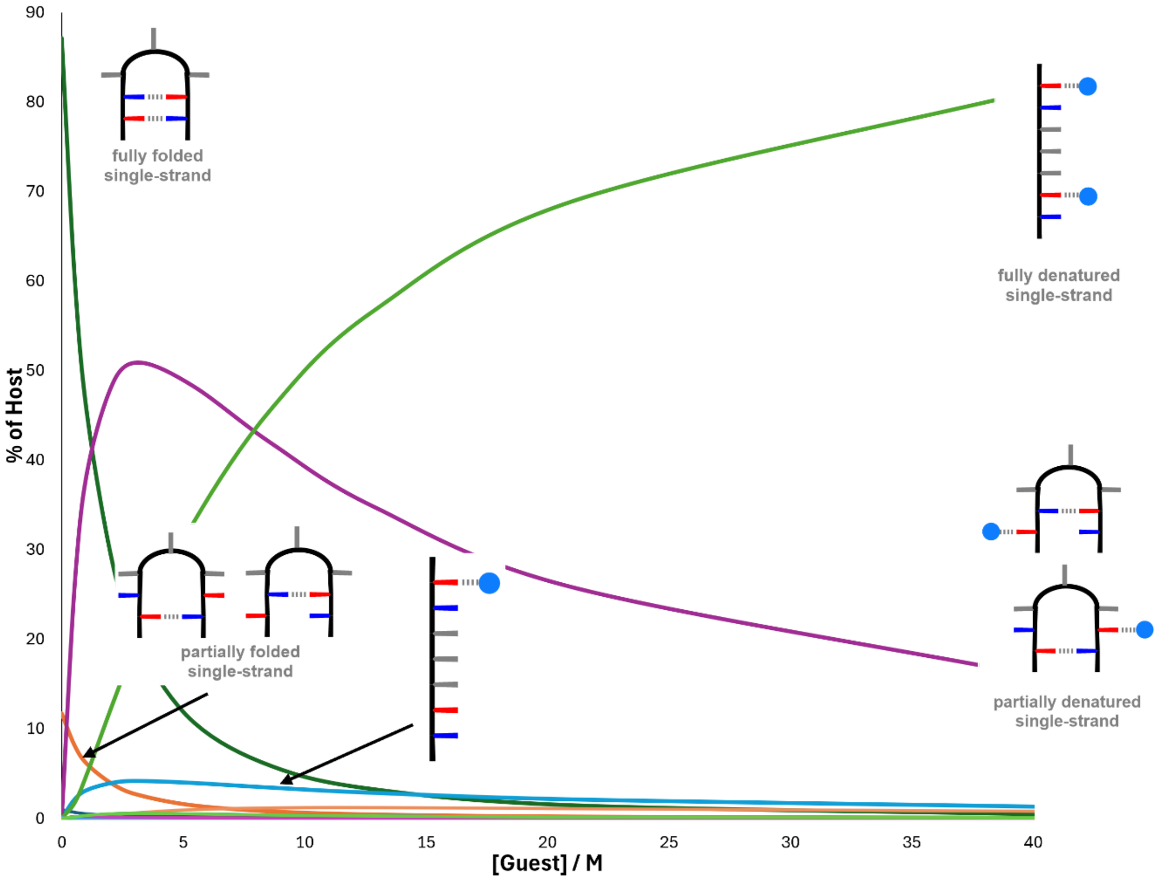


*Figure S 155 Calculated populations of different species containing **$ADO_3AD$** (only populated species shown) using the folding model.*

# **ADO$_4$AD•**PFTB fitted to folding model

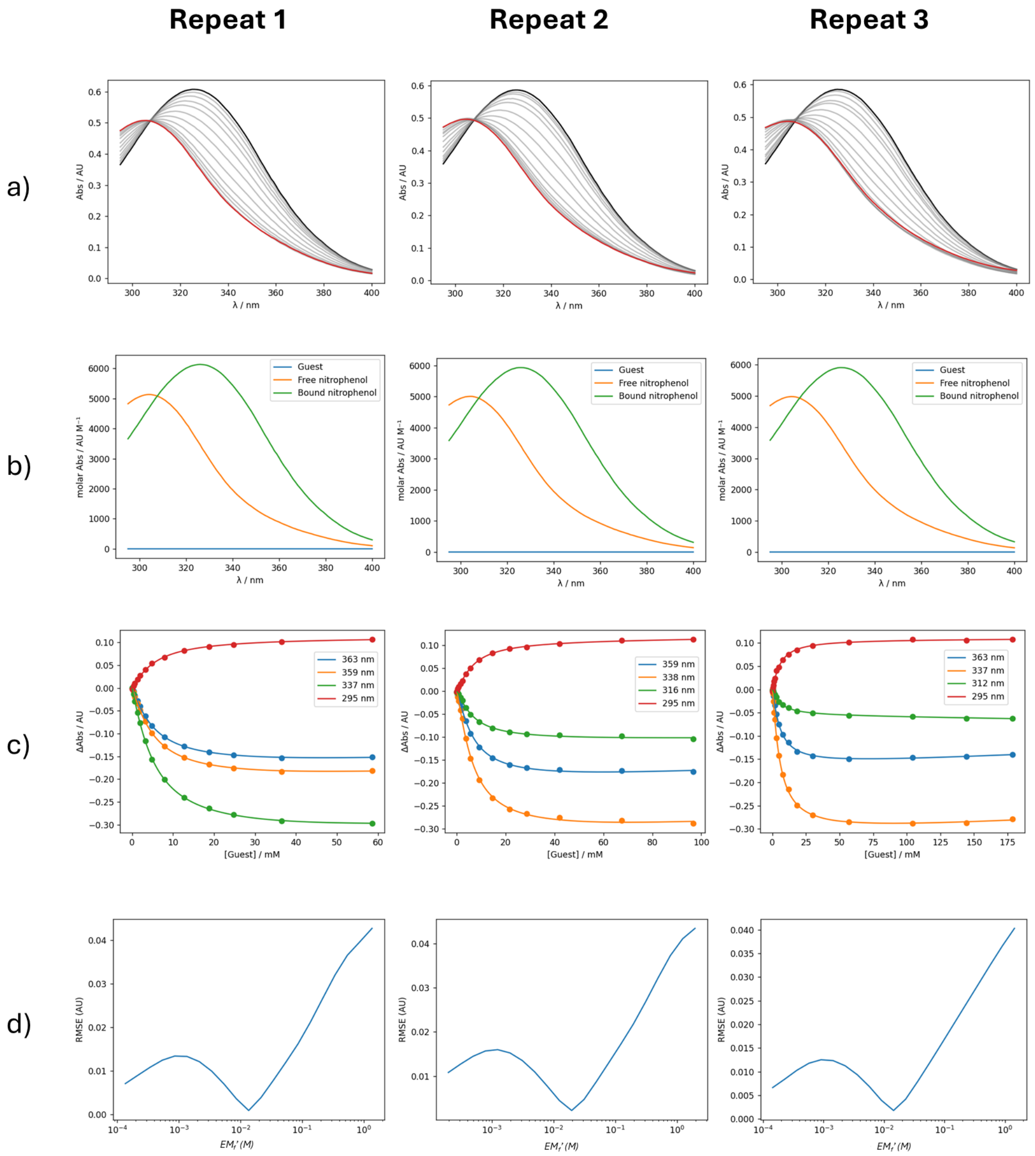


*Figure S 156 UV-Vis absorption denaturation of* ***ADO$_4$AD*** *(50 µM) with PFTB (guest) in dichloromethane at 298 K. a) UV-Vis absorption spectra showing the starting spectrum in black and the final spectrum in red. b) Best fit of the change in UV-Vis absorbance at selected wavelengths to an 14-species isotherm as described in the duplex model in Figure S 146 and Table S 6 allowing for guest absorption. c) Fitted spectra of free 4-nitrohenol, bound 4-nitrophenol and PFTB d) Relationship between the RMSE between the experimental data and calculated spectra plotted as a function of the value of* ***EM$_f$'****.*

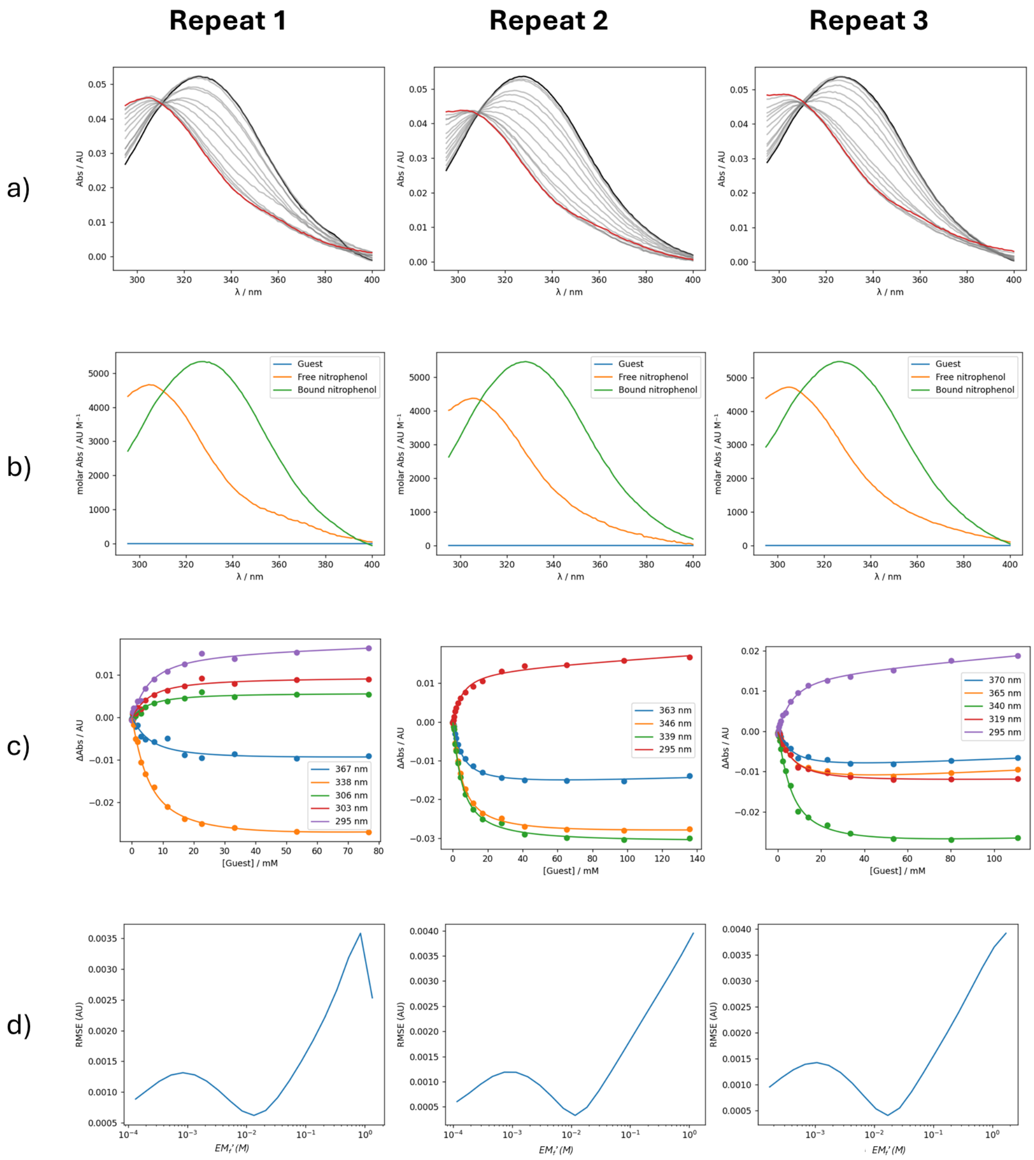


*Figure S 157 UV-Vis absorption denaturation of* $\mathbf{ADO_4AD}$ *(5 µM) with PFTB (guest) in dichloromethane at 298 K. a) UV-Vis absorption spectra showing the starting spectrum in black and the final spectrum in red. b) Best fit of the change in UV-Vis absorbance at selected wavelengths to an 14-species isotherm as described in the duplex model in Figure S 146 and Table S 6 allowing for guest absorption. c) Fitted spectra of free 4-nitrohenol, bound 4-nitrophenol and PFTB d) Relationship between the RMSE between the experimental data and calculated spectra plotted as a function of the value of* $\mathbf{EM_f}$*'.*

*Table S 10 Summary of results of fitting data of the titrations of PFTB into* ***ADO₄AD*** *to different models*

| Model | Parameter | [oligomer] = 50 µM | [oligomer] = 5 µM | Conclusions |
|---|---|---|---|---|
| duplex | $EM_{d2}$ | 0.5 ± 0.1 mM | 4 ± 1 mM | $EM_{d2}$ should not be concentration-dependent, so the model is incorrect |
| folding | $EM_f'$ | 16 ± 3 mM | 14 ± 3 mM | $EM_f'$ is not concentration-dependent, so the model is correct |

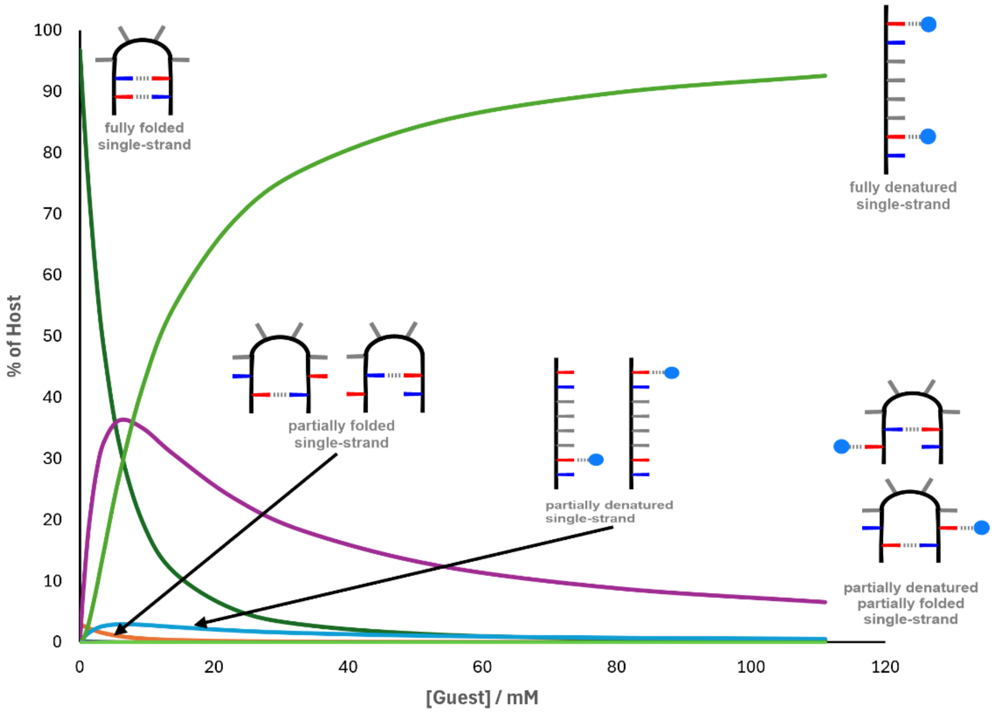


*Figure S 158 Calculated populations of different species containing* ***ADO₄AD*** *(only populated species shown) using the folding model.*

## **ADO$_5$AD•**PFTB fitted to folding model

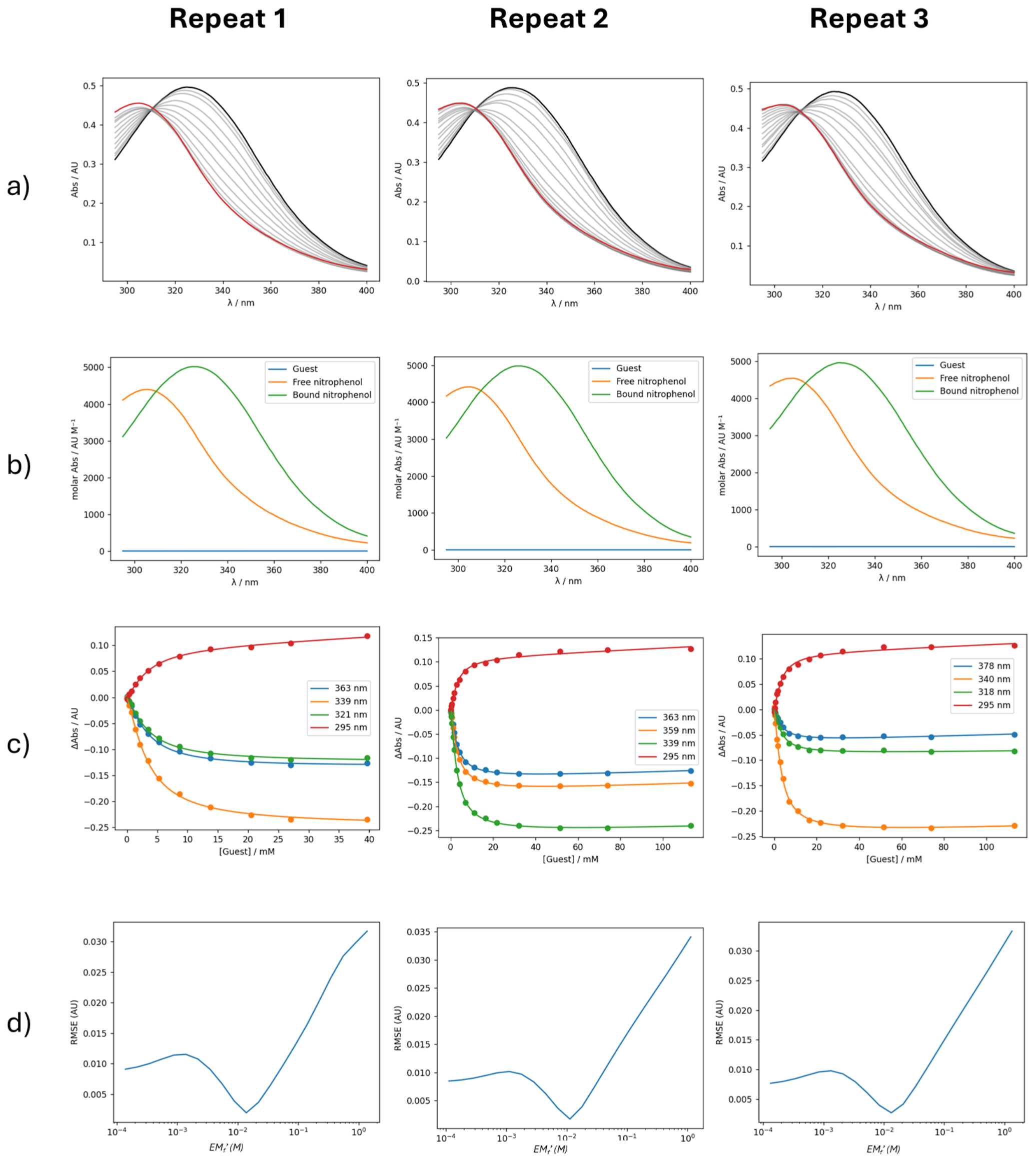


*Figure S 159 UV-Vis absorption denaturation of **ADO$_4$AD** (50 µM) with PFTB (guest) in dichloromethane at 298 K. a) UV-Vis absorption spectra showing the starting spectrum in black and the final spectrum in red. b) Best fit of the change in UV-Vis absorbance at selected wavelengths to an 14-species isotherm as described in the duplex model in Figure S 146 and Table S 5 allowing for guest absorption. c) Fitted spectra of free 4-nitrohenol, bound 4-nitrophenol and PFTB d) Relationship between the RMSE between the experimental data and calculated spectra plotted as a function of the value of **EM$_f$'**.*

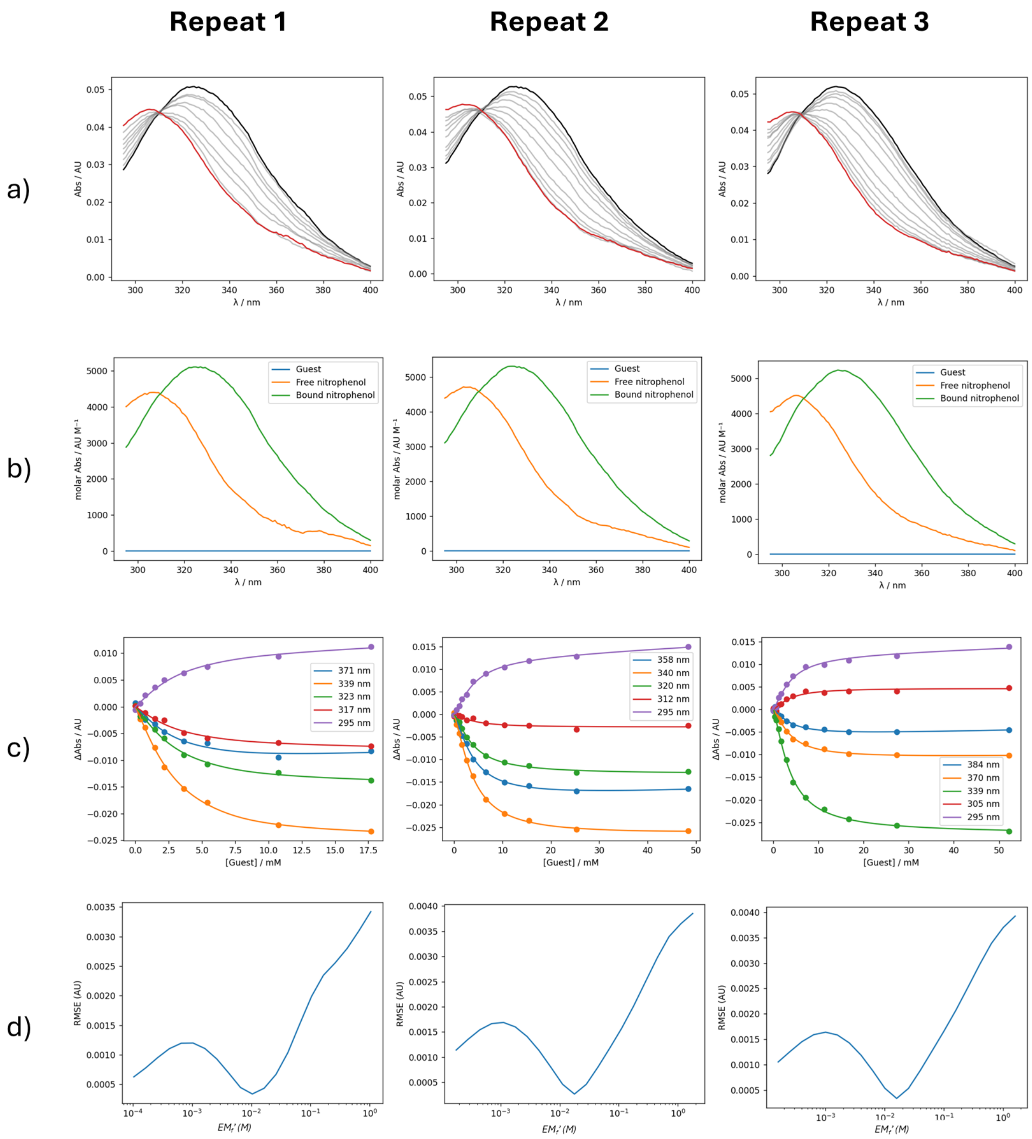


*Figure S 160 UV-Vis absorption denaturation of **ADO$_5$AD** (5 µM) with PFTB (guest) in dichloromethane at 298 K. a) UV-Vis absorption spectra showing the starting spectrum in black and the final spectrum in red. b) Best fit of the change in UV-Vis absorbance at selected wavelengths to an 14-species isotherm as described in the duplex model in Figure S 146 and Table S 6 allowing for guest absorption. c) Fitted spectra of free 4-nitrohenol, bound 4-nitrophenol and PFTB d) Relationship between the RMSE between the experimental data and calculated spectra plotted as a function of the value of **EM$_f$'**.*

*Table S 11 Summary of results of fitting data of the titrations of PFTB into **ADO₅AD** to different models*

| Model | Parameter | [oligomer] = 50 µM | [oligomer] = 5 µM | Conclusions |
|---|---|---|---|---|
| duplex | $\boldsymbol{EM_{d2}}$ | 70 ± 20 mM | 630 ± 370 mM | $\boldsymbol{EM_{d2}}$ should not be concentration-dependent, so the model is incorrect |
| folding | $\boldsymbol{EM_f}$**'** | 13 ± 1 mM | 15 ± 4 mM | $\boldsymbol{EM_f}$**'** is not concentration-dependent, so the model is correct |

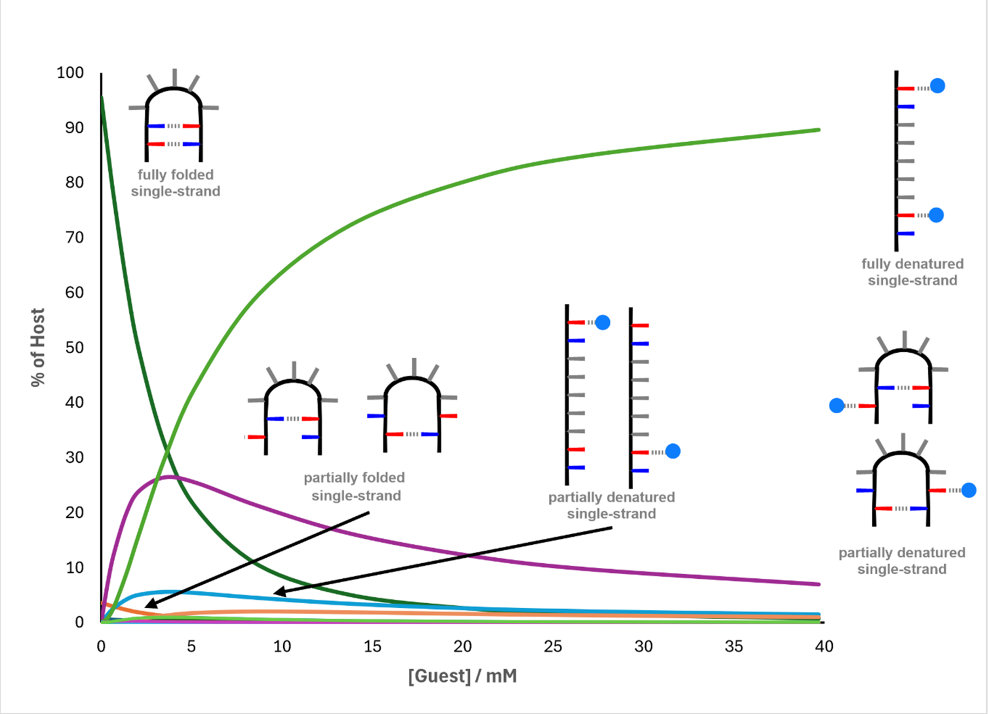


*Figure S 161 Calculated populations of different species containing* ***ADO₅AD*** *(only populated species shown) using the folding model.*

# References

(1) Soloviev, D. O.; Hunter, C. A. Musketeer: A Software Tool for the Analysis of Titration Data. *Chem. Sci.* **2024**.

(2) Troselj, P.; Bolgar, P.; Ballester, P.; Hunter, C. A. High-Fidelity Sequence-Selective Duplex Formation by Recognition-Encoded Melamine Oligomers. *J. Am. Chem. Soc.* **2021**, *143* (23), 8669–8678.

(3) Balduzzi, F.; Munasinghe, V.; Evans, O. N.; Lorusso Notaro Francesco, A.; Anderson, C. J.; Nigrelli, S.; Escobar, L.; Cabot, R.; Smith, J. T.; Hunter, C. A. Length and Sequence-Selective Polymer Synthesis Templated by a Combination of Covalent and Noncovalent Base-Pairing Interactions. *J. Am. Chem. Soc.* **2024**, *146* (47), 32837–32847.

(4) Dhiman, M.; Cabot, R.; Hunter, C. A. Efficient Automated Solid-Phase Synthesis of Recognition-Encoded Melamine Oligomers. *Chem. Sci.* **2024**, *15* (16), 5957–5963.
